# Supplementary material for: Domain mapping of disease mutations reveals pathogenic SORL1 variants in Alzheimer’s disease
Source: Mol Neurodegener. 2025 Dec 1;20:122. doi: 10.1186/s13024-025-00907-z (PMC12667055; doi:10.1186/s13024-025-00907-z)
Supplement: Supplementary file 2 — Supplementary Material 2 [file 13024_2025_907_MOESM2_ESM.pdf]

SUPPLEMENTARY NOTE TO:

Domain Mapping of Disease Mutations Reveals Pathogenic *SORL1* Variants in Alzheimer's Disease

Author list

Olav M. Andersen[1,*,#]; Matthijs W. J. de Waal[2,3,*]; Giulia Monti[1]; Niccolo Tesi[2,3,4,5]; Anne Mette G. Jensen[1]; Christa de Geus[6]; Rosalina van Spaendonk[7]; Maartje Vogel[7]; Shahzad Ahmad[8,9]; Najaf Amin[8,10]; Philippe Amouyel[11]; Gary W. Beecham[12]; Céline Bellenguez[11]; Claudine Berr[13]; Joshua C. Bis[14]; Anne Boland[15]; Paola Bossù[16]; Femke Bouwman[3,4]; Jose Bras[17,18]; Camille Charbonnier[19]; Jordi Clarimon[20,21]; Carlos Cruchaga[22,23,24]; Antonio Daniele[25]; Jean-François Dartigues[26]; Stéphanie Debette[26,27]; Jean-François Deleuze[15]; Nicola Denning[28]; Anita L. DeStefano[29,30,31]; Oriol Dols-Icardo[20,21]; Cornelia M. van Duijn[8,10]; Lindsay A. Farrer[29,30,31,32,33]; Maria Victoria Fernández[22,23,24]; Wiesje M. van der Flier[3,4]; Nick C. Fox[34,35]; Daniela Galimberti[36,37]; Emmanuelle Genin[38]; Johan J.P. Gille[7]; Benjamin Grenier-Boley[11]; Detelina Grozeva[39]; Yann Le Guen[40]; Rita Guerreiro[17,18]; Jonathan L. Haines[41]; Clive Holmes[42]; Holger Hummerich[43]; M. Arfan Ikram[8]; M. Kamran Ikram[8]; Amit Kawalia[44]; Robert Kraaij[45]; Jean-Charles Lambert[11]; Marc Lathrop[46]; Afina W. Lemstra[3,4]; Alberto Lleó[20,21]; Richard M. Myers[47]; Marcel M. A. M. Mannens[48]; Rachel Marshall[39]; Eden R. Martin[12,49]; Carlo Masullo[50]; Richard Mayeux[51,52]; Simon Mead[43]; Patrizia Mecocci[53,54]; Alun Meggy[28]; Merel O. Mol[55]; Benedetta Nacmias[56,57]; Adam C. Naj[58,59]; Valerio Napolioni[40,60]; J. Nicholas Cochran[47]; Gaël Nicolas[19]; Florence Pasquier[61]; Pau Pastor[62,63]; Margaret A. Pericak-Vance[12,49]; Yolande A. L. Pijnenburg[3,4]; Fabrizio Piras[64]; Olivier Quenez[19]; Alfredo Ramirez[44,65,66,67,68]; Rachel Raybould[28]; Richard Redon[69]; Marcel J.T. Reinders[5]; Anne-Claire Richard[19]; Steffi G Riedel-Heller[70]; Fernando Rivadeneira[45]; Jeroen G. J. van Rooij[45,55]; Stéphane Rousseau[19]; Natalie S. Ryan[34,35]; Pascual Sanchez-Juan[21,71]; Gerard D. Schellenberg[58]; Philip Scheltens[3,4]; Jonathan M. Schott[34,35]; Sudha Seshadri[30,31,66]; Daoud Sie[7]; Rebecca Sims[39]; Erik A. Sistermans[2,48]; Sandro Sorbi[56,57]; John C. van Swieten[55]; Betty Tijms[3,4]; André G. Uitterlinden[45]; Pieter Jelle Visser[3,4]; Michael Wagner[67,68]; David Wallon[19]; Li-San Wang[58]; Julie Williams[39,72]; Jennifer S. Yokoyama[73]; Aline Zarea[19]; Sven J. van der Lee[2,3,4]; Johan G. Olsen[74]; Marc Hulsman[2,3,4,5]; Henne Holstege[2,3,4,5,*,#]

*Authors contributed equally to this work.

Corresponding Author:

Henne Holstege, h.holstege@amsterdamumc.nl

Olav M. Andersen, o.andersen@biomed.au.dk

1. Dept. of Biomedicine, Aarhus University, Denmark.
2. Department of Human Genetics, Amsterdam UMC, location Vrije Universiteit Amsterdam, Amsterdam, The Netherlands.
3. Amsterdam Neuroscience, Neurodegeneration, Amsterdam, The Netherlands.
4. Alzheimer Center Amsterdam, Neurology, Amsterdam UMC, location Vrije Universiteit Amsterdam, Amsterdam, The Netherlands.
5. Delft Bioinformatics Lab, Delft University of Technology, Delft, The Netherlands.
6. Clinical Genetics, Human Genetics, Amsterdam UMC, Amsterdam, The Netherlands.
7. Genome Analysis Laboratory, Human Genetics, Amsterdam UMC, location Vrije Universiteit Amsterdam, the Netherlands.
8. Department of Epidemiology, Erasmus Medical Centre, Rotterdam, The Netherlands.
9. LACDR, Leiden, The Netherlands.
10. Nuffield Department of Population Health Oxford University.
11. Univ. Lille, Inserm, CHU Lille, Institut Pasteur Lille, LabEx DISTALZ-U1167-RID-AGE - Facteurs de risque et déterminants moléculaires des maladies liées au vieillissement, Lille, France.
12. The John P. Hussman Institute for Human Genomics, University of Miami, Miami, Florida, USA.
13. Univ Montpellier, Inserm, INM (Institute for Neurosciences of Montpellier), Montpellier, France
14. Cardiovascular Health Research Unit, Department of Medicine, University of Washington, Seattle, WA, USA.
15. Université Paris-Saclay, CEA, Centre National de Recherche en Génomique Humaine Evry, France.
16. Experimental Neuro-psychobiology Laboratory, Department of Clinical and Behavioral Neurology, IRCCS Santa Lucia Foundation, Rome, Italy.
17. Division of Psychiatry and Behavioral Medicine, Michigan State University College of Human Medicine, Grand Rapids, MI, USA.
18. Department of Neurodegenerative Science, Van Andel Institute, Grand Rapids, MI, USA.
19. Univ Rouen Normandie, Normandie Univ, Inserm U1245 and CHU Rouen, Departments of Genetics and CNRMAJ, F-76000 Rouen, France.
20. Memory Unit, Neurology Department and Institut de Recerca Sant Pau, Hospital de la Santa Creu i Sant Pau, Universitat Autònoma de Barcelona, Sant Quintí 77-79, 08041, Barcelona, Spain.
21. CIBERNED, Network Center for Biomedical Research in Neurodegenerative Diseases, National Institute of Health Carlos III, Madrid, Spain.
22. Psychiatry Department, Washington University School of Medicine, St Louis, MO, USA.
23. Neurogenomics and Informatics Center, Washington University School of Medicine, St Louis, MO, USA
24. Hope Center for Neurological Disorders, Washington University School of Medicine, St Louis, MO, USA.
25. Department of Neuroscience, Catholic University of Sacred Heart, Fondazione Policlinico Universitario A. Gemelli IRCCS, Rome, Italy.
26. University Bordeaux, Inserm, Bordeaux Population Health Research Center, France.
27. Department of Neurology, Bordeaux University Hospital, Bordeaux, France.
28. UKDRI at Cardiff, School of Medicine, Cardiff University, Cardiff, UK.
29. Department of Biostatistics, Boston University School of Public Health, Boston, MA, USA.
30. Framingham Heart Study, Framingham, MA, USA.
31. Department of Neurology, Boston University School of Medicine, Boston, MA, USA.
32. Department of Medicine (Biomedical Genetics), Boston University, Boston, MA, USA.
33. Department of Epidemiology, Boston University, Boston, MA, USA.
34. UK Dementia Research Institute at UCL, London, UK.
35. Dementia Research Centre, UCL Queen Square Institute of Neurology, London, UK.
36. University of Milan, Milan, Italy.
37. Fondazione IRCCS Ca' Granda, Ospedale Policlinico, Milan, Italy.
38. Univ Brest, Inserm, EFS, CHU Brest, UMR 1078, GGB, F-29200, Brest, France.
39. Division of Psychological Medicine and Clinical Neuroscience, School of Medicine, Cardiff University, Cardiff, UK.
40. Quantitative Sciences Unit, Department of Medicine, Stanford University, Stanford, CA, USA.
41. Department of Population and Quantitative Health Sciences, School of Medicine, Case Western Reserve University, Cleveland, Ohio, USA.

42. Clinical and Experimental Science, Faculty of Medicine, University of Southampton, Southampton, UK.
43. MRC Prion Unit at UCL, UCL Institute of Prion Diseases, London, UK.
44. Division of Neurogenetics and Molecular Psychiatry, Department of Psychiatry and Psychotherapy, Faculty of Medicine and University Hospital Cologne, University of Cologne, Cologne Germany
45. Department of Internal Medicine, Erasmus Medical Centre, Rotterdam, The Netherlands.
46. McGill University and Genome Quebec Innovation Centre, Montreal, QC, Canada.
47. HudsonAlpha Institute for Biotechnology, Huntsville, AL, USA.
48. Department of Human Genetics, Amsterdam UMC, University of Amsterdam, Amsterdam Reproduction and Development Research Institute Amsterdam, The Netherlands.
49. Dr. John T. Macdonald Foundation Department of Human Genetics, University of Miami, Miami, Florida, USA.
50. Institute of Neurology, Catholic University of the Sacred Heart, Rome, Italy.
51. Taub Institute on Alzheimer's Disease and the Aging Brain, Department of Neurology, Columbia University, New York, New York, USA.
52. Gertrude H. Sergievsky Center, Columbia University, New York, NY, USA.
53. Division of Gerontology and Geriatrics, Department of Medicine and Surgery, University of Perugia, Perugia, Italy.
54. Division of Clinical Geriatrics, Department of Neurobiology, Care Sciences and Society, Karolinska Institutet, Stockholm, Sweden.
55. Department of Clinical Genetics, Erasmus Medical Center, Rotterdam, The Netherlands.
56. IRCCS Fondazione Don Carlo Gnocchi, Florence, Italy.
57. Department of Neuroscience, Psychology, Drug Research and Child Health University of Florence, Florence, Italy.
58. Penn Neurodegeneration Genomics Center, Department of Pathology and Laboratory Medicine, University of Pennsylvania Perelman School of Medicine, Philadelphia, PA, USA.
59. Penn Neurodegeneration Genomics Center, Department of Biostatistics, Epidemiology, and Informatics; University of Pennsylvania Perelman School of Medicine, Philadelphia, PA, USA.
60. Genomic And Molecular Epidemiology (GAME) Lab, School of Biosciences and Veterinary Medicine, University of Camerino (UNICAM) Camerino, 62032, Italy.
61. Univ. Lille, Inserm, CHU Lille, UMR1172, Resources and Research Memory Center (MRRC) of Distalz, Licend, Lille France
62. Unit of Neurodegenerative diseases, Department of Neurology, University Hospital Germans Trias i Pujol, Badalona, Barcelona, Spain.
63. The Germans Trias i Pujol Research Institute (IGTP), Badalona, Barcelona, Spain.
64. Laboratory of Neuropsychiatry, Department of Clinical and Behavioral Neurology, IRCCS Santa Lucia Foundation, Rome, Italy.
65. Cluster of Excellence Cellular Stress Responses in Aging-Associated Diseases (CECAD), University of Cologne, Cologne, Germany.
66. Department of Psychiatry and Glenn Biggs Institute for Alzheimer’s and Neurodegenerative Diseases, San Antonio, TX, USA.
67. German Center for Neurodegenerative Diseases (DZNE, Bonn), Bonn, Germany.
68. Department of Neurodegenerative Diseases and Geriatric Psychiatry, University Hospital Bonn, Medical Faculty, Bonn, Germany.
69. Université de Nantes, CHU Nantes, CNRS, INSERM, l'institut du thorax, Nantes, France.
70. Institute of Social Medicine, Occupational Health and Public Health, University of Leipzig, Leipzig, Germany.
71. Neurology Service, Marqués de Valdecilla University Hospital (University of Cantabria and IDIVAL), Santander, Spain.
72. MRC UK Dementia Research Institute, Division of Psychological Medicine, Cardiff University, Cardiff, UK.
73. Memory and Aging Center, Department of Neurology Weill Institute for Neurosciences, and Department of Radiology and Biomedical Imaging, University of California, San Francisco, CA, USA.
74. Department of Biology, University of Copenhagen, Ole Maaløes Vej 5, DK2200, Copenhagen, Denmark.

INDEX

1 SUPPLEMENTARY DATA **8**

1.1 Sample Description **8**

1.1.1 ADES-FR 8

1.1.2 AgeCoDe-UKBonn 10

1.1.3 Barcelona- SPIN 11

1.1.4 AC-EMC 11

1.1.5 ERF 12

1.1.6 Rotterdam Study 12

1.1.7 ADC-Amsterdam 13

1.1.8 Netherlands Brain Bank 13

1.1.9 Amsterdam-UMC 13

1.1.10 100-plus Study 14

1.1.11 EMIF-AD 90-plus Study 14

1.1.12 CBC: Control Brain Consortium 14

1.1.13 PERADES 15

1.1.14 StEP-AD 16

1.1.15 Knight-ADRC 16

1.1.16 UCSF/NYGC/UAB 17

1.1.17 UCL-DRC EOAD 17

1.1.18 ADSP 18

1.1.19 Variant calling 19

1.2 Effects of non-HPV and non-PTV rare *SORL1* variants **19**

1.2.1 Effects of rare *SORL1* variants with moderate priority: MPVs 19

1.2.2 Effects of rare *SORL1* variants with low and no priority: LPVs and NPVs 20

1.2.3 Association of variants with MAF>0.05% with AD 21

1.3 Comparison of DMDM with REVEL score and AlphaMissense. **22**

2 SUPPLEMENTARY FIGURES AND TABLES **24**

2.1 Supplementary Figures 24
2.1.1 Fig S1. PCA population 24
2.1.2 Fig S2. Sensitivity Analysis of *SORL1* Variant Associations in European and Non-European Populations. 25
2.1.3 Fig S3: The effects of MPVs, LPVs and NPVs in context of APOE. 26
2.1.4 Fig S4: Age at onset for specific variant carriers per priority group in context of APOE-genotype. 27
2.1.5 Figure S5. Age at onset annotated by APOE genotype. 28
2.1.6 Figure S6: Comparison of DMDM with REVEL score and AlphaMissense 29

2.2 Supplemental Tables: 30
2.2.1 Table S1. XLS FILE: Rare variants (MAF < 0.05%) 30
2.2.2 Table S2. XLS FILE: Non-rare variants (MAF > 0.05%) 30
2.2.3 Table S3. XLS FILE: All excluded variants 30
2.2.4 Table S4: List of high-priority variants based on DMDM analysis and sequence conservation. 31
2.2.5 Table S5: Moderate-priority variants 32
2.2.6 Table S6. Associations of priority groups using logistic regression 33
2.2.7 Table S7. Age at onset per variant carrier group relative to WT *SORL1* carriers 34
2.2.8 Table S8. Differences in age at onset per priority category in context of APOE genotype. 35

3 COMPENDIUM 36

3.1 The VPS10p-domain (residues 1-753) 36
3.1.1 Sequence details 36
3.1.2 Supplemental Figure S7. VPS10p-domain 40
3.1.3 *SORL1* variants in VPS10p-domain 41

3.2 The YWTD-repeated β-propeller (residues 754-1013) 42
3.2.1 Sequence details 42
3.2.2 Supplemental Figure S8. YWTD-domain 46
3.2.3 *SORL1* variants in YWTD-domain 47
3.2.4 YWTD-domain alignment 50
3.2.5 Identity of mapped YWTD variants: disease proteins and disease variants 53
3.2.6 YWTD disease variants listed according to domain positions 56

3.3 The EGF-domain (residues 1014-1074) 58
3.3.1 Sequence details 58
3.3.2 Supplemental Figure S9. EGF-domain 61
3.3.3 *SORL1* variants in EGF-domain 62
3.3.4 EGF-domain alignment 63

3.4 **The CR-cluster (residues 1075-1550)** **65**
3.4.1 Sequence details 65
3.4.2 Supplemental Figure S10. CR-domains 69
3.4.3 *SORL1* variants in CR-domains 70
3.4.4 CR-domain alignment 74
3.4.5 Identity of mapped CR variants: disease proteins and disease variants 76
3.4.6 CR disease variants listed according to domain positions 78

3.5 **The 3Fn-cassette (residues 1551-2121)** **80**
3.5.1 Sequence details 80
3.5.2 Supplemental Figure S11. 3Fn-domains 85
3.5.3 *SORL1* variants in 3Fn-domains 86
3.5.4 3Fn-domain alignment 88
3.5.5 Identity of mapped 3Fn variants: disease proteins and disease variants 97
3.5.6 3Fn disease variants listed according to domain positions 102

3.6 **Transmembrane and cytoplasmic domains (residues 2122-2214)** **106**
3.6.1 Sequence details 106
3.6.2 Supplemental Figure S12. Transmembrane and cytoplasmic domains 110
3.6.3 *SORL1* variants in transmembrane and tail-domains 111

3.7 **SORL1 sequences for alignment: species conservation** **112**

3.8 **SORL1 sequence alignment** **114**

3.9 **Phylogenetic tree for SORL1** **127**
3.9.1 Supplemental Figure S13. Phylogenetic tree 129

3.10 **Supplemental methods** **130**
3.10.1 SORL1 DOMAIN SEQUENCE ALIGNMENTS 130
3.10.2 SORL1-SPECIFIC DISEASE-MUTATION DOMAIN-MAPPING 131
3.10.3 PHYLOGENETIC TREE GENERATION: 132
3.10.4 CONSERVATION ANALYSIS: ALIGNMENT OF 40 REPRESENTATIVE SORL1 SEQUENCES: 133

3.11 **Putting the pieces together - the conformational space** **134**

3.12 **Supplemental Figure S14. The SORL1 conformational space** **139**

4 ACKNOWLEDGMENTS **140**

4.1 Study participants, personnel, and compute infrastructure **140**
4.1.1 Participants 140
4.1.2 SURF supercomputer facility 140

4.2 Study Cohorts **140**
4.2.1 ADES-FR 140
4.2.2 AgeCoDe-UKBonn 141
4.2.3 Barcelona- SPIN 141
4.2.4 AC-EMC 141
4.2.5 ERF 141
4.2.6 Rotterdam Study 141
4.2.7 ADC-Amsterdam 142
4.2.8 100-plus Study 143
4.2.9 EMIF-AD 90+ 143
4.2.10 CBC: Control Brain Consortium 143
4.2.11 PERADES 143
4.2.12 StEP-AD cohort 145
4.2.13 Knight-ADRC 145
4.2.14 UCSF/NYGC/UAB 145
4.2.15 UCL-DRC EOAD 145
4.2.16 ADSP 145

4.3 Supplementary Authors **150**
4.3.1 PERADES Cohort: 150
4.3.2 StEP AD investigators 151
4.3.3 Knight ADRC investigators 151
4.3.4 ADNI database 151

5 REFERENCES **152**

1 Supplementary Data

1.1 Sample Description

We analyzed a total sample of 52,361 individuals (40,852 after QC) sequenced with Illumina technology, collected as part of the Alzheimer Disease European Sequencing consortium (ADES), comprising 15 studies from Germany, France, The Netherlands, Spain, Italy, the United Kingdom and the USA (majority of which were from the Alzheimer's Disease Sequencing Project (ADSP)[1]. All studies were approved by the ethics committees of respective institutes, and all participants provided informed consent for study participation.

Across all studies, AD cases were defined according to NIAA criteria[2] for possible or probable AD or according to NINCDS-ADRDA criteria[3] depending on the date of diagnosis. When possible, supportive evidence for an AD pathophysiological process was sought (including CSF biomarkers) or the diagnosis was confirmed by neuropathological examination. Cases were annotated with the age at onset or age at diagnosis otherwise, samples were classified as late onset AD. Controls were not diagnosed with AD.

1.1.1 ADES-FR

The ADES-FR project combines WES and WGS data from AD cases and controls from France[4]. Part of the patients are from the CNRMAJ-Rouen center (n=921) and patient ascertainment is described in detail in Nicolas et al.[5] including an update of the inclusions by the French National network CNR-MAJ (national reference center for young Alzheimer patients). Briefly, unrelated cases with early-onset AD (age at onset ≤65 years) from France were recruited among patients who fulfilled the NIAA criteria[2]. The clinical examination included personal medical and family history assessment, neurologic examination, neuropsychological assessment, and neuroimaging. In addition, cerebrospinal fluid (CSF) biomarkers indicative of AD were available for 67% of the cases. Cases with CSF biomarkers not consistent with AD diagnostics were excluded. A positive family history (i.e., at least a secondary case among first- or second-degree relatives, whatever the age of onset) was present in 45% of cases.

Patients were either screened by Sanger sequencing and QMPSF for pathogenic variants in *APP*, *PSEN1* or *PSEN2* prior to WES or by the interpretation of WES data or both. Carriers of pathogenic variants were not included for WES or were secondarily excluded following WES analysis so that none of the CNRMAJ-Rouen patients included in this work prior to shared analyses is a carrier of a pathogenic variant in *APP*, *PSEN1*, *PSEN2* as well as in a list of Mendelian dementia causative genes[6]. In addition, some controls were recruited directly from the CNRMAJ (n=30). Another large part of the samples was from the European Alzheimer's Disease Initiative (EADI) dataset[7]. This study combined clinical prevalent and incident cases of AD (n=1,121) (i) from Lille cross-sectional studies and (ii) from the Three-City (3C) study, a population-based, prospective study with 12-years of follow-up[8]. Diagnoses were established according to the DSM-III-R and NINCDS-ADRDA criteria[3]. Controls were selected among the 3C individuals not diagnosed with dementia after a 12-year follow-up (n=670). In addition, other controls were obtained from the FREX consortium[9]. These controls (n=576) were specifically designed from 6 French cities with the aim of studying and establishing the French population genetic structure of rare variants. Overall, the ADES-FR samples includes 2,042 AD cases (1,088 EOAD and 954 LOAD) and 1,276 controls. All patients and controls provided informed written consent for genetic analyses in a clinical and/or in a research setting, according to each study. In addition, the ethics committee of the Rouen University Hospital approved the use of retrospective data in the context of the ADES-FR project and with other ADES European and American partners (CERNI notifications 2017-015 and 2019-055).

Furthermore, entire exomes of 529 independent and unrelated AD patients, including 384 patients from the ECASCAD study were included. All had CSF biomarkers consistent with AD (except two patients who had neuropathological confirmation), and 90% of them were EOAD cases, the remaining 10% cases had an age of onset between 65 and 75 years. As controls, we used the extracted BAM files of the *SORL1* gene among the genome sequencing data from the FranceGenRef study. Individuals included in this study were selected based on the places of birth of their grandparents within France and at a maximum distance of 30 kilometers. A total of 862 individuals (274 females and 588 males) were sampled from three different studies: 50 individuals (25 females and 25 males) were blood donors sampled in the Finistère district, 354 individuals (177 females and 177 males) were blood donors from the PREGO biobank with ancestries in the other districts of Brittany (Côtes d'Armor, Ile-et-Vilaine, Morbihan) and in the

5 districts of the Pays-de-la-Loire region (Loire-Atlantique, Maine-et-Loire, Mayenne, Sarthe, Vendée), 458 individuals (72 females and 386 males) were volunteers from the GAZEL cohort (www.gazel.inserm.fr/en) who were selected among the volunteers who gave a blood sample and who answered a questionnaire on their parents and grandparents' places of birth. All individuals signed informed consent for genetic studies at the time they were enrolled and had their blood collected.

1.1.2 AgeCoDe-UKBonn

The AgeCoDe-UKBonn sample was derived from the following two sources, the German study on Aging, Cognition, and Dementia in primary care patients (AgeCoDe, n=294) and the interdisciplinary Memory Clinic at the University Hospital of Bonn (UKBonn, n=100).

—**The German study on Aging, Cognition, and Dementia**: The AgeCoDe study is a multicenter prospective general practice-based cohort study since 2001, including community dwelling elderly aged 75 years or older that were recruited at six study sites (Bonn, Düsseldorf, Hamburg, Leipzig, Mannheim, and Munich). The AgeCoDe study was approved by the local ethics committees of the Universities of Bonn, Hamburg, Düsseldorf, Heidelberg/Mannheim, Leipzig, and Munich. Before participation written informed consents were collected from all subjects. The AgeCoDe study aims to identify risk factors and predictors of cognitive decline and dementia[10,11]. Participants were recruited from general practitioner (GP) registries. Inclusion criteria were an age of 75 and older, absence of dementia, one or more visits to the GP in the past year, no hearing or vision impairments and German as a native language. Exclusion criteria were only home-based GP consultations, severe illness with a fatal outcome within 3 months and a language barrier. The baseline assessment including 3,327 subjects was completed between 2002 and 2003. After the baseline assessment 70 subjects were excluded due to presence of dementia after standard assessment and 40 subjects were excluded with an age below 75 years. Participants were interviewed for follow up every 18 months. All assessments are performed at the participant's home by a trained study psychologist or physician. At all visits, assessment includes the Structured Interview for Diagnosis of Dementia of Alzheimer type, Multi-infarct Dementia, and Dementia of other etiology according to DSM-IV and ICD-10 (SIDAM)[12]. The SIDAM comprises: (1) a 55-item neuropsychological test battery, including all 30 items of the MMSE and assessment of several

cognitive domains (orientation, verbal and visual memory, intellectual abilities, verbal abilities/ calculation, visual–spatial constructional abilities, aphasia/ apraxia); (2) a 14-item scale for the assessment of the activities of daily living (SIDAM-ADL-Scale); and (3) the Hachinski Rosen-Scale. Dementia was diagnosed according to DSM-IV criteria. AgeCoDe provided DNA from 294 persons who progressed to late onset AD dementia at any follow up.

—**UKBonn**: The interdisciplinary Memory Clinic of the Department of Psychiatry and Department of Neurology at the University Hospital in Bonn provided early-onset AD patients (n=100). Diagnoses were assigned according the NINCDS/ADRDA criteria[3] and on the basis of clinical history, physical examination, neuropsychological testing (using the CERAD neuropsychological battery, including the MMSE), laboratory assessments, and brain imaging.

1.1.3 Barcelona- SPIN

Neuropathological samples were obtained from the Neurological Tissue Bank of the Biobanc-HospitalClinic-IDIBAPS, and disease evaluation was performed according to international consensus criteria. Clinical samples were recruited from the multimodal Sant Pau Initiative on Neurodegeneration (SPIN) cohort (https://santpaumemoryunit.com/our-research/spin-cohort/)[13], and were evaluated at the Memory Unit at Hospital de Sant Pau (Barcelona). The repository includes clinical data of more than 6,000 participants, >2900 plasma samples, genetic material (DNA and RNA) of >3,200 and >400 subjects, respectively, and >2,000 CSF samples. All controls had normal cognitive scores in the formal neuropsychological evaluation and normal core CSF AD biomarkers, based on previously published cut-offs[14]. AD patients fulfilled clinical criteria of “probable AD dementia with evidence of the AD pathophysiological process”[3] and therefore had abnormal core AD biomarkers (low Aβ1–42 and high t-Tau or p-Tau) in the CSF. The original protocol and the subsequent amendments were approved by our local Ethics Committee at the Sant Pau Research Institute as well as the Committee of the Neurological Tissue Bank. The SPIN cohort is based on blinded enrollment and only clinically relevant biomarker results are disclosed.

1.1.4 AC-EMC

The Alzheimer Center Erasmus MC cohort (AC-EMC) includes patient referred to the Department of Neurology of the Erasmus Medical Center (Rotterdam, the Netherlands). DNA

samples from 125 patients with probable AD were included in the current study. The average age at onset was 60 years (range 41-77). A large fraction of the patients had a positive family history, defined as at least one first degree relative with dementia. All patients underwent clinical examination, neuropsychological assessment, neuroimaging, and if indicated, a lumbar puncture. The diagnosis was established according to the National Institute of Neurological and Communicative Disorders and Stroke-Alzheimer's Disease and Related Disorders Association (NINCDS-ADRDA) criteria for AD[3].The study was approved by the Medical Ethical Committee of the Erasmus Medical Center, and written informed consent was obtained from all participants or their legal representatives.

1.1.5 ERF

The Erasmus Rucphen Family (ERF) Study is a family-based cohort study that is embedded in the Genetic Research in Isolated Populations (GRIP) program in the South West of the Netherlands. The aim of this program was to identify genetic risk factors in the development of complex disorders. For the ERF study, 22 families that had at least five children baptized in the community church between 1850-1900 were identified with the help of genealogical records. All living descendants of these couples and their spouses were invited to take part in the study. Data collection started in June 2002 and was finished in February 2005.

1.1.6 Rotterdam Study

The Rotterdam Study[15] is an ongoing prospective population-based cohort study, focused on chronic disabling conditions of the elderly[16] of which a random subset was exome sequenced. Participants were screened for dementia at baseline and at follow-up examinations using the Mini-Mental State Examination (MMSE) and the Geriatric Mental Schedule (GMS) organic level[17]. Screen-positives (MMSE <26 or GMS organic level >0) underwent extensive examination[18]. Finally, individuals were diagnosed in accordance with standard criteria for dementia (Diagnostic and Statistical Manual of Mental Disorders, Third Edition, Revised (DSM-III-R)) and Alzheimer's disease, NINCDS-ADRDA[3]. Follow-up for incident dementia was complete until January 1st, 2014. The Rotterdam Study has been approved by the Medical Ethics Committee of the Erasmus MC and by the Ministry of Health, Welfare and Sport of the Netherlands, implementing the Wet Bevolkingsonderzoek: ERGO (Population Studies Act:

Rotterdam Study). All participants provided written informed consent to participate in the study and to obtain information from their treating physicians.

1.1.7 ADC-Amsterdam

The ADC-Amsterdam cohort includes patients who visit the memory clinic of the Alzheimer Center at the Amsterdam University Medical Center, The Netherlands, and was described previously[19]. DNA samples from 854 patients with probable and possible AD were included in the current study. Additionally, 353 individuals diagnosed with psychiatric and subjective cognitive complaints were included as controls. Individuals in this cohort were extensively characterized to reduce the chance of misdiagnosis. Patients underwent an extensive standardized dementia assessment, including medical history, informant-based history, a physical examination, routine blood and CSF laboratory tests, neuropsychological testing, electroencephalogram (EEG) and MRI of the brain. The diagnosis of probable AD was based on the clinical criteria formulated by the National Institute of Neurological and Communicative Disorders and Stroke—Alzheimer's Disease and Related Disorders Association (NINCDS-ADRDA) and based on National Institute of Aging–Alzheimer association (NIA-AA)[2]. Clinical diagnosis is made in consensus-based, multidisciplinary meetings. All patients gave informed consent for biobanking and for the use of their clinical data for research purposes. Selection for whole exome sequencing was based on an early age-of-onset (age at diagnosis <70 years) and available CSF biomarkers.

1.1.8 Netherlands Brain Bank

From the Netherlands Brain Bank[20] we selected brain tissues donated by patients diagnosed with Alzheimer Disease. DNA was isolated and used for WES sequencing.

1.1.9 Amsterdam-UMC

This cohort consists of WES data that were generated as part of a diagnostic work-up. All samples are from healthy adults for whom WES analysis was performed to aid the analysis of a patient, in most cases these were healthy parents of an affected child for whom trio-WES

analysis was performed. These parents either have no pathogenic variant, or are carrier of one recessive pathogenic variant that does not affect health.

1.1.10 100-plus Study

The 100-plus Study, is a prospective cohort study of cognitively healthy centenarians that associated with the Alzheimer Center at the Amsterdam University Medical Center. Detailed participant recruitment and procedures were described previously[21]. Trained researchers visited the centenarians at their home residence annually, where they were subjected to questionnaires regarding demographics, lifestyle, medical history, physical well-being and objective measurements of cognitive and physical functions. Cognitive function is tested by an extensive neuropsychological testing battery. For the current study, DNA samples 375 centenarians were included who completed at least one neuropsychological test at baseline, and exome sequencing from 349 centenarians passed QC (removal was mostly due to kinship). Centenarians who scored >22 on the MMSE were regarded as controls, while centenarians who scored ≤22 were regarded as cases[22]. The Medical Ethics Committee of the Amsterdam UMC approved this study and informed consent was obtained from all participants. The study has been conducted in accordance with the declaration of Helsinki.

1.1.11 EMIF-AD 90-plus Study

The EMIF-AD 90+ study[23] is a cohort-study of the oldest-old (90+), situated at the Amsterdam UMC and the University of Manchester. The study contributed n=72 controls. Controls were tested to have a Mini-Mental State Examination (MMSE) >=26 and a global Clinical Dementia Rating (CDR) score of 0 at baseline.

1.1.12 CBC: Control Brain Consortium

The Control Brain Consortium was previously described[24]. It consists of whole-exome sequencing in 478 samples derived from several brain banks in the United Kingdom and the United States of America. Samples were included when subjects were, at death, over 60 years of age, had no signs of neurological disease and were subjected to a neuropathological

examination, which revealed no evidence of neurodegeneration. The data was made publicly available at www.alzforum.org/exomes/hex.

1.1.13 PERADES

The PERADES sample (Defining Genetic, Polygenic and Environmental Risk for Alzheimer's Disease) comprises individuals with Alzheimer's disease (AD) and healthy controls recruited across UK, Italy and Spain. The majority of the individuals are from the UK (n=4095 with samples recruited in Cardiff: n=2405), while the rest (n=841) were recruited in Spain and Italy. More specifically the recruitment centres were: MRC Centre for Neuropsychiatric Genetics and Genomics, Cardiff University, Cardiff, UK; Institute of Psychiatry, London, UK; University of Cambridge, Cambridge, UK; University of Southampton, Southampton, UK; University of Nottingham, Nottingham, UK; Catholic University of Rome, Rome, Italy; Santa Lucia Foundation, Rome, Italy; Instituto di Neurologia Policlinico Universitario, Rome, Italy; University of Milan, Milan, Italy; Laboratory of Gene Therapy, San Giovanni Rotondo, Italy; University of Perugia, Perugia, Italy; University of Cantabria and IDIVAL, Santander, Spain and the Regional Neurogenetic Centre (CRN), ASP Catanzaro, Lamezia Terme, Italy. The collection of the samples within the MRC Centre for Neuropsychiatric Genetics and Genomics, Cardiff University was through national recruitment through multiple channels, including specialist NHS services and clinics, research registers and Join Dementia Research (JDR) platform. The participants were assessed at home or in research clinics along with an informant, usually a spouse, family member or close friend, who provided information about and on behalf of the individual with dementia. Established measures were used to ascertain the disease severity: Bristol activities of daily living (BADL), Clinical Dementia Rating scale (CDR), Neuropsychiatric Inventory (NPI) and Global Deterioration Scale (GIDS). Individuals with dementia completed the Addenbrooke's Cognitive Examination (ACE-r), Geriatric Depression Scale (GeDS) and National Adult Reading Test (NART) too. Control participants were recruited from GP surgeries and by means of self-referral (including existing studies and Joint Dementia Research platform). For all other recruitment, all AD cases met criteria for either probable (NINCDS-ADRDA, DSM-IV) or definite (CERAD) AD. All elderly controls were screened for dementia using the Mini Mental State Examination (MMSE) or ADAS-cog, were determined to be free from dementia at neuropathological examination or had a Braak score of 2.5 or lower. Control

samples were chosen to match case samples for age, gender, ethnicity and country of origin. Informed consent was obtained for all study participants, and the relevant independent ethical committees approved study protocols. The whole exome sequencing (WES) was performed in-house at the MRC Centre for Neuropsychiatric Genetics and Genomics, Cardiff University. With the Nextera technology (Nextera Rapid Capture Exome v1.2), DNA was simultaneously fragmented and tagged with sequencing adapters in a single step. The enriched libraries were sequenced using the Illumina HiSeq 4000 (Illumina, USA) as paired-end 75 base reads according to manufacturer's protocols.

1.1.14 StEP-AD

The overall goals of the Stanford Extreme Phenotypes in AD (StEP AD) project are to identify and characterize novel genetic variants that promote resilience to AD pathology in the presence of the APOE4 allele or that drive pathogenesis in the absence of the APOE4 allele. Genomes are collected from several sources, some intramural and some extramural. Invariably, the cognitive assessment protocols for these different sources vary somewhat but all include APOE genotyping, extensive neuropsychological testing, collection of one or more AD biomarkers, and consensus adjudication. Genomes were sequenced for subjects in the following three categories: (1) Protected APOE4 carriers that have the APOE3/4 genotype, are at least 80 years old, and have normal cognition. If additional follow-up is expected we will accept subjects as young as 77; (2) Super-protected APOE4 carriers that have the APOE4/4 genotype, are at least 70 years old, and have normal cognition (if additional follow-up is expected subjects as young as 67 will be accepted); (3) APOE4-negative, early-onset cases that have the APOE2/2, 2/3, or 3/3 genotype and are diagnosed with probable AD before age 65. Most are also negative for known PSEN1, PSEN2 or APP mutations.

1.1.15 Knight-ADRC

The samples Samples from the Charles F. and Joanne Knight Alzheimer's Disease Research Center (Knight ADRC) were recruited at Washington University School of Medicine (WUSM) in Saint Louis, MO (USA). (REF). All the cases received a diagnosis of dementia of the Alzheimer's type (DAT), using criteria equivalent to the National Institute of Neurological and Communication Disorders and Stroke-Alzheimer's Disease and Related Disorders Association

for probable AD[3,25]. Cognitively normal participants received the same assessment as the cases, and were deemed nondemented. Prior written consent, participants are genotyped for APOE4 allele and screened for known mutation in APP, PSEN1, PSEN2, MAPT, GRN, or C9orf72 by the Clinical and Genetics Core of the Knight ADRC. The approval number for the Knight ADRC Genetics Core family studies is 201104178.

1.1.16 UCSF/NYGC/UAB

Studies in the UCSF/NYGC/UAB dataset were described previously[26]. Cases were selected from the University of California, San Francisco (UCSF) Memory and Aging Center with an intentional selection of early-onset cases to maximize the likelihood of identifying genetic contributors, along with healthy older adult controls (a total of 664 cases and 102 controls). All UCSF cases and controls were clinically assessed during an in-person visit to the UCSF Memory and Aging Center (MAC) that included a neurological exam, cognitive assessment, and medical history. Each participant's study partner (i.e., spouse or close friend) was also interviewed regarding functional abilities. A multidisciplinary team composed of a neurologist, neuropsychologist, and nurse then established clinical diagnoses for cases according to consensus criteria. This cohort was intentionally depleted of cases with known Mendelian variants associated with neurodegenerative diseases. A small number of samples (19 cases and 21 controls) were obtained from the University of Alabama at Birmingham (UAB) from an expert clinician who employed the same diagnostic procedures.

1.1.17 UCL-DRC EOAD

University College London Dementia Research Centre (UCL-DRC) early-onset Alzheimer's disease cohort included patients seen at the Cognitive Disorders Clinics at The National Hospital for Neurology and Neurosurgery (Queen Square), or affiliated hospitals. Individuals were assessed clinically and diagnosed as having probable Alzheimer's disease based on contemporary clinical criteria in use at the time, including imaging and neuropsychological testing where appropriate. All individuals consented for genetic testing and had causative mutations for Alzheimer's disease (*PSEN1, PSEN2, APP*) and prion disease (*PRNP*) excluded prior to entry into this study.

1.1.18 ADSP

ADSP: Cases and controls were selected from over 30,000 non-Hispanic Caucasian subjects from multiple cohorts described in detail elsewhere[27]. All controls were greater than 60 years and were cognitively normal based on direct assessment. All cases met NINCDS-ADRDA criteria for possible, probably, or definite Alzheimer's disease. All cases had a documented age-at-onset, and for those with pathologically conformed AD, an age-at death. APOE genotypes were available for all. Cases were selected to have a minimal AD risk based on sex, age and APOE genotype. Controls were selected as those with the least probability of converting to AD by age 85. Controls were older (86.1 years, SD = 5.2) than cases (76.0 years, SD = 9.2). The selection criteria and the rationale for study design are described elsewhere[28]. Eventually, 5,096 cases and 4,965 controls were selected for exome sequencing by this protocol, as well as 682 additional cases from multiplex families with a strong AD family history.

ADSP extension and Augmentation phase: Under funding provided by NHGRI, an additional 3,000 subjects were whole genome sequenced. This included 1,466 cases and 1,534 controls. Of these 1,000 each of Non-Hispanic White (NHW), Caribbean Hispanic (CH), and African American (AA) descent were sequenced. Of these a total of 739 autopsy samples were sequenced [568 cases (500 NHW cases and 68 AA cases) and 171 controls (164 NHW and 7 AA)]. The Case-Control and Enriched Case Study spans 24 cohorts provided by the Alzheimer's Disease Genetics Consortium (ADGC) and the Cohorts for Heart and Aging Research in Genomic Epidemiology (CHARGE) Consortium. The Augmentation Phase encompasses sequencing done under private and NIH funding by investigators who are not members of the ADSP. The investigators for these studies have agreed to share their GWAS, WGS and WES data with the ADSP. Private funding has been provided by industry and anonymous donors. Under the NIA AD Genetics Sharing Policy and the NIAGADS Data Distribution Agreement, individual NIA funded investigators studying the genetics and the genomics of AD provide their data to NIAGADS.

Alzheimer's Disease Neuroimaging Initiative (ADNI): A public-private partnership, the purpose of ADNI is to develop a multisite, longitudinal, prospective, naturalistic study of normal cognitive aging, mild cognitive impairment (MCI), and early Alzheimer's disease as a public domain research resource to facilitate the scientific evaluation of neuroimaging and other biomarkers for the onset and progression of MCI and Alzheimer's disease. In 2017, ADNI

geneticists began collaborations with the ADSP. Whole genome sequence data on 809 ADNI subjects (cases, mild cognitive impairment, and controls) have been harmonized using the ADSP pipeline. Data used in the preparation of this article were obtained from the Alzheimer's Disease Neuroimaging Initiative (ADNI) database (adni.loni.usc.edu). The ADNI was launched in 2003 as a public-private partnership, led by Principal Investigator Michael W. Weiner, MD. The primary goal of ADNI has been to test whether serial magnetic resonance imaging (MRI), positron emission tomography (PET), other biological markers, and clinical and neuropsychological assessment can be combined to measure the progression of mild cognitive impairment (MCI) and early Alzheimer's disease (AD). For up-to-date information, see www.adni-info.org.

1.1.19 Variant calling

All contributing datasets were sequenced using a paired-end Illumina platform, but different exome capture kits were used, and a subset of the sample was sequenced using whole genome sequencing. For detailed information on variant and sample quality control and calling methods, please see Holstege and Hulsman et al., 2022[1]

1.2 Effects of non-HPV and non-PTV rare *SORL1* variants

1.2.1 Effects of rare *SORL1* variants with moderate priority: MPVs

MPVs have a relevant position in a functional domain as indicated by DMDM, but for which associated damagingness is currently unclear and requires more evidence (accompanying manuscript[29]). We identified 65 unique MPVs, carried by 80 AD cases and 61 controls. In aggregate, the risk of carrying an MPV associates with a 1.5-fold increased risk of AD (95%CI 1.1 - 2.1), and concentrates on the LOAD stage (OR=1.7, 95%CI 1.2 - 2.4, $p=1.2 \times 10^{-1}$).

Compared to *SORL1* WT patients, carriers of a MPV have an expedited AAO at later ages (**Fig 4A, Table 3 in manuscript**). MPVs with REVEL>50 associated with a 1.9-fold aggregated increased risk of (95%CI 1.3-3.0), p=2.2x10^{-3}, indicating that several, but not all MPVs are functionally relevant. We identified 6 MPVs that affected the hydrophobic core of the YWTD domain (res 754-1013) at domain positions 6, 8, 15 and 42, carried by 6 cases and three controls. Furthermore, 2 patients and 3 controls carried the p.N924S substitution, affecting YWTD-domain at position 4 (part of the SBiN-motif) of the 5th blade. In the CR-cluster (aa 1075-1550), we identified ten unique variants affecting the partly conserved glycine at position 38 which occurs in eight of the eleven CR-domains in SORL1, which were carried by 26 cases and 15 controls that affected in CR domains 5, 6, 9, 10, or 11, and in aggregate associated with a two-fold increased risk (OR = 2.0; 95%CI 1.06 – 3.8; p = 0.040). Of these, variant p.G1536S, was carried by 10 cases and 5 controls, contributing substantially to the aggregate effect of MPVs on AD risk. We further identified one AD patient who carried p.S1148R, which affects a domain-stabilizing serine at domain position 46, as part of an 'Asx-turn'. Other MPVs in the CR domain were the fingerprint at residue 39, which we observed in 5 AD cases and 3 controls. We identified 14 MPVs in the 3Fn domains (aa 1551-2121): which were carried by 7 cases and 10 controls. Lastly, in the tail domain (aa 2161-2214), we observed 4 variants affecting the conserved FANSHY motif, which is essential for SORL1 binding to the retromer core complex: three cases carried respectively p.A2173T, p.S2175R (11:121498424:C>A), and p.H2176R. A fourth variant, p.S2175R (11:121498424:C>G) was carried by 10 cases and 11 controls. Lastly, two AD cases carried the p.D2207G variant in the GGA-binding motif of the SORL1 tail, involved in binding the adapter protein AP1. Overall, more research is necessary to understand the effect of specific tail-motif substitutions in the FANSHY or the GGA-binding motifs on AD risk.

1.2.2 Effects of rare *SORL1* variants with low and no priority: LPVs and NPVs

We identified 72 LPVs, which were carried by 85 AD cases with median AAO of 70 years (95%CI: 67-75) and 85 non-demented controls. Carrying an LPV does not associate with an increased risk of AD (OR=1.2, 95%CI (0.9 – 1.6; p=1) (**Table 3 in manuscript**). Similarly, we identified 267 NPVs, which were carried by 300 AD cases with a median AAO of 73 years, (95%CI: 71-74) and 312 controls. Carrying an NPV also does not associate with increased risk

of AD (OR=1.1; 95%CI 0.9 - 1.3, p=1) (**Table 3**). Furthermore, a survival analysis indicated that carrying an LPV or an NPV does not lead to a significantly expedited AAO of AD relative to carriers of WT *SORL1* (**Fig 4A, Table 3 in manuscript**).

1.2.3 Association of variants with MAF>0.05% with AD

A total of 8,578 individuals in our sample (21%) carried at least one of the 52 variants with MAF>0.05%, 18 of which were common enough for imputation in the latest AD GWAS[30], allowing the identification of variant specific effects (**Table S2**). The most common coding *SORL1* variant, the p.A528T substitution (rs2298813, CADD score 25.5, REVEL score 0.112, carried by 3.6% of the individuals in the sample), associates with a 1.11-fold increased AD risk ($p=5.79x10^{-8}$). We find no evidence for an effect on AD by the p.E270K substitution, carried by 1.9% of all individuals in the sample and that also affects the VPS10p domain: (rs117260922, CADD score 31, REVEL score 0.31, GWAS OR=1.02; $p=5.89x10^{-1}$). The AAO of carriers from p.A528T and p.E270K variant carriers fully overlaps with carriers of WT *SORL1*. The p.D2065V substitution in the 3Fn domain (rs140327834, CADD score 28.4, REVEL score 0.568, carried by 0.46% of the sample), is associated with a 1.36-fold increased risk of AD in GWAS ($p=1.61x10^{-6}$), and we observed a slightly expedited AAO for carriers. Our analysis provides no evidence that any of the other non-rare variants in our sample associates with altered risk of AD (**Fig 4C, Table S2)**.

1.3 Comparison of DMDM with REVEL score and AlphaMissense.

Since the DMDM prioritization scheme could not be applied to the VPS10p domain, we relied on REVEL>0.5 to determine HPVs in the VPS10p domain. However, the AlphaMissense algorithm was released during our analyses, which led us to compare the performances between both algorithms when applied to all 1,103 carriers of the 511 unique rare coding *SORL1* (MAF <0.05%) identified in our dataset. To investigate whether prioritization of the 107 *SORL1* variants by the manual DMDM approach outperformed prioritization by the in-silico by the REVEL algorithm[31] (score-range: 0-1) and the AlphaMissense algorithm[32] (score-range: 0-1), we compared its outcome in terms of effects on AD risk. Of the 107 HPVs, the REVEL score was not available for one variant and was therefore excluded, such that analyses were performed in comparison with 172 carriers of 106 HPV variants. For both the AlphaMissense and the REVEL algorithms, the effect of selected *SORL1* variants on AD risk increased with higher thresholds; compared to non-*SORL1*-variant carriers, variants with the highest REVEL scores associated with a 3.9-fold increased AD risk (95%CI:1.9-8.0), and variants with the highest AlphaMissense scores associated with 3.4-fold increased AD risk (95%CI: 2.1-5.5). In comparison, HPVs selected with the manual DMDM associated with a 6.1-fold AD-risk (95%CI: 4.1 – 9.0, $p=5.3x10^{-24}$), indicating that the manual DMDM greatly outperformed the in silico-algorithms **(Fig S6-A)**. The number of unique rare variants that passed increasing thresholds declined similarly for each algorithm **(Fig S6-B)**. The variant prioritization scores generated by REVEL or AlphaMissense do not fully correlate (r = 0.52; 95%CI 0.48 - 0.56; $p < 2.2x10^{-16}$), as many variants with REVEL<0.75 had low AlphaMissense scores, which indicates a differential reliance on variant-features between algorithms **(Fig S6-C)**. Notably, the scores of the variants in the VPS10p domain have correlated better (r = 0.80; 95%CI 0.75 - 0.84; $p < 2.2x10^{-16}$), suggesting that these variants may have features that are weighted strongly by both algorithms. Nevertheless, several variants with REVEL scores <0.5 had AlphaMissense score >0.5, of which the majority affected the VPS10p domain. These variants were consequentially not annotated as HPVs, as we used the REVEL score >0.5 to select 27 VPS10p HPVs which, in aggregate, associated with an 8.8-fold increased risk of AD (95%CI 3.5- 22.3; $p=3.4\ x10^{-7}$). To investigate whether REVEL or AlphaMissense algorithms yielded the most damaging p.VPS10p variants, we compared the effect sizes. The 52 VPS10p variants selected with AlphaMissense score >0.5 associated with an aggregate 3.5-fold increased risk of AD (95%CI

2.2-5.7; $p=8x10^{-8}$), while the 24 variants with REVEL>0.5 associated with an aggregate 6.9-fold increased risk of AD (95%CI 2.4-20.0; $p=2.7x10^{-5}$). Furthermore, it seems that HPVs excluding those in VPS10p (which were preselected using REVEL) overlap more strongly with high REVEL scores than with high AlphaMissense scores. Together, our findings suggest that for our purpose of *SORL1* missense variant prioritization, using the REVEL algorithm might be preferred over the AlphaMissense algorithm, but that the manual DMDM outperforms both algorithms. This, in part, may explain the limited correlation between AlphaMissense and REVEL scores of the 106 HPVs: R=0.29 (95%CI 0.14 - 0.42; $p = 1.3x10^{-4}$). Finally, we performed a Receiver Operating Characteristic (ROC) Analysis indicated a marginal performance improvement for the AlphaMissense algorithm over the REVEL algorithm, using a combination of both algorithms (by testing the sum of both scores) slightly surpasses individual performance **(Fig S6-D**).

2 Supplementary Figures and Tables

2.1 Supplementary Figures

2.1.1 Fig S1. PCA population

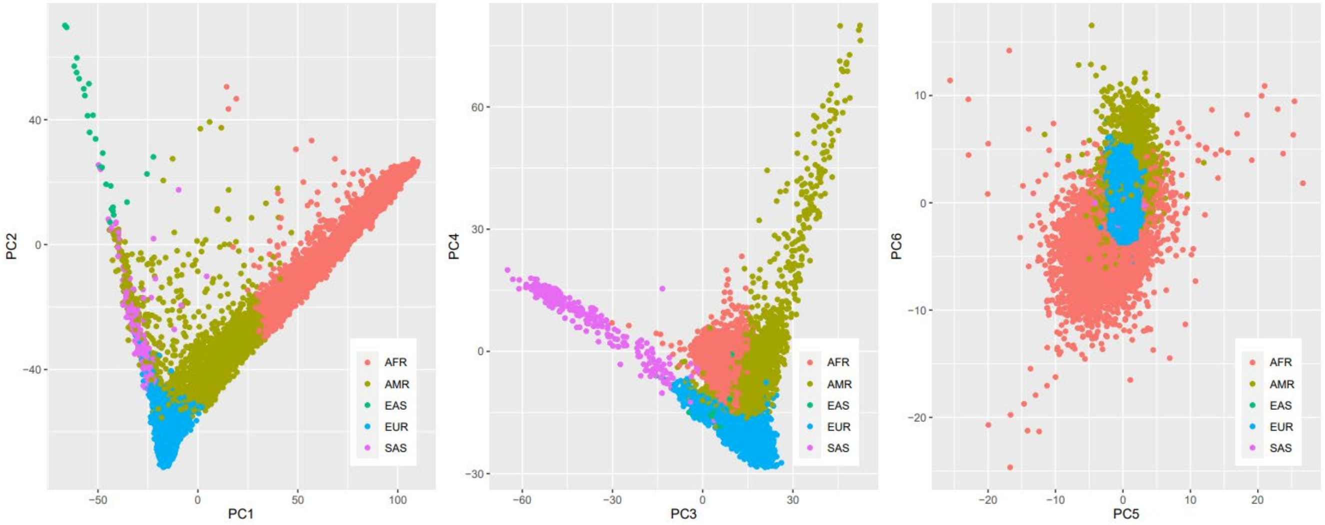

Principal Component Analysis. PCA1 vs. PCA2, PCA3 vs. PCA4, PCA5 vs. PCA6 were plotted across all samples for which whole exome sequencing was available, colored according to population group by 1000G (AFR = African, AMR = Admixed American, EAS = East Asian, EUR = European, SAS = South Asian). Note: Samples from Knight-ADRC and StEP-AD cohorts were not included here, as they were extracts of the coding sequences of the *SORL1*, *TREM2*, *ABCA7*, *ATP8B4*, *ABCA1*, *ADAM10*, *RIN3*, *CLU*, *ZCWPW1*, *ACE, and CBX3* genes as described previously[1], and based on a separate PCA on these extracts, samples were annotated as EUR as we did not identify population outliers.

2.1.2 Fig S2. Sensitivity Analysis of *SORL1* Variant Associations in European and Non-European Populations.

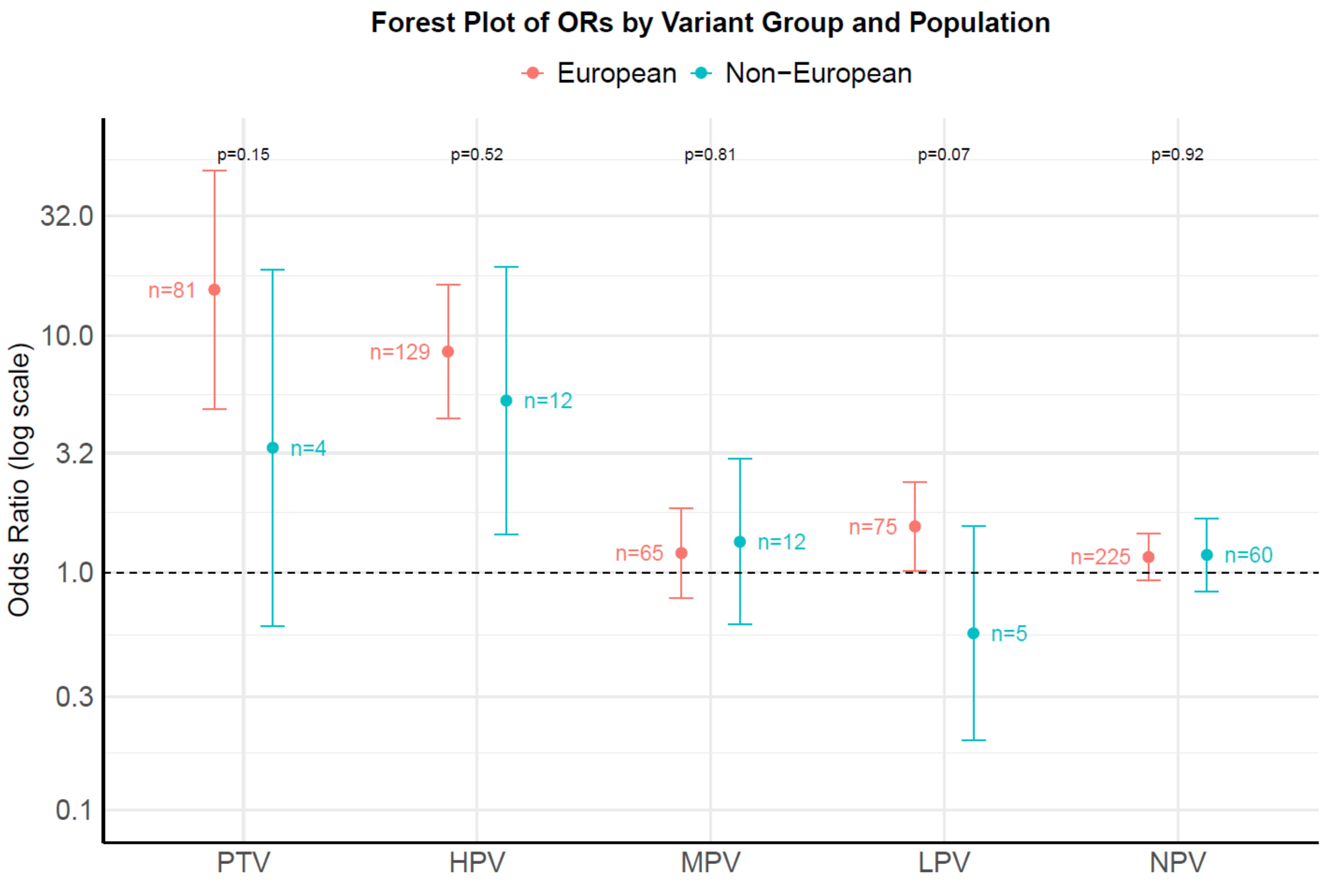

Sensitivity analysis comparing odds ratios between individuals of European and non-European ancestry, adjusted for ancestry principal components (PCs) and APOE genotype, revealed no significant differences in effect sizes across variant priority groups. Despite differences in sample sizes (European: n=15,326; non-European: n=2,682), the significant association of high-priority variants (HPVs) observed in the European sample (OR = 8.6, 95% CI: 4.5–16.5; $p_{Bonferroni} = 5.5 \times 10^{-10}$) was supported by a similar effect size in non-European individuals (OR = 5.3, 95% CI: 1.5–19.6), with a trend toward significance after Bonferroni correction ($p_{Bonferroni} = 0.06$).

2.1.3 Fig S3: The effects of MPVs, LPVs and NPVs in context of APOE.

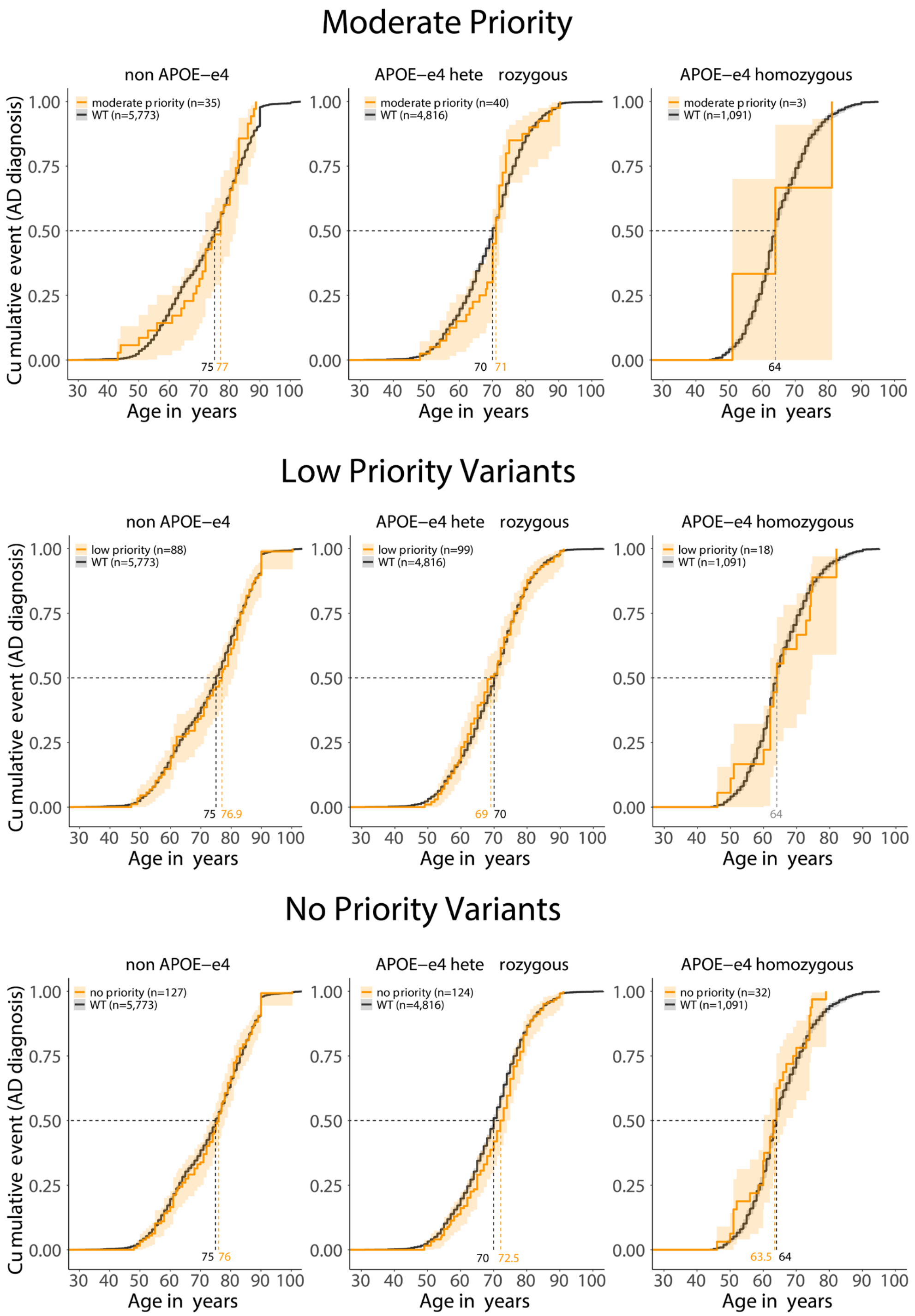

A. Age at onset of Moderate-priority variants relative to *SORL1* WT carriers. The orange ages indicate at what age respectively 50% of the variant carriers had Alzheimer's Disease, the black ages indicate at the age at which 50% of the *SORL1* WT carriers developed AD. B. Low-priority variants in context of APOE genotype. C. No-priority variants in context of APOE genotype.

2.1.4 Fig S4: Age at onset for specific variant carriers per priority group in context of APOE-genotype.

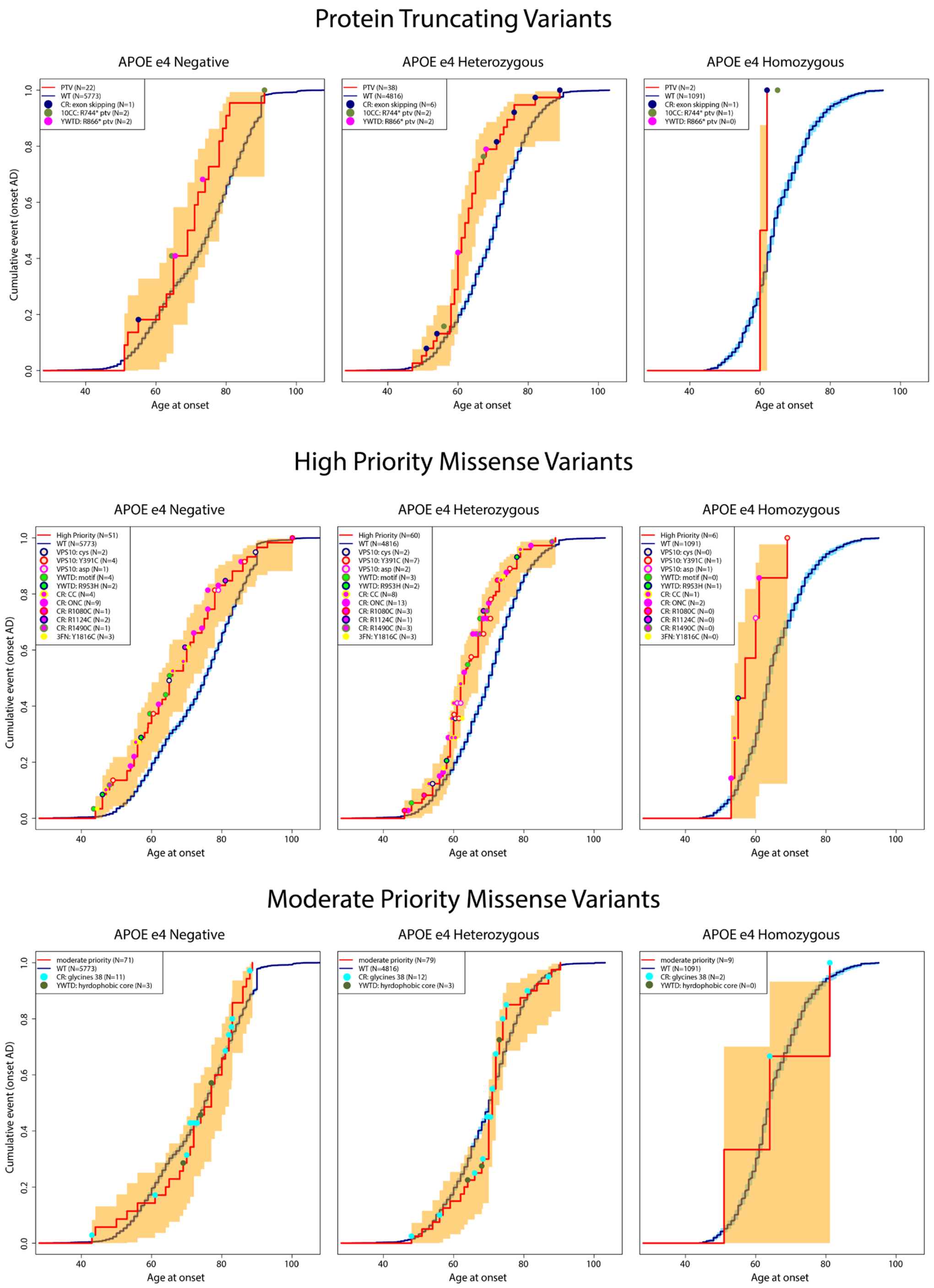

Age at onset of individual carriers with a specific variant (colored dot), in context of their *APOE*-genotype and priority group.

2.1.5 Figure S5. Age at onset annotated by APOE genotype.

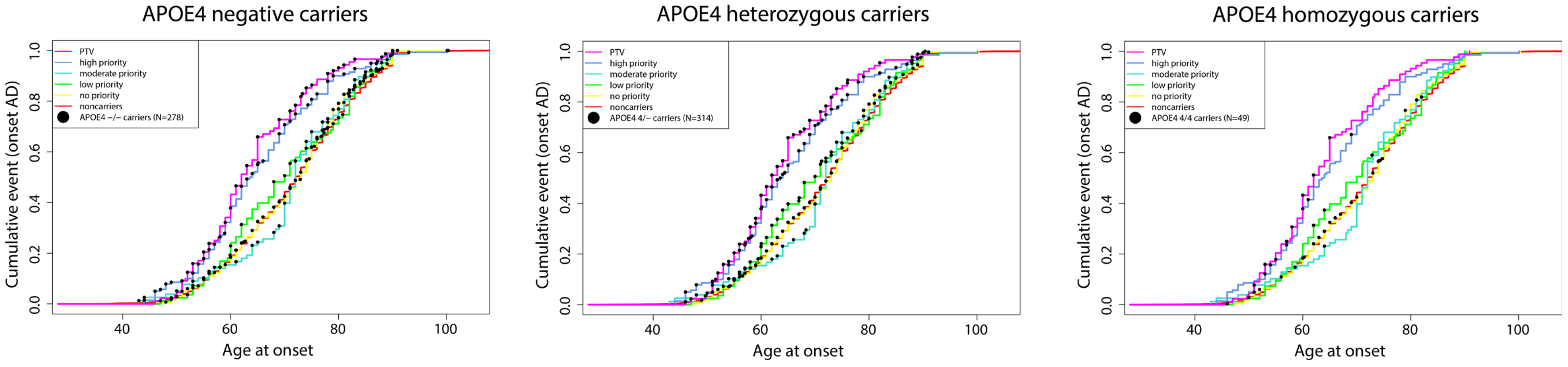

The age at onset of individual carriers with a specific APOE genotype (black dot), on prioritized categories.

2.1.6 Figure S6: Comparison of DMDM with REVEL score and AlphaMissense

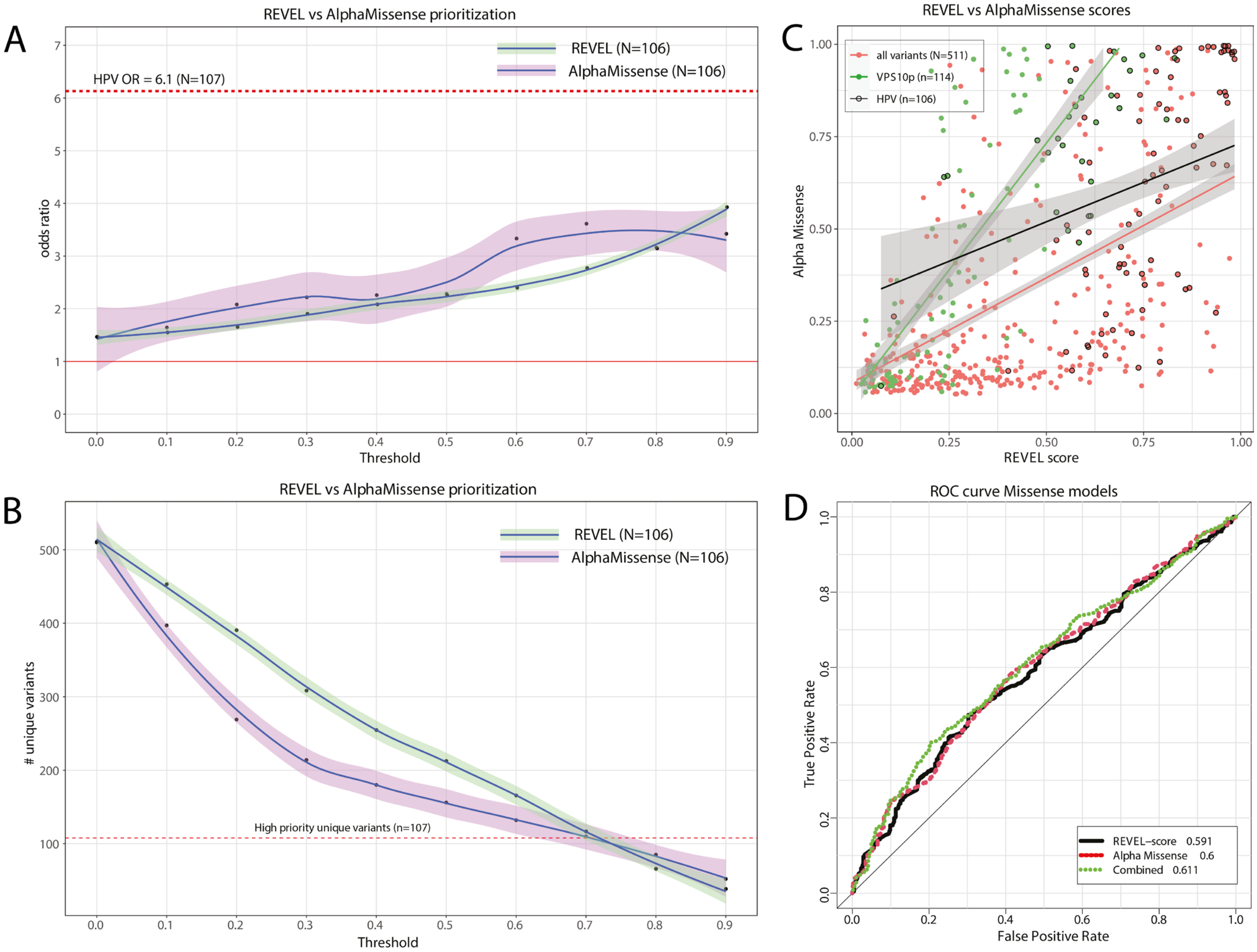

Analyses were performed with 172 carriers of 106 HPV variants, as the REVEL score was unavailable for one of the 107 HPVs. **A)** The increased risk of AD (odds ratio, y-axis), for carriers of variants with a REVEL or AlphaMissense threshold higher than a given threshold (x-axis). For comparison, the red dotted line indicates the OR in the HPV group. **B)** The number of unique variants (y-axis) with a REVEL or AlphaMissense score above a given threshold (x-axis); the red dotted line demonstrates the number of unique variants found in the high-priority category. **C)** Dotplot comparing REVEL scores with AlphaMissense scores for each variant considered in the study, annotated by variants occurring in the VPS10p domain, and variants considered HPVs. **D)** Receiver Operating Characteristic (ROC) Analysis in which the true positive and false positive rate for every unique threshold is calculated for all 106 variants for which REVEL score AND AlphaMissense scores were known. AD patients who carry a variant with an AlphaMissense score or REVEL score >X is regarded a ‘true positive’ while controls who carry a *SORL1* variant with an AlphaMissense score or REVEL score >X is regarded a ‘false positive’.

2.2 Supplemental Tables:

The list of variants considered in the burden-analysis are available for download as spreadsheets in the Supplementary Data file.

2.2.1 Table S1. XLS FILE: Rare variants (MAF < 0.05%)

2.2.2 Table S2. XLS FILE: Non-rare variants (MAF > 0.05%)

2.2.3 Table S3. XLS FILE: All excluded variants

2.2.4 Table S4: List of high-priority variants based on DMDM analysis and sequence conservation.

SORL1 motifs	domain pos	Tolerated Substitutions	SORL1 residues (aa number per subdomain)
**VPS10p**			
L2 Cys lost	Conserved disulfides	none	C^{467}, C^{473}
L1 & L2 Cys gained	Disturb disulfide	NA	L1: 391 – 411; L2: 457 – 493; L1 & L2 protrusions
Asp-box specific variants in domain positions: with REVEL>0.5	42, 44, 46, 48,49	NA	(β1) S^{138}, D^{140}, G^{142}, S^{144}, F^{145}, (β2) T^{190}, D^{192}, NA, T^{196}, NA (β3) S^{234}, D^{236}, G^{238}, T^{240}, W^{241}, (β4) S^{280}, D^{282}, NA, S^{286}, NA, (β5) S^{329}, NA, NA, NA, NA, (β6) S^{375}, NA, G^{379}, NA, F^{382}, (β7) T^{443}, D^{445}, G^{447}, T^{449}, W^{450}, (β8) S^{523}, NA, G^{527}, NA, W^{530}, (β9) S^{564}, N^{566}, G^{568}, T^{570}, W^{571}<br>Positions are likely most pathogenic when affecting a 'conserved' residue
Variants with REVEL>0.5	Random	NA	Positions are likely most pathogenic when affecting a 'conserved' residue
**10CC**			
ONC (Cys loss)	Conserved disulfides	none	(CCa) C^{625}, C^{643}, C^{660}, C^{675}, (CCb) C^{677}, C^{684}, C^{699}, C^{716}, C^{736}, C^{752}
ONC (Cys gained)	Disturb disulfide	NA	Random
**YWTD**			
YWTD-motif	16, 17, 18, 19	none	(β2) Y^{803}, W^{804}, S^{805}, D^{806}; (β3) Y^{847}, W^{848}, NA, D^{850}; (β4) F^{891}, W^{892}, T^{893}, D^{894}; (β5) Y^{934}, W^{935}, T^{936}, D^{937}; (β6) Y^{974}, W^{975}, NA, D^{977}
Partly conserved Pro	3	none	(β4) P^{878}, (β5) P^{923}, (β6) P^{963}
Partly conserved Asp	9	none	(β2) D^{796}, (β5) D^{929}
Highly conserved Ile	27	none	(β1) I^{769}, (β2) I^{812}, (β3) I^{856}, (β4) I^{902}, (β5) I^{943}, (β6) I^{983}
Highly conserved Arg	29	none	(β1) R^{771}, (β2) R^{814}, (β3) NA, (β4) R^{904}, (β5) R^{945}, (β6) R^{985}
Highly conserved G	35	none	(β1) G^{777}, (β2) G^{819}, (β3) G^{863}, (β4) G^{909}, (β5) G^{950}, (β6) G^{991}
Partly conserved R*	38	none	(β3) R^{866}, (β5) R^{953}; DMDM only when R changed
Cysteines Disulfide β2	Likely disulfide	none	(β2) C^{801}, (β2) C^{816}
**EGF**			
ONC (Cys loss)	Conserved disulfides	none	C^{1021}, C^{1026}, C^{1030}, C^{1040}, C^{1042}, C^{1058}, C^{1060}, C^{1071}
ONC (Cys gain)	Disturb disulfides	NA	random
**CR**			
Calcium cage (D,D,D,E)	37, 41, 47, 48	none	(CR1) D^{1098}, D^{1102}, D^{1108}, E^{1109}; (CR2) D^{1139}, D^{1143}, D^{1149}, E^{1150}; (CR3) D^{1178}, D^{1182}, D^{1188}, E^{1189}; (CR4) D^{1219}, D^{1223}, D^{1229}, E^{1230}; (CR5) D^{1257}, D^{1261}, D^{1267}, E^{1268}; (CR6) D^{1297}, Q^{1301}, D^{1307}, E^{1308}; (CR7) D^{1345}, D^{1349}, D^{1355}, E^{1356}; (CR8) D^{1389}, D^{1393}, D^{1399}, E^{1400}; (CR9) D^{1439}, D^{1443}, D^{1449}, E^{1450}; (CR10) D^{1492}, D^{1496}, D^{1502}, E^{1503}; (CR11) D^{1535}, D^{1539}, D^{1545}, E^{1546}
ONC (Cys loss)	15, 23, 29, 36, 42, 55<br>Conserved disulfides	none	(CR1) C^{1078}, C^{1085}, C^{1090}, C^{1097}, C^{1103}, C^{1112}; (CR2) C^{1117}, C^{1125}, C^{1131}, C^{1138}, C^{1144}, C^{1153}; (CR3) C^{1158}, C^{1165}, C^{1170}, C^{1177}, C^{1183}, C^{1192}; (CR4) C^{1199}, C^{1206}, C^{1211}, C^{1218}, C^{1224}, C^{1235}; (CR5) C^{1239}, C^{1244}, C^{1249}, C^{1256}, C^{1262}, C^{1271}; (CR6) C^{1275}, C^{1283}, C^{1289}, C^{1296}, C^{1302}, C^{1315}; (CR7) C^{1325}, C^{1332}, C^{1337}, C^{1344}, C^{1350}, C^{1359}; (CR8) C^{1368}, C^{1376}, C^{1381}, C^{1388}, C^{1394}, C^{1403}; (CR9) C^{1419}, C^{1426}, C^{1431}, C^{1438}, C^{1444}, C^{1453}; (CR10) C^{1471}, C^{1478}, C^{1484}, C^{1491}, C^{1497}, C^{1506}; (CR11) C^{1514}, C^{1521}, C^{1527}, C^{1534}, C^{1540}, C^{1549}
ONC (Cys gain)	Disturb disulfides	NA	random
Asx-turn (D)	44	none	(CR1) D^{1105}, (CR2) D^{1146}, (CR3) D^{1185}, (CR4) D^{1226}, (CR5) D^{1264}, (CR6) D^{1304}, (CR7) D^{1352}, (CR8) D^{1396}, (CR9) D^{1446}, (CR10) D^{1499}, (CR11) D^{1542}
**3Fn**			
Partly conserved prolines	P's at 6, 7, 28, 79	none	(3FN1) NA, NA; P^{1578}, P^{1619}; (3FN2) P^{1654}, NA; P^{1676}, NA; (3FN3) P^{1749}, P^{1750}; NA, NA; (3FN4) P^{1843}, P^{1844}, P^{1865}, NA; (3FN5) P^{1934}, P^{1935}, P^{1955}, P^{1998}; (3FN6) NA, P^{2027}, NA, NA
W; B-strand	25	none	(3FN1) W^{1575}, (3FN2) W^{1673}, (3FN3) (L^{1767}), (3FN4) W^{1862}, (3FN5) W^{1952}, (3FN6) W^{2043}
Partly conserved glycines	G's at 36, 96	none	(3FN2) G^{1681}, (3FN2) G^{1732}, (3FN4) G^{1917}
Y; C-strand	41	aromatic resi-dues tolerated	(3FN1) Y^{1588}, (3FN2) Y^{1686}, (3FN3) Y^{1778}, (3FN4) Y^{1870}, (3FN5) Y^{1965}, (3FN6) Y^{2059}
L; EF-loop (tyrosine corner)	77	none	(3FN1) L^{1617}, (3FN2) L^{1713}, (3FN3) L^{1810}, (3FN4) NA, (3FN5) L^{1996}, (3FN6) L^{2087}
Y; F-strand (tyrosine-corner)	83	none	(3FN1) Y^{1623}, (3FN2) Y^{1719}, (3FN3) Y^{1816}, (3FN4) Y^{1905}, (3FN5) Y^{2002}, (3FN6) Y^{2093}
Arg hotspot	88:	none	(3FN4) R^{1910} Based on Arg substitutions in DMDM pathogenic
Cysteines 1st domain	39, 91, disulfide	none	(3FN1) C^{1586}, (3FN1) C^{1631}
Cysteines 6th domain	91, 98: disulfide	none	(3FN6) C^{2101}, (3FN6) C^{2108}

Prioritization of positions likely to harbor pathogenic mutations based on either domain sequence conservation or because the DMDM analysis. Analysis of enrichment: number of observations in sample (cases and controls) per possible positions. NA: in the specific domain, the prioritized variant does not exist. * When Arg at position 38 in the YWTD-domain of LDLR, LRP4 or LRP5 are substituted by other residues, this leads to autosomal dominant inherited forms of diseases. SORL1 only has a Arg at position 38 in β3 and β5: R^{866} and R^{953}.

2.2.5 Table S5: Moderate-priority variants

SORL1 motifs	domain pos	Tolerated Substitutions	SORL1 residue/aa no., per subdomain
**VPS10p**			
RGD (in ProP)	NA	none	R^{63}, G^{64}, D^{65}
RRKR (in ProP)	NA	none	R^{78}, R^{79}, K^{80}, R^{81}
Hydrophobic; A-strand	5, 6	Conservative (hydrophobic) substitutions likely tolerated	(β1) V^{106}, V^{107}, (β2) F^{167}, Y^{168}, (β3) L^{209}, L^{210}, (β4) NA, F^{251}, (β5) F^{300}, NA, (β6) Y^{349}, Y^{350}, (β7) F^{414}, NA, (β8) L^{495}, NA, (β9) Y^{539}, Y^{540}, (β10) V^{583}, Y^{584}
Hydrophobic; B-strand	19, 20, 21	Conservative (hydrophobic) substitutions likely tolerated	(β1) I^{117}, V^{118}, A^{119}, (β2) Y^{177}, I^{178}, F^{179}, (β3) L^{218}, L^{219}, L^{220}, (β4) I^{264}, Y^{265}, I^{266}, (β5) Y^{306}, M^{307}, F^{308}, (β6) V^{359}, F^{360}, V^{361}, (β7) Y^{424}, I^{425}, A^{426}, (β8) I^{504}, I^{505}, A^{506}, (β9) I^{548}, I^{549}, NA, (β10) V^{596}, F^{597}, NA
Hydrophobic; C-strand	39, 40, 41	Conservative (hydrophobic) substitutions likely tolerated	(β1) V^{135}, Y^{136}, V^{137}, (β2) L^{187}, W^{188}, I^{189}, (β3) L^{231}, W^{232}, NA, (β4) V^{277}, F^{278}, NA, (β5) L^{326}, W^{327}, V^{328}, (β6) L^{372}, Y^{373}, I^{374}, NA, V^{441}, I^{442}, (β7) V^{520}, Y^{521}, I^{522}, (β8) L^{561}, NA, Y^{563}, (β9) L^{613}, NA, V^{615}
**YWTD**			
hydrophobic	6, 8, 15	Conservative (hydrophobic) substitutions likely tolerated	(β2) L^{793}, F^{795}, L^{802}; (β3) L^{837}, F^{839}, L^{846}; (β4) L^{881}, L^{883}, M^{890}; (β5) I^{926}, V^{928}, I^{933}; (β6) I^{966}, V^{968}, I^{973}; (β1) M^{1007}, I^{1009}, NA
hydrophobic	41, 42	Conservative (hydrophobic) substitutions likely tolerated	(β1) NA, L^{782}; (β2) V^{825}, I^{826}; (β3) I^{869}, V^{870}; (β4) L^{915}, V^{916}; (β5) I^{956}, L^{957}; (β6) I^{996}, L^{997}
Highly conserved L	47	none	(β1) L^{787}, (β2) L^{831}, (β3) L^{875}, (β4) V^{920}, (β5) L^{960}, (β6) L^{1001}
SBIN, NXI, Ligand binding?	4, 20 blades β4, β5, β6	uncertain	(β4) R^{879}, (β4) W^{895}, (β5) N^{924}, (β6) Y^{964}, (β6) W^{978}
**CR**			
Asx-turn (S)	46	none	(CR1) S^{1107}, (CR2) S^{1148}, (CR3) S^{1187}, (CR4) S^{1228}, (CR5) S^{1266}, (CR6) S^{1306}, (CR7) S^{1354}, (CR8) S^{1398}, (CR9) S^{1448}, (CR10) NA, (CR11) S^{1544}
Fingerprints, Involved in ligand binding	34, 39	uncertain	(CR1) W^{1095}, D^{1100}; (CR2) Y^{1136}, E^{1141}; (CR3) W^{1175}, D^{1180}; (CR4) W^{1216}, D^{1221}; (CR5) K^{1254}, L^{1259}; (CR6) M^{1294}, I^{1299}; (CR7) W^{1342}, M^{1347}; (CR8) W^{1386}, E^{1391}; (CR9) W^{1436}, Y^{1441}; (CR10) K^{1489}, H^{1494}; (CR11) E^{1532}, F^{1537}
Phe-Ile hyd core, Strong conservation	21, 30	Uncertain, (few variants in DMDM analysis)	(CR1) Y^{1083}, I^{1091}; (CR2) F^{1123}, I^{1132}; (CR3) Y^{1163}, I^{1171}; (CR4) F^{1204}, I^{1212}; (CR5) F^{1242}, I^{1250}; (CR6) F^{1281}, L^{1290}; (CR7) F^{1330}, I^{1338}; (CR8) F^{1374}, I^{1382}; (CR9) Y^{1424}, V^{1432}; (CR10) F^{1476}, I^{1485}; (CR11) F^{1519}, I^{1528}
Partly conserved glycines, (Few variants in DMDM analysis)	27, 38	uncertain	(CR1) G^{1088}, NA; (CR2) G^{1129}, NA; (CR3) G^{1168}, G^{1179}; (CR4) G^{1209}, G^{1220}; (CR5) G^{1247}, G^{1258}; (CR6) NA, G^{1298}; (CR7) G^{1335}, G^{1346}; (CR8) G^{1379}, NA; (CR9) G^{1429}, G^{1440}; (CR10) NA, G^{1493}; (CR11) NA, G^{1536}
**3Fn**			
Hydrophobic	11, 13	Conservative (hydrophobic) substitutions likely tolerated	(3FN1) L^{1561}, $\underline{W^{1563}}$; (3FN2) L^{1659}, L^{1661}; (3FN3) I^{1753}, I^{1755}; (3FN4) L^{1848}, A^{1850}; (3FN5) L^{1938}, V^{1940}; (3FN6) L^{2030}, I^{2032}
Hydrophobics in B-strand	21, 23	Conservative (hydrophobic) substitutions likely tolerated	(3FN1) V^{1571}, L^{1573}; (3FN2) I^{1669}, NA; (3FN3) L^{1763}, F^{1765}; (3FN4) V^{1858}, C^{1860}; (3FN5) V^{1948}, I^{1950}; (3FN6) V^{2039}, L^{2041}
Hydrophobics in C-strand	43, 45	Conservative (hydrophobic) substitutions tolerated	(3FN1) V^{1590}, Y^{1592}; (3FN2) V^{1688}, Y^{1690}; (3FN3) V^{1780}, L^{1782}; (3FN4) I^{1872}, Y^{1874}; (3FN5) V^{1967}, V^{1969}; (3FN6) I^{2061}, M^{2063}
Partly conserved glycines	94	uncertain	(3FN2) G^{1730}, (3FN3) G^{1827}, (3FN6) G^{2104}
Hydrophobics in F-strand	85, 87, 89	Conservative (hydrophobic) substitutions tolerated	(3FN1) V^{1625}, V^{1627}, V^{1629}; (3FN2) V^{1721}, V^{1723}, A^{1725}; (3FN3) I^{1818}, A^{1820}, A^{1822}; (3FN4) F^{1907}, V^{1909}, V^{1911}; (3FN5) I^{2004}, V^{2006}, L^{2008}; (3FN6) F^{2095}, V^{2097}, A^{2099}
Hydrophobics in E-strand	72, 74	Conservative (hydrophobic) substitutions tolerated	(3FN1) NA, L^{1614}; (3FN2) NA, I^{1710}; (3FN3) NA, V^{1807}; (3FN4) V^{1897}, V^{1899}; (3FN5) Y^{1991}, L^{1993}; (3FN6) F^{2082}, I^{2084}
Trp-ladders		uncertain	(3FN1) R^{1593}, W^{1600}, K^{1626}, H^{1636}; (3FN2) E^{1690}, W^{1698}, R^{1722}, W^{1734}
**Tail**			
FANSHY		uncertain	F^{2172}, A^{2173}, N^{2174}, S^{2175}, H^{2176}, Y^{2177}
Acidic		uncertain	D^{2190}, D^{2191}, L^{2192}, G^{2193}, E^{2194}, D^{2195}, D^{2196}, E^{2197}, D^{2198}
GGA		uncertain	D^{2207}, D^{2208}, V^{2209}, P^{2210}, M^{2211}, V^{2212}, I^{2213}, A^{2214}

Rare variants (MAF<0.05%) in these positions are considered moderate-priority. Variants correspond with the grey residues in **Figure 3**. NA: the specific domain does not have the prioritized variant. Underlined: Note that we observed a moderate-priority variant leading to a W1563C substitution of a hydrophobic residue at position 13 in the 3Fn domain. This is a non-rare variant, with a non-neuro pop-max MAF of 6.7×10^{-4} (GnomAD) a REVEL score of 0.476 and a CADD score of 32. We observed no evidence for an effect on AD of this variant in the latest GWAS: OR=0.95 (95% CI 0.56-1.6], $p=8.35 \times 10^{-1}$.

2.2.6 Table S6. Associations of priority groups using logistic regression

Variant group	Variant Subcategory	Type of Analysis	carriers	Variant effect on AD risk					
		Logistic reg./ Fisher	all/EOAD/LOAD/ controls	All carriers OR	All carriers p value	EOAD OR	EOAD p value	LOAD OR	LOAD p value
**Protein Truncating Variants**									
**PTV**	Total	Logistic reg.	90/58/27/5	11.29 (4.51 - 28.21)	5.94E-06	22.37 (8.36 - 59.84)	1.62E-08	7.02 (2.66 - 18.51)	2.16E-03
		Fisher	95/59/30/6	17.2 (7.5 - 39.3)	1.20E-21	35.3 (15.2 - 81.8)	6.20E-31	8.6 (3.6 - 20.6)	3.80E-07
**Rare Missense variants**									
**HPV: high priority**	Total	Logistic reg.	154/74/67/13	8.06 (4.52 - 14.38)	4.32E-11	13.78 (7.38 - 25.75)	5.13E-15	6.02 (3.28 - 11.05)	1.82E-07
		Fisher	180/80/71/29	6.1 (4.1 - 9.0)	5.30E-24	9.9 (6.5 - 15.2)	7.80E-29	4.2 (2.7 - 6.5)	1.30E-10
**MPV: Moderate priority**	all	Logistic reg.	124/19/58/47	1.25 (0.85 - 1.83)	1	0.93 (0.51 - 1.68)	1	1.36 (0.91 - 2.03)	1
		Fisher	141/20/60/61	1.5 (1.1 - 2.1)	0.39	1.2 (0.7 - 1.9)	1	1.7 (1.2 - 2.4)	1.20E-01
**LPV: low priority**	all	Logistic reg.	127/31/49/47	1.33 (0.91 - 1.94)	1	1.67 (1.01 - 2.79)	1	1.23 (0.81 - 1.87)	1
		Fisher	170/33/52/85	1.2 (0.9 - 1.6)	1	1.4 (0.9 - 2.1)	1	1.0 (0.7 -1.5)	1
**NPV: no priority**	all	Logistic reg.	491/95/190/206	1.17 (0.97 - 1.42)	1	1.29 (0.97 - 1.72)	1	1.13 (0.92 - 1.39)	1
		Fisher	612/101/199/312	1.1 (0.9 - 1.3)	1	1.2 (0.9 - 1.4)	1	1.1 (0.9 - 1.3)	1

Comparison of *SORL1* variant subtype effects on Alzheimer's disease risk, estimated by logistic regression (adjusted for PCs 1–6 and APOE) and Fisher's exact test. P-values were corrected for multiple testing. While some effect sizes shifted slightly between logistic regression and Fisher's exact test, the overall associations remained consistent with those observed (**Table 3**) using Fisher's exact test, supporting the robustness of the findings.

OR: Odds ratio; EOAD: Early onset AD; LOAD: Late onset AD; PTV: Protein truncating variant

2.2.7 Table S7. Age at onset per variant carrier group relative to WT *SORL1* carriers

***SORL1* variant type**	**Median age at onset 95%CI on median**	**10%-90% Inter percentile range**	**Δ median AAO vs WT (95% CI; p value*)**	**Δ median AAO vs PTV (95% CI, p value*)**
**Fig 4a**				
SORL1 WT	72 (72 - 73)	56-87	NA	10 (12 — 8); 2.7E-11
PTV	62 (60 - 65)	52-78	-10 (-12 — -8); 2.7E-11	NA
High-priority missense	64 (62 - 67)	53-79	-8 (-10 — -6); 5.3E-9	2 (2 – 2); 1
Moderate-priority missense	72 (71 - 74)	54-86	0 (-1 — 1); 1	10 (11 – 9); 2.3E-3
Low-priority missense	70 (67 - 75)	56-84.9	-2 (-5 — 2); 1	8 (7 – 10); 6.9E-3
No-priority missense	73 (71 - 74)	55-86	1 (-1 - 1); 1	11 (11 – 9); 3.3E-7
**Fig 4b**				
YWTD-motif	64 (48 - NA)	44-68	-8 (-24 — NA); 2.6E-3	2 (-12 – NA); 1
calcium cage	60 (56 - NA)	54-73	-12 (-16 — NA); 7.7E-4	-2 (-4 – NA); 1
VPS10 REVEL>50	59.5 (56 - 78)	46-83	-12.5 (-16 —5); 1	-2.5 (-4 – 13); 1
ONC in CR domain	68 (63 - 74.3)	53-82	-4 (-9 — 1.3); 1	6 (3 – 9.3); 1
10CC	67 (63 - 73)	57-78	-5 (-9 — 0); 1	5 (3 – 8); 1
Y391C VPS10p Loop1	67.5 (60.1 - NA)	60-78	-4.5 (-11.9 — NA); 1	5.5 (0.1 – NA); 1
other HPV carriers	61 (60 - 70)	53-79	-11 (-12 — -3); 9.4E-3	-1 (0 – 5); 1
**Fig 4C**				
D2065V	70 (68 - 72)	53.1-85	-2 (-4 — -1); 7.1E-1	8 (8 – 7); 9.7E-4
E270K	72 (71 - 73)	56-87	0 (-1 — 0); 1	10 (11 – 8); 5.2E-9
A528T	71 (70 - 72)	55-86	-1 (-2 — -1); 1.7E-1	9 (10 – 7); 4.8E-8
other MAF >0.05%	74 (73 - 74)	57-87	2 (1 — 1); 1	12 (13 – 9); 4.9E-13

The estimated median age at onset per priority group or specific variant carrier group and the difference between the age at onset of carriers of WT *SORL1*. For all the different variant groups, both the age at onset when 50% and 80% of the variant carriers developed AD is given. *P value was calculated using a log-rank test. See **Figure 4** for age distributions per variant group. ONC: odd numbered cysteines (gain or loss of a cysteine).

2.2.8 Table S8. Differences in age at onset per priority category in context of APOE genotype.

SORL1 variant type	APOE-4 genotype	Median age at onset (95%CI)	10%-90% Inter percentile range	Δ median AAO v.s. WT (95% CI); p value*	Δ median AAO v.s. PTV (95% CI); p value*
**Fig 5**					
WT	E4-NEG	75 (75 - 76)	56-89	NA	6 (10 — 2); 2.7E-2
	E4-HET	70 (70 - 71)	55-82	NA	9 (10 — 6); 4.0E-6
	E4-HOM	64 (63 - 64)	54-77	NA	4 (6 - NA); 9.2E-2
PTV	E4-NEG	69 (65 - 74)	52-81	-6 (-10 — -2); 2.7E-2	NA
	E4-HET	61 (60 - 65)	51-74	-9 (-10 — -6); 4.0E-6	NA
	E4-HOM	60 (57 - NA)	53-65	-4 (-6 - NA); 9.2E-2	NA
HPV	E4-NEG	66 (62 - 74.3)	48-86	-9 (-13 – -1.7); 1.7E-2	-3 (-3 - 0.3); 1
	E4-HET	63 (62 - 67)	54-78	-7 (-8 —-4); 1.6E-4	2 (2 - 2); 1
	E4-HOM	58.5 (55 - NA)	53-69	-5.5 (-8 — NA); 2.9E-2	-1.5 (-2 - NA); 1
**Fig S2**					
MPV	E4-NEG	77 (72 - 82)	53-86	-2 (-3 — 6); 1	8 (7 - 8); 0.9
	E4-HET	71 (70 - 73)	56.5-82.4	1 (0 - 2); 1	10 (10 - 8); 2.7E-2
	E4-HOM	64 (51 - NA)	51-81.1	0 (-12 - NA); 1	4 (-6 - NA); 1
LPV	E4-NEG	78.2 (71 - 83)	53-88	3.2 (4 - 7); 1	9.2 (6 - 9); 0.4
	E4-HET	66 (63 - 71)	56-81	-4 (-7 - 0); 1	5 (3 - 6); 0.8
	E4-HOM	72.8 (64 - NA)	62-82	-8.8 (1 - NA); 1	12.8 (7 - NA); 0.2
NPV	E4-NEG	76 (74 - 78)	55-89	1 (1 - 2); 1	7 (9 - 4); 0.1
	E4-HET	72.5 (70 - 74)	57-82	2.5 (0 - 3); 1	11.5 (10 - 9); 1.5E-05
	E4-HOM	63.5 (60.3 - 67)	51-74	-0.5 (-2.7 - 3); 1	3.5 (3.3 - NA); 0.7

The estimated median age at onset per priority group or specific variant carrier group, in context of its APOE-E4 genotype, and the difference between the age at onset of carriers of WT *SORL1*. For all the different variant groups, both the age at onset when 50% and 80% of the variant carriers developed AD is given. *P value was calculated using a log-rank test.

3 Compendium

3.1 The VPS10p-domain (residues 1-753)

3.1.1 Sequence details

Signal peptide (residues 1-28)

The most N-terminal part encoded by the *SORL1* gene is a signal peptide (SP) (residues 1-28) that directs the polypeptide into the endoplasmatic reticulum (ER) and is lost by signal peptidase cleavage upon translocation into the ER, similar to other transmembrane proteins. Accordingly, functional SORL1 protein in cells does not contain this initial part of the polypeptide.

Propeptide (residues 29-81)

Residues 29-81 of SORL1 is a propeptide (ProP), that can be removed from the remaining part of the receptor by enzymatic cleavage by the prohormone convertase furin that is active in secretory vesicles in the late Golgi/TGN [33]. The ^{78}RRKR^{81} tetrapeptide serves as a recognition site for furin binding and cleavage between residues 81-82 [34]. The ProP also contains a ^{63}RGD^{65} tripeptide motif, which serves as an interaction site for adhesive proteins, including integrins in other proteins. This suggests that SORL1 proteins that escape cleavage by furin still include a ProP and may have a unique function in cell adhesion and integrin binding. The presence of ProP is also suggested to block binding of small ligands to the VPS10p-domain (see next section), which is speculated to prevent binding of certain ligands to the VPS10p-domain in the ER of cells where receptor and ligand are co-expressed [34].

10-bladed β-propeller (residues 82-617)

After the ProP follows the VPS10p-domain: with its 536 residues, this domain is one of the largest protein domains known. There is only modest sequence conservation between domains across the five members of the VPS10p family (SORL1, sortilin, SorCS1, SorCS2, SorCS3) [35]. The crystal structure of the VPS10p-domain of sortilin was solved in 2009 [36], and in 2015 for the SORL1 domain [37], making it the only full SORL1-domain for which the three-dimensional conformation is currently determined. Both sortilin and SORL1 VPS10p-domain structures are organized in a ten-bladed β-propeller, each blade composed of four antiparallel β-strands (A-D) arranged around a central conical tunnel. C-terminal to both VPS10p-domains is a small domain, corresponding to 136 amino acids of SORL1 including ten conserved

cysteine residues with a stringent spacing, known as the 10CC region. This domain (residues 618-753 in SORL1) interacts extensively with the β-propeller (see below) (**Supplemental Figure S7a**). The overall dimensions of the β-propeller resemble a 94 Å x 72 Å x 56 Å ellipsoid, and for SORL1 the central cavity narrows toward the bottom face with a maximum width of ~25 Å [37]. The β-propeller is further characterized by a defined and almost flat bottom and top face, demarcated by the loops between strands A-B and C-D, and strands B-C and D-A, respectively (**Supplemental Figure S7b,c**). Interestingly, the solved domain structures of both sortilin and SORL1 were determined in the presence of complexed ligands, which indicated that small (peptide) ligands can bind inside the tunnel.

Alignment of the ten sequences that define each blade, β1-β10 (residues 82-617) (**Supplemental Figure S7c**), revealed several conserved and functional motifs. The presence of two stretches of hydrophobic amino acids in the inner strands (A: positions 5, 6, and B: positions 19, 20, 21) of each blade is critical for domain stability. Similarly, 2-3 hydrophobic residues in strand C (positions 39, 40, 41) establish hydrophobic interactions among the blades that enable the formation of the large central tunnel. Additionally, they partake in the generation of a hydrophobic surface that allows the interaction of small lipophilic ligands with the propeller cavity, as demonstrated for the binding of the Amyloid-β peptide to SORL1 [37].

Asp-boxes (part of β-propeller)

The motif with consensus sequence S(or T)-X-D(or N)-X-G-X-T(or S)-W(or F/Y) (spanning positions 42-49 of each blade) is known as the Asp-box [38], which folds as a conserved β-hairpin [39]. The SORL1 VPS10p-domain contains Asp-boxes in the loops located (at the bottom of the domain) between the third (C) and fourth (D) strands of the blades (**Supplemental Figure S7b,c**). The exact function of this motif has not been clarified. However, the Asp-boxes of the SORL1 domain are unlikely to be directly involved in ligand recognition, since only the top face of propellers appears to be used for this purpose [40]. Rather, the conserved amino acids of the Asp-box motif likely stabilize the propeller, forming blade-to-blade interactions, contacts with preceding loops, or with the nearby 10CC-domains.

L1-L2 Loops (part of the β-propeller)

The two sequences connecting β6 with β7 and β7 with β8 are longer than the sequences connecting other blades. Part of these two longer protrusions (residues Y391-F411 and A457-P493, respectively) include the two "*loop structures*" termed L1 (residues Y391-A412) and L2 (residues L481-P493), respectively [37] (**Supplemental Figure S7c**; loop residues underligned). L2 is located close to the upper entrance of the tunnel, and seems to push bound ligand against the tunnel wall at neutral pH. The L1 segment occupies a more central position, blocking part of the pore. Interestingly, L1 is not present in VPS10p-domains in other proteins, which suggests that the VPS10p-domain in SORL1 has a unique ligand binding profile.

Indeed, both L1 and L2 appear to be essential for peptide binding since removal of either of these two protrusions completely abolishes binding activity [37]. Both loops are flexible and undergo conformational changes at different pH, assuming a more stable conformation at pH 6.5 than at pH 4.5. The disordering of the loops at low pH was suggested as important for ligand release at acidic pH, providing a molecular mechanism for dissociating ligands from this SORL1 domain in lysosomes, when SORL1 encounters the low pH in this organelle.

10CC region (residues 618-753)

The VPS10p β-propeller fold is stabilized by the neighboring 10CC region, which is split into two shorter domains named 10CCa (residues 618-675) and 10CCb (residues 676-753). While rather similar to each other, these domains show neither sequence nor structural resemblance to other known domains. The ten conserved cysteines form five intrachain disulfide bridges with connectivity between cysteines as Cys^{I}-Cys^{III} and Cys^{II}-Cys^{IV} (in 10CCa), and Cys^{V}-Cys^{IX}, Cys^{VI}-Cys^{VII}, and Cys^{VIII}-Cys^{X} (in 10CCb) [37,41]. Structurally, the two domains wrap around the bottom face of the propeller, and form strong contacts with the propeller through numerous hydrogen and ionic bonds. Attempts to express either the propeller or the 10CC regions alone were not successful, which suggests that these interactions between propeller and the 10CC region ensure a compact structure and provide stability to the entire unit. However, the 10CC region is mobile and undergoes a pH-dependent conformational change as evidenced for both sortilin and SORL1 [36,37]. For SORL1 the 10CCb-domain exhibits the largest rearrangement, with a “lever-like” motion when the pH increases from acidic conditions with the 10CCb-domain is tightly associated with the propeller, to more neutral conditions with the 10CCb-domain forming almost no contacts. Keeping in mind that full-length SORL1 is a modular multi-domain protein, such a movement may affect the overall receptor conformation and is likely relevant for ligand binding activity and possibly receptor dimerization as it traffics between cellular compartments with different pH conditions.

Andersen
Supplemental Figure S7 - VPS10p-domain

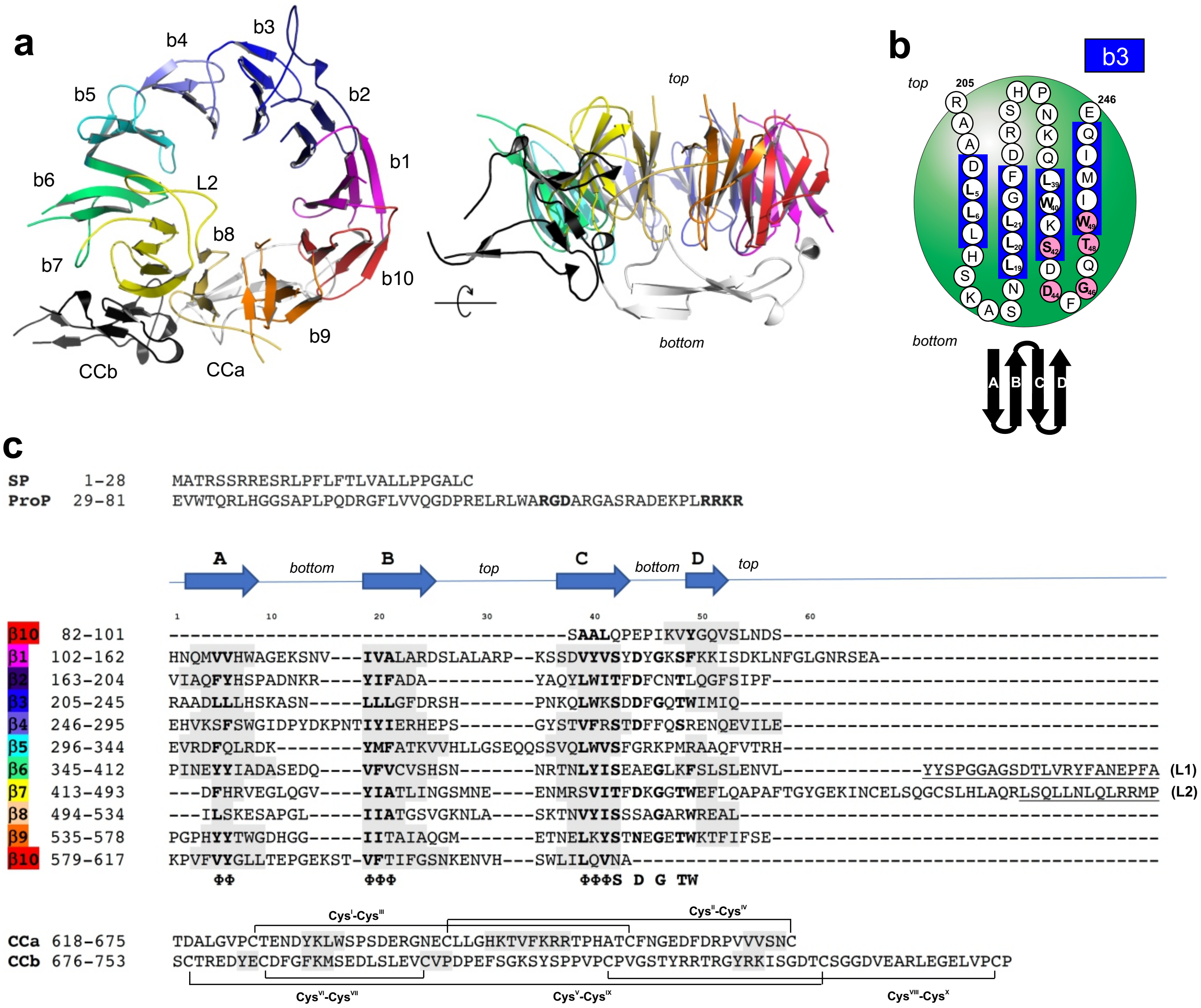

c

```
SP     1-28    MATRSSRRESRLPFLFTLVALLPPGALC
ProP   29-81   EVWTQRLHGGSAPLPQDRGFLVVQGDPRELRLWARGDARGASRADEKPLRRKR
```

```
                  A          bottom     B         top        C      bottom D   top
               1        10        20        30        40        50        60
β10  82-101    ----------------------------------SAALQPEPIKVYGQVSLNDS--------------------------------------
β1   102-162   HNQMVVHWAGEKSNV---IVALARDSLALARP--KSSDVYVSYDYGKSFKKISDKLNFGLGNRSEA--------------------------
β2   163-204   VIAQFYHSPADNKR----YIFADA----------YAQYLWITFDFCNTLQGFSIPF------------------------------------
β3   205-245   RAADLLLHSKASN-----LLLGFDRSH-------PNKQLWKSDDFGQTWIMIQ---------------------------------------
β4   246-295   EHVKSFSWGIDPYDKPNTIYIERHEPS-------GYSTVFRSTDFFQSRENQEVILE-----------------------------------
β5   296-344   EVRDFQLRDK--------YMFATKVVHLLGSEQQSSVQLWVSFGRKPMRAAQFVTRH-----------------------------------
β6   345-412   PINEYYIADASEDQ----VFVCVSHSN-------NRTNLYISEAEGLKFSLSLENVL-------------YYSPGGAGSDTLVRYFANEPFA (L1)
β7   413-493   ---DFHRVEGLQGV----YIATLINGSMNE----ENMRSVITFDKGGTWEFLQAPAFTGYGEKINCELSQGCSLHLAQRLSQLLNLQLRRMP (L2)
β8   494-534   ---ILSKESAPGL-----IIATGSVGKNLA----SKTNVYISSSAGARWREAL---------------------------------------
β9   535-578   PGPHYYTWGDHGG-----IITAIAQGM-------ETNELKYSTNEGETWKTFIFSE------------------------------------
β10  579-617   KPVFVYGLLTEPGEKST-VFTIFGSNKENVH---SWLILQVNA-------------------------------------------------
                   ΦΦ            ΦΦΦ                 ΦΦΦS D G TW
```

```
                       Cysᴵ-Cysᴵᴵᴵ                    Cysᴵᴵ-Cysᴵⱽ
CCa  618-675   TDALGVPCTENDYKLWSPSDERGNECLLGHKTVFKRRTPHATCFNGEDFDRPVVVSNC
CCb  676-753   SCTREDYECDFGFKMSEDLSLEVCVPDPEFSGKSYSPPVPCPVGSTYRRTRGYRKISGDTCSGGDVEARLEGELVPCP
                     Cysⱽᴵ-Cysⱽᴵᴵ               Cysⱽ-Cysᴵˣ                 Cysⱽᴵᴵᴵ-Cysˣ
```

3.1.2 Supplemental Figure S7. VPS10p-domain

a. The VPS10p-domain folds into a 10-bladed β-propeller (PDB: 3WSX [37]) presented from the top (left) and from the side (right), with the 10CC-domains located at the bottom of the folded propeller. Each blade is colored according to the code from ref [37].

b. Amino acids 205-246 corresponding to β3 are presented by their one-letter-code. The blue rectangular background indicates the four A-D anti-parallel strands. Asp-box residues are presented on a pink background towards the bottom of the folded domain in the C-D loop. Sheet topology is indicated below.

c. Residues 1-753 in SORL1 comprise a signal peptide (SP; residues 1-28), a pro-peptide (ProP; residues 29-81), 10 repeats forming the VPS10p-domain (residues 82-617), and two parts with conserved cysteines (CCa/b; residues 618-753). Numbering of the sequence follows Uniprot entry Q92673 from as originally described by ref [42]. The 10 repeats are aligned based on the identified four β-strands within each repeat (grey; A-D), with positions of conserved hydrophobic amino acids in strands A-C as well as amino acids of the Asp-box motifs (**S**X**D**X**G**X**TW**) shown in bold. Horizontal lines indicate the locations of the five disulfides that bridge the 10 cysteines in CCa and CCb.

3.1.3 *SORL1* variants in VPS10p-domain

The VPS10p-domain is found only in the 5 proteins of the VPS10p-receptor family, with too little pathological information such that there are no orthologous proteins suitable for evaluation of disease-associated variants. In many cases pathogenicity may be inferred from interrogation of the available crystal structure [37]. This has been done for the p.G511R variant (predicted as "*likely pathogenic*"), which was previously reported to segregate with AD across 2 generations [43]. While the residue is located outside the L1/L2 regions, it maps to a position that fixes one end of the L2 loop that is directly involved in Aβ binding. This suggests that conversion of the small glycine to a large amino acid (i.e. like arginine) could cause a severe disturbance in the conformation (or stability) of L2 with possibly the loss of Aβ-binding ability [37]. In line with this reasoning, functional studies suggested that this mutation impairs binding of Aβ to the VPS10p-domain, leading to decreased lysosomal delivery of Aβ and a consequential increase of secreted Aβ [44]. Taken together, this variant may, at least in part, explain the observed AD in the family [43].

Despite the very small (A528T) or absence (E270K) of a variant effect on AD-risk observed in GWAS studies, functional assays using cells transfected with these variants indicated an impaired ability of mutant SORL1 to decrease APP processing [45]. Inspection of the VPS10p crystal structure and our sequence alignment (**Fig. 4, Supplemental Figure S7c**) is in agreement with neither of these variants being located at very dangerous positions.

3.2 The YWTD-repeated β-propeller (residues 754-1013)

3.2.1 Sequence details

Immediately following the VPS10p- and 10CC-domains, SORL1 contains a region spanning 260 amino acids containing five incomplete copies of a characteristic YWTD-tetrapeptide (**Supplemental Figure S8c, strand B**). In general, a YWTD-repeat region folds into a compact 6-bladed β-propeller, and each blade contains four antiparallel β-strands that are organized around a central pseudo symmetrical axis, forming an internal tunnel. The dimensions of this β-propeller type mimic a 68 Å x 58 Å x 40 Å ellipsoid. Accordingly, this domain is significantly smaller than the 10-bladed VPS10p β-propeller, and there are no reports describing ligands being able to bind inside these narrow tunnels, that often are so tight they appear closed. YWTD-repeated β-propellers are found in all core members of the LDLR family (**Supplemental Figure S8a**) as well as in the physiologically unrelated proteins Nidogen, Osteonidogen, and the precursor of EGF [46].

Crystal structures of homologous domains from LDLR [47], LRP4 [48], LRP6 [49-52], ApoER2 [53] and Nidogen [54] have all been solved, showing how only 5 Å separates the N- and C-terminal residues of the domain in space although they are separated by 260 amino acids in the primary structure (**Supplemental Figure S8**).

Alignment – unique residues

As in homologous YWTD-propellers, the YWTD-motif is absent from the β1 blade where the tetrapeptide is represented by the ^{760}FILY^{763} sequence (**Supplemental Figure S8c**). For blades β2-β6, the YWTD-motifs are located in the second (B) strand (**Supplemental Figure S8c**). The conserved aromatic residues at the Tyr and Trp positions (16 and 17 of each blade) are embedded inside the domain and contribute to an apparent hydrophobic core. The aspartate residues at position 19 participate in extensive hydrogen bonding with residues in strand A and C of the same blade and strand A of the adjacent blade, thereby maintaining the structural integrity of the β-propeller fold [47] (**Supplemental Figure S8b**). Hydrophobic residues within strand A (positions 6 and 8), B (position 15), and D (positions 41 and 42) are present in blades β2-β6 and add further to a hydrophobic core (**Supplemental Figure S8b,c**). A conserved isoleucine (position 27) also contributes to stabilization of the hydrophobic contacts. An arginine is frequently located at position 29 in strand C, where it also functions in domain stabilization through interactions with the Tyr of the YWTD-motif. Three of the six β-propeller blades include a conserved Pro residue at position 3, which supports the loop-

structure between blades. Furthermore, a Gly (position 35) and Leu (position 47) represent conserved positions in most YWTD-domain sequences including that of SORL1 (**Fig. 4, Supplemental Figure S8**). Together, the conserved residues in the YWTD-domain of SORL1 point towards an essential role in maintaining the rigidity of the propeller.

A SBiN-type YWTD-domain in SORL1

To understand how YWTD-domains interact with binding partners, several studies have analyzed the structures of ligand-bounded YWTD-domains [54]. The ligand often contains an Asn-Ile pair (the NXI-pair, with the N and I residues separated by a variable residue) that binds a motif called the *Shutter Binding NXI* (SBiN)-motif, found in several YWTD-domain sequences [48 55]. The Asn side chain of the ligand NXI-pair interacts with a Trp (pos 20), a Phe (pos 4) and an Asn (pos 4) of the β-propeller, whereas the Ile of the ligand NXI-pair engages with an additional paired Trp (pos 20) - Arg (pos 4) of the YWTD-domain [54]. In aggregate, the residues at these five positions all locate to positions 4 or 20 of the β-blade sequence alignment and make up the SBiN-motif [48,55]. The SORL1 YWTD-propeller contains an SBiN-motif composed of residues W895 (β4, pos 20), N924 (β5, pos 4), Y964 (β6, pos 4), and the R879-W978 (β4 pos 4-β6 pos 6) pair (**Supplemental Figure S8c – residues highlighted, black background**). All five residues are brought together at the top of the folded domain in line with their position in the sequence either before strand A or at the end of strand B.

The SORL1 sequence contains five *internal* NXI-pairs, which suggests that other SORL1 domains may also serve as ligand to the β-propeller to form intramolecular (*intrinsic*) contacts. The five internal NXI-pairs are located in blades β1 and β4 of the VPS10p-domain (^{115}NVI^{117} and ^{262}NTI$^{264)}$, in the first CR-domain (^{1089}NCI^{1091} and ^{1092}NSI^{1094}), and in the sixth 3Fn-domain (^{2105}NQI^{2107}). As the two NXI sequences within the VPS10p-domain are situated at the bottom of the large β-propeller (in agreement with their position in the primary sequence between strands A and B), it is unlikely they would be in contact with the YWTD-domain SBiN motif. The recently determined model of the full SORL1 protein ectodomain by AlphaFold [56] shows no indication that internal NXI-pairs bind to the YWTD-domain.

Takes two to tangle

Our phylogenetic analysis indicates that in all known SORL1 receptors the single YWTD-propeller coexists with the preceding VPS10p β-propeller, suggesting these two domains form a single rigid unit (**Supplemental Figure S13**). This is in agreement with the crystal structure of the extracellular domain of LRP6, which contains four YWTD-propellers that form pairs of two rigid structural blocks, with a short intervening hinge that restrains their relative orientation [50]. This pairing is observed in several proteins with multiple YWTD-domains (i.e. LRP4 and

LRP6): it enables interactions with large ligands including co-receptors in multimeric complexes, or large soluble ligands requiring two adjacent β-propellers for efficient binding [50] [48]. It is tempting to speculate that both the VPS10p and YWTD β-propellers of SORL1 exclusively bind ligands to their top faces. The SBiN residues in the YWTD domain locate to the top face (**Supplemental Figure S8c**), while an EGF-domain, located to the C-terminal end of the β-propeller, forms intimate domain-domain interactions with the bottom face, making it unavailable for ligand-interactions [57]. Similarly, the bottom face of the VPS10p-domain is occupied by the 10CC-domains, such that this side is also unavailable for ligand-interactions.

Andersen
Supplemental Figure S8 - YWTD-domain

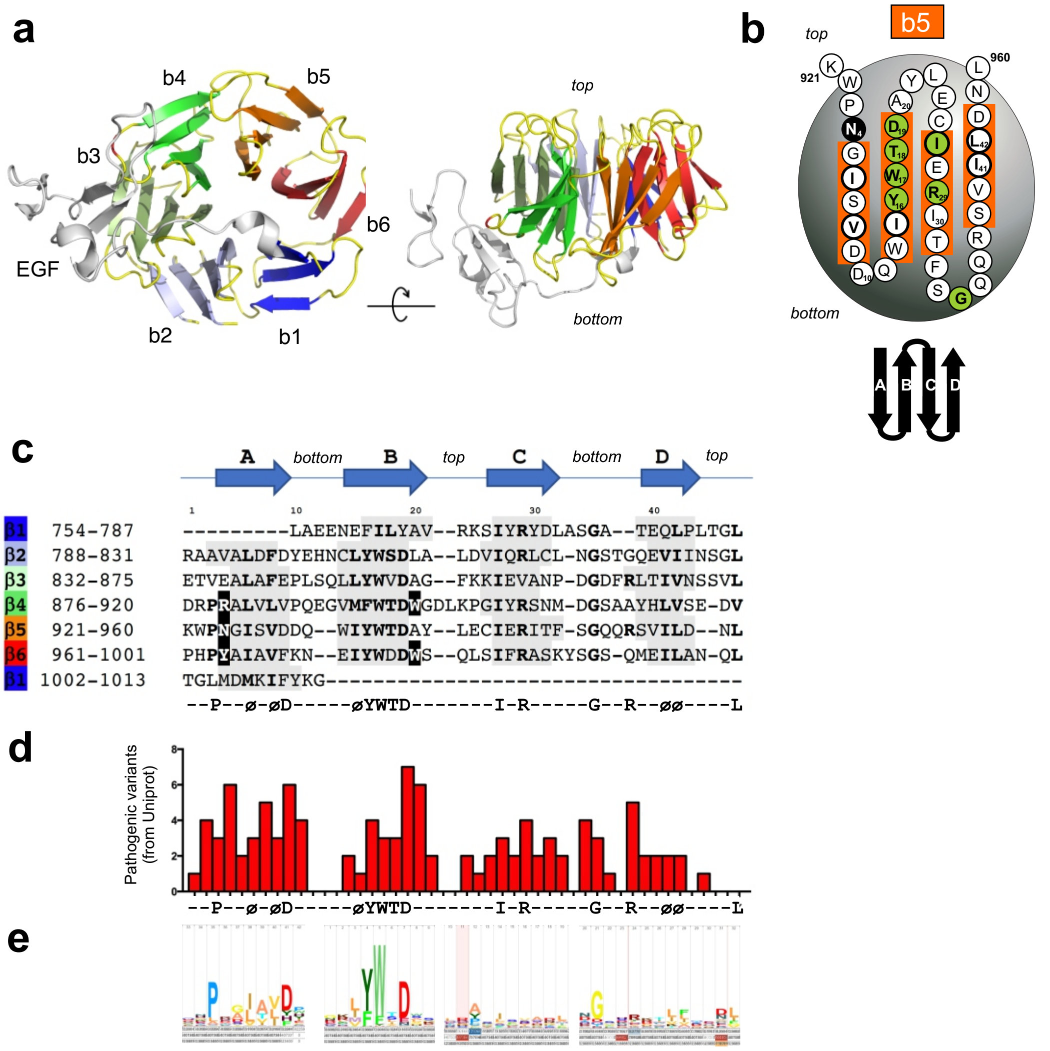

3.2.2 Supplemental Figure S8. YWTD-domain

a. The YWTD-domain of LDLR folds into a 6-bladed β-propeller (PDB: 1IJQ [47]), likely reflecting a similar structure for the YWTD-domain of SORL1. The propeller is shown from the top (left) and from the side (right). The EGF-domain of LDLR is shown in light grey at the bottom of the propeller.

b. Schematics of the fifth β-sheet (β5) of the β-propeller (gray circle), depicting residues 921-960 by their one-letter-code with indication of the four A-D anti-parallel strands in orange. The YWTD-motif in strand B together with some of the few additionally conserved residues of each repeat are presented with green circles and bold letters. Blade topology is shown below.

c. Alignment of the SORL1 sequence spanning residues 754-1013 and corresponding to an YWTD-domain was done based on the YWTD-motif and the presence of the predicted four β-strands of each repeat (grey background). Lower case letters or capital letters indicate positions occupied by similar or identical residues in each repeat, respectively. Residues R^{879}, W^{905}, N^{924}, Y^{964}, W^{979} forming the SBiN-motif are shown in white letters on a black background.

d. Histograms showing the number of pathogenic variants that occur for each YWTD-domain position as listed in **Supplemental Information 3.2.6**.

e. Logo representation of the domain sequence conservation: the larger the letter the higher the conservation across YWTD-domain sequences.

3.2.3 *SORL1* variants in YWTD-domain

YWTD-motif at positions 16-19:

Not surprisingly, all four positions (16-19) of the tetrameric YWTD-sequence are potentially deleterious of protein/domain function (**Supplemental Figure S8d**). **Tyr (pos 16),** was identified to be mutated in LDLR1, LRP2, and twice in LRP5 for different patient/diseases. Intriguingly, for each disease-variant the Tyr is replaced by a His: p.Y442H^{LDLR} (identified in patients with FHCL1; [58]), p.Y2522H^{LRP2} (considered causal of Donnai-Barrow syndrome (DBS); [59]), p.Y733H^{LRP5} (identified in a patient with OPPG; [60]), and p.Y1168H^{LRP5} (identified in woman with total retinal detachment and retinoschisis (EVR4); [61]) (**Supplemental Information 3.2.5**). This suggests that introduction of a His is particularly damaging for position 16 of the propeller-domain. **Trp (pos 17)**, was frequently mutated in patients with FH (W577S^{LDLR} [62], W577G^{LDLR} [63], W577R^{LDLR} [58], and functional characterization of mutant proteins with either Gly substitution [63] or Ser substitution [64] showed that substitutions completely abolish receptor membrane expression and LDL uptake. **Thr (pos 18)** in β-propellers of LRP5 (p.T390K^{LRP5}) associates with increased risk for osteoporosis-pseudoglioma syndrome (OPPG) [60] or is considered causal (p.T253I^{LRP5}) of Osteopetrosis, Autosomal Dominant 1 (OPTA1) in two related families on Fyn in Denmark [65], respectively. **Asp** (**pos 19),** mutations of this residue in LDLR are linked to familial hypercholesterolaemia (FH) (p.D492N^{LDLR} [66], p.D579N^{LDLR} [62,67,68] (for this variant less than 2% of receptor activity is reported) and p.D579Y^{LDLR} [69]), while mutations in LRP5 or LRP4 are found in patients with OPPG (p.D434N^{LRP5} [60]) or considered causal of Cenani-Lenz syndactyly syndrome (CLSS) (p.D529N^{LRP4} [70], p.D1403H^{LRP4} [71]), respectively (**Supplemental Information 3.2.5**). Taken together, this suggests that variants at these positions in *SORL1* may be risk-increasing or even causative for AD, and should alert the clinical geneticist as variants with high priority when observed in a patient-carrier. In line with this p.D806N occurring at position 19 was observed so far only in AD patients and not controls [72].

SBiN-motif at positions 4 and 20:

In proteins that contain a YWTD-repeated β-propeller with SBiN-motifs, three of the six blades include residues of the ligand-binding SBiN motifs (i.e. β4-β6) at positions 4 and 20 (**Supplemental Figure S8d**) [55]. Variants that map to these positions in blades β4-β6 may be more pathogenic than if they map to β1-β3. This is supported by the pathogenicity of variants listed in Uniprot in LDLR, LRP4, and LRP5 that affect the SBiN-residues: p.N564S^{LDLR} [73] and p.N564H^{LDLR} [62,66,74-76] (position 4 of β5) in patients with FH. Functional analysis of p.N564H^{LDLR} found 64-73% reduced uptake and

degradation of LDL in fibroblasts from heterozogous and compound carriers [75,76]. Variant p.W1186S^{LRP4} was identified in patient with Sclerosteosis (SOST2) [77] and show impaired Wnt-suppressing activity of the mutant receptor [78], and p.W478R^{LRP5} (position 20 both in β4) in a family with OPPG where the variant segregate with affected subjects [79], respectively. Interestingly, *SORL1* variant p.N924S (conservation of Asn: 40/40 if **Supplemental Information 3.8**) affects the Asn residue in the SBiN-motif (position 4 in β5) has been reported in patients with AD [72], but there is no functional studies nor genetic statistical support to yet claim this variant to be pathogenic.

<u>Arg at position 29:</u>

In SORL1, 5 of 6 YWTD blades contain an Arg at position 29. Uniprot lists 4 pathogenic variants for this position, corresponding to substitution of an Arg: p.R570W^{LRP5} (patients with OPPG [60,80]), p.R570Q^{LRP5} (patients with EVR [60,81]), p.R1277H^{LRP4} (patients with CMS17 [77]), and p.R473Q^{LRP6} (segregate with disease in a family with metabolic syndrome; ADCAD2 [82]). This suggests that removal of Arg at position 29 may increase risk of AD. The preference for Arg at this position is not obvious from the logo-web consensus (**Supplemental Figure S8e**), but is noticeable in our alignments (**Supplemental Information 3.2.4**).

<u>Asp at position 9:</u>

Position 9 is the top position for mutations in YWTD-propellers (**Supplemental Figure S8d**) with 6 disease-associated variants listed in Uniprot: p.D482H^{LDLR} (in FHCL1 patients, listed as causal genetic variant [83,84]), p.D203N^{LRP5} (in OPPG patients [60]), p.D381N^{LRP5} (identified in a small family with familial EVR1 and functional analysis showed mutations lead to complete receptor inactivity [85]), p.D511A^{LRP5} (identified in a small family with familial EVR4 [86]), p.D683N^{LRP5} (identified in patients with OPPG [60]), and p.T852M^{LRP5} (identified in a family with EVR4 and determined as pathogenic because of 95% reduction in LRP5 activity [87]). Notably, there is also preference of Asp at position 9 in the consensus sequence (**Supplemental Information 3.2.4**), and the above listed mutations strongly suggest that substitutions that replace an Asp is very likely disease-associated. In the SORL1 sequence only two of the six β-blades have an Asp at position 9; D794 (β2) and D929 (β5) (**Fig. 4**). The *SORL1* variant p.D929Y has been identified in both a control and an AD case leaving it an open question still whether this is a dangerous position for the SORL1 domain [72].

We would like to provide an example how to apply our species conservation tool, trying to decide whether the variant leading to p.V884M substitution is dangerous. Based on the identification of this variant in 4 AD cases and not yet in any controls [72], it could be speculated that this variant is pathogenic. But when using the alignment of multiple SORL1 sequences from 40 different species (**Supplemental Information 3.8**), it is evident that there is no specific requirement for a valine at

this position of SORL1 as other hydrophobic residues as Leu and Ile are present at this position for other species, and several species even carry a Met at this position (e.g. SORL1 from horse, salmon, pike, piranha, and zebrafish). This strongly suggests that the p.V884M is a benign mutation.

<u>*Arg at position 38:*</u>

Our DMDM analysis based on pathogenic variants listed in Uniprot identified 4 variants for position 38 that associate with four diseases: one in LDLR: p.R595W^{LDLR} (in FHCL1 patients [66]), and three in LRP5: p.R494Q^{LRP5} (in patients with OPPG [60,80]), p.R752G^{LRP5} (in a family with EVR4 [81]), and p.R1188W^{LRP5} (segregate with disease in large family pedigrees with PCLD4 [88]). Although no strong preference for an Arg in the SORL1 sequence (**Fig. 4**), but a slight enrichment in the bigger sequence alignment for the β-blade at this position (**Supplemental Information 3.2.4**), it is interesting that each of the four disease-variants involve the replacement of this positively charged amino acid. In SORL1 two of the YWTD blades have an Arg at position 38: R866 (β3) and R953 (β5) (**Fig. 4**). We suggest that variants that affect these two amino acids may be deleterious of SORL1 function and associated with AD. The ADES-ADSP dataset includes p.R953H in three cases with very early ages at onset ranging between 46 and 58 years, and not in controls [72].

3.2.4 YWTD-domain alignment

Mapping of naturally occurring variants found in human YWTD-domain containing proteins, and being listed as associated with pathology at www.uniprot.org. The alignment follows the SORL1 alignment shown on top, and pathogenic variants are highlighted on a red background.

```
1        10        20        30        40
         LAEENEFILYAV--RKSIYRYDLASGA--TEQLPLTGL
RAAVALDFDYEHNCLYWSDLA--LDVIQRLCL-NGSTGQEVIINSGL
ETVEALAFEPLSQLLYWVDAG--FKKIEVANP-DGDFRLTIVNSSVL
DRPRALVLVPQEGVMFWTDWGDLKPGIYRSNM-DGSAAYHLVSE-DV
KWPNGISVDDQ--WIYWTDAY--LECIERITF-SGQQRSVILD--NL
PHPYAIAVFKN--EIYWDDWS--QLSIFRASKYSGS-QMEILAN-QL
TGLMDMKIFYKG-----------------------------------
  p  Φ Φd     ΦYWTD       I R    dG   r  ΦΦ     L
LDLR:
        KAVGSIAYLFFTN--RHEVRKMTL-DRSEYTSLIPNL
RNVVALDTEVASNRIYWSDLS--QRMICSTQLDRAHGVSSYDTVISRDI
QAPDGLAVDWIHSNIYWTDSV--LGTVSVADT-KGVKRKTLFRENG
SKPRAIVVDPVHGFMYWTDWG-TPAKIKKGGL-NGVDIYSLVTENI
QWPNGITLDLLSGRLYWVDSK--LHSISSIDVNGGN-RKTILEDEKRL
AHPFSLAVFED--KVFWTDII--NEAIFSANRLTGS-DVNLLAENL
LSPEDMVLFHN
LRP2_6:
         AISTENFLIFALSNSLRSLHLDPENHSPPFQTINVE
RTVMSLDYDSVSDRIYFTQNLASGVGQISYATLSSGIHTPTVIASGI
GTADGIAFDWITRRIYYSDYL--NQMINSMAE-DGSNRTVIARV
PKPRAIVLDPCQGYLYWADWD-THAKIERATL-GGNFRVPIVNSSL
VMPSGLTLDYEEDLLYWVDAS--LQRIERSTL-TGVDREVIVNAA
VHAFGLTLYGQ--YIYWTDLY--TQRIYRANKYDGSGQIAMTTNLL
SQPRGINTVVKNQKQQ
LRP5_1:
       PAPAAASPLLLFAN---RRDVRLVDAGGVKLESTIVVSGL
EDAAAVDFQFSKGAVYWTDVS-EEAIKQTYLNQTGAAVQNVVISGL
VSPDGLACDWVGKKLYWTDSE--TNRIEVANL-NGTSRKVLFWQDL
DQPRAIALDPAHGYMYWTDWG-ETPRIERAGM-DGSTRKIIVDSDI
YWPNGLTIDLEEQKLYWADAK--LSFIHRANL-DGSFRQKVVEGSL
THPFALTLSGD--TLYWTDWQ--TRSIHACNKRTGGKRKEILSAL
YSPMDIQVLSQERQPFFHTR
LRP5_2:
         KAGAEEVLLLAR--RTDLRRISL-DTPDFTDIVLQVDDI
RHAIAIDYDPLEGYVYWTDDE--VRAIRRAYL-DGSGAQTLVNTEI
NDPDGIAVDWVARNLYWTDTG--TDRIEVTRL-NGTSRKILVSEDL
DEPRAIALHPVMGLMYWTDWG-ENPKIECANL-DGQERRVLVNASL
GWPNGLALDLQEGKLYWGDAK--TDKIEVINV-DGTKRRTLLED
KLPHIFGFTLLGDFIYWTDWQ--RRSIERVHK-VKASRDVIID
QLPDLMGLKAVNVAKVVGTNP
LRP5_3:
          IVPEAFLVFTS--RAAIHRISL-ETNNNDVAIPLTGV
KEASALDFDVSNNHIYWTDVS--LKTISRAFM-NGSSVEHVVEFGL
DYPEGMAVDWMGKNLYWADTG--TNRIEVARL-DGQFRQVLVWRDL
DNPRSLALDPTKGYIYWTEWG-GKPRIVRAFM-DGTNCMTLVDKV
GRANDLTIDYADQRLYWTDLD--TNMIESSNM-LGQER-VVIAD
DLPHPFGLTQYSDYIYWTDWN--LHSIERADKTSGRNR-TLIQGHL
```

```
        DFVMDILVFHSSRQDGLND
LRP5_4:
                  SPPTTFLLFSQ--KSAISRMIPDDQHSPDLILPLHGL
        RNVKAIDYDPLDKFIYWVDGR---QNIKRAKD-DGTQPFVLTSLSQGQNPD
        RQPHDLSIDIYSRTLFWTCEA--TNTINVHRL-SGEAMGVVLRGDR
        DKPRAIVVNAERGYLYFTNMQDRAAKIERAAL-DGTEREVLFTTGL
        IRPVALVVDNTLGKLFWVDAD--LKRIESCDL-SGANRLTLEDANI
        VQPLGLTILGK--HLYWIDRQ--QQMIERVEKTTGDKRTRIQGRVAHLTGI
        HAVEEVSLEEFSAHP
LRP4_1:
                KALGPEPVLLFAN--RIDIRQVLP-HRSEYTLLLNNL
        ENAIALDFHHRRELVFWSDVT--LDRILRANL-NGSNVEEVVSTGL
        ESPGGLAVDWVHDKLYWTDSG--TSRIEVANL-DGAHRKVLLWQNL
        EKPRAIALHPMEGTIYWTDWG-NTPRIEASSM-DGSGRRIIADTHL
        FWPNGLTIDYAGRRMYWVDAK--HHVIERANL-DGSHRKAVISQGL
        PHPFAITVFED--SLYWTDWH--TKSINSANKFTGKNQ-EIIRNKL
        HFPMDIHTLHPQRQPAGKN
LRP4_2:
           ISSHACAQSLDKFLLFAR--RMDIRRISF-DTEDLSDDVIPLADV
        RSAVALDWDSRDDHVYWTDVS--TDTISRAKW-DGTGQEVVDTSL
        ESPAGLAIDWVTNKLYWTDAG--TDRIEVANT-DGSMRTVLIWENL
        DRPRDIVVEPMGGYMYWTDWG-ASPKIERAGM-DASGRQVIISSNL
        TWPNGLAIDYGSQRLYWADAG--MKTIEFAGL-DGSKRKVLIGSQL
        PHPFGLTLYGE--RIYWTDWQ--TKSIQSADRLTGLDRETLQENLENLMDIHVFHRRRP
        PVSTPCAMEN
LRP4_3:
    PTGINLLSDGKTCSPGMNSFLIFAR--RIDIRMVSL-DIPYFADVVVPINITM
        KNTIAIGVDPQEGKVYWSDST--LHRISRANL-DGSQHEDIITTGL
        QTTDGLAVDAIGRKVYWTDTG--TNRIEVGNL-DGSMRKVLVWQNL
        DSPRAIVLYHEMGFMYWTDWG-ENAKLERSGM-DGSDRAVLINNNL
        GWPNGLTVDKASSQLLWADAH--TERIEAADL-NGANRHTLVSPV
        QHPYGLTLLDS--YIYWTDWQ--TRSIHRADK--GTGSNVILVRS
        NLPGLMDMQAVDRAQPLGF
LRP4_4:
                 DPSPETYLLFSS--RGSIRRISL-DTSDHTDVHVPVPEL
        NNVISLDYDSVDGKVYYTDVF--LDVIRRADL-NGSNMETVIGRGL
        KTTDGLAVDWVARNLYWTDTG--RNTIEASRL-DGSCRKVLINNSL
        DEPRAIAVFPRKGYLFWTDWG-HIAKIERANL-DGSERKVLINTDL
        GWPNGLTLDYDTRRIYWVDAH--LDRIESADL-NGKLRQVLVSHV
        SHPFALTQQDR--WIYWTDWQ--TKSIQRVDKYSGRNKETVLANVEGL
        MDIIVVSPQRQTGTNA
LRP6_1:
                VLLRAAPLLLYAN--RRDLRLVDATNGKENATIVVGGL
        EDAAAVDFVFSHGLIYWSDVS--EEAIKRTEFNKTESVQNVVVSGL
        LSPDGLACDWLGEKLYWTDSE--TNRIEVSNL-DGSLRKVLFWQEL
        DQPRAIALDPSSGFMYWTDWG-EVPKIERAGM-DGSSRFIIINSEI
        YWPNGLTLDYEEQKLYWADAK--LNFIHKSNL-DGTNRQAVVKGSL
        PHPFALTLFED--ILYWTDWS--THSILACNKYTGEGLREIHSDI
        FSPMDIHAFSQQRQPNATNP
LRP6_2:
                 KDGATELLLLAR--RTDLRRISL-DTPDFTDIVLQLEDI
        RHAIAIDYDPVEGYIYWTDDE--VRAIRRSFI-DGSGSQFVVTAQI
        AHPDGIAVDWVARNLYWTDTG--TDRIEVTRL-NGTMRKILISEDL
        EEPRAIVLDPMVGYMYWTDWG-EIPKIERAAL-DGSDRVVLVNTSL
        GWPNGLALDYDEGKIYWGDAK--TDKIEVMNT-DGTGRRVLVED
        KIPHIFGFTLLGDYVYWTDWQ--RRSIERVHK-RSAEREVIIDQLPDLMGLKATNVHRVIG
        SNPMGAVLRSLLA
LRP6_3:
                  IVPEAFLLFSR--RADIRRISL-ETNNNNVAIPLTGV
        KEASALDFDVTDNRIYWTDIS--LKTISRAFM-NGSALEHVVEFGL
        DYPEGMAVDWLGKNLYWADTG--TNRIEVSKL-DGQHRQVLVWKDL
        DSPRALALDPAEGFMYWTEWG-GKPKIDRAAM-DGSERTTLVPNV
        GRANGLTIDYAKRRLYWTDLD--TNLIESSNM-LGLNR-EVIAD
        DLPHPFGLTQYQDYIYWTDWS--RRSIERANKTSGQNR-TIIQGHL
        DYVMDILVFHSSRQSGWNE
```

LRP6_4:

SAPTT**FLLF**SQ--KSA**I**N**R**MVI-DEQQSPD**II**LPIHSL
RNVRA**I**D**Y**DPLDKQ**LYWID**SR--QNM**I**RKAQE-D**G**SQGFT**VV**VSSVPSQNLE
IQPYD**L**S**I**DIYSRY**IYWTC**EA--TNV**I**NVTRL-D**G**RSVGV**VL**KGEQ
DRPRA**V**V**V**NPEKGY**MYFTN**LQERSPK**I**E**R**AAL-D**G**TEREV**LF**FSG**L**
SKPIA**L**A**L**DSRLGK**LFWAD**SD--LRR**I**ESSDL-S**G**ANR-I**VL**EDSNI
LQPVG**L**T**V**FEN--W**LYWID**KQ--QQM**I**EKIDM-T**G**REGRTK**V**QAR**I**
AQLSD**I**H**A**VKELNLQEYRQHP

3.2.5 Identity of mapped YWTD variants: disease proteins and disease variants

Summary of included proteins containing naturally occurring variants associated with diseases in domains shared with SORL1.

Gene	Protein	Uniprot	#dom	Associated Diseases/Syndromes (CODE)	Variants mapped onto big alignments (from Uniprot entries except where a specific ref is provided)	#variants	#positions	Citations for variants discussed in main text
LDLR	Low-density lipoprotein receptor	P01130	1x6	Familial hypercholesterolemia (FHCL1)	**FHCL1**: A399D, L401V, F403L, T404P, R406W, E408K, L414R, D415G, R416W, I423T, V429M, A431T, L432V, D433H, T434K, **Y442H**, I451T, T454N, L479P, D482H, W483R, **D492N**, V523M, P526S, G549D, N564H/N564S, L568V, R574C/R574H, **W577G/W577S/W577R**, **D579N/D579Y**, I585T, G592E, **R595W**, D601H, P608S, R633C, V639D, P649L	43	38	**Y422H** identified in two patients with FHCL1 [58]<br>**D482H** as causal of FH [83,84]<br>**R595W** in FH pts [66]<br>**D492N** in FH pts [66]<br>**N564S** in Polish familial FH [73]<br>**N564H** in Brazilian, German, French and Danish familial FH [62,66,74-76]<br>**W577G** in FH pts, and functional test show no membrane expression and zeron LDL uptake, ie pathogenic [63]<br>**W577R** in FH pts [58]<br>**W577S** in FH pts [62] and functional test showed no

								LDLR maturation nor surface expression [64]<br>**D579N** has less than 2% receptor activity [62,67,68]<br>**D579Y** in FH pts [69]
LRP4	Low-density lipoprotein receptor-related protein 4	O75096	4x6	Cenani-Lenz syndactyly syndrom (CLSS)<br>Sclerosteosis 2 (SOST2)<br>Myasthenic syndrome, congenital, 17 (CMS17)	**CLSS**: D449N, T461P, L473F, **D529N**, I450V [71], L953P [71],<br>**Syndactyly**: **D1403H** [89], Q1564K [89]<br>**SOST2**: R1170W, **W1186S**<br>**CMS1**: E1233K, **R1277H**	12	12	**W1168S** in SOST2 pts and with impaired Wnt-suppressing activity [77,78]<br>**R1277H** in patients [77]
LRP5	Low-density lipoprotein receptor-related protein 5	O75197	4x6	Vitreoretinopathy, exudative 1 (EVR1)<br>Vitreoretinopathy, exudative 4 (EVR4)<br>Osteoporosis-pseudoglioma syndrome (OPPG)<br>High bone mass trait (HBM)<br>Endosteal hyperostosis, Worth type (WENHY)<br>Osteopetrosis, autosomal dominant 1 (OPTA1)<br>Polycystic liver disease 4 with or without kidney cysts (PCLD4)	**EVR1**: **D381N**, R348W<br>**EVR4**: L145F, T173M, A422T, E441K, R444C, **D511A**, A522T, T535M, L540P, G550R, **R570Q**, **R752G**, T798A, R805W, **T852M**, N1182D, **Y1168H**<br>**OPPG**: **D203N**, T244M, R348W, R353Q, S356L, **T390K**, A400E, G404R, T409A, **D434N**, E460K, **W478R**, R494Q, W504C, **G520V**, N531I, **R570W**, D683N, **Y733H**, D1099Y, R1113C<br>**HBM**: R154M, M282V<br>**WENHY**: A214T/A214V, A242T<br>**OPTA1**: D111Y, G171R, A242T, **T253I**,<br>**PCLD4**: V454M, V684A,	49	47	D203N, T390K, D434N, G520V, Y733H identified in OPPG patients [60]<br>**W478R** in a family with OPPG and segregates with disease [79]<br>**D381N** in a family with EVR. Functional test show complete mutant receptor inactivation [85]<br>**D511A** in a family with EVR [86]<br>**R570W** in pts with OPPG [60,80]<br>**R570Q** in pts with EVR [60,81]<br>**R752G** in a family with EVR [81]<br>**Y1168H** identified in patient with EVR [61]. Further

								evidence needed to establish if causal **T852M** in a family with EVR and pathogenic based on 95% reduced mutant activity [87] **T253I** most likely disease causing and segregate in two (related) families on Fyn/Denmark [65]
LRP6	Low-density lipoprotein receptor-related protein 6	O75581	4x6	Coronary artery disease, autosomal dominant, 2 (ADCAD2)	**ADCAD2**: R360H, N433S, **R473Q**	3	3	**R473Q** segregate with disease in a family with metabolic syndrome [82]
LRP2	Low-density lipoprotein receptor-related protein 2	P98164	8x6	Donnai-Barrow Syndrom (DBS)	**DBS**: **Y2522H** [59]	1	1	**Y2522H** causal of DBS [59]
						**108**	101	

3.2.6 YWTD disease variants listed according to domain positions

Disease-mutations domain-mapping analysis with identification of pathogenic variants in other proteins with YWTD-domains (as listed in **Supplemental Information 3.2.5**). Here variants are mapped onto domain positions following alignment of internally repeated sequences in the SORL1 domain sequences. The number of hits for each position depicted in the bar diagram of **Supplemental Figure 3.2.4**.

**YWTD-domain positions**	Number of hits	Identified variants	Domain conservation	Priority
1	1	p.R360H(LRP6)		
2	4	p.E460K(LRP5), p.W504C(LRP5), p.R805W(LRP5), p.R1113C(LRP5)		
3; p	3	p.V429M(LDLR), p.P608S(LDLR), p.P649L(LDLR)	Loss of Pro	high
4(sbin)	6	p.N564S(LDLR), p.N564H(LDLR), p.D111Y(LRP5), p.R154M(LRP5), p.M282V(LRP5), p.R154M(LRP5)	Loss of SBIN	moderate
5	2	p.A431T(LDLR), p.A242T(LRP5)		
6; ø	3	p.L432V(LDLR), p.L479P(LDLR), p.L473F(LRP4)	Loss of Leu?	moderate
7	5	p.D433H(LDLR), p.V523M(LDLR), p.T244(LRP5), p.A(LRP5), p.G(LRP5)		
8; ø	4	p.T434K(LDLR), p.L568V(LDLR), p.L(LRP4), p.Q(LRP4)		
9; d	6	p.D482H(LDLR), p.D203N(LRP5), p.D381N(LRP5), p.D511A(LRP5), p.D683N(LRP5), p.T852M(LRP5)	Loss of Asp	high
10	4	p.W483R(LDLR), p.P526S(LDLR), p.V684A(LRP5), p.N1121D(LRP5)		
11	0			
12	0			
13	0			
14	2	p.R574C(LDLR), p.R574H(LDLR)		
15; ø	1	p.A399D(LDLR)		moderate
16; **Y**	4	p.Y442H(LDLR), p.Y733H(LRP5), p.Y1168H(LRP5) p.Y2522H(LRP2)[59]	Loss of Tyr	high
17; **W**	3	p.L401V(LDLR), p.W577G(LDLR), p.W577S(LDLR)	Loss of Trp	high
18; **T**	3	p.T253I(LRP5), p.T390K(LRP5), p.G520V(LRP5)	Loss of Thr	high
19; **D**	7	p.F403L(LDLR), p.D492H(LDLR), p.D579N(LDLR), p.D579Y(LDLR), p.D434N(LRP5), p.D529N(LRP4)[71], p.D1403H(LRP4)[71]	Loss of Asp	high
20(sbin)	6	p.T404P(LDLR), p.A214T(LRP5), p.A214V(LRP5), p.W478R(LRP5), p.A522T(LRP5), p.W1186S(LRP4)		moderate
21	2	p.G171R(LRP5), p.R348W(LRP5)		
22	0			
23	0			

24	2	p.R406W(LDLR), p.T173M(LRP5)		
25	1	p.E1233K(LRP4)		
26	2	p.E408K(LDLR), pD449N(LRP4)		
27; **I**	3	p.I451T(LDLR), p.I585T(LDLR), p.I450V(LRP4)[71]	Loss of Ile	high
28	2	p.R353Q(LRP5), p.E441K(LRP5)		
29; **R**	4	p.R570Q(LRP5), p.R570W(LRP5), p.R1277H(LRP4), p.R473Q(LRP6)	Loss of Arg	high
30	2	p.T454N(LDLR), p.A400E(LRP5)		
31	3	p.S356L(LRP5), p.R444C(LRP5), p.N531I(LRP5)		
32	2	p.L414R(LDLR)), p.R633C(LDLR)		
33	0			
34; d/n	4	p.D415G(LDLR), p.G592E(LDLR), p.D1099Y(LRP5), p.N433S(LRP6)		
35; **G**	3	p.R416W(LDLR), p.G549D(LDLR), p.G404R(LRP5)		high
36	1	p.T535M(LRP5)		
37	0			
38; R	5	p.R595W(LDLR), p.R494Q(LRP5), p.R752G(LRP5), p.R1188W(LRP5), p.R632H(LRP4)[90]	Loss of Arg	high
39	2	p.V639D(LDLR), p.T461P(LRP4)		
40	2	p.T798A(LRP5), p.T409A(LRP5)		
41; ø	2	p.L145F(LRP5), p.L540P(LRP5)		moderate
42; ø	2	p.I423T(LDLR), p.V454M(LRP5)		moderate
43	0			
44	1	p.D601H(LDLR)		
45	0			
46	0			
47; **L**	0			moderate
	**109**			

3.3 The EGF-domain (residues 1014-1074)

3.3.1 Sequence details

EGF-domains are widely present in proteins with diverse biological functions [91]. This domain typically has ~40 amino acids with several short β-strands containing conserved cysteines invariably forming intradomain disulfide bridges [91]. In the mammalian proteome, EGF-domains are commonly divided into two subgroups: (1) those containing eight cysteines, which often occur in proteins of the extracellular space, (i.e. Laminin, Fibrillin, and the β-subunit of integrins) and (2) those with six cysteines commonly found in LDLRs. Indeed, the YWTD β-propellers in LDLR, LRP4, LRP6, and ApoER2 are most often flanked with 1 or 2 *six*-cysteine-EGF-domains before, and always by at least one *six*-cysteine EGF-domain at their C-terminal end [47,92] (**Fig. 2**). While SORL1 has historically been acknowledged as a member of the LDLR family [42,93,94] it includes only one *eight*-cysteine EGF-domain C-terminal to the β-propeller and none before (**Fig. 1, Fig. 4**). Moreover, the SORL1 EGF-domain contains 61 amino acids (residues 1014-1074; encoded by a single exon 22) such that it is substantially larger than EGF-domains in other LDLR family members. (**Supplemental Information 3.3.4**). Only a single amino acid separates the fourth and fifth cysteines and there is also only a single residue between the sixth and the seventh cysteines of the SORL1 domain [95]. The disulfide connectivity for integrin-type domains is Cys^{I}-Cys^{V}, Cys^{II}-Cys^{IV}, Cys^{III}-Cys^{VI}, and Cys^{VII}-Cys^{VIII}. While, based on the number of the cysteine residues and their spacing in the sequence, the SORL1 EGF-domain might resemble the “integrin-like” type, there is little similarity between the SORL1 EGF-domain and the EGF-domains from integrin β-subunits (**Supplemental Information 3.3.4**). In contrast, several residues from the SORL1-EGF domain can be aligned with the sequence of EGF-domains positioned C-terminal to YWTD β-propellers from the LDLR family with the highest sequence similarity between the two last cysteine residues (**Supplemental Information 3.3.4**). This suggests that the SORL1 EGF-domain is evolutionary related to the LDLR family: the connectivity of the outer six cysteines follows the stereotypic pattern of LDLR EGF-domains, and the two additional central cysteines (Cys^{IV} and Cys^{V}) could allow the formation of a loop onto the regular EGF-domain fold (**Supplemental Figure S9b**).

The solved structure of the YWTD-EGF domain pair from LDLR indicated that the C-terminal EGF-domain stabilizes the 6-bladed YWTD β-propeller. The EGF-domain forms contacts with the propeller through a series of tight hydrophobic interactions with the linker region and the YWTD-domain that assist to fold the combined two-domain structure,

analogous to the association between 10CC- and VPS10p-domain pairs. Indeed, functional studies demonstrated that it was impossible to isolate the propeller without the EGF-domain of LDLR [47]. In LDLR, the EGF-A domain forms a protein-protein interaction with the catalytic domain of the protein PCSK9, which assists in the endocytosis and subsequent lysosomal degradation of LDLR in the liver [96].

Andersen

Supplemental Figure S9 - EGF-domain

a

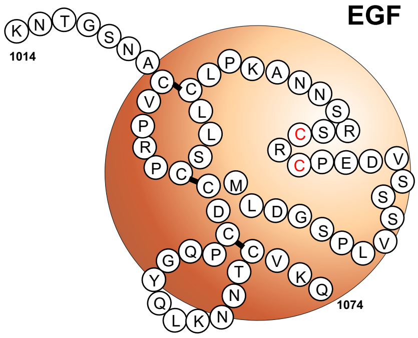

b

disulfides as in integrin-type EGF-domains

```
EGF   1014-1074   KNTGSNACV-----PRPCSLLCLPKANNSRSCRCPEDVSSSVLPSGDLMCDCPQGYQLKNN--TCVKQ   SORLA

                     GVNWCERTTLSNGGCQYLCLPAPQI-------------NPHSPKFTCACPDGMLLARDMRSCL   LDLR
                     -PDACELSVQPNGGCEYLCLPAPQI-------------SSHSPKYTCACPDTMWLGPDMKRCY   APOER2
                     GKNWCEED-MENGGCEYLCLPAPQI-------------NDHSPKYTCSCPSGYNVEENGRDCQ   VLDLR
                     ATNPCGID---NGGCSHLCLMSPVK-----------------PFYQCACPTGVKLLENGKTCK   LRP6_1
                     GSNPCAEE---NGGCSHLCLYRPQG-------------------LRCACPIGFELISDMKTCI   LRP6_2
                     GWNECASS---NGHCSHLCLAVPVG------------------GFVCGCPAHYSLNADNRTCS   LRP6_3
                     RQHPCAQD---NGGCSHICLVKGDG------------------TTRCSCPMHLVLLQDELSCG   LRP6_4
                     GKNRCGDN---NGGCTHLCLPSGQN-------------------YTCACPTGFR-KISSHACA   LRP4_1
                     VSTPCAME---NGGCSHLCLRSPNP-----------------SGFSCTCPTGINLLSDGKTCS   LRP4_2
                     GFNKCGSR---NGGCSHLCLPRPSG-------------------FSCACPTGIQLKGDGKTCD   LRP4_3
                     GTNACGVN---NGGCTHLCFARASD-------------------FVCACPD----EPDSRPCS   LRP4_4
                     FHTRCEED---NGGCSHLCLLSPSE-----------------PFYTCACPTGVQLQDNGRTCK   LRP5_1
                     GTNPCADR---NGGCSHLCFFTPHA-------------------TRCGCPIGLELLSDMKTCI   LRP5_2
                     GLNDCMHN---NGQCGQLCLAIPGG-------------------HRCGCASHYTLDPSSRNCS   LRP5_3
                     SAHPCARD---NGGCSHICIAKGDG------------------TPRCSCPVHLVLLQNLLTCG   LRP5_4
                     G-N-C--D---NGGCS-LCLP-P---------------------Φ-C-CP-GΦ-L--D--TC-
```

disulfides as in LDLR EGF-domains

3.3.2 Supplemental Figure S9. EGF-domain

a. Schematics of the EGF-domain of SORL1 (orange circle), depicting residues 1014-1074 by their one-letter-code with indication of the three speculated disulfides, and the possible long loop including the extra pair of cysteines (the fourth and fifth Cys out of the eight, in red).

b. Alignment of SORL1 residues 1014-1074 with sequences of EGF-domains located at the C-terminal of β-propellers of members from the LDLR family (as presented in **Fig. 2**). All cysteines are shown in red, and residues at positions with strong (capital letters) or weak (lower case letters) conservation are shown below the alignment.

On the top of the SORL1 sequence is indicated the Cys connectivity for the eight Cys residues located within integrin-type EGF-domains (as shown in **Supplemental Information 3.3.4**).

3.3.3 *SORL1* variants in EGF-domain

The importance of the 61-residue EGF-domain in SORL1 as a whole is underscored by a pedigree for a Swedish AD-family carrying variant 11:121437647, which translates to c.3050-2A>G that leads to loss of adjacent splice acceptor site and exclusion of exon 22 – and thus deletion of the entire EGF-domain (p.Gly1017-Glu1074del) [97]. We speculate that lack of the EGF-domain is likely not compatible with folding of a functional receptor, which may also explain the observed co-occurrence of the YWTD-domain with a neighboring EGF-domain at its C-terminal end in LDLRs. Due to the limited sequence similarity between the SORL1 EGF-domain and the EGF-domains in the LDLR proteins it was not possible to accurately assess pathogenic variants in Uniprot that map to the SORL1 EGF-domain.

3.3.4 EGF-domain alignment

We prepared alignments of the SORL1 EGF-domain sequence with homologous domains from LDLR or from Integrin beta2 and beta4. The SORL1 EGF-domain share overall more similarly to YWTD- than integrin-like EGF-domains, despite the presence of 8 Cys residues in the SORL1 domain and ony 6 Cys in the LDLR-type EGF-domains. The color code indicates partial amino acid conservation across the domain sequence.

Alignment between EGF-domain sequences located C-terminal to YWTD-propellers (see Fig 2)

```
LRP6_1:   ATNPCGID---NGGCSHLCLMSPVK-----------------PFYQCACPTGVKLLENGKTCK
LRP6_2:   GSNPCAEE---NGGCSHLCLYRPQG-------------------LRCACPIGFELISDMKTCI
LRP6_3:   GWNECASS---NGHCSHLCLAVPVG------------------GFVCGCPAHYSLNADNRTCS
LRP6_4:   RQHPCAQD---NGGCSHICLVKGDG------------------TTRCSCPMHLVLLQDELSCG
LRP4_1:   GKNRCGDN---NGGCTHLCLPSGQN-------------------YTCACPTGFR-KISSHACA
LRP4_2:   VSTPCAME---NGGCSHLCLRSPNP-----------------SGFSCTCPTGINLLSDGKTCS
LRP4_3:   GFNKCGSR---NGGCSHLCLPRPSG-------------------FSCACPTGIQLKGDGKTCD
LRP4_4:   GTNACGVN---NGGCTHLCFARASD-------------------FVCACPD----EPDSRPCS
LRP5_1:   FHTRCEED---NGGCSHLCLLSPSE-----------------PFYTCACPTGVQLQDNGRTCK
LRP5_2:   GTNPCADR---NGGCSHLCFFTPHA-------------------TRCGCPIGLELLSDMKTCI
LRP5_3:   GLNDCMHN---NGQCGQLCLAIPGG-------------------HRCGCASHYTLDPSSRNCS
LRP5_4:   SAHPCARD---NGGCSHICIAKGDG------------------TPRCSCPVHLVLLQNLLTCG
LDLR:     GVNWCERTTLSNGGCQYLCLPAPQI-------------NPHSPKFTCACPDGMLLARDMRSCL
APOER:     PDACELSVQPNGGCEYLCLPAPQI-------------SSHSPKYTCACPDTMWLGPDMKRCY
VLDLR:    GKNWCEED-MENGGCEYLCLPAPQI-------------NDHSPKYTCSCPSGYNVEENGRDCQ
SORL1: KNTGSNACV-----PRPCSLLCLPKANNSRSCRCPEDVSSSVLPSGDLMCDCPQGYQLKNNT--CVKQ
```

Alignment with EGF-domains from integrin beta2 and beta4 following a published alignment of these domains in refs [95,98]

```
Beta2_1:      CR---------DQSR----DRSLCHG----KGFLEC-------------GICRCDT----GY--IGKNCE
Beta2_2:      CQTQGRSSQELEGSCRKDNNSIICSG----LGDCVC-------------GQCLCHTSDVPGKLIYGQYCE
Beta2_3:      CD---------TINCERY-NGQVCGG--PGRGLCFC-------------GKCRCHP----GF--EGSACQ
Beta2_4:      CER-------TTEGCLNP-RRVECSG----RGRCRC-------------NVCECHS----GY--QLPLCQ
Beta4_1:      CE--------LQKEV----RSARCSF----NGDFVC-------------GQCVCSE----GW--SGQTCN
Beta4_2:      CST-G--SLSDIQPCLREGEDKPCSG----RGECQC-------------GHCVCYGE---GR-YEGQFCE
Beta4_3:      Y---------DNFQCPRT-SGFLCND----RGRCSM-------------GQCVCEP----GW--TGPSCD
Beta4_4:      CPL-------SNATCIDS-NGGICNG----RGHCEC-------------GRCHCHQQ----SLYTDTICEINYS
SORL1: KNTGSNACV---------PRPCS-----LLCLPKANNSRSCRCPEDVSSSVLPSGDLMCDCPQ----GYQLKNNTCVKQ
```

3.4 The CR-cluster (residues 1075-1550)

3.4.1 Sequence details

More than half of all the ligands identified to bind the SORL1 receptor, including APP, interact with the CR-cluster [99-101]. In fact, SORL1 that lacks all its eleven CR-domains fails to bind APP [102], making it unlikely that APP binds to regions outside the CR-cluster.

CR-clusters of LDLR family members all contain 2 to 12 CR-domains (**Fig. 2**), while proteins that belong to the complement system include single CR-domains (i.e. C6, C7, C8α, C8β, C9, factor 1). This has led to two different definitions of the same module: "LDLR-type A repeats" and "Complement-type repeat (CR)-domains". One CR-domain sequence contains approximately 40 amino acids with several highly conserved residues, including six cysteines and five conserved acidic residues. The 6 cysteines (positions 15, 23, 29, 36, 42, 55) form three invariable disulfide bridges with the connectivity Cys^{I}-Cys^{III}, Cys^{II}-Cys^{V}, and Cys^{IV}-Cys^{VI} [103,104] (**Supplemental Figure S10)**, whereas the 5 conserved acidic residues were originally thought to engage in binding of ligands containing exposed basic residues in their receptor-binding site. In addition, a serine (position 46), together with a pair of hydrophobic residues at positions 21 (phenylalanine) and position 30 (isoleucine) in the more N-terminal part of the sequence are also highly conserved across CR-domains (**Supplemental Information 3.4.4**). Furthermore, the CR-domains in SORL1 also contain a pair of glycines (positions 27 and 38) that is conserved in eight of the eleven CR-domains (**Fig. 4**). With this high sequence conservation for 16 out of 40 positions across CR-domains from several proteins, it is not surprising that they all show a very similar folding (including domains from LDLR, ApoER2, LRP1, and LRP2) [105-113]. Structure determination by NMR indicates a very compact folding consisting of a β-hairpin structure with two short strands, followed by a series of β-turns (**Supplemental Figure S10**). The glycine at position 27 is at the center of the β-hairpin-turn, which requires a small side chain or none at all. The conserved phenylalanine (position 21) and isoleucine (position 30) pack against each other in a small hydrophobic core of the domain interior, preventing their side chains from engaging in ligand interactions (**Supplemental Figure S10**).

In members of the LDLR family, the combination of CR-domains plays a key role in ligand binding [114], outlining a functional consequence of exon skipping in some CR-clusters. This is best exemplified by the human ApoER2 gene (aka LRP8), which can encode a receptor with eight different CR-domains [115]. Most of the translated ApoER2 molecules *lack* three

central CR-domains CR4-CR6, producing a receptor that binds efficiently to Reelin but not α_2-macroglobulin. However, when CR4-CR6 is included this longer ApoER2 isoform can also bind α_2-macroglobulin [116]. Another ApoER2 isoform, with an unknown effect on ligand binding, lacks the most C-terminal CR-domain due to a second alternative splice event. Similarly, exon 4 skipping of human very low-density lipoprotein receptor *VLDLR* leads to a receptor isoform that lacks the third CR-domain, which increases VLDLR-affinity for ApoE-lipoproteins compared to VLDLR containing all its eight CR-domains [117]. Accordingly, alternative splicing of exons encoding CR-domains presents a mechanism to generate receptor variants with unique patho/physiological ligand binding properties.

A conserved Calcium cage - and the minimal motif

Ligand-binding to members of the LDLR family is critically dependent on calcium ions, which are coordinated by four of the conserved acidic residues in each CR-domain (at positions 37, 41, 47, and 48) (**Supplemental Figure S10a, red arrows in panel c**). Their acidic side chains form an octahedral calcium cage [107], which also stabilizes the folding of the C-terminal part of the domain [118]. The side chain of a fifth conserved acidic residue (aspartate at position 44) forms a structure known as an “Asx-turn”: it makes a hydrogen bond with the backbone amides of two residues: one residue upstream and the conserved serine two residues downstream (position 46) [119]. Two additional amino acids (positions 34 and 39) contribute to the calcium coordination with their backbone carbonyl groups (**Supplemental Figure S10a, and black arrows in panel c**). This geometry makes the side chains of residues at these two positions (most often a Trp-Asp pair in LDLR family proteins) ideally positioned at the domain’s molecular surface to engage in calcium-dependent ligand interactions [120-122]. These two amino acids, at positions 34 and 39, have therefore been named “CR-domain fingerprint residues” [55]. APP binding to SORL1 Is also strictly dependent on the presence of calcium [99].

The side chains of the two fingerprint residues interact most often with a lysine residue of the ligand (often positioned on a helical structure [123]) and a residue containing a hydrophobic side chain. It is intriguing how such a simple motif, called the “the minimal motif” [55,113,124], allows for discrimination of interaction partners [113,125-128]. The fingerprint of the SORL1 CR-cluster is strongly conserved among all the 11 CR-domains across evolution, highlighting the importance of the CR-cluster function (**Supplemental Information 3.8**). Substitutions at positions of fingerprint residues can impair binding of specific ligands, but do not affect overall folding and stability of CR-domains [120-122,129].

The CR-domain fingerprint residues of five of the eleven CR-modules in SORL1 (CR1, CR2, CR3, CR4, and CR8) are represented by the canonical Trp-Asp pair of amino acids common to the receptor:ligand complexes for the LDLR family (**Supplemental Figure S10a**). The

remaining six pairs of fingerprint residues have a hydrophobic residue instead of the aspartate, and three CR-domains (CR5, CR10 and CR11) have a charged residue (Glu or Lys) instead of the Trp, suggesting that the SORL1 CR-cluster may also bind ligands with motifs different from the common lysine-based motif for ligands of LDLR family members. The SORL1 ligand profile of these CR-domains may be more similar to SCO-spondin which contains 10 CR-domains, none of which has a Trp-Asp fingerprint pair [130]. Alternatively, the binding partner for these CR-domains may be another part of the SORL1 receptor, maybe depending on its overall folded conformation.

The necklace model and linker length

CR-domains fold independently from neighboring CR-domains, and substitutions that cause local misfolding of one CR-domain (e.g., substitutions of residues in the Calcium cage) still allow for correct folding of its adjacent CR-domain *in vitro* [131-133]. This agrees with the observed negligible interdomain interactions [53,134,135], suggesting that CR-clusters are like a necklace with the individual CR-domains behaving as “pearls-on-a-string” [134]. This modular organization allows for a high degree of flexibility that seems primarily determined by the length (and eventually the composition) of the interdomain sequence of amino acids; ie.e., the so-called linkers. This flexibility enables different CR-domains to wrap around larger ligands and engage in minimal motif interactions with multiple sites of the ligand. Such avidity, including two or more receptor CR-domains, leads to high-affinity ligand binding [120,136]. The linker sequences of CR-clusters are commonly 3 to 4 residues, with 12 amino acids as the longest connective string in LDLR. Many linker sequences in the SORL1 CR-cluster are longer, and the linkers towards the most C-terminal part of the cluster are extremely long. The sequences connecting CR8 with CR9 and CR9 with CR10 contain 15 and 17 amino acids, respectively, suggesting a unique flexibility of the SORL1 CR-cluster. However, as these linker-sequences are also highly conserved among species (**Supplemental Information 3.8**), we hypothesize that they fulfill an important role in the physiological function of SORL1. Interestingly, some of the SORL1 linkers become modified by *O*-linked glycans, including residues T1198 and T1508 [137,138], but how that relates to receptor activity has not yet been determined.

Andersen
Supplemental Figure S10 - CR-cluster

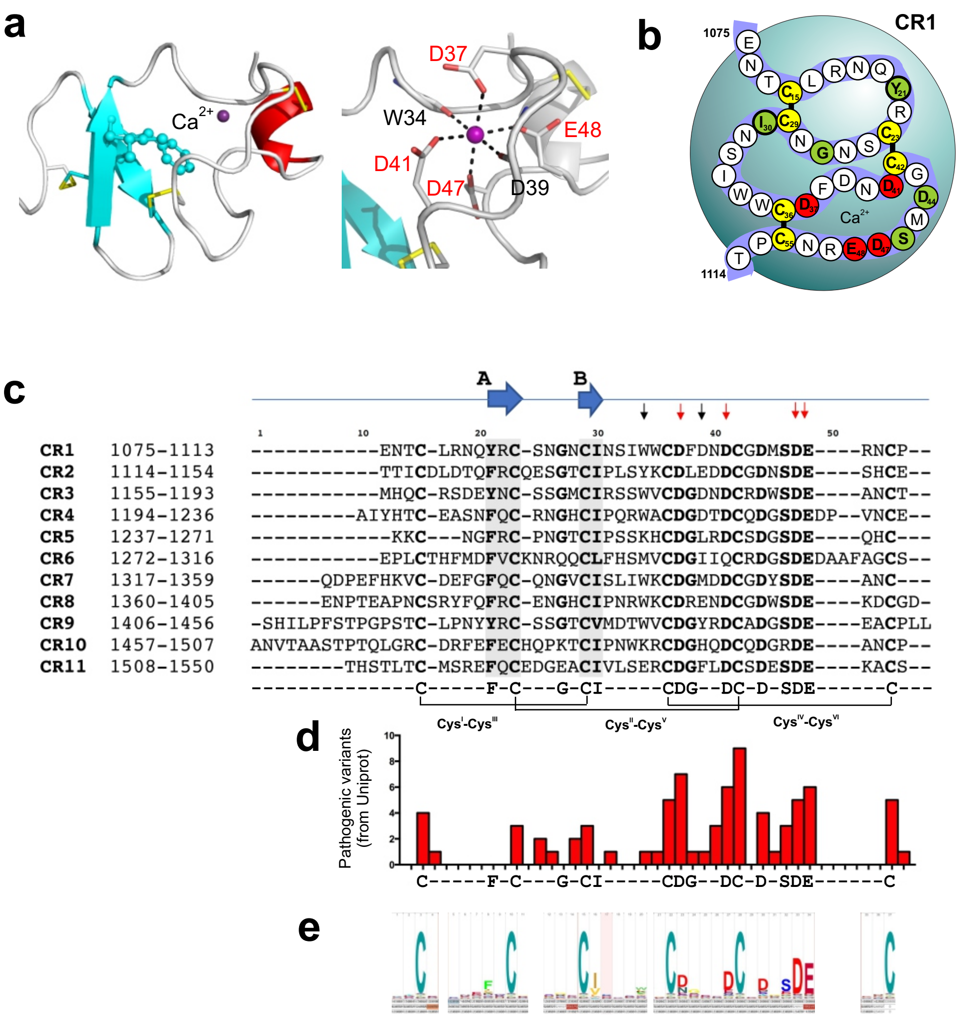

3.4.2 Supplemental Figure S10. CR-domains

a. The structure of CR7 from LRP1 (PDB: 1J8E) [110] with two short strands (light blue) and a short α-helix (pink). Close-up of the octahedral coordination of a Ca^{2+} ion (purple) by side chain carboxylates of D37, D41, D47, and E48 as well as backbone carbonyls from residues at position 34 and 39 (numbering according to sequence position in panel **c**).

b. Schematic of the residues 1075-1114 of CR1 of SORL1 with conserved residues on a colored background: cysteines (yellow), Ca^{2+} coordination via side chain carboxylates (red), and other highly conserved positions (green).

c. Alignments of the eleven CR-domains of SORL1 (residues 1075-1550). Conserved residues including six cysteines, two hydrophobic amino acids at positions 21 and 30, and 5 acidic residues and a Ser are all indicated below the alignment. Red and black arrows on top of the alignment indicate positions involved in calcium chelation via side chain or backbone carbonyls, respectively. Horizontal lines indicate the locations of the three invariable disulfides.

d. Histograms showing the number of pathogenic variants that occur for each CR-domain position as listed in **Supplemental Information 3.4.6**.

e. Logo representation of the domain sequence conservation: the larger the letter the higher the conservation across CR-domain sequences.

3.4.3 *SORL1* variants in CR-domains

Odd-numbered cysteines (ONC, positions 15, 23, 29, 36, 42, and 55 + all):

Either removal of one of the six conserved cysteines or introduction of a cysteine residue at another position of the domain sequence may disrupt the disulfide connectivity of CR-domains. There is strong evidence from our DMDM analysis that variants leading to an odd number (5 or 7) of cysteines (ONC) result in dysfunctional folding for other proteins with CR-clusters (**Supplemental Information 3.4.6**). Most prominently, we found that 21 out of 51 positions in LDLR linked to FH involve the replacement of a cysteine residue within the CR-cluster (**Supplemental Information 3.4.5**). Also the introduction of an extra cysteine, p.R78C^{LDLR} is considered pathogenic [83]. The DMDM analysis also identified disease associated variants that involve replacement of cysteines in other LDLR family members: p.C160Y^{LRP4} causal for CLSS [70] and p.C1361G^{LRP5} in patients with EVR4 [60,61]. Such ONC mutations are also pathogenic in proteins outside the LDLR family, e.g. the transmembrane proteinase TMPRSS6, (p.C510S^{TMPRSS6} [139,140] and p.C510R^{TMPRSS6} [139] in patients with IRIDA) or the COP9 protein (p.C119G^{C9} [141] in patients with C9D).

Two different ONC variants in *SORL1* has lately been reported to segregate with AD in small pedigrees that indicate that such mutations are truly pathogenic. First, a Swedish family (PED.25) with segregating variant p.R1303C in the sixth CR-domain was identified [97], and later a family in Saudi Arabia with variant p.R1084C in the first CR-domain was reported [142], both resulting in CR-domains with 7 cysteines. Also, a 59-year-old AD patient with variant p.C1192Y that results in having 5 cysteines in the third CR-domain has been identified [143], supporting that ONC variants in *SORL1* is associated with high risk for AD. The variant p.C1344R was reported associated with a possible family history in Finland [97], and also variants p.C1453S and p.C1249S were identified exclusively in AD patients [144].

Additional analysis of ONC variants in *SORL1* from the ADES-ADSP dataset [72], show how they occur predominantly in AD cases (p.R1080C, p.W1095C, pC1112Y, p.R1124C, p.R1172C, p.C1177Y, p.C1196CY, p.R1243C, p.R1260C, p.C1275S, p.C1286C, p.R1303C, p.Y1371C, p.Y1424C, p.Y1441C, p.C1453F, p.C1478S, p.R1490C, p.C1521R, p.C1540S; n=31) compared to controls (p.Y1196C, p.R1490C, p.C1497Y; n=5). Hence, ONC substitutions associate with a very strong increased risk of AD (OR = 6.31 95% CI: 2.45 - 16.24, p=5.1E-6; Fisher Exact test).

We note the similarity between ONC variants in *SORL1* associated with AD and variants in *NOTCH3* causal of Cerebral Autosomal Dominant Arteriopathy with Subcortical Infarcts and Leukoencephalopathy (CADASIL), where stereotypic causal variants also result in an odd-number of cysteines in EGF-domains of NOTCH3 carrying 32-34 copies of this domain type [145]. Or how 22 of 80 cysteines from the sequence of ten EGF-domains (of the eight-cysteine type) from the Usherin protein (encoded by *USH2A* are found mutated in patients with retinitis pigmentosa (*USH2A* LOVD mutation database, http://www.lovd.nl/USH2A). This demonstrates a general mechanism how variants in small cysteine-rich protein disulfide-containing domains that affects the number of cysteine residues may associate with a very high disease penetrance, and suggest that also ONC variants in *SORL1* should with high confidence be considered pathogenic and causal of AD.

Calcium cage (CaCa, positions 37,41, 47, and 48):

In proteins with CR-domains, residues at positions 37 and 41 and 47 are almost all Asp (in SORL1, there is a single exception; Gln[1301] at pos 41 in CR6) and positions 48 are all Glu (**Supplemental Figure S10c**). The side chains of these residues coordinate Ca^{2+} establishing an octahedral Ca^{2+} cage (CaCa) that is critical for domain folding [133,146]. As a consequence, substitutions of these variants may be strongly associated with disease.

Our DMDM analysis showed that in LDLR, substitutions of CaCa residues are associated with FH; **position 37**: p.D90G [83], p.D90N [83], p.D90Y [147], p.D168A [66], p.D168H [148], p.D168N [83,149], p.D168Y [150], p.D172N [62,149], p.D221G [58,66,83,84,151,152], p.D221N [74,84], p.D221Y [151,152]; **position 41**: p.D301G [66,149,153], p.D301A [154]; **position 47**: p.D139H, p.D227E [83,155], p.D266E [58]; and **position 48**: p.E101K [83,84], p.E140K [62,147,156]; p.E228K [66,157], p.E228Q [66]. Note the two disease-associated substitutions of Asp with Glu at position 47, which is generally considered a conservative – and often non-pathogenic – substitution. However, in CR-domains there is not enough space in the Calcium cage to accommodate the larger glutamate side chain at position 47 [107]. Uniprot also lists disease-associated variants for CaCa positions in other proteins: a variant in LRP2 at position 37 associates with intellectual disability (p.D3779N[LRP2]) [158] and another LRP2 variant at position 47 causes Stickler syndrome (p.D3828G[LRP2]) [159]. Uniprot further lists disease-associated variants in LRP5 (p.E1367K[LRP5] in patients with EVR [60,81]), TMRPSS3 (p.D103G[TMPRSS3] causal of deafness [160,161]) and TMRPSS6 (variants p.D521G[TMPRSS6] [139], p.D521N[TMPRSS6] [162,163] and p.E522K[TMPRSS6] [163] considered causative of IRIDA) (**Supplemental Information 3.4.5**).

There is also evidence that CaCa variants in *SORL1* is associated with AD. Studies report carriers of variant p.D1389V (position 37, CR8) only in AD patients, and these have very early

onset (<51 years) [164-166]. Other studies identified variant p.D1545E of CR11 (position 47; CADD score 15.9) [164], p.D1182N (position 41, CR3), and p.D1267E (position 47, CR5) [144] in AD patients only. Interestingly, the p.D1267E and p.D1545E variants have very low CADD scores (~16) presumably because a substitution of an Asp with a Glu is generally considered benign, only not when present in a CR-domain Calcium cage. We also recently found that the CaCa p.D1545V (position 47, CR11) variant is acting as a dominant negative and causal variant of AD in an Icelandic family [167].

The ADES-ADSP dataset includes such variants (p.D1108N, p.D1219G, p.D1261G, p.D1267N, p.D1345N, p.D1389V, p.D1502G, p.D1535N, p.D1545N, p.D1545G, p.D1545E) at positions in Calcium cages exclusively in AD cases (n=13) with a relatively early age at onset (median 60 years, ranging from 47-73 years) such that they are associated with a strong increased risk of AD (OR = INF) and in practice should be considered as causative for AD as loss-of-function variants that is now accepted as causal for AD [168].

Asx-turn: aspartate at position 44:

In SORL1 all eleven CR-domains contain an aspartate at position 44, which forms the Asx-turn [107]. Functional studies indicated that in LDLR, even the slightest modification of this residue results in FH, exemplified by the conservative aspartate to asparagine mutation in an LDLR CR-domain, because both carboxylate oxygens of the aspartate are necessary for hydrogen bonding [107]. The large alignment of CR-domain sequences confirms a strong preference for aspartate at position 44, and it may thus be considered a hotspot for pathogenic mutations (**Supplemental Information 3.4.6**). Uniprot lists four disease-associated variants at this position: p.D175N^{LDLR} (causal of FH in Afrikaners [155]), p.D175Y^{LDLR} (in FH patients [169]), p.D224V^{LDLR} (causal of elevated LDL cholesterol in FH patients [151]), and p.D137N^{LRP4} (variant is considerd causal of CLSS [70]).

In the ADES-ADSP dataset, we observed variants p.D1105H and p. D1146N at this position in *SORL1*, in respectively a 64- and a 48-year-old AD patient and none in controls [72]. Functional tests in cell culture studies showed strongly reduced shedding of D1105H mutant SORL1, and found low sSORL1 levels in CSF from two carriers of the variant, supporting these *SORL1* variants as pathogenic.

Hydrophobic "core": phenylalanine and Isoleucine at positions 21 and 30

These two positions in the CR-domain sequence are strongly conserved and stabilize the N-terminal part of CR-domains [105,106]. *In vitro* studies showed that mutation of residues at these two positions destabilized the CR-domain folding and impaired ligand-binding activity of LDLR

[170]. Surprisingly, our DMDM analysis of disease-associated variants in Uniprot did not find any pathogenic variants for neither of these two positions in any CR-domain containing proteins, including LDLR (**Supplemental Information 3.4.6**). This is surprising but suggests that despite being positions with strongly conserved amino acids, it may not be dangerous to substitute with other residues at these positions.

3.4.4 CR-domain alignment

Mapping of naturally occurring variants found in human CR-domain containing proteins, and being listed as associated with pathology at www.uniprot.org. The alignment follows the SORL1 alignment shown on top, and pathogenic variants are highlighted on a red background.

```
                    1        10             20             30             40             50
CR1   1075-1113     -----------ENTC-LRNQYRC-SNGNCINSIWWCDFDNDCGDMSDE----RNCP--
CR2   1114-1154     -----------TTICDLDTQFRCQESGTCIPLSYKCDLEDDCGDNSDE----SHCE--
CR3   1155-1193     -----------MHQC-RSDEYNC-SSGMCIRSSWVCDGDNDCRDWSDE----ANCT--
CR4   1194-1236     ---------AIYHTC-EASNFQC-RNGHCIPQRWACDGDTDCQDGSDE--DPVNCE--
CR5   1237-1271     ------------KKC---NGFRC-PNGTCIPSSKHCDGLRDCSDGSDE----QHC---
CR6   1272-1316     -----------EPLCTHFMDFVCKNRQQCLFHSMVCDGIIQCRDGSDEDAAFAGCS--
CR7   1317-1359     ------QDPEFHKVC-DEFGFQC-QNGVCISLIWKCDGMDDCGDYSDE----ANC---
CR8   1360-1405     ------ENPTEAPNCSRYFQFRC-ENGHCIPNRWKCDRENDCGDWSDE----KDCGD-
CR9   1406-1456     -SHILPFSTPGPSTC-LPNYYRC-SSGTCVMDTWVCDGYRDCADGSDE----EACPLL
CR10  1457-1507     ANVTAASTPTQLGRC-DRFEFECHQPKTCIPNWKRCDGHQDCQDGRDE----ANCP--
CR11  1508-1550     --------THSTLTC-MSREFQCEDGEACIVLSERCDGFLDCSDESDE----KACS–
                                  C     F C   g CI     CDG  DC D SDE      C

LDLR:
                              VGDRC-ERNEFQC-QDGKCISYKWVCDGSAECQDGSDE--SQETCLS
Δ=GG                            VTC-KSGDFSCΔRVNRCIPQFWRCDGQVDCDNGSDE----QGCP
                               PKTC-SQDEFRC-HDGKCISRQFVCDSDRDCLDGSDE----ASCPV
                                LTC-GPASFQC-NSSTCIPQLWACDNDPDCEDGSDE--WPQRCRG
                        LYVFQGDSSPC-SAFEFHC-LSGECIHSSWRCDGGPDCKDKSDE----ENCA
                               VATC-RPDEFQC-SDGNCIHGSRQCDREYDCKDMSDE----VGCV
                              NVTLCEGPNKFKC-HSGECITLDKVCNMARDCRDWSDE--PIKECG
TMPRSS6:
                                 PC--PGEFLCSVNGLCVPA---CDGVKDCPNGLDE----RNC
                                 VC--RATFQCKEDSTCISLPKVCDGQPDCLNGSDE----EQCQ

Δ=PQ                             PC-GTFTFQC-EDRSCVKKPNΔCDGRPDCRDGSDE----EHCD
COP9:

                                DDC--GNDFQC-STGRCIKMRLRCNGDNDCGDFSDE----DDCES
LRP4:

Δ=ALGEC                          AC-GRSHFTCAVSΔTCIPAQWQCDGDNDCGDHSDE----DGCI
                               LPTC-SPLDFHC-DNGKCIRRSWVCDGDNDCEDDSDE----QDCP
                               PREC-EEDEFPC-QNGYCIRSLWHCDGDNDCGDNSDE-----QCD
                               MRKC-SDKEFRC-SDGSCIAEHWYCDGDTDCKDGSDE----ENCP
                           SAVPAPPC-NLEEFQC-AYGRCILDIYHCDGDDDCGDWSDE----SDCS
                              SHQPC-RSGEFMC-DSGLCINAGWRCDGDADCDDQSDE----RNCT
                               TSMC-TAEQFRC-HSGRCVRLSWRCDGEDDCADNSDE----ENCE
                            NTGSPQC-ALDQFLC-WNGRCIGQRKLCNGVNDCGDNSDE-SPQQNCRPR
LRP5:
Δ=AT                             TC-SPDQFACΔGEIDCIPGAWRCDGFPECDDQSDE----EGCP
                                 VC-SAAQFPC-ARGQCVDLRLRCDGEADCQDRSDE----ADCD
                                AIC-LPNQFRC-ASGQCVLIKQQCDSFPDCIDGSDE----LMCEI
Corin:
                                LLCGRGENFLC-ASGICIPGKLQCNGYNDCDDWSDE----AHC
                                 NC-SENLFHC-HTGKCLNYSLVCDGYDDCGDLSDE----QNC
                                 DCNPTTEHRC-GDGRCIAMEWVCDGDHDCVDKSDE----VNC
                                 SCHSQGLVEC-RNGQCIPSTFQCDGDEDCKDGSDE----ENCSV
                                 EC-SPSHFKC-RSGQCVLASRRCDGQADCDDDSDE----ENC
                                 GCKERDLWECPSNKQCLKHTVICDGFPDCPDYMDE----KNC
                                SFC-QDDELEC-ANHACVSRDLWCDGEADCSDSSDE----WDCVT
TMPRSS3:
```

DC--SGKYRCRSSFKCIELIARCDGVSDCKDGEDE----YRCV

CFAI:

VCYTQKADSPMDDFFQC-VNGKYISQMKACDGINDCGDQSDE----LCCK

AC-QGKGFHC-KSGVCIPSQYQCNGEVDCITGEDE----VGCA

3.4.5 Identity of mapped CR variants: disease proteins and disease variants

Summary of included proteins containing naturally occurring variants associated with diseases in domains shared with SORL1.

Gene	Protein	Uniprot	#dom	Associated Diseases/Syndromes (CODE)	Variants mapped onto big alignments (from Uniprot entries except where a specific ref is provided)	#variants	#positions	Citations for variants discussed in main text
LDLR	Low-density lipoprotein receptor	P01130	7	Familial hypercholesterolemia (FHCL1)	**FHCL1**: **C27W**, **C46S**, A50S/A50T, S56P, **R78C**, W87G, **C89Y**, **D90G/D90N/D90Y**, Q92E, **C95G**, **E101K**, **C116R**, **C134F/C134W**, **E140K**, **C143R**, **C148Y**, **C155Y**, **C160Y**, **D168A/D168H/D168N/D168Y**, **D172N**, **C173W**, **D175N/D175Y**, S177L, **C184W/C184Y**, **C197R**, H211L, **D221G/D221N/D221Y**, **C222Y**, **D224V**, **D227E**, **E228K/E228Q**, **C231G**, **C243R**, **C248Y**, Q254P, **C261F**, **D266E**, **C276R/C276W/C276Y**, E277K, H285Y, S286R, E288K, R300G, **D301G**, **C302W/C302Y**, S306L, **C313R**, G314R	63	48	**C27W** [83]<br>**C46S** [171]<br>**C89Y** [83,84]<br>**C95G** [152]<br>**C116R** [149,152]<br>**C134F** [69]<br>**C134W** [69]<br>**C143R** [172]<br>**C148Y** [172]<br>**C155Y** [63,153]<br>**C160Y** [66,83]<br>**C173W** [173]<br>**C184W** [172]<br>**C184Y** [66,174]<br>**C197R** [154]<br>**C222Y** [69]<br>**C231G** [175]<br>**C243R** [62]<br>**C248Y** [154]<br>**C261F** [176]<br>**C276R** [69]

								**C276W** [66]<br>**C276Y** [66]<br>**C302W** [154]<br>**C302Y** [152]<br>**C313R** [83]<br>**D175N** causal in Afrikaners [155]<br>**D224V** causal of LDL cholesterol elevations [151]
LRP4	Low-density lipoprotein receptor-related protein 4	O75096	8	Cenani-Lenz syndactyly syndrome (CLSS)	**CLSS**: **D137N, C160Y**	2	2	**D137N** and **C160Y** considered causal of CLSS [70]
LRP5	Low-density lipoprotein receptor-related protein 5	O75197	3	Vitreoretinopathy, exudative 4 (EVR4)	**EVR4**: **C1361G, E1367K**	2	2	**C1361G** identified in patient with EVR4 [60,61]. Further evidence needed to establish if causal
TMPRSS6	Transmembrane protease serine 6	Q8IU80	3	Iron-refractory iron deficiency anemia (IRIDA)	**IRIDA**: **C510R/C510S, D521G/D521N, E522K**	5	3	**C501R** [139]<br>**C501S** [139,140]<br>**D521G** [139]<br>**D521N** [162,163]<br>**E522K** [163]
TMPRSS3	Transmembrane protease serine 3	P57727	1	Deafness, autosomal recessive, 8 (DFNB8)	**DFNB8**: **D103G**	1	1	**D103G** causal of deafness [160,161]
C9	Complement component 9	P02748	1	Complement component 9 deficiency (C9D)	**C9D**: **C119G**	1	1	**C119G** [141]
CORIN	Atrial natriuretic peptide-converting enzyme	Q9Y5Q5	7	Pre-eclampsia/eclampsia 5 (PEE5)	**PEE5**: K317E	1	1	
CFI	Complement factor I	P05156	2	CFI deficiency<br>Hemolytic uremic syndrome atypical 3 (AHUS3)	**CFI deficiency**: G243D<br>**AHUS3**: G287R	2	2	
						**77**	60	

3.4.6 CR disease variants listed according to domain positions

Disease-mutations domain-mapping analysis with identification of pathogenic variants in other proteins with CR-domains (as listed in **Supplemental Information 3.4.5**). Here variants are mapped onto domain positions following alignment of internally repeated sequences in the SORL1 domain sequences. The number of hits for each position depicted in the bar diagram of **Supplemental Figure S10d**.

**CR-domain positions**	Number of hits	Identified variants		
1				
2				
3				
4				
5				
6				
7				
8				
9				
10				
11				
12				
13				
14				
15; **C**	4	p.C27W(LDLR), p.C148Y(LDLR), p.C197Y(LDLR), p.C276Y(LDLR)	Loss of Cys	high
16	1	P:E277K(LDLR)		
17	0			
18	0			
19	0			
20	0			
21; **F**	0		Loss of Phe/Tyr?	moderate
22	0			
23; **C**	3	p.C155Y(LDLR), p.C116R(LDLR), p.C243R(LDLR)	Loss of Cys	high
24	0			
25	2	p.R78C(LDLR), p.H285Y(LDLR)		
26	1	p.S286R(LDLR)		
27; G	0			
28	2	p.E288K(LDLR), p.K317E(CORIN)		
29; **C**	3	p.C160Y(LDLR), pC248Y(LDLR), p.C160Y(LRP4)	Loss of Cys	high
30; **I**	0		Loss of Ile?	moderate

31	1	p.H211L(LDLR)		
32	0			
33	0			
34; fp	1	p.W87G/LDLR)		moderate
35	1	p.Q254P(LDLR)		
36; **C**	5	p.C46S(LDLR), p.C89Y(LDLR), p.C510R(TMPRSS6), p.C510S(TMPRSS6), p.C119G(C9)	Loss of Cys	high
37; **D**	7	p.D90G(LDLR), p.D90N(LDLR), p.D90Y(LDLR), p.D168A(LDLR), p.D168H(LDLR), p.D168N(LDLR), p.D168Y(LDLR)	Loss of Asp	high
38	1	p.G243D(CFI)		
39; fp	1	p.Q92E(LDLR)		moderate
40	3	p.A50S(LDLR), p.A50T(LDLR), p.R300G(LDLR)		
41; **D**	6	p.D172N(LDLR), p.D221G(LDLR), p.D221N(LDLR), p.D221Y(LDLR), p.D301A(LDLR), p.D301G(LDLR)	Loss of Asp	high
42; **C**	9	p.C95G(LDLR), p.C134F(LDLR), p.C134W(LDLR), p.C173W(LDLR), p.C222Y(LDLR), p.C261F(LDLR), p.C302W(LDLR), p.C302Y(LDLR), p.C1361G(LRP5)	Loss of Cys	high
43	0			
44; **D**	4	p.D175N(LDLR), p.D175Y(LDLR), p.D224V(LDLR), p.D137N(LRP4)	Loss of Asp	high
45	1	p.G287R(CFI)		
46; **S**	3	p.S56P(LDLR), p.S177L(LDLR), p.S306L(LDLR)	Loss of Ser	moderate
47; **D**	5	p.D227E(LDLR), p.D266E(LDLR), p.D521G(TMPRSS6), p.D521N(TMPRSS6), p.D103G(TMPRSS3)	Loss of Asp	high
48; **E**	6	p.E101K(LDLR), p.E140K(LDLR), p.E228K(LDLR), p.E228Q(LDLR), p.E522K(TMPRSS6), p.E1367K(LRP5)	Loss of Glu	high
49	0			
50	0			
51	0			
52	0			
53	0			
54	0			
55; **C**	5	p.C143R(LDLR), p.C184W(LDLR), p.C184Y(LDLR), p.C231G(LDLR), p.C313Y(LDLR)	Loss of Cys	high
56	1	p.G314R(LDLR)		
	**76**			

3.5 The 3Fn-cassette (residues 1551-2121)

3.5.1 Sequence details

Although the region containing the six 3Fn-domains corresponds to almost a third of the entire SORL1 extracellular part (the ectodomain), surprisingly little is known about its function. Many mutations observed in AD patients locate within the 3Fn-domains of SORL1, which suggests that this receptor region represents an important structural and/or functional aspect of SORL1 [144,177,178]. In other proteins, 3Fn-domains are commonly involved in ligand binding, but for the SORL1 protein, no interaction partner has yet been identified to bind to this region [101]. It can therefore be speculated that this region is important for the structural integrity of SORL1, and may be involved in bending of the full modular receptor, thereby arranging binding surfaces in space. Interaction "partners" may therefore rather be another SORL1 molecule to form SORL1-dimers [56], or other domains within the same SORL1 protein instead of foreign ligands.

3Fn-domains were originally identified in the modular protein Fibronectin, hence the name of the domain [179]. Fibronectin, which includes 15 copies of the 3Fn-domains, plays myriad fundamental biological roles such as adhesion, cell migration, and hemostasis, and similar multifunctionality is also true for many other proteins containing 3Fn-domains. This domain type is present in a high number of animal and bacterial protein families including extracellular matrix proteins, cell surface receptors, kinases and phosphatases, muscle proteins, etc. [180]. Accordingly, in contrast to the VPS10p-domain and the YWTD/EGF- and CR-domains that are representative of two distinct receptor families, a similar clear affiliation for 3Fn-domain containing proteins is not possible. More than 2,100 domains are listed in PFAM as being 3Fn-domains [180]. The structure of the second SORL1 3Fn-domain is deposited in the protein databank (2DM4.pdb), but not yet described in any publication.

Binding motif for SORL1 3Fn-domains with interacting partners not yet clear

3Fn-domains may occur as single repeats in proteins, but they are more frequently clustered as 2-6 adjacent domains [180]. In membrane anchored receptor proteins, including SORL1, the clustered 3Fn-domains are most often located directly proximal to the plasma membrane, where they may engage in contact with other proteins. While ligand interactions are characterized by RGD- or GSWGS-motifs in some 3Fn-domain-containing receptors [181-185], a unifying motif for ligand binding has not (yet) been identified for 3Fn-domains in general nor for the six SORL1 domains in particular.

Structure and important amino acids

A typical 3Fn-domain structure has an ellipsoid shape with approximate dimensions 38 Å x 20 Å x 25 Å [186]. The incoming and outgoing amino acid sequence ends at opposite sides of the folded domain (**Supplemental Figure S11**), which is in agreement with one of its main functions: to act as a spacer for proper positioning of protein structures [186]. This fold is topologically closely related to that of Immunoglobulin (IgG)-like domains: however, the 3Fn-domains lack the conserved disulfide bonds, and strands A and C' are interchanged between sheets relative to the IgG domains [186]. 3Fn-domains are typically composed of a sequence with 90-100 residues, arranged in seven β-strands (named A, B, C, C', E, F, and G) forming two anti-parallel β-sheets (strands: A-B-E and strands: C-C'-F-G, respectively) (**Supplemental Figure S11**). It is remarkable that despite high similarity in tertiary structure, sequence identity across 3Fn-domains is conspicuously low, typically less than 20% between domains in general [187], which complicates alignment of 3Fn-domain sequences. However, the presence of a few highly conserved amino acids enables unambiguous identification of strands B, C, and F (**Supplemental Figure S11**).

Strand B is characterized by a tryptophan (position 25) preceded by two hydrophobic residues at positions 21 and 23; **strand C** contains a tyrosine (position 41) followed by two hydrophobic residues at positions 43 and 45, with the latter position very often occupied by an additional tyrosine residue; **strand F** begins with a tyrosine (position 83) followed by three additional hydrophobic residues at positions 85, 87, and 89, with the latter position often being an alanine. As the hydrophobic residues alternate within a β-strand secondary structure, their side chains point towards the same side of their respective strand, such that they form a large hydrophobic domain-core – sometimes described as 'the glue' between the two β-blades (**Supplemental Figure S11**). The four remaining strands do not contain highly conserved residues, complicating their identification based on their primary structure. However, pairs of alternating hydrophobic residues are likely located in these β-strands as well, such that they can contribute to a hydrophobic core. This is often the case for strand A (positions 11 and 13) where the two hydrophobic amino acids are between 5 and 8 amino acids upstream of strand B, and either one or two prolines at the very beginning (positions 6 and 7) of the 3Fn-domain sequence (**Supplemental Information 3.5.4**). The other three strands (C', E, and G) have very little sequence conservation and can't be identified based on their amino acid sequence analysis, and their location is better identified needs by secondary structure prediction tools.

Both the first and the sixth 3Fn-domain of SORL1 include two cysteine residues, suggesting that these two domains contain an intradomain disulfide bond. This is supported by a model showing how their side chains, which we predict are in close proximity, can

facilitate intradomain disulfide bond formation in the folded conformation (**Fig. 1**). A single, likely unpaired, cysteine is located in strand B of the fourth 3Fn-domain, but predicted to point its side chain into a hydrophobic core and not be surface exposed.

Bottom Loops – including The Tyrosine Corner

Following the conserved folding topology, the loop regions between strands can be grouped as part of the "*top*" or "*bottom*" of a 3Fn-domain (**Supplemental Figure S11**). The BC-, C'E- and FG-loops combined with the N-terminal incoming sequence form the *top* of each 3Fn-domain, whereas the AB-, CC'- and EF-loops form the *bottom* of the domain. Some of these loops also contain conserved residues informative for sequence alignment. Most prominently, in the loop connecting strands E and F (**EF-loop**) that crosses from one sheet to the other, a leucine (position 77) is located six residues upstream of the tyrosine (position 83) in the beginning of strand F. In many 3Fn-domains, including two of the SORL1 domains, this loop also contains a conserved proline (position 79) frequently accompanied by a glycine (position 80) (**Supplemental Figure S11**). This structural motif is known as "***the tyrosine corner***", which contributes strongly to the stability of 3Fn-domains: the side chain of Leu-77 packs next to the Tyr-83 ring [188]. Moreover, the -OH group of the Tyr-83 engages in H-bonding with the backbone of the residue five residues upstream (i.e. position 78), naming the 3Fn-domain as the $\Delta 5$ subtype of tyrosine corners [189]. While all other loops can elongate without significant loss of conformational stability, the length of the EF-loop is critical to maintain a stable domain fold [190].

Top Loops – antigen binding homology and N-glycosylations

Among the loops at the top, a few conserved positions can be noticed: the **BC-loop** often begins with at least one proline at positions 27 and/or 28 as well as a glycine at position 36, and the **FG-loop** preferentially contains a glycine at position 94 – often together with another glycine at position 96 (**Supplemental Information 3.5.4**). This is not evident from the six SORL1 sequences due to the low conservation at these positions, but is clearer when looking at our alignment across multiple Fn3-domain sequences (**Supplemental Information 3.5.4**).

Within – or close to – each of the six **C'E-loops** of the SORL1 3Fn-domain is an NXT-motif, each of which represents a potential glycosylation acceptor site [191] (**Supplemental Figure S11d**). From our alignment of almost 300 3Fn-domain sequences, we noticed that an *N*-glycan acceptor site is frequently present in the C'E-loop. The function of these glycans probably varies for individual 3Fn-domains. A 3Fn-domain in the interleukin 21 receptor (IL21R) carries a large glycan at the C'E-loop, and this modification was first shown to be essential to keep the relative orientation between two neighboring domains in a fixed position, allowing interactions with residues from the adjacent 3Fn-domain [192]. Second, it was shown

that this glycan is essential for the successful transport of IL21R to the cell surface [193]. We recently found that the composition of *N*-glycans in SORL1 regulates the proteolytic cleavage by sheddase (TACE) of the membrane-bound SORL1 protein at the cell surface, and thus shedding of the soluble SORL1 ectodomain [194]. It is tempting to speculate that the regulatory glycan(s) locate in the proximity of the TACE cleavage site, i.e. somewhere in the 3Fn-domain region, and that the glycan(s) induce steric/conformational changes of SORL1, allowing the sheddase to access the cleavage site located just C-terminal to the sixth 3Fn-domain [194].

For IgG-domains, the *top* loops correspond to antigen binding regions (**Supplemental Figure S11**). Possibly, the top loops of the SORL1 3Fn-domains may bind extrinsic ligands. However, there are still many unclarities: many 3Fn-domains occur in tandem arrays of varying lengths, and the structure-function relationship of the entire region is highly dependent on the relative orientations between domain pairs, often measured as "*tilt*" and "*twist*" angles between domains [195] (see **Supplemental Information 3.11**). These measures are in part determined by the "linkers" between the domains but certainly also by the amino acid composition located in the loop regions.

Trp-ladders

Many 3Fn-domains share a motif that resembles a ladder with steps provided by alternating aromatic and charged amino acid side chains [196]. Since the aromatic "steps" are often tryptophan residues, this ladder is termed the "tryptophan-ladder". Generally, such ladders contain six or more "steps", but they may also be shorter. Upon inspection of the SORL1 3Fn-domain sequences, the presence of two short Trp-ladders was observed in the first and second domains most distal to the membrane, with steps made by (3Fn1: R1593, W1600, K1626, and H1636 and 3Fn2: E1690, W1698, R1722, and W1734). The function of these motifs is not completely understood. However, for some proteins it is suggested that they interact directly with sulphated glycoconjugates [197]. It has been demonstrated that some *N*-glycans in the VPS10p-domain of SORL1 are terminally sulphated [198], raising the hypothesis that they may be intrinsic targets of the Trp-ladders in 3Fn-region, possibly regulating SORL1-dimer formation and/or self-binding of bent-conformations of SORL1 (see **Supplemental Information 3.11**).

Andersen
Supplemental Figure S11-3Fn-domain

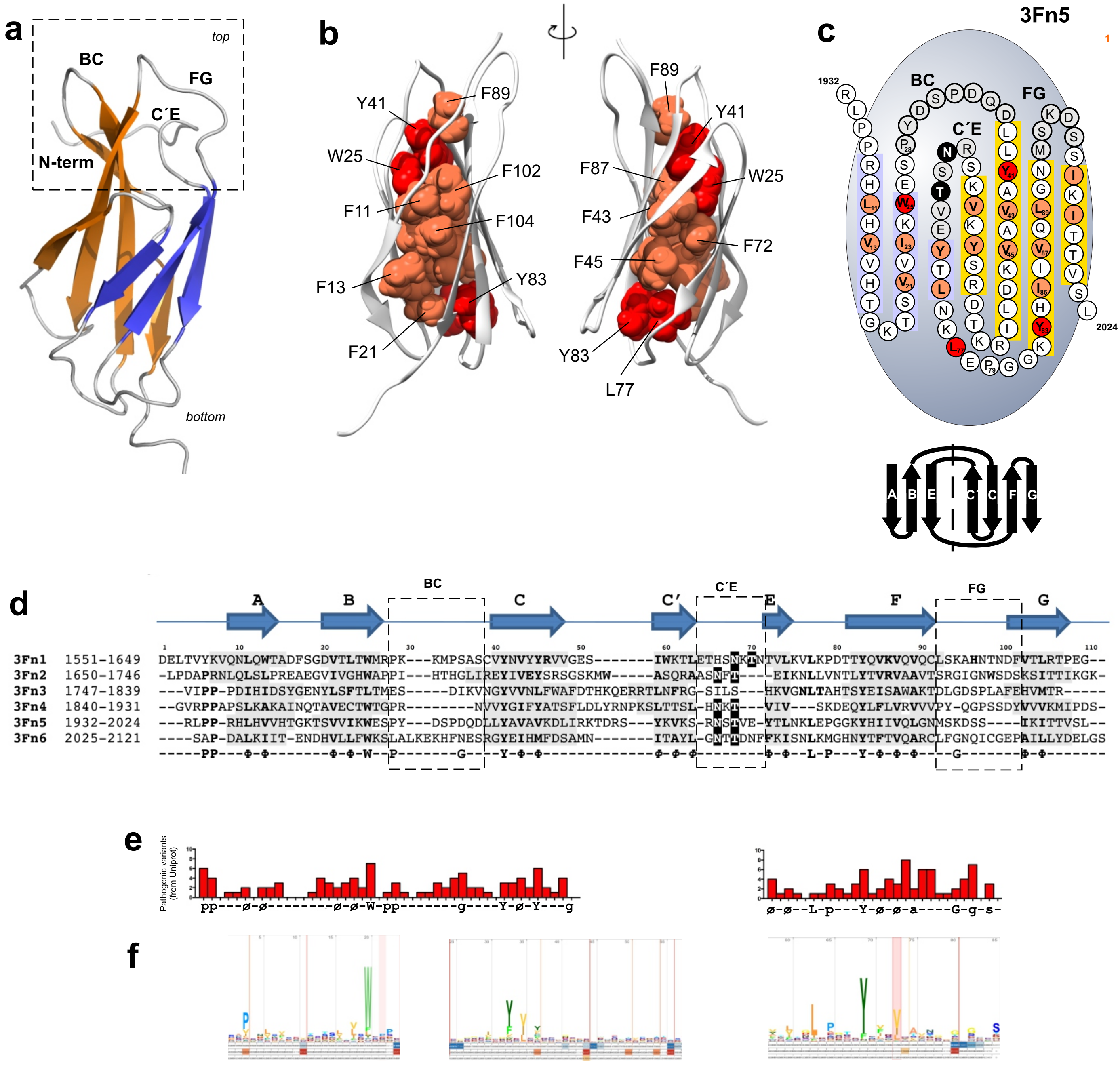

3.5.2 Supplemental Figure S11. 3Fn-domains

a. Structure of the second SORL1 3Fn-domain (PDB: 2DM4), showing the two-bladed sandwich conformation representative for 3Fn-domain structures in general. The BC-, C'E- and FG-loops together with the N-terminal residues (boxed) correspond to the antigen binding part of the similarly folded IgG-domains.

b. Structure of the 3Fn-domain showing how the alternating hydrophobic residues contribute to a compact core of the domain (orange colored side chains – identified in *panel c*), forming hydrophobic interactions between their side chains that keep the two sheets tightly together. The side chain of the four conserved residues at positions 25 (Trp), 41 (Tyr), 77 (Leu), and 83 (Tyr) are colored red. The two structures represent a 180° turn seeing into the sandwich from opposite sites.

c. Residues 1932-2024 of the fifth 3Fn-domain presented according to the characteristic anti-parallel strand topology A-B-E-C'-C-F-G. The two β-sheets are indicated by purple and yellow background for their strands, respectively. The alternating residues that contribute to the hydrophobic domain interior are indicated by orange circles, amino acids that are part of the BC-, C'E-, and FG-loops are on grey background, and the four most conserved residues W^{25}, Y^{41}, L^{77}, and Y^{83} are presented on red circles. Sheet topology is indicated below.

d. The SORL1 sequence of residues 1551-2121 represents six 3Fn-domains. Alignment of the 3Fn-domain sequences was done according to the β-strand secondary structure diagram shown in panel **c**. The limited number of positions with conserved amino acids and the identified alternating hydrophobic residues (Φ) are presented below the alignment. Residues in top loop regions are included in the broken line boxes. The alignment was specifically designed to allow the conserved consensus motif for *N*-glycosylation (NXT, black background) to be located in the C'E-loop when possible.

e. Histograms showing the number of pathogenic variants that occur for each 3Fn-domain position as listed in **Supplemental Information 3.5.6**.

f. Logo representation of the domain sequence conservation: the larger the letter the higher the conservation across 3Fn-domain sequences.

3.5.3 *SORL1* variants in 3Fn-domains

Trp at position 25 (strand B):

The 3Fn-domain is vulnerable for mutation of the Trp at position 25, for which Uniprot lists seven disease-associated variants: p.W2744C^{USH2A} (segregate with USH2A in a family [199]), p.W3521R^{USH2A} (in patients with USH2A [200]), p.W1036L^{L1CAM} (in patients with HSAS, and functional test of the mutant protein show defective cellular transport [201]), p.W1925R^{FN1} (causal of GFND2 [202]), p.W571R^{ANOS1} (in patients with Kallmann Syndrom/HH1 [203]), p.W792R^{MYBPC3} (in patients with CM44 [204]), and p.W68R^{GHR} (in patients with LARS [205]). In line with the the orthologue data, a report described identification of an AD patient carrying p.W1862C (position 25 in the fourth 3Fn-domain) [206], which suggests that variants at this position in SORL1 domains may associate with an increased risk for AD.

Tyr at position 83 (Tyr-corner):

The tyrosine at position 83 at the beginning of strand F is conserved across all 3Fn-domains including those in SORL1 (**Fig. 4**). Our DMDM analysis found that five of the six other proteins for which Uniprot lists disease-associated substitutions for this position, each time the Tyr was replaced by a Cys: in Tie2, p.Y611C^{TEK} (reduced response to ligand and decreased ligand-induced phosphorylation GLC3E [207]), in the insulin receptor, p.Y818C^{IR} (abolishes post-translational processing, LEPRC [208,209]), in Fibronectin causal for GFND2 p.Y973C^{FN1} (GFND2 [202]), in L1CAM, p.Y784C^{L1CAM} (HSAS [210]) and in the growth hormone receptor, p.Y226C^{GHR} (causal of LARS [211]) (**Supplemental Information 3.5.5**).

It has previously been reported that variant p.Y1816C that locate to the third 3Fn-domain of SORL1 was found in AD patients and but not controls [144]. Interestingly, also in the case of SORL1, the tyrosine is substituted by a cysteine, such that a possible pathogenic effect may either be due to loss of the tyrosine or due to introduction of the cysteine, or both. We note that all the other proteins that harbor this type of variant form active dimers by their 3Fn-region, which suggests that such mutations, including p.Y1816C in SORL1, may act via impaired dimerization.

Position 88 (strand F):

For position 88, located between the alternating hydrophobic residues in strand F, there is no sequence conservation (**Supplemental Figure S11d, Supplemental Information 3.5.4**). However, the side chain of the residue at this position will expose towards the domain surface. Intriguingly, Uniprot lists as many as 9 disease-associated variants at this position

(**Supplemental Information 3.5.5**), of which 8 correspond to arginine replacements: p.R926W^{IR} (mutated IR with markedly impaired insulin binding and impaired post-translational processing in patients with LEPRCH [209,212]), p.R312P^{CRLF1} (CISS1 [213]), p.R224W^{IL2RG} (XSCID [214]), p.R201L^{IL21R} (mutant receptor with defective trafficking, misfolding, and impaired processing IMD56 [192,193]), p.R213W^{IL12RB1} (IMD30 [215]), p.R114C^{IFNGR2} (misfolding and abnormal glycosylation, mistrafficking, reduced response to INFG, IMD28 [216,217]), p.R257L^{MPL} (CAMT [218]), and p.R308C^{CSF3R} (decreased localization to plasma membrane and decreased receptor signaling, SCN7 [219]). Detailed analysis of the variant in IL21R revealed how this arginine side chain is required for interaction with a glycan from a neighboring 3Fn-domain and the relative domain-domain orientation [192]. The SORL1 sequence includes only an arginine at position 88 in the fourth 3Fn-domain, and no variants is yet identified for this residue.

Positions 94 and 96 (glycines in FG-loop):

From Uniprot we identified seven disease-associated variants at position 96 of the 3Fn-domain according to our DMDM analysis (**Supplemental Information 3.5.5**). There is a slight preference for Gly at this position according to the 3Fn-domain consensus sequence, but it is not nearly as conserved as Gly at position 94. (**Supplemental Information 3.5.4**). However, we noticed that in five of the seven identified disease variants for position 96, a Gly was substituted. Furthermore, four of these variants were observed when the Gly two residues upstream (at position 94) in the sequence was also present: p.G698R^{L1CAM} (causal for hydrocephalus HSAS/MASA and mutation is inherited in a mendelian fashion segregating with disease [220,221]), p.G805E^{DCC} (causal of isolated agenesis of the corpus callosum (MRMV1) in a family, and mutation disturb nestrin-1 binding to the FG-loop of the DCC 3Fn-domain [222]), p.G2757V^{SPEG} (causal of Centronuclear Myopathy with Dilated Cardiomyopathy (CNM5) in a small family [223]), and p.G516R^{EPHB4} (mutation segregate with the Capillary Malformation-Arteriovenous Malformation (CMAVM2)-phenotype in three small families [224]). We speculate that co-occurrence of both Gly residues could have functional relevance relating to their localization in the FG-loop region preferring to accommodate residues with small side chains. In SORL1, only the second 3Fn-domain contains this double Gly at positions 94 and 96 (amino acids 1730 and 1732) (**Fig. 4**). Very interestingly variant p.G1732A corresponding to position 96 is reported to segregate with AD in a Swedish family [97], in support of this variant being pathogenic.

3.5.4 3Fn-domain alignment

Mapping of naturally occurring variants found in human 3Fn-domain containing proteins, and being listed as associated with pathology at www.uniprot.org. The alignment follows the SORL1 alignment shown on top, and pathogenic variants are highlighted on a red background.

SORL1:

```
    DELTVYKVQNLQWTA-DFSGDVTLTWMRPK---KMPSASCVYNVYYRVVGES-------IWKTLETHSNKTN-----------------TVLKVLKPDTTYQVKVQVQCLSKAHNTNDFVTLRTPEG--
    -LPDAPRNLQLSLPR-EAEGVIVGHWAPPI---HTHGLIREYIVEYSRSGSKMW-----ASQRAASNFT--------------------EIKNLLVNTLYTVRVAAVTSRGIGNWSDSKSITTIKGK-
    ---VIPP-PDIHIDS-YGENYLSFTLTMES-----DIKVNGYVVNLFWAFDTHKQERRTLNFRG-SILS--------------------HKVGNLTAHTSYEISAWAKTDLGDSPLAFEHVMTR-----
    --GVRPPAPSLKAKA-INQTAVECTWTGPR--------NVVYGIFYATSFLDLYRNPKSLTTSL-HNKT--------------------VIV---SKDEQYLFLVRVVVPY-QGPSSDYVVVKMIPDS-
    ---RLPP-RHLHVVH-TGKTSVVIKWESPY---DSPDQDLLYAVAVKDLIRKTDRS---YKVKS-RNSTVE------------------YTLNKLEPGGKYHIIVQLGNMSKDSS----IKITTVSL-
    ----SAP-DALKIIT--ENDHVLLFWKSLALKEKHFNESRGYEIHMFDSAMN-------ITAYL-GNTTDNF-----------------FKISNLKMGHNYTFTVQARCLFGNQICGEPAILLYDELGS
         pp    Φ Φ          Φ Φ W pp       g     Y Φ Φ    g          Φ Φ Φ                    Φ Φ  L p   Y Φ Φ a    g        Φ Φ
                            sø ø W PP       g ø   Y ø ø                                           ø gL p t Y ø ø a    G g s
```

Usherin:

```
    PFQQPPP--RGQVQS---SSAINLSWSPPD---SPNAHWLTYSLLRDGFEIYTTEDQYPYSIQY-------------------------FLDTDLLPYTKYSYYIETTNVHGSTRSVAVTYKTKP
 GVPEGNLTLS--YIIPIG---SDSVTLTWTTLS---NQSGPIEKYILSCAPLAGGQPCVSYEGHETS-------------------------ATIWNLVPFAKYDFSVQACTSGGCLHSLPITVTTAQAPPQ
     RLSPP--KMQKIS---STELHVEWSPPA---ELNGIIIRYELYMRRLRSTKETTSEESRVFQSSGWLSPHSFVESANENALKPPQTMTTITGLEPYTKYEFRVLAVNMAGSVSSAWVSERTGESAPVF
      MIPP-SVFPLSS----YSLNISWEKPA-DNVTRGKVVGYDINMLSEQSPQQSIPMAFSQLLHTAKSQELS----------------YTVEGLKPYRIYEFTITLCNSVGCVTSASGAGQTLAAAPA
      RGAVVNLASVSSGAVRVNLDGCLSTDS-AVNCRGNDSILVYQGKEQS---------------------------------------VYEGGLQPFTEYLYRVIASHEGGSVYSDWSRGRTTGAAPQ
    SVPTPS--RVRSLN---GYSIEVTWDEPV----VRGVIEKYILKAYSEDSTRPPRMPSASAEFVNTSNLT------------------GILTGLLPFKNYAVTLTACTLAGCTESSHALNISTPQE
   APQEVQPP--VAKSL----PSSLLLSWNPPK---KANGIITQYCLYMDGRLIYSGSEEN-------------------------------YIVTDLAVFTPHQFLLSACTHVGCTNSSWVLLYTAQLPPE
     HVDSP--VLTVLD---SRTIHIQWKQPR---KISGILERYVLYMSNHTHDFTIWSVIYNSTELFQD---------------------HMLQYVLPGNKYLIKLGACTGGGCTVSEASEALTDED
   IPEGVPAP--KAHSYS---PDSFNVSWTEPE---YPNGVITSYGLYLDGILIHNSSELSYRAYGFAPWSLHSFRVQACT-----------AKGCALGPLVENRTLEAPPE
       GTVNVFVKTQG---SRKAHVRWEAPF---RPNGLLTHSVLFTGIFYVDPVGNNYTLLNVTKVMYSGEETNLW-------------VLIDGLVPFTNYTVQVNISNSQGSLITDPITIAMPPG
   APDGVLPP--RLSSAT---PTSLQVVWSTPA-RNNAPG-SPRYQLQMRSGDSTHGFLELFSNPSASLS----------------------YEVSDLQPYTEYMFRLVASNGFGSAHSSWIPFMTAED
   KPGPVVPP--ILLDVK---SRMMLVTWQHPR---KSNGVITHYNIYLHGRLYLRTPGNVTN----------------------------CTVMHLHPYTAYKFQVEACTSKGCSLSPESQTVWTLPG
```

```
APEGIPSP--ELFSDT---PTSVIISWQPPT---HPNGLVENFTIERRVKGKEEVTTLVTLPRSHSMRFI--------------------DKTSALSPWTKYEYRVLMSTLHGGTNSSAWVEVTTRPS
RPAGVQPP--VVTVLE---PDAVQVTWKPPL---IQNGDILSYEIHMPDPHITLTNVTSAVLS---------------------------QKVTHLIPFTNYSVTIVACSGGNGYLGGCTESLPTYVTTHPT
 VPQNVGP-LSVIPLS---ESYVVISWQPPS---KPNGPNLRYELLRRKIQQPLASNPPEDLNRWHNIYSGTQWL---------------YEDKGLSRFTTYEYMLFVHNSVGFTPSREVTVTTLAG
LPERGANL--TASVLN---HTAIDVRWAKPT-VQDLQGEVEYYTLFWSSATSNDSLKILPDVNS--------------------------HVIGHLKPNTEYWIFISVFNGVHSINSAGLHATTCDG
EPQGMLPP--EVVIIN---STAVRVIWTSPS---NPNGVVTEYSIYVNNKLYKTGMNVPGS-----------------------------FILRDLSPFTIYDIQVEVCTIYACVKSNGTQITTVEDTPSD
    IPTP--TIRGIT---SRSLQIDWVSPR---KPNGIILGYDLLWKTWYPCAKTQKLVQDQSDELCKAVRC-----------------QKPESICGHICYSSEAKVCCNGVLYNPKPGHRCCEE
   CPASMEATEHCGRCDFNFTSHICTVIRG-SHNSTGKASIEEMCSSAEETIHTGSVNTYS----------------------------YIDVNLKPYMTYEYRISAWNSYGRGLSKAVRARTKED
VPQGVSPP--TWTKID-NLEDTIVLNWRKPI---QSNGPIIYYILLRNGIERFRGTSLSF------------------------------SDKEGIQPFQEYSYQLKACTVAGCATSSKVVAATTQG
VPESILPP--SITALS---AVALHLSWSVPE---KSNGVIKEYQIRQVGKGLIHTDTTDRRQ----------------------------HTVTGLQPYTNYSFTLTACTSAGCTSSEPFLGQTLQAAPE
  GVWVTP--RHIIIN---STTVELYWSLPE---KPNGLVSQYQLSRNGNLLFLGGSEEQN-----------------------------FTDKNLEPNSRYTYKLEVKTGGGSSASDDYIVQTPMS
TPEEIYPP-YNITVIG---PYSIFVAWIPPG--ILIPEIPVEYNVLLNDGSVTPLAFSVGHHQS--------------------------TLLENLTPFTQYEIRIQACQNGSCGVSSRMFVKTPEA
APMDLNSP--VLKALG---SACIEIKWMPPE---KPNGIIINYFIYRRPAGIEEESVLFVWSEGALEFM---------------------DEGDTLRPFTLYEYRVRACNSKGSVESLWSLTQTLEA
PPQDFPAP--WAQATS---AHSVLLNWTKPE---SPNGIISHYRVVYQERPDDPTFNSPTVHAFTVKGTSHQ------------------AHLYGLEPFTTYRIGVVAANHAGEILSPWTLIQTLES
    SPSGLRNFIVEQKENGRALLLQWSEPM---RTNGVIKTYNIFSDGFLEYSGLNRQ-------------------------------FLFRRLDPFTLYTLTLEACTRAGCAHSAPQPLWTDEA
PPDSQLAP--TVHSVK---STSVELSWSEPV---NPNGKIIRYEVIRRCFEGKAWGNQTIQADEKIVFTEYNTERNTFM-----------YNDTGLQPWTQCEYKIYTWNSAGHTCSSWNVVRTLQA
PPEGLSPP--VISYVS-MNPQKLLISWIPPE---QSNGIIQSYRLQRNEMLYPFSFDPVTFN----------------------------YTDEELLPFSTYSYALQACTSGGCSTSKPTSITTLEAAPSE
    VSPP--DLWAVS---ATQMNVCWSPPT---VQNGKITKYLVRYDNKESLAGQGLC-------------------------------LLVSHLQPYSQYNFSLVACTNGGCTASVSKSAWTMEALPE
   NMDSP--TLQVTG---SESIEITWKPPR---NPNGQIRSYELRRDGTIVYTGLETR-------------------------------YRDFTLTPGVEYSYTVTASNSQGGILSPLVKDRTSPS
APSGMEPP--KLQARG---PQEILVNWDPPV---RTNGDIINYTLFIRELFERETKIIHINTTHNSFGMQS-------------------YIVNQLKPFHRYEIRIQACTTLGCASSDWTFIQTPEIAPLM
    QPPPHLEVQMAPGGFQPTVSLLWTGPL---QPNGKVLYYELYRRQIATQPRKSNPVLIYNCSSTS---------------------FIDSELLPFTEYEYQVWAVNSAGKAPSSWTWCRTGPA
PPEGLRAP--TFHVIS---STQAVVNISAPG---KPNGIVSLYRLFSSSAHGAETVLSEGMATQ--------------------------QTLHGLQAFTNYSIGVEACTCFNCCSKGPTAELRTHPA
PPSGLSSP--QIGTLA---SRLASFRWSPPM---FPNGVIHSYELQFHVACPPDSALPCTPSQIETKYTGLGQK----------------ASLGGLQPYTTYKLRVVAHNEVGSTASEWISFTTQKELP
```

L1CAM:

```
  SPGPVPRLVLSDLHLLTQSQVRVSWSPA---EDHNAPIEKYDIEFEDKEMAPEKWYSLGKVPGNQT----------------------STTLKLSPYVHYTFRVTAINKYGPGEPSPVSETVVTPEA
  APEKNP-VDVKGEG-NETTNMVITWKPL-RWMDWNAPQVQYRVQWRPQGTRGPWQEQIVSDPF-------------------------LVVSNTSTFVPYEIKVQAVNSQGKGPEPQVTIGYSGED
  YPQAIP-ELEGIEI-LNSSAVLVKWRPV-DLAQVKGHLRGYNVTYWREGSQRKHSKRHIHKDHVVVPANTTS----------------VILSGLRPYSSYHLEVQAFNGRGSGPASEFTFSTPEG
   VPGHP-EALHLEC-QSNTSLLLRWQPP---LSHNGVLTGYVLSYHPLDEGGKGQLSFNLRDPELRT---------------------HNLTDLSPHLRYRFQLQATTKEGPGEAIVREGGTMALS
    GISDFGNISATA--GENYSVVSWVPK-----EGQCNFRFHILFKALGEEKGGASLSPQYVSYNQSS--------------------YTQWDLQPDTDYEIHLFKERMFRHQMAVKTNGTGRVRLPPA
```

INSR:

```
    CENELLKFSYIR-TSFDKILLRWEPYW--PPDFRDLLGFMLFYKEAPYQNVTEFDGQDACGSNSWTVVDIDPPLRSNDPKSQNHPGWLMRGLKPWTQYAIFVKTLVTFSDERRTYGAKSDIIYVQTDA
```

```
            NPSVPLDPISVSN--SSSQIILKWKPPS---DPNGNITHYLVFWERQAEDSELFELDYCLΛNKES---------------------
LVISGLRHFTGYRIELQACNQDTPEERCSVAAYVSARTMPEAKADD
             IVGPVTHEIFEN----NVVHLMWQEPK---EPNGLIVLYEVSYRRYGDEELHLCVSRKHFALERG--------------------CRLRGLSPG-NYSVRIRATSLAGNGSWTEPTYFYVTDY
```

IGF1R:

```
           PERKRRDVMQVANTTMSSRSRNTTAADTYNITDPEELETEYPFFESRVDNKER----------------------------------TVISNLRPFTLYRIDIHSCNHEAEKLGCSASNFVFARTMPA
               IP-GPVTWEP-RPENSIFLKWPEPE---NPNGLILMYEIKYGSQVEDQRECVSRQEYRKYGG---------------------AKLNRLNPG-NYTARIQATSLSGNGSWTDPVFFYVQAK
```

Fibronectin:

```
               GPVEVFITETPSQPNSHPIQWNAPQ-----PSHISKYILRWRPKNSVGRWKEATIPGHLNS----------------------YTIKGLKPGVVYEGQLISIQQYGHQEVTRFDFTTTST
                TSESVTEIT---ASSFVVSWVSAS------DTVSGFRVEYELSEEGDEPQYLDLPSTATS----------------------VNIPDLLPGRKYIVNVYQISEDGEQSLILSTSQTTAPD
              APP--DTTVDQ-VDDTSIVVRWSRPQ------APITGYRIVYSPSVEGSSTELNLPETANS-----------------------VTLSDLQPGVQYNITIYAVEENQESTPVVIQQETTGTPRSDT
             VPSP-RDLQFVE-VTDVKVTIMWTPPE------SAVTGYRVDVIPVNLPGEHGQRLPISRNTF----------------------AEVTGLSPGVTYYFKVFAVSHGRESKPLTAQQTTKLD
               AP-TNLQFVN-ETDSTVLVRWTPPR------AQITGYRLTVGLTRRGQPRQYNVGPSVSK-----------------------YPLRNLQPASEYTVSLVAIKGNQESPKATGVFTTLQPG
            SSIPP-YNTEVTE----TTIVITWTPAP--------RIGFKLGVRPSQGGEAPREVTSDSGS------------------------IVVSGLTPGVEYVYTIQVLRDGQERDAPIVNKVVTPLS
               PP-TNLHLEANPDTGVLTVSWERST-----TPDITGYRITTTPTNGQQGNSLEEVVHADQSS---------------------CTFDNLSPGLEYNVSVYTVKDDKESVPISDTIIPEVPQL
                  TDLSFVD-ITDSSIGLRWTPLN-----SSTIIGYRITVVAAGEGIPIFEDFVDSSVGY----------------------YTVTGLEPGIDYDISVITLINGGESAPTTLTQQTAV
              PPP-TDLRFTN-IGPDTMRVTWAPPP-----SIDLTNFLVRYSPVKNEEDVAELSISPSDNA----------------------VVLTNLLPGTEYVVSVSSVYEQHESTPLRGRQKTGLD
               SP-TGIDFSD-ITANSFTVHWIAPR------ATITGYRIRHHPEHFSGRPREDRVPHSRNS----------------------ITLTNLTPGTEYVVSIVALNGREESPLLIGQQSTVSD
               VP-RDLEVVA-ATPTSLLISWDAPA------VTVRYYRITYGETGGNSPVQEFTVPGSKST----------------------ATISGLKPGVDYTITVYAVTGRGDSPASSKPISINYRTEID
               KP-SQMQVTD-VQDNSISVKWLPSS------SPVTGYRVTTTPKNGPGPTKTKTAGPDQTE----------------------MTIEGLQPTVEYVVSVYAQNPSGESQPLVQTAVTNID
               RP-KGLAFTD-VDVDSIKIAWESPQ------GQVSRYRVTYSSPEDGIHELFPAPDGEEDT----------------------AELQGLRPGSEYTVSVVALHDDMESQPLIGTQSTAIP
               AP-TDLKFTQ-VTPTSLSAQWTPPN------VQLTGYRVRVTPKEKTGPMKEINLAPDSSS----------------------VVVSGLMVATKYEVSVYALKDTLTSRPAQGVVTTLENVS
               PP-RRARVTD-ATETTITISWRTKT------ETITGFQVDAVPANGQTPIQRTIKPDVRS-----------------------YTITGLQPGTDYKIYLYTLNDNARSSPVVIDASTAID
               AP-SNLRFLA-TTPNSLLVSWQPPR------ARITGYIIKYEKPGSPPREVVPRPRPGVTE----------------------ATITGLEPGTEYTIYVIALKNNQKSEPLIGRKKTDELP
           GPGLNP--NASTGQ-EALSQTTISWAPFQ-------DTSEYIISCHPVGTDEEPLQFRVPGTSTS----------------------ATLTGLTRGATYNVIVEALKDQQRHKVREEVVTVGNSVNEG
```

DCC:

```
               APRDVVPVLV--SSRFVRLSWRPPA---EAKGNIQTFTVFFSREGDNRERALNTTQPGSLQ----------------------LTVGNLKPEAMYTFRVVAYNEWGPGESSQPIKVATQPE
           LQVPGPVENLQAVS-TSPTSILITWEPPA---YANGPVQGYRLFCTEVSTGKEQNIEVDGLS-------------------------YKLEGLKKFTEYSLRFLAYNRYGPGVSTDDITVVTLSD
           VPSAPP-QNVSLEV-VNSRSIKVSWLPPP-----SGTQNGFITGYKIRHRKTTRRGEMETLEPNNLW--------------------YLFTGLEKGSQYSFQVSAMTVNGTGPPSNWYTAETPEN
       DLDESQVPDQP-SSLHVRP--QTNCIIMSWTPPL---NPNIVVRGYIIGYGVGSPYAETVRVDSKQRY------------------------YSIERLESSSHYVISLKAFNNAGEGVPLYESATTRSITDPT
    FPTSVPDLSTPMLPP-VGVQAVA-LTHDAVRVSWADNSVPKNQKTSEVRLYTVRWRTSFSASAKYKSEDTTSLS----------------------YTATGLKPNTMYEFSVMVTKNRRSSTWSMTAHATTYEA
           APTSAPKDLTVITREGKPRAVIVSWQPPL---EANGKITAYILFYTLDKNIPIDDWIMETISGDRLT--------------------HQIMDLNLDTMYYFRIQARNSKGVGPLSDPILFRTLKV
```

Leptin receptor:

PP-LGLHMEI-TDDGNLKISWSSPP----LVPFPLQYQVKYSENSTTVIREADKIVSATS------------------------LLVDSILPGSSYEVQVRGKRLDGPGIWSDWSTPRVFTTQDV

PP-SSVKAEITINIGLLKISWEKPV----FPENNLQFQIRYGLSGKEVQWKMYEVYDAKSKS----------------------VSLPVPDLCAVYAVQVRCKRLDGLGYWSNWSNPAYTVVM

DIKVPMRGPEFWRIINGDTMKKEKNVTLLWKPLM-KNDSLCSVQRYVINHHTSCNGTWSEDVGNH----------------------------TKFTFLWTEQAHTVTVLAINSIGASVANFNLTFS

WPMSKVNIVQSLSAYP-LNSSCVIVSWILSP----SDYKLMYFIIEWKNLNEDGEIKWLRISSSVKKY----------------------YIHDHFIPIEKYQFSLYPIFMEGVGKPKIINSFTQDDI

Prolactin receptor:

PPGKP-EIFKCRS-PNKETFTCWWRPGT----DGGLPTNYSLTYHREGETLMHECPDYITGGPNSCH--------------------FGKQYTSMWRTYIMMVNATNQMGSSFSDELYVDVTYIVQPD

PPLELAVEVKQPE-DRKPYLWIKWSPPT---LIDLKTGWFTLLYEIRLKPEKAAEWEIHFAGQQTE---------------------FKILSLHPGQKYLVQVRCKPDHGYWSAWSPATFIQIPSD

CRLF1:

PPEKP-VNISCWS-KNMKDLTCRWTPGA--HGETFLHTNYSLKYKLRWYGQDNTCEEYHTVGPHSC---------------------HIPKDLALFTPYEIWVEATNRLGSARSDVLTLDILDVVTTDP

PP-DVHVSRVGGLEDQLSVRWVSPP-ALKDFLFQAKYQIRYRVEDSVDWKVVDDVSNQTS------------------------CRLAGLKPGTVYFVQVRCNPFGIYGSKKAGIWSEWSHPTAA

Integrin beta4:

APQNP--NAKAAG---SRKIHFNWLPPS------GKPMGYRVKYWIQGDSESEAHLLDSKVPS------------------------VELTNLYPYCDYEMKVCAYGAQGEGPYSSLVSCRTHQE

VPSEP-GRLAFNV-VSSTVTQLSWAEPA---ETNGEITAYEVCYGLVNDDNRPIGPMKKVLVDNPKNRM------------------LLIENLRESQPYRYTVKARNGAGWGPEREAIINLATQPK

FSALGPTSLRVSWQEPR----CERPLQGYSVEYQLLNGGELHRLNIPNPAQTS-----------------------VVVEDLLPNHSYVFRVRAQSQEGWGREREGVITIESQVHPQ

LPGSAFTLSTPSAP-GPLVFTA-LSPDSLQLSWERPR---RPNGDIVGYLVTCEMAQGGGPATAFRVDGDSPESR---------------------

LTVPGLSENVPYKFKVQARTTEGFGPEREGIITIESQDGGPFP

ROBO3:

PDPPTEPSSPPGAP-SQPVVTE-ITKNSITLTWKPNP---QTGAAVTSYVIEAFSPAAGNTWRTVADGVQLET-----------------------HTVSGLQPNTIYLFLVRAVGAWGLSEPSPVSEPVRTQDS

VAVRLQEP---IVLGP----RTLQVSWTVDG----PVQLVQGFRVSWRVAGPEGGSWTMLDLQSPSQQS---------------------TVLRGLPPGTQIQIKVQAQGQEGLGAESLSVTRSIPE

PPQGVAVALGGDGNSSITVSWEPPL-PSQQNGVITEYQIWCLGNESRFHLNRSAAGWARS------------------------AMLRGLVPGLLYRTLVAAATSAGVGVPSAPVLVQLPSPPDL

ROBO4:

TLLNP-DPAEGPK--PRPAVWLSWKVSG----PAAPAQSYTALFRTQTAPGGQGAPWAEELLAGWQS--------------------AELGGLHWGQDYEFKVRPSSGRARGPDSNVLLLRLPEK

VPSAPP-QEVTLKP--GNGTVFVSWVPPP-AENHNGIIRGYQVWSLGNTSLPPANWTVVGEQT-------------------------QLEIATHMPGSYCVQVAAVTGAGAGEPSRPVCLLLEQAMERA

Tenascin:

PP-KDLVVTE-VTEETVNLAWDNEM-------RVTEYLVVYTPTHEGGLEMQFRVPGDQTS-----------------------TIIQELEPGVEYFIRVFAILENKKSIPVSARVATYLPAP

EGLKFKS-IKETSVEVEWDPLD------IAFETWEIIFRNMNKEDEGEITKSLRRPETS----------------------YRQTGLAPGQEYEISLHIVKNNTRGPGLKRVTTTRLD

AP-SQIEVKD-VTDTTALITWFKPL------AEIDGIELTYGIKDVPGDRTTIDLTEDENQ-----------------------YSIGNLKPDTEYEVSLISRRGDMSSNPAKETFTTGLD

AP-RNLRRVS-QTDNSITLEWRNGK------AAIDSYRIKYAPISGGDHAEVDVPKSQQATTK---------------------TTLTGLRPGTEYGIGVSAVKEDKESNPATINAATELDTPKD

LQVSE-TAETSLTLLWKTPL------AKFDRYRLNYSLPTGQWVGVQLPRNTTS-------------------------YVLRGLEPGQEYNVLLTAEKGRHKSKPARVKASTEQAP

ELENLTVTE-VGWDGLRLNWTAAD------QAYEHFIIQVQEANKVEAARNLTVPGSLRA-----------------------VDIPGLKAATPYTVSIYGVIQGYRTPVLSAEASTGE

TPNLGEVVVAE-VGWDALKLNWTAPE------GAYEYFFIQVQEADTVEAAQNLTVPGGLRS-----------------------TDLPGLKAATHYTITIRGVTQDFSTTPLSVEVLTEE

VPDMGNLTVTE-VSWDALRLNWTTPD------GTYDQFTIQVQEADQVEEAHNLTVPGSLRS-----------------------MEIPGLRAGTPYTVTLHGEVRGHSTRPLAVEVVTEDLPQL

```
GDLAVSE-VGWDGLRLNWTAAD------NAYEHFVIQVQEVNKVEAAQNLTLPGSLRA-----------------------VDIPGLEAATPYRVSIYGVIRGYRTPVLSAEASTAK
EPEIGNLNVSD-ITPESFNLSWMATD------GIFETFTIEIIDSNRLLETVEYNISGAERT-----------------------AHISGLPPSTDFIVYLSGLAPSIRTKTISATATTEA
LPLLENLTISD-INPYGFTVSWMASE------NAFDSFLVTVVDSGKLLDPQEFTLSGTQRK-----------------------LELRGLITGIGYEVMVSGFTQGHQTKPLRAEIVTEA
EPEVDNLLVSD-ATPDGFRLSWTADE------GVFDNFVLKIRDTKKQSEPLEITLLAPERT-----------------------RDITGLREATEYEIELYGISKGRRSQTVSAIATTAMG
SP-KEVIFSD-ITENSATVSWRAPT------GAQVESFRITYVPITGGTPSMVTVDGTKTQ-----------------------TRLVKLIPGVEYLVSIIAMKGFEESEPVSGSFTTALD
N-ITDSEALARWQPAI------GATVDSYVISYTGEKVPEITRTVSGNTVE------------------------YALTDLEPATEYTLRIFAEKGPQKSSTITAKFTTDLD
E-VQSETALLTWRPPR------GASVTGYLLVYESVDGTVKEVIVGPDTTS------------------------YSLADLSPSTHYTAKIQALNGPLRSNMIQTIFTTIGLL
```

TenascinX:

```
RPQELGELRVLGRDETGRLRVVWTAQP------GDTFAYFQLRMRVPEGPGAHEEVLPGDVRQ-----------------------ALVPPPPPGTPYELSLHGVPPGGKPSDPIIYQGIMDKDEEKP
RLGELTVTD-RTSDSLLLRWTVPE------GEFDSFVIQYKDRDGQPQVVPVEGPQRS-------------------------AVITSLDPGRKYKFVLYGFVGKKRHGPLVAEAKILPQSD
PSPGTPPHLGNLWVTD-PTPDSLHLSWTVPE------GQFDTFMVQYRDRDGRPQVVPVEGPERS-------------------------FVVSSLDPDHKYRFTLFGIANKKRYGPLTADGTTAPERKE
RPEFLEQPLLGELTVTG-VTPDSLRLSWTVAQ------GPFDSFMVQYKDAQGQPQAVPVAGDENE-------------------------VTVPGLDPDRKYKMNLYGLRGRQRVGPESVVAKTAPQEDVDE
GTEAPESPEEPLLGELTVTG-SSPDSLSLFWTVPQ------GSFDSFTVQYKDRDGRPRAVRVGGKESE-------------------------VTVGGLEPGHKYKMHLYGLHEGQRVGPVSAVGVTAPQQEET
TESPLEPRLGELTVTD-VTPNSVGLSWTVPE------GQFDSFIVQYKDKDGQPQVVPVAADQRE-------------------------VTVYNLEPERKYKMNMYGLHDGQRMGPLSVVIVTAPLPPAPA
KPPLEPRLGELTVTD-ITPDSVGLSWTVPE------GEFDSFVVQYKDRDGQPQVVPVAADQRE-------------------------VTIPDLEPSRKYKFLLFGIQDGKRRSPVSVEAKTVARGDA
SPGAPPRLGELWVTD-PTPDSLRLSWTVPE------GQFDSFVVQFKDKDGPQVVPVEGHERS--------------------------VTVTPLDAGRKYRFLLYGLLGKKRHGPLTADGTTEARSAMDD
KRPPKPRLGEELQVTT-VTQNSVGLSWTVPE------GQFDSFVVQYKDRDGQPQVVPVEGSLRE-------------------------VSVPGLDPAHRYKLLLYGLHHGKRVGPISAVAITAGREETET
APTPPAPEPHLGELTVEE-ATSHTLHLSWMVTE------GEFDSFEIQYTDRDGQLQMVRIGGDRND-------------------------ITLSGLESDHRYLVTLYGFSDGKHVGPVHVEALTVPEEEKPS
ATPEPPIKPRLGELTVTD-ATPDSLSLSWTVPE------GQFDHFLVQYRNGDGQPKAVRVPGHEEG-------------------------VTISGLEPDHKYKMNLYGFHGGQRMGPVSVVGVTAAEEETPS
APEPAEEPLLGELTVTG-SSPDSLSLSWTVPQ------GRFDSFTVQYKDRDGRPQVVRVGGEESE-------------------------VTVGGLEPGRKYKMHLYGLHEGRRVGPVSAVGVTAPEE
ESPDAPLAKLRLGQMTVRD-ITSDSLSLSWTVPE------GQFDHFLVQFKNGDGQPKAVRVPGHEDG-------------------------VTISGLEPDHKYKMNLYGFHGGQRVGPVSAVGLTAPGKDEEM
EPPTPEPPIKPRLEELTVTD-ATPDSLSLSWTVPE------GQFDHFLVQYKNGDGQPKATRVPGHEDR-------------------------VTISGLEPDNKYKMNLYGFHGGQRVGPVSAIGVTAAEEETPS
APEPPEEPLLGELTVTG-SSPDSLSLSWTVPQ------GRFDSFTVQYKDRDGRPQVVRVGGEESE-------------------------VTVGGLEPGRKYKMHLYGLHEGRRVGPVSTVGVTAPQEDVDE
APGPPEEPLLGELTVTG-SSPDSLSLSWTVPQ------GRFDSFTVQYKDRDGRPQAVRVGGQESK-------------------------VTVRGLEPGRKYKMHLYGLHEGRRLGPVSAVGVTEDEAETTQ
VPTMTPEPPIKPRLGELTMTD-ATPDSLSLSWTVPE------GQFDHFLVQYRNGDGQPKAVRVPGHEDG-------------------------VTISGLEPDHKYKMNLYGFHGGQRVGPISVIGVTAAEEETPS
APEPPEEPLLGELTVTG-SSPDSLSLSWTIPQ------GHFDSFTVQYKDRDGRPQVMRVRGEESE-------------------------VTVGGLEPGRKYKMHLYGLHEGRRVGPVSTVGVTAPEDEAET
PPNKPRLGELTVTD-ATPDSLSLSWMVPE------GQFDHFLVQYRNGDGQPKVVRVPGHEDG-------------------------VTISGLEPDHKYKMNLYGFHGGQRVGPISVIGVTAAEEETPA
APEPPEEPLLGELTVTG-SSPDSLSLSWTIPQ------GRFDSFTVQYKDRDGRPQVVRVRGEESE-------------------------VTVGGLEPGCKYKMHLYGLHEGQRVGPVSAVGVTAPKDEAE
AVPTMTPEPPIKPRLGELTVTD-ATPDSLSLSWMVPE------GQFDHFLVQYRNGDGQPKAVRVPGHEDG-------------------------VTISGLEPDHKYKMNLYGFHGGQRVGPVSAIGVTEEETPSPT
APEAPEEPLLGELTVTG-SSPDSLSLSWTVPQ------GRFDSFTVQYKDRDGQPQVVRVRGEESE-------------------------VTVGGLEPGRKYKMHLYGLHEGQRVGPVSTVGITAPLPTPLP
VEPRLGELAVAA-VTSDSVGLSWTVAQ------GPFDSFLVQYRDAQGQPQAVPVSGDLRA-------------------------VAVSGLDPARKYKFLLFGLQNGKRHGPVPVEARTAPDTKPS
PRLGELTVTD-ATPDSVGLSWTVPE------GEFDSFVVQYKDKDGRLQVVPVAANQRE-------------------------VTVQGLEPSRKYRFLLYGLSGRKRLGPISADSTTAPLEK
```

ELPPHLGELTVAE-ETSSSLRLSWTVAQ------GPFDSFVVQYRDTDGQPRAVPVAADQRT--------------------------VTVEDLEPGKKYKFLLYGLLGGKRLGPVSALGMTAPEEDTPA
APEPPEEPRLGVLTVTD-TTPDSMRLSWSVAQ------GPFDSFVVQYEDTNGQPQALLVDGDQSK--------------------------ILISGLEPSTPYRFLLYGLHEGKRLGPLSAEGTTGLAPAGQT
SEESRPRLSQLSVTD-VTTSSLRLNWEAPP------GAFDSFLRFGVPSPSTLEPHPRPLLQRELMVPGTRHS----------------AVLRDLRSGTLYSLTLYGLRGPHKADSIQGTARTLSPVLE
SP-RDLQFSE-IRETSAKVNWMPPP------SRADSFKVSYQLADGGEPQSVQVDGQART-------------------------QKLQGLIPGARYEVTVVSVRGFEESEPLTGFLTTVPD
GP-TQLRALN-LTEGFAVLHWKPPQ------NPVDTYDVQVTAPGAPPLQAETPGSAVD--------------------------YPLHDLVLHTNYTATVRGLRGPNLTSPASITFTTGLE
AP-RDLEAKE-VTPRTALLTWTEPP------VRPAGYLLSFHTPGGQNQEILLPGGITS--------------------------HQLLGLFPSTSYNARLQAMWGQSLLPPVSTSFTTGGLRIP

SPEG:

KLAPP----EVPQ-TYQDTALVLWKPGD-----SRAPCTYTLERRVDGESVWHPVSSGIPDCYYN----------------------VTHLPVGVTVRFRVAC--ANRAGQGPFSNSSEKVFVRGTQDS

COL6A3:

MSREVQVFE-ITENSAKLHWERAE-----PPGPYFYDLTVTSAHDQSLVLKQNLTVTD--------------------------RVIGGLLAGQTYHVAVVCYLRSQVRATYHGSFSTKKSQPPPP

IL2RG:

IPWAP-ENLTLHK-LSESQLELNWNNRF-----LNHCLEHLVQYRTDWDHSWTEQSVDYRHK--------------------------FSLPSVDGQKRYTFRVRSRFNPLCGSAQHWSEWSHPIHWGSN

CDON:

PDAP-IILSPPQTHTPDTYNLVWRAGK---DGGLPINAYFVKYRKLDDGVGMLGSWHTVRVPGSENE-------------------
LHLAELEPSSLYEVLMVARSAAGEGQPAMLTFRTSKEKTASSKNTQASSPPV
PEAP-DRPTIST-ASETSVYVTWIPRA---NGGSPITAFKVEYKRMRTSNWLVAAEDIPPSKLS----------------------VEVRSLEPGSTYKFRVIAINHYGESFRSSASRPYQVVGFPNR
FSSRPITGP-HIAYTEA-VSDTQIMLKWTYIP-SSNNNTPIQGFYIYYRPTDSDNDSDYKRDVVEGSKQW--------------------
HMIGHLQPETSYDIKMQCFNEGGESEFSNVMICETKVKRVPGASEYPVKDLS

COL12A1:

PP-SDLNFKI-IDENTVHMSWAKPV------DPIVGYRITVDPTTDGPTKEFTLSASTTE-------------------------TLLSELVPETEYVVTITSYDEVEESVPVIGQLTIQTGS
PP-SNLIAME-VSSKYVKLNWNPSP------SPVTGYKVILTPMTAGSRQHALSVGPQTTT------------------------LSVRDLSADTEYQISVSAMKGMTSSEPISIMEKTQPMK
PP-KDLSFSE-VTSYGFKTNWSPAG------ENVFSYHITYKEAAGDDEVTVVEPASSTS-------------------------VVLSSLKPETLYLVNVTAEYEDGFSIPLAGEETTEEVKG
AP-RNLKVTD-ETTDSFKITWTQAP------GRVLRYRIIYRPVAGGESREVTTPPNQRR-------------------------RTLENLIPDTKYEVSVIPEYFSGPGTPLTGNAATEEVRG
NP-RDLRVSD-PTTSTMKLSWSGAP------GKVKQYLVTYTPVAGGETQEVTVRGDTTN-------------------------TVLQGLKEGTQYALSVTALYASGAGDALFGEGTTLEER
GSP-QDLVTKD-ITDTSIGAYWTSAP------GMVRGYRVSWKSLYDDVDTGEKNLPEDAIH------------------------TMIENLQPETKYRISVFATYSSGEGEPLTGDATTELSQD
SKTLKVDE-ETENTMRVTWKPAP------GKVVNYRVVYRPHGRGKQMVAKVPPTVTS-------------------------TVLKRLQPQTTYDITVLPIYKMGEGKLRQGSGTTASRFK
SP-RNLKTSD-PTMSSFRVTWEPAP------GEVKGYKVTFHPTGDDRRLGELVVGPYDNT------------------------VVLEELRAGTTYKVNVFGMFDGGESSPLVGQEMTTLSD
AP-SNLVISE-RTHRSFRVSWTPPS------DSVDRYKVEYYPVSGGKRQEFYVSRMETS-------------------------TVLKDLKPETEYVVNVYSVVEDEYSEPLKGTEKTLPVP
VVSLNIYD-VGPTTMHVQWQPVG-------GATGYILSYKPVKDTEPTRPKEVRLGPTVND----------------------MQLTDLVPNTEYAVTVQAVLHDLTSEPVTVREVTLPLP
RP-QDLKLRD-VTHSTMNVFWEPVP------GKVRKYIVRYKTPEEDVKEVEVDRSETS-------------------------TSLKDLFSQTLYTVSVSAVHDEGESPPVTAQETTRPVPAP
TNLKITE-VTSEGFRGTWDHGA------SDVSLYRITWAPFGSSDKMETILNGDENT-------------------------LVFENLNPNTIYEVSITAIYPDESESDDLIGSERTLPILTTQ
GP-RNLQVYN-ATSNSLTVKWDPAS------GRVQKYRITYQPSTGEGNEQTTTIGGRQNS------------------------VVLQKLKPDTPYTITVSSLYPDGEGGRMTGRGKTKPLNTVRN

LRVYD-PSTSTLNVRWDHAE------GNPRQYKLFYAPAAGGPEELVPIPGNTNY------------------------AILRNLQPDTSYTVTVVPVYTEGDGGRTSDTGRTLMRG

LARNVQVYN-PTPNSLDVRWDPAP------GPVLQYRVVYSPVDGTRPSESIVVPGNTRM-----------------------VHLERLIPDTLYSVNLVALYSDGEGNPSPAQGRTLPRS

GP-RNLRVFG-ETTNSLSVAWDHAD------GPVQQYRIIYSPTVGDPIDEYTTVPGRRNN-----------------------VILQPLQPDTPYKITVIAVYEDGDGGHLTGNGRTVGLL

PP-QNIHISD-EWYTRFRVSWDPSP------SPVLGYKIVYKPVGSNEPMEAFVGEMTS------------------------YTLHNLNPSTTYDVNVYAQYDSGLSVPLTDQGTTLYLN

VTDLKTYQI--GWDTFCVKWSPHR-------AATSYRLKLSPADGTRGQEITVRGSETS------------------------HCFTGLSPDTDYGVTVFVQTPNLEGPGVSVKEHTTVKPTEAP

EPHB4:

PPSAP--RSVVSR-LNGSSLHLEWSAPL--ESGGREDLTYALRCRECRPGGSCAPCGGDLTFDPGPRDLVEPW--------------VVVRGLRPDFTYTFEVTALNGVSSLATGPVPFEPVNVTTDRE

VPPAVSDIRVTR-SSPSSLSLAWAVPR---APSGAVLDYEVKYHEKGAEGPSSVRFLKTSENR----------------------AELRGLKRGASYLVQVRARSEAGYGPFGQEHHSQTQLDE

IL31RA:

KP-ENISCVY-YYRKNLTCTWSPGK-----ETSYTQYTVKRTYAFGEKHDNCTTNSSTSENRASCS------------------FFLPRITIPDNYTIEVEAENGDGVIKSHMTYWRLENIAKT

EPPKIFRVKPVLGIKRMIQIEWIKPE--LAPVSSDLKYTLRFRTVNSTSWMEVNFAKNRKDKNQT--------------------YNLTGLQPFTEYVIALRCAVKESKFWSDWSQEKMGMTEEE

APCGLELWRVLKPAEA-DGRRPVRLLWKKAR-GAPVLEKTLGYNIWYYPESNTNLTETMNTTNQ--------------------------QLELHLGGESFWVSMISYNSLGKSPVATL

RIPAIQEKSFQCIEVMQACVAEDQLVVKWQSSA------LDVNTWMIEWFPDVDSEPTTLSWESVSQATNWT--------------------IQQDKLKPFWCYNISVYPMLHDKVGEPYSIQAYA

KEGVPSEGP--ETKVEN-IGVKTVTITWKEIP-KSERKGIICNYTIFYQAEGGKGFSKTVNSSILQ------------------------YGLESLKRKTSYIVQVMASTSAGGTNGTSINFKTLSF

IL21R:

PDLVCYTDYLQTVICILEMWNLHP-----STLTLTWQDQYEELKDEATSCSLHRSAHNATHATYT------------------CHMDVFHFMADDIFSVNITDQSGNYSQECGSFLLAESI

KPAPP-FNVTVTF---SGQYNISWRSDY------EDPAFYMLKGKLQYELQYRNRGDPWAVSPRRKLISVDSRSVS-----------LLPLEFRKDSSYELQVRAGPMPGSSYQGTWSEWSDPVIFQTQ

IL11RA:

PPARP--VVSCQA-ADYENFSCTWSPSQ----ISGLPTRYLTSYRKKTVLGADSQRRSPSTGPWPCPQDPLGAAR------------CVVHGAEFWSQYRINVTEVNPLGASTRLLDVSLQSILRPD

PPQGLRVESVPGYPRRLRASWTYPA----SWPCQPHFLLKFRLQYRPAQHPAWSTVEPAGLE----------------------EVITDAVAGLPHAVRVSARDFLDAGTWSTWSPEAWGTPST

IL12RB:

GP-RDLRCYR-ISSDRYECSWQYEG----PTAGVSHFLRCCLSSGRCCYFAAGSATRL-------------------------QFSDQAGVSVLYTVTLWVESWARNQTEKSPEVTLQLYNS

VKYEPPLGDIKVSK--LAGQLRMEWETPD---NQVGAEVQFRHRTPSSPWKLGDCGPQDDDTESC-----------------------LCPLEMNVAQEFQLRRRQLGSQGSSWSKWSSPVCVPPE

NPPQPQVRFSVEQLGQDGRRRLTLKEQPTQLELPEGCQGLAPGTEVTYRLQLHMLSCPCKAKATRTLHL------------------GKMPYLSGA-AYNVAVISSNQFGPGLNQTWHIPAD

THTEP-VALNISV--GTNGTTMYWPARA-------QSMTYCIEWQPVGQDGGLATCSLTAPQDPDPAGMATYSWS-----------RESGAMGQEKCYYITIFASAHPEKLTLWSTVLSTYHFGGNAS

AAGTP-HHVSVKN-HSLDSVSVDWAPSL-LSTCPGVLKEYVVRCRDEDSKQVSEHPVQPTETQ------------------------VTLSGLRAGVAYTVQVRADTAWLRGVWSQPQRFSIEVQ

Interferon gamma receptor2:

PAPQHPKIRLYN----AEQVLSWEPVA--LSNSTRPVVYQVQFKYTDSKWFTADIMSIGVNCTQITATECDFT-------------AASPSAGFPMDFNVTLRLRAELGALHSAWVTMPWFQHYRNV

TVGPP-ENIEVTP--GEGSLIIRFSSPFDIADTSTAFFCYYVHYWEKGGIQQVKGPFRSNS--------------------------ISLDNLKPSRVYCLQVQAQLLWNKSNIFRVGHLSNISCYETM

Nephrin:

PP-SGLKVVS-LTPHSVGLEWKPGF----DGGLPQRFCIRYEALGTPGFHYVDVVPPQATT----------------------FTLTGLQPSTRYRVWLLASNALGDSGLADKGTQLPITTPGL

IL7R:

AP-FDLSVVYREGANDFVVTFNTSH-------LQKKYVKVLMHDVAYRQEKDENKWTHVNLSSTKLT-----------------LLQRKLQPAAMYEIKVRSIPDHYFKGFWSEWSPSYYFRTPEI

Anosmin-1:

PLKPRKELRFTE-LQSGQLEVKWSSKF---NISIEPVIYVVQRRWNYGIHPSEDDATHWQTVAQTTDER-----------------VQLTDIRPSRWYQFRVAAVNVHGTRGFTAPSKHFRSSKDPS

APPAPANLRLANSTVNSDGSVTVTIVWDLPE---EPDIPVHHYKVFWSWMVSSKSLVPTKKKRRKTTDGFQNS-----------------VILEKLQPDCDYVVELQAITYWGQTRLKSAKVSLHFTSTHAT

RPTRPLEVGAPFYQ-DGQLQVKVYWKKTE-----DPTVNRYHVRWFPEACAHNRTTGSEASSGMTHENY-------------------IILQDLSFSCKYKVTVQPIRPKSHSKAEAVFFTTPPCS

KPENLSASFIVQDVNITGHFSWKMAK--ANLYQPMTGFQVTWAEVTTESRQNSLPNSIISQSQILPSDHYV--------------LTVPNLRPSTLYRLEVQVLTPGGEGPATIKTFRTPELPPS

MYBPC3:

IDVPDAPAAPKISNVGEDSCTVQWEPPA--YDGGQPILGYILERKKKSYRWMRLNFDLIQELS-----------------------HEARRMIEGVVYEMRVYAVNAIGMSRPSPASQPFMPIGPP

SEP--THLAVED-VSDTTVSLKWRPPE--RVGAGGLDGYSVEYCPEGCSEWVAALQGLTEHTS-----------------------ILVKDLPTGARLLFRVRAHNMAGPGAPVTTTEPVTVQEI

KPSPP-QDLRVTD-AWGLNVALEWKPPQ--DVGNTELWGYTVQKADKKTMEWFTVLEHYRRTH-----------------------CVVPELIEGNGYYFRVFSQNMVGFSDRAATTKEPVFIPRPG

SPEG:

PDGAP-QVVAVTG----RMVTLTWNPPR---SLDMAIDPDSLTYTVQHQVLGSDQWTALVTGLREPG--------------------WAATGLRKGVQHIFRVLSTTVKSSSKPSPPSEPVQLLEH

KLAPP--EVPQTY---QDTALVLWKPGD-----SRAPCTYTLERRVDGESVWHPVSSGIPDCY-----------------------YNVTHLPVGVTVRFRVACANRAGQGPFSNSSEKVFVRGTQDS

Thrombopoietin receptor:

GPRDPKNSTG---PTVIQLIATETC-CPALQRPHSASALDQSPCAQPTMPWQDGPKQTSPSREASALTAEGGS-----------CLISGLQPGNSYWLQLRSEPDGISLGGSWGSWSLPVTVDLPG

PTPNLHWRE-ISSGHLELEWQHPS---SWAAQETCYQLRYTGEGHQDWKVLEPPLGAR-------------------------GGTLELRPRSRYRLQLRARLNGPTYQGPWSSWSDPTRVETAT

OSMR:

NP-FSVNFEN-VNATNAIMTWKVHS-----IRNNFTYLCQIELHGEGKMMQYNVSIKVNGE----------------------YFLSELEPATEYMARVRCADASHFWKWSEWSGQNFTTLEA

APSEAPDVWRIVSLEP--GNHTVTLFWKPLS-KLHANGKILFYNVVVENLDKPSSSELHSIPAPA-------------------------NSTKLILDRCSYQICVIANNSVGASPASVIVISADPEN

KEVEEERIAG--TEGGFSLSWKPQP------GDVIGYVVDWCDHTQDVLGDFQWKNVGPNTTSTV-------------------ISTDAFRPGVRYDFRIYGLSTKRIACLLEKKTGYSQEL

APSDNP--HVLVDT-LTSHSFTLSWKDYS-----TESQPGFIQGYHVYLKSKARQCHPRFEKAVLSDGSECCKYKIDNPEEKA-----LIVDNLKPESFYEFFITPFTSAGEGPSATFTKVTTPDE

GHR:

NPGLKTNSSKEP-KFTKCRS-PERETFSCHWTDEV--HHGTKNLGPIQLFYTRRNTQEWTQEWKECPDYVSAGENSCY----------------FNSSFTSIWIPYCIKLTSNGGTVDEKCFSVDEIVQPD

PPIALNWTLLNVSLTG--IHADIQVRWEAPRNADIQKGWMVLEYELQYKEVNETKWKMMDPILTTS-----------------------VPVYSLKVDKEYEVRVRSKQRNSGNYGEFSEVLYVTLPQM

CSF3R:

IP-HNLSCLMNLTTSSLICQWEPGP----ETHLPTSFTLKSFKSRGNCQTQGDSILDCVPKDGQSHCC----------------IPRKHLLLYQNMGIWVQAENALGTSMSPQLCLDPMDVVKLEP

MLRTMDP-SPEAAPP--QAGCLQLCWEPWQ---PGLHINQKCELRHKPQRGEASWALVGPLPLEALQ---------------------YELCGLLPATAYTLQIRCIRWPLPGHWSDWSPSLELRTTER

APTVRLDTWWRQRQLDPRTVQLFWKPVP-LEEDSGRIQGYVVSWRPSGQAGAILPLCNTTE--------------------------LSCTFHLPSEAQEVALVAYNSAGTSRPTPVVFSESRGP

ALTRLHAMA-RDPHSLWVGWEPPN------PWPQGYVIEWGLGPPSASNSNKTWRMEQNGRATGF------------------LLKENIRPFQLYEIIVTPLYQDTMGPSQHVYAYSQEM

APSHAP--ELHLKH-IGKTWAQLEWVPEP-PELGKSPLTHYTIFWTNAQNQSFSAILNASSRG-------------------------FVLHGLEPASLYHIHLMAASQAGATNSTVLTLMTLTPEGS

CSF2R:

YPNSGREGTAAQNFSCFI-YNADLMNCTWARGP----TAPRDVQYFLYIRNSKRRREIRCPYYIQDSGTHVGCHL----------------DNLSGLTSR-NYFLVNGTSREIGIQFFDSLLDTKKIERFN

PP-SNVTVRC--NTTHCLVRWKQPRTYQKLSYLDFQYQLDVHRKNTQPGTENLLINVSGDLENR--------------------YNFPSSEPRAKHSVKIRAADVRILNWSSWSEAIEFGSDDG

Tie2/TEK:

KPLNAP-NVIDTGHN--FAVINISSEPYF----GDGPIKSKKLLYKPVNHYEAWQHIQVTNEI-------------------------VTLNYLEPRTEYELCVQLVRRGEGGEGHPGPVRRFTTASI

GLPPP-RGLNLLPK-SQTTLNLTWQPIF-----PSSEDDFYVEVERRSVQKSDQQNIKVPGNLTS----------------------VLLNNLHPREQYVVRARVNTKAQGEWSEDLTAWTLSD

ILPPQP-ENIKISNI-THSSAVISWTILD-----GYSISSITIRYKVQGKNEDQHVDVKIKNATITQ---------------------YQLKGLEPETAYQVDIFAENNIGSSNPAFSHELVTLPES

3.5.5 Identity of mapped 3Fn variants: disease proteins and disease variants

Summary of included proteins containing naturally occurring variants associated with diseases in domains shared with SORL1.

Gene	Protein	Uniprot	#dom	Associated Diseases/Syndromes (CODE)	Variants mapped onto big alignments (from Uniprot entries except where a specific ref is provided)	#variants	#positions	Citations for variants discussed in main text
USH2A	Usherin	O75445	34	Usher syndrome 2A (USH2A)<br>Retinitis pigmentosa 39 (RP39)	USH2A: P1059L, P1212L, A1953G, K2080N, H2116R, C2128Y/C2128F, S2196T, E2238A, A2249D, S2260P, R2292H, R2354H, V2562K, S2639P, D2738N, **W2744C**, G2752R, F2786S, A2795S, R3124G, E3448K, T3462I, W3479C, P3504T, D3515G, **W3521R**, G3529S, G3546R, T3571M, Y3747C, **I3844M**, N3894D, G3895E, R3904K, T3976M, S4054I, R4115C, S4174R, P4232R, P4269R, T4337M, I4386F, T4425M, V4433L, TR4439I, Y4487C, R4570H, Q4592H, Q4662E, G4692R, G4763R, **L4795R**, C4808R, G4817R, P4818L, T4918M<br>RP39: F1442S, P1978S, D2237Y, R2460H, R2573H, N2930K, L3606P, G3618S, S3669R, R3719H, N4094K, R4115C, R4192H, H4248N, T4425M, M4447V, R4674G, L4840P, T4844M	74	73	**W2744C** [199]<br>**W3521R** [200]<br>**I3844M** [225]
FN1	Fibronectin	P02751	17	Glomerulopathy with fibronectin deposits 2 (GFND2)	GFNDS2: **Y973C**, **W1925R**, **L1974R**	3	3	**Y973C** causal of GFND2 [202]. Segregate with disease in four unrelated families<br>**L1974R** causal [202]<br>**W1925R** causal [202]

TNXB	Tenascin-X	P22105	30	Ehlers-Danlos Syndrome, classic-like (EDSCLL)<br>Vesicourecteral reflux 8 (VUR8)	**EDSCLL**: V1108M<br>**VUR8**: T1244R, V3212I	3	3	
TNC	Tenascin	P24821	15	Deafness, autosomal dominant, 56 (DFNA56)	**DFNA56**: V1773M, T1796S	2	2	
L1CAM	Neural cell adhesion molecule L1	P32004	5	Hydrocephalus due to stenosis of the aqueduct of Sylvius (HSAS)<br>Mental retardation, aphasia, shuffling gait, and adducted thumbs syndrome (MASA)	**HSAS**: K655E, A691T, G698R, M741T, V752M, V768F, **Y784C**, L935P, P941L, **W1036L**, Y1070C<br>**MASA**: R632P, S674C, A691D, G698R, V952M, D770N, P941L	16	15	**Y784C** [210]<br>**W1036L** defective protein [201]
TEK	Angiopoietin-1 receptor	Q02763	2	Glaucoma 3, primary congenital, E (GLC3E)	**GLC3E**: **Y611C**	1	1	**Y611C** in a pedigree and mutant protein can't undergo ligand induced phosphorylation [207]
INSR	Insulin receptor	P06213	3	Rabson-Mendenhall syndrome (RMS)<br>Leprechaunism (LEPRCH)<br>Diabetes mellitus, non-insulin-dependent (NIDDM)	**RMS**: S635L, S835I, A842V, P874L, N878S<br>LEPRCH: V657F, W659R, **Y818C**, **I925T**, **R926W**, T937M<br>**NIDDM**: T858A	12	12	**Y818C** abolishes post-translational processing [208,209]<br>**I925T** abolish post-translational processing; abolish insulin binding [209,212]<br>**R926W** markedly impaired isulin binding, impaired post-translational processing [209,212]
IGF1R	Insulin-like growth factor 1 receptor	P08069	4	Insulin-like growth factor 1 resistance (IGF1RES)	**IGF1RES**: R739Q, Y865C	2	2	
DCC	Netrin receptor DCC	P43146	6	Mirror movements 1 (MRMV1)<br>Gaze palsy, familial horizontal, with progressive scoliosis, 2, with impaired intellectual development (HGPPS2)	**MRMV1**: R597P, M743L, V754G, V793G, G805E, A893T<br>**HPPPS2**: Q691K	7	7	

ROBO3	Roundabout homolog 3	Q96MS0	3	Gaze palsy, familial horizontal, with progressive scoliosis, 1 (HGPPS1)	**HGPPS1**: R703P, S705P	2	2	
ROBO4	Roundabout homolog 4	Q8WZ75	2	Aortic valve disease 3 (AOVD3)	**AOVD3**: **Y280S**, H411Q	2	2	
LEPR	Leptin receptor	P48357	4	Leptin receptor deficiency (LEPRD)	**LEPRD**: C604G, L786P	2	2	
PRLR	Prolactin receptor	P16471	2	Multiple fibroadenomas of the breast (MFAB)<br>Hyperprolactinemia (HPRL)	**MFAB**: I170L<br>**HPRL**: H212R	2	2	
SPEG	Striated muscle preferentially expressed protein kinase	Q15772	2	Myopathy, centronuclear, 5 (CNM5)	**CNM5**: G2757V	1	1	
ITGB4	Integrin beta4	P16144	4	Epidermolysis bullosa letalis, with pyloric atresia (EB-PA)	**EB-PA**: R1225H, R1281W	2	2	
CRLF1	Cytokine receptor-like factor 1	O75462	2	Crisponi/Cold-induced sweating syndrome 1 (CISS1)	**CISS1**: P138L, S145P, R216C, F268S, W284C, **R312P**, R340C	7	7	**R312P** [213]
COL6A3	Collagen alpha-3(VI) chain	P12111	1	Dystonia 27 (DYT27)	**DYT27**: R3043H, P3082R	2	2	
IL2RG	Cytokine receptor common subunit gamma	P31785	1	Severe combined immunodeficiency X-linked T-cell-negative/B-cell-positive/NK-cell-negative (XSCID)<br>X-linked combined immunodeficiency (XCID)	**XSCID**: A156V, L162H, L172P/L172Q, C182R, L183S, **R224W**, R226C/R226H, F227C, L230P, C231Y, G232R, W240C, S241I<br>**XCID**: R222C	16	14	**R224W** [214]
CDON	Cell adhesion molecule-related/down-regulated by oncogenes	Q4KMG0	3	Holoprosencephaly 11 (HPE11)	**HPE11**: T684S, P689A, V691M, V780E, T790A, S940R	6	6	
COL12A1	Collagen alpha-1(XII) chain	Q99715	18	Bethlem myopathy 2 (BTHLM2)	**BTHLM2**: R1965C	1	1	
EPHB4	Ephrin type-B receptor 4	P54760	2	Capillary malformation-arteriovenous malformation 2 (CMAVM2)	**CMAVM2**: V469G, G516R	2	2	
IL31RA	Interleukin-31 receptor subunit alpha	Q8NI17	5	Amyloidosis, primary localized cutaneous, 2 (PLCA2)	**PLCA2**: S489F	1	1	
IL21R	Interleukin-21 receptor	Q9HBE5	2	Immunodeficiency 56 (IMD56)	**IMD56**: **R201L**	1	1	**R201L** defective trafficking, misfolding and impaired processing [192,193]

IL11RA	Interleukin-11 receptor subunit alpha	Q14626	2	Craniosynostosis and dental anomalies (CRSDA)	CRSDA: P221R, S245C, R296W	3	3	
IL12RB1	Interleukin-12 Receptor subunit beta	P42701	5	Immunodeficiency 30 (IMD30)	IMD30: **R213W**	1	1	**R213W** [215]
IFNGR2	Interferon gamma receptor 2	P38484	2	Immunodeficiency 28 (IMD28)	IMD28: **R114C**, S124F, G141R, T168N, G227R	5	5	**R114C** misfolding and abnormal glycosylation, mistrafficking, reduced response to INFG [216,217]
NPHS1	Nephrin	O60500	1	Nephrotic syndrome 1 (NPHS1)	NPHS1: R976S, S1016N, G1020V	3	3	
IL7R	Interleukin-7 receptor subunit alpha	P16871	1	Severe combined immunodeficiency autosomal recessive T-cell-negative/B-cell-positive/NK-cell-positive (T(-)B(+)NK(+) (SCID)	SCID: P132S	1	1	
ANOS1	Anosmin-1	P23352	4	Hypogonadotropic hypogonadism 1 with or without anosmia (HH1)	HH1: R262P, N267K, N304S, S396L, E514K, F517L, **W571R**, V587L	8	8	**W571R** in patients with Kallmann Syndrome/HH1 [203]
MYBPC3	Myosin-binding protein C, cardiac-type	Q14896	3	Cardiomyopathy, familial hypertrophic 4 (CMH4)	CMH4: D770N, V771M, **W792R**, R810H, K811R, R820Q, A833T/A833V, R834W, P873H, N948T, T957S, T958I, I1131T	14	13	**W792R** in patients with CMH4 [204]
MPL	Thrombopoietin receptor	P40238	2	Congenital amegakaryocytic thrombocytopenia (CAMT)	CAMT: **R257L**, P257T, W435C	3	3	**R257L** [218]
OSMR	Oncostatin-M-specific receptor subunit beta	Q99650	4	Amyloidosis, primary localized cutaneous, 1 (PLCA1)	PLCA1: G618A, D647V, I691T, P694L, K697T	5	5	
GHR	Growth hormone receptor	P10912	(2)	Laron syndrome (LARS)<br>Growth hormone insensitivity, partial (GHIP)	LARS: C56S, S58L, **W68R**, R89K, F114S, V143A, P149Q, V162D, D170H, I171T, Q172P, V173G, **Y226C**, R229G, S244I<br>GHIP: E62K, R179C	17	17	**Y226C** is causal of severe growth retardation [211]<br>**W68R** in LARS patient [205]
CSF3R	Granulocyte colony-stimulating factor receptor	Q99062	5	Neutropenia, severe congenital 7, autosomal recessive (SCN7)	SCN7: **R308C**	1	1	**R308C** Decreased localization at cell surface and less

								signaling. Impaired glyco-sylation [219]
CSF2RA	Granulocyte-macrophage colony-stimulating factor receptor subu-nit alpha	P15509	2	Pulmonary surfactant metabolism dysfunction 4 (SMDP4)	**SMDP4**: G196R	1	1	
						**229**	224	

3.5.6 3Fn disease variants listed according to domain positions

Disease-mutations domain-mapping analysis with identification of pathogenic variants in other proteins with 3Fn-domains (as listed in **Supplemental Information 3.5.5**). Here variants are mapped onto domain positions following alignment of internally repeated sequences in the SORL1 domain sequences. The number of hits for each position depicted in the bar diagram of **Supplemental Figure S11e**.

**3Fn-domain positions**	Number of hits	Identified variants		Priority
1				
2				
3				
4				
5				
6; P	6	p.P1059L(USH2A), p.N2930K(USH2A), p.P3504T(USH2A), p.R739Q(IGF1R), p.A156V(IL2RG), p.P873H(MYBPC3)	Loss of Pro *R739 of IGF1R part of furin site*	high
7; P	4	p.P4269R(USH2A), p.P138L(CRLF1), p.P221R(IL11RA), p.P132S(IL7R)	Loss of Pro	high
8	0			
9	1	p.T858A(INSR)		
10	1	p.R1255H(ITGB4)		
11; **ø**	2	p.A2249D(USH2A), p.V162D(GHR)	Loss of hydrophobic	moderate
12	0			
13; **ø**	2	p.L162H(IL2RG), p.C56S(GHR)	Loss of hydrophobic	moderate
14	2	p.T3976M(USH2A), p.L4840P(USH2A)		
15	3	p.S145P(CRLF1), p.N304S(ANOS1), p.S58L(GHR)		
16	0			
17	0			
18	1	p.S635L(INSR)		
19	4	p.D2738N(USH2A), p.R3124G(USH2A), p.D3515G(USH2A), p.E62K(GHR)		
20	3	p.S2639P(USH2A), p.T4844M(USH2A), p.D170H(GHR)		
21; **ø**	2	p.L3606P(USH2A), p.I171T(GHR)	Loss of hydrophobic	moderate
22	3	p.R632P(L1CAM), p.L935P(L1CAM), p.Q172P(GHR)		
23; **ø**	4	p.M743L(DCC), p.L172P(IL2RG), p.L172Q(IL2RG), p.V173G(GHR)	Loss of hydrophobic	moderate

24	2	p.S2260P(USH2A), p.S4174R(USH2A)		
25; **W**	7	p.W2744C(USH2A), p.W3521R(USH2A), p.W1036L(L1CAM), p.W1925R(FN1), p.W571R(ANOS1), p.W729R(MYBPC3), p.W68R(GHR)	Loss of Trp	high
26	0			
27; p	1	p.D647V(OSMR)		
28; p	3	p.P1978S(USH2A), p.P941L(L1CAM), p.P874L(INSR)	Loss of Pro	high
29	1	p.R179C(GHR)		
30	0			
31	1	p.R2460H(USH2A)		
32	1	p.M741T(L1CAM)		
33	3	p.K2080N(USH2A), p.R2354H(USH2A), p.Q4662E(USH2A)		
34	2	p.S245C(IL11RA), p.T168N(INGR2)		
35	4	p.N4094K(USH2A), p.N3894D(USH2A), p.N878S(INSR), p.F268S(CRLF1)		
36; G	5	p.G2752R(USH2A), p.G3529S(USH2A), p.G3618S(USH2A), p.G3895E(USH2A), p.G4763R(USH2A)	Loss of Gly	high
37	2	p.V2562A(USH2A), p.V754G(DCC)		
38	2	p.I4386F(USH2A), p.C182R(IL2RG)		
39	1	p.L183S(IL2RG)		
40	0			
41; **Y**	3	p.Y865C(IGF1R), p.Y280S(ROBO4), p.Y217D(ANOS1)[226]	Loss of Tyr	high
42	3	p.R751P(L1CAM), p.R703P(ROBO3), p.R1965C(COL12A1)		
43; **ø**	4	p.V752M(L1CAM), p.V657F(INSR), p.V469G(EPHB4), p.V587L(ANOS1)	Loss of hydrophobic	moderate
44	2	p.S705P(ROBO3), p.R976S(NPHS1)		
45; **Y**	6	p.R3719H(USH2A), p.R3904K(USH2A), p.R4192H(USH2A), p.R4674G(USH2A), p.W659R(INSR), p.R810H(MYBPC3)		moderate
46	2	p.R4570H(USH2A), p.K811R(MYBPC3)		
47	1	p.I170L(PRLR)		
48	4	p.E3448K(USH2A), p.R2573H(USH2A), p.K655E(L1CAM), p.R89K(GHR)		
49	0			
50		*Alignment not precise enough*		
51		*Alignment not precise enough*		
52		*Alignment not precise enough*		
53		*Alignment not precise enough*		
54		*Alignment not precise enough*		
55		*Alignment not precise enough*		
56		*Alignment not precise enough*		
57		*Alignment not precise enough*		
58		*Alignment not precise enough*		
59		*Alignment not precise enough*		
60		*Alignment not precise enough*		

61		*Alignment not precise enough*		
62		*Alignment not precise enough*		
63		*Alignment not precise enough*		
64		*Alignment not precise enough*		
65		*Alignment not precise enough*		
66		*Alignment not precise enough*		
67		*Alignment not precise enough*		
68		*Alignment not precise enough*		
69		*Alignment not precise enough*		
70		*Alignment not precise enough*		
71		*Alignment not precise enough*		
72; **ø**	4	p.S674C(L1CAM), p.R3043H(COL6A3), p.V780E(CDON), p.F114S(GHR)		moderate
73	1	p.T3462I(USH2A)		
74; **ø**	2	p.A833T(MYBPC3), p.A833V(MYBPC3)		moderate
75	1	p.R834W(MYBPC3)		
76	0			
77; **L**	1	p.L4795R(USH2A)		high
78	1	p.H411Q(ROBO4)		
79; P	3	p.P1212L(USH2A), p.P4232R(USH2A), p.I1131T(MYBPC3)	Loss of Pro	high
80	2	p.F2786S(USH2A), p.C604G(LEPR)		
81	1	p.V1773M(TNC)		
82	3	p.Q691K(DCC), p.T790A(CDON), p.S489F(IL31RA)		
83; **Y**	6	p.H2116R(USH2A), p.Y784C(L1CAM), p.Y818C(INSR), p.Y973C(FN1), p.Y226C(GHR), p.Y611C(TEK)	Loss of Tyr	high
84	1	p.V793G(DCC)		
85; **ø**	2	p.F1442S(USH2A), p.Y3747C(USH2A)		moderate
86	4	p.R597P(DCC), p.R222C(IL2RG), p.R262P(ANOS1), p.R229G(GHR)	Loss of Arg	
87; **ø**	3	p.I3844M(USH2A), p.I925T(INSR), p.L1974R(FN1)	Loss of hydropho-bic	moderate
88	8	p.R926W(INSR), p.R312P(CRLF1), p.R224W(IL2RG), p.R201L(IL21R), p.R213W(IL12RB), p.R114C(INGR2), p.R257L(MPL), p.R308C(CSF3R)	Loss of Arg	high
89; **a**	2	p.A2795S(USH2A), p.A691T(L1CAM)	Loss of Ala (or hyd.)	moderate
90	6	p.W3479C(USH2A), p.C4808R(USH2A), p.R226C(IL2RG), p.R226H(IL2RG), p.R296W(IL11RA), p.S1016N(NPHS1)		
91	6	p.T3571M(USH2A), p.T4337M(USH2A), p.T4425M(USH2A), p.F227C(IL2RG), p.N276K(ANOS1), p.N948T(MYBPC3)		
92	1	p.R216C(CRLF1)		
93	1	p.H212R(PRLR)		
94; g	2	p.L230P(IL2RG), p.G1020V(NPHS1)	Loss of Gly	moderate
95	4	p.H4248N(USH2A), p.C2128Y(USH2A), p.C2128F(USH2A), p.C231Y(IL2RG)		

96; g	7	p.T4918M(USH2A), p.G698R(L1CAM), p.G805E(DCC), p.G232R(IL2RG), p.G516R(EPHB4), p.G1020V(NPHS1), p.G2757V(SPEG)	Loss of Gly	high
97	0			
98; s	3	p.S3669R(USH2A), p.S4054I(USH2A), p.S124F(INGR2)	Loss of Ser?	
99		*Alignment not precise enough*		
100		*Alignment not precise enough*		
101		*Alignment not precise enough*		
102		*Alignment not precise enough*		
103		*Alignment not precise enough*		
104		*Alignment not precise enough*		
105		*Alignment not precise enough*		
106		*Alignment not precise enough*		
107		*Alignment not precise enough*		
108		*Alignment not precise enough*		
109		*Alignment not precise enough*		
110		*Alignment not precise enough*		
111		*Alignment not precise enough*		
	**173***			

* A number of identified variants (listed in **Supplemental Information 3.5.5**) map within 3Fn-domain sequences where the alignment does not allow unambiguous domain position identification

3.6 Transmembrane and cytoplasmic domains (residues 2122-2214)

3.6.1 Sequence details

Membrane-anchoring domain

Immediately following the 3Fn-domains is a 16 amino acid long stalk region (residues 2122-2137) suggested to lift the ectodomain a short distance from the plasma membrane, enabling TACE-dependent cleavage, leading to ectodomain shedding when mature SORL1 is at the cell surface [194,227]. After the stalk region, SORL1 contains a 23-amino acid single-pass transmembrane (TM) domain (residues 2138-2160), presumably alpha-helical, and a cytoplasmic domain (CD; often referred to as the 'tail') including 54 amino acids (residues 2161-2214) (**Fig. 4**).

The TM-domains of mammalian SORL1 proteins are highly conserved during evolution, with human and insects sharing >95% sequence identity [228] (**Supplemental Figure S12**). The number of amino acids in the TM-domain may influence the localization of a protein within the cell [229]. With this reasoning, the number of residues in the SORL1 TM-domain might underlie its manifestation in the membranes of the trans-Golgi and trafficking network. Similar to most membrane-anchored proteins, the amino acid composition of the SORL1 TM-domain is mainly characterized by hydrophobic residues that form non-polar interactions that stabilize the helix structure within the phospholipid bilayer. Therefore, the preservation of the hydrophobicity of TM-domains is key for proper insertion of the protein into the membrane: substitutions of hydrophobic residues with polar or charged residues in the LDLR TM-domain prevented membrane insertion, which was causative for familial hypercholesterolemia [230,231]. Thus far, it is unknown whether mutations in the SORL1-TM domain will have similar effects.

The intracellular cytoplasmic domain

Similar to most members of the LDLR and VPS10p receptor families, a short (~50 amino acid) cytoplasmic tail constitutes the intracellular part of SORL1. This tail determines the complex intracellular sorting itinerary that SORL1 follows (**Supplemental Figure S12**). Binding to different intracellular adaptor proteins via short motifs in the tail determines key sorting steps of SORL1 and its cargo, such as endocytosis and intracellular trafficking between Golgi/TGN and endo-lysosomal compartments (see below) (**Supplemental Figure S12**). Deletion of the entire tail leads to the direct vesicular transportation of the receptor from the TGN to the

plasma membrane and, upon cleavage by sheddase (i.e. TACE), its release into extracellular space [99,232].

GGA

The very C-terminal part of SORL1 contains a DXXLL-like motif (^{2208}DVPMV^{2212}), which is a target for the Golgi-localized proteins GGA1, GGA2 and GGA3. These adaptors regulate trafficking of a set of membrane-spanning proteins from Golgi to endosomal compartments and – at least in the case of GGA3 – from endosome towards lysosome [233,234] [235]. This suggests that SORL1 can sort directly from Golgi to the endosome after its synthesis. The minimal motif required for binding of GGA adaptors to the SORL1 cytoplasmic domain is characterized by two acidic residues ^{2207}DD^{2208} preceding methionine M^{2211} of the hydrophobic cluster at the very C-terminal end [236]. In 2009, the structure of part of the SORL1 tail was determined in complex with the VHS domain of GGA1 [237] confirming that the GGA adaptor directly binds these residues. This highly conserved motif is crucial for SORL1 function: a mutation in this motif yields a receptor with compromised trafficking to the cell surface [232,238,239]. The overlapping ^{2212}VIA^{2214} motif was recently shown to bind PICK1, suggesting that also this protein is capable of regulating SORL1's intracellular itinerary [240].

FANSHY

The SORL1 tail also includes the ^{2172}FANSHY^{2177} motif, which is strictly conserved from human to insects suggesting an indispensable physiological function (**Supplemental Figures S12**). This motif is similar to the (F/Y)XNPXY motif as identified in the cytoplasmic domain of many proteins including APP, LDLR, and LRP, in which it serves as an endocytosis signal [241]. However, in SORL1, the Pro residue is absent, and here, the motif is instead required for binding the retromer core complex (VPS26, VPS29 and VPS35), which is involved in the recycling of various transmembrane receptors between the endosomal network to the cell surface or in the retrograde trafficking to the Golgi/TGN [242]. The Phe-2172 of the ^{2172}FANSHY motif is essential for association of the SORL1 tail with the VPS26 subunit of the retromer complex [243].

The tail-motif of several receptors is the simple NXXY motif, i.e. also without a proline residue as well as the preceding aromatic F/Y residue, and these receptors can be bound to endosomal recycling proteins such as SNX17, SNX27, and SNX31 [244,245], raising the possibility that the FA**N**SH**Y** site in the SORL1 tail may interact not also interact with such sorting complexes for endosomal sorting, which may assist SORL1 cycling from endosomes to the cell surface [246]. Interestingly, a fragment of the intracellular domain spanning the FANSHY motif folds into an amphiphatic α-helical structure with the potential for sensing membrane curvature as yet another determinant of SORL1 intracellular transport [247].

Acidic

An acidic cluster in the tail of SORL1 (corresponding to residues ^{2190}DDLGEDDED^{2198}) is reported to bind cytoplasmic adaptor proteins including PACS1, depending on cellular localization and cell type [232,248-250]. In clathrin-vesicles, SORL1-binding goes via the AP1 and AP2 adaptor-binding proteins, which bind the EXXXLL-like motif (^{2197}EDAPMI^{2202}). Interactions with the AP1, AP2 and PACS1 adaptor proteins regulate mainly retrograde sorting pathways from cell surface to endosome (AP2) or endosomes to TGN (PACS1). However, AP1 is also involved in directing cargo in the anterograde direction from TGN towards endosomes, and described to do so partly in concert with GGAs [251,252]. Receptors carrying substitutions within this acidic motif have a strong defect in endocytosis, due to lack of AP2 binding [232,248]. It has also been reported that the relatively unknown HSPA12A cytosolic protein binds to the acidic motif within the tail of SORL1 [253].

Phosphorylation

Twelve of the 54 amino acids of the SORL1 tail are potential targets of phosphorylation (Ser/Thr/Tyr) and several of these residues occur in consensus target sequences for specific kinases [254], suggesting phosphorylation might play important functions in the sorting of SORL1 (**Fig. 4**). For example, the ROCK2 kinase can phosphorylate Ser (S^{2206}), which modifies the cytoplasmic domain of SORL1 such that ectodomain shedding of SORL1 is increased [255]. Particularly, the presence of both a tyrosine and a serine in the FANSHY motif may provide possible phosphorylation-dependent regulatory mechanisms of SORL1 endosomal sorting. The significance of such modifications is currently under investigation.

Andersen
Supplemental Figure S12 - Tail-domain

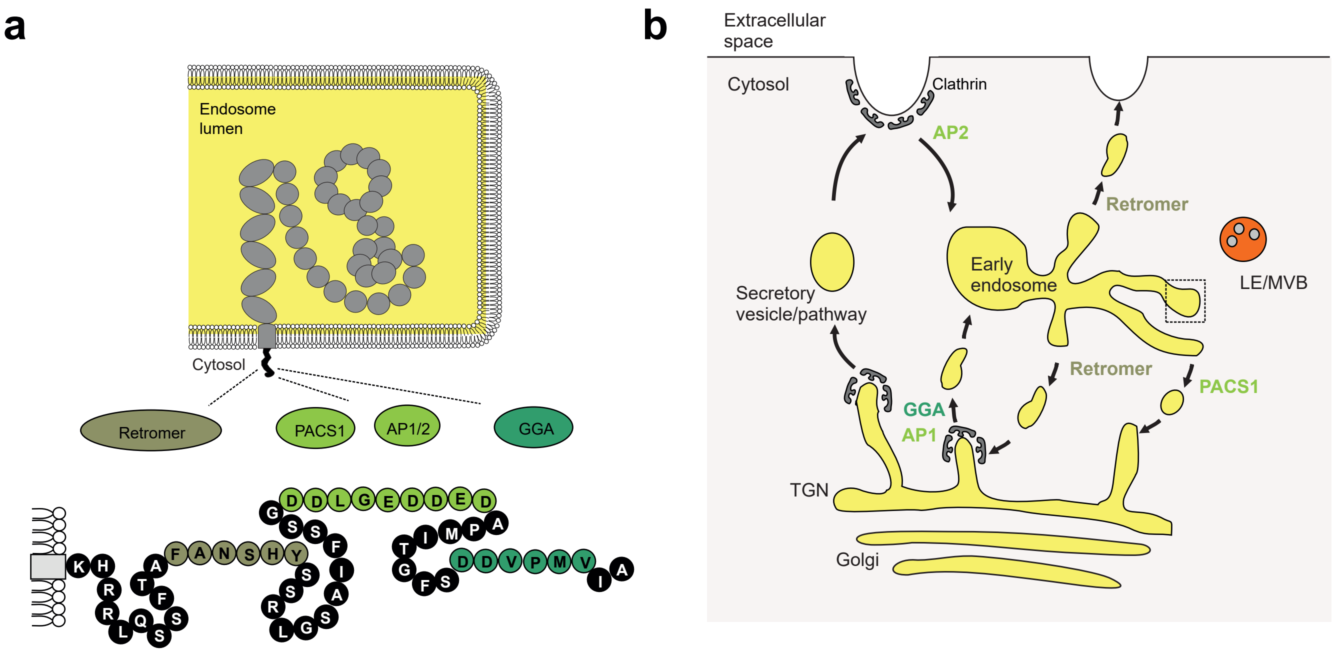

c

		Stalk	Transmembrane	Cytoplasmic tail
				1 10 20 30 40 50
Human	2122-2214	GADASATQAARSTDVA	AVVVPILFLILLSLGVGFAILYT	KHRRLQSSFTA**FANSHY**SSRLGSAIFSSG**DDLGEDDED**APMITGFS**DDVPMVIA**
Monkey		GADASAMQAARSTDVA	AVVVPILFLILLSLGVGFAILYT	KHRRLQSSFTAFANSHYSSRLGSAIFSSGDDLGEDDEDAPMITGFSDDVPMVIA
Pig		GRDVSAIQATRSTDVA	AVVVPILFLILLSLGIGFAILYT	KHRRLQNSFTAFANSHYSSRLGSAIFSSGDDLGEDDEDAPMITGFSDDVPMVIA
Rat		GGDATVVQTARSTDVA	AVVVPILFLILLSLGVGFAVLYT	KHRRLQSSFTAFANSHYSSRLGSAIFSSGDDLGEDDEDAPMITGFSDDVPMVIA
Mouse		GADAAVIQAARSTDVA	AVVVPILFLILLSLGVGFAILYT	KHRRLQSSFSAFANSHYSSRLGSAIFSSGDDLGEDDEDAPMITGFSDDVPMVIA
Dog		GGGASAFQAARSTDVA	AVVVPILFLILLSLGVGFAILYT	KHRRLQSSFTAFANSHYSSRLGSAIFSSGDDLGEDDEDAPMITGFSDDVPMVIA
Cat		GAGASSARAARSTDVA	AVVVPILFLILLSLAVGFAVLYT	KHRRLRSSFTAFANSHYSSRLGSAIFSSGDDLGEDDEDAPMITGFSDDVPMVIA
Horse		GGDASAFQAARSTDVA	AVVVPILFLILLSLGVGFAILYT	KHRRLQSSFTAFANSHYSSRLGSAIFSSGDDLGEDDEDAPMITGFSDDVPMVIA
Cow		GGDASTIQAARSTDVA	AVVVPILFLILLSLGVGFAILYT	KHRRLQNSFTAFANSHYSSRLGSAIFSSGDDLGEDDEDAPMITGFSDDVPMVIA
Frog		--DPLYSKNVQSTDVA	AIVVPILFLLLVATGFGFVILYT	RHRRLQNSFTAFANSHYSSRLGAAIFSSGDDIG-DDDDAPMITGFSDDVPMVIA
Zebrafish		GQNDAASQSGKSEDMA	AIVVPVLFLLLVGVCGGLVVLYL	RHRRLQHSFTAFANSHYNSRLGSAIFSSGDELGDDDEDAPMISGFSDDVPMVIA
Parasitoid		SSLAPWPATINSTNML	SIAIPICLLI-VALGVGLAYFFV	RHRRLSNSFTQFANSHYDTRRGQATFPGTTDAL-DEEDSPVIRGFSDDEPLVIA
Honey bee		AEVSSLPVTINTSNIL	SFAIPICLLII-ALGSALAYFVV	RHRRLTNSFTQFANSHYDTRRGQATFPGTIDGL-EEEDSPVIRGFSDDEPLVIA
Termite		MPVGSWKAVLSPRNIV	SVVVPVGLVMIALCGA-LAFFVL	RHRRLQRSFSSFANSHYDTRSGAATFSGGDGL--DEEDSPVIRGFSDDEPLVIA
Plant bug		VPLGAWASVMTPTRVM	GIFVPICLVLIIVAGA-FVIFIV	RHRRLQNNFVTFANSHYSTRSGAATFTT-DGL--DDEDSPVIRGFSDDEPLVVA
				**FANSHY** **DDLGEDDED** **DDVPMVIA**

3.6.2 Supplemental Figure S12. Transmembrane and cytoplasmic domains

a. The cytosolic tail of SORL1 contains different motifs that recognize molecular adaptors responsible of the intracellular localization of the receptor, incl sites for binding to Retromer (motif: FANSHY), PACS1 and AP1/AP2 (acidic motif: DDLGEDDED), and GGA (motif: DDVPMVIA).

b. Schematic of the intracellular trafficking pathways in which different adaptor proteins determine if SORL1 goes into the secretory, endocytic, endosome delivery, or endosome recycling pathways.

c. Alignment of the human SORL1 residues 2122-2214 with the corresponding regions of SORL1 from different species including mammals, fish and insects. Residues 2138-2160 correspond to the transmembrane segment (yellow box) forming an α-helical structure (lower panel). The three motifs in the cytoplasmic tail known to interact with adaptor proteins are shown below the alignment in bold letters.

3.6.3 *SORL1* variants in transmembrane and tail-domains

The 16 residues that make up the stalk and transmembrane, represent a small fraction of the entire SORL1 protein. The ADES-ADSP dataset includes a variant that maps to the stalk region: the relatively prevalent p.T2134M variant which was observed in 22 cases and 13 controls [72], associating with a 1.7-fold increased AD risk (OR = 1.72 95% CI: 0.87 - 3.42 p=0.08), however, the much larger GWAS study does not support a pathogenic role of this variant (OR=1.14; p=0.4904) [256]. Functional testing indicated that mutated receptor was unable to protect APP from cleavage into Aβ by γ-secretase when compared to non-mutated SORL1, likely because the formation of a complex with APP was perturbed [257]. Also, lower levels of SORL1 located to the cell surface of transfected cells, suggesting impaired trafficking properties of mutant SORL1.

Little is known about how the 23-residue TM region affects SORL1 function, making it difficult to assess whether a variant will provide AD risk. But as pathogenic variants in the TM of LDLR have been reported to associate with FH [230], some variants in the SORL1 TM region could also be speculated to be pathogenic.

Position 3 in the cytoplasmic tail, very close to the TM region, is part of a highly conserved sequence rich in positively charged amino acids (^{2161}KHRR^{2164}). These four residues are suggested to constitute a nuclear localization motif that is responsible for the translocation of the cytoplasmic tail of SORL1 when liberated from the membrane after γ-secretase cleavage and with a role in signaling processes [258]. The ADES-ADSP dataset includes two variants at this position: p.R2163W observed in one case and p.R2163Q observed in one control. The dataset further includes several variants within the conserved ^{2172}FANSHY motif, which may lead to a perturbed interaction with retromer and perturbed engagement in endosomal recycling. The ADES-ADSP dataset includes variants p.A2173T (conservation: 40/40; "*likely pathogenic*") observed in an AD case, p.S2175R (resulting from two independent genetic changes with different allele frequencies; conservation 40/40) that was observed in 11 cases and 10 controls, such that pathogenicity of this substitution is questionable. The dataset further includes the p.H2176R substitution, observed in one AD case. Together, the assessment of pathogenicity-levels for substitutions in the FANSHY motif require additional testing of variant effects. Finally, the ADES-ADSP dataset includes 3 variants that substitute key residues of the acidic/DXXXLL and gga/DXXVI: p.D2190N observed in an AD case and in a control, p.E2194K in two AD cases and a control and p.D2207G in an AD case. While such very

conserved variants are likely linked with SORL1 dysfunction, we observed them both in cases and controls such that more evidence is necessary to estimate their effects.

3.7 SORL1 sequences for alignment: species conservation

A total of 40 different SORL1 protein sequences are included in the species alignment (**Supplemental Information 3.8**).

This alignment allows look-up if genetic variants that make substitutions in the human protein leads to inclusion of amino acids present in SORL1 from other species – in which case such a variant is more likely to be tolerated than if the position is strictly conserved across all species.

Homo sapiens (human): NP_003096.2/Q92673-1
Macaca mulatta (rhesus macaque): XP_014971461.2/H9ZCQ1-1
Pan troglodytes (chimpanzee): XP_016777658.1/H2Q4Z6-1
Sus scrofa (pig): XP_020918668.1/I3L8K1-1
Capra hircus (goat): XP_017915127.1/A0A452FGN5-1
Ovis aries (sheep): XP_027835138.1/W5QA68-1
Equus caballus (horse): XP_023500779.1/F7CG82-1
Bos taurus (cow): NP_001179686.1/E1BPZ1-1
Loxodonta africana (elephant): XP_003418317.2/G3T328-1
Canis lupus familiaris (dog): XP_536545.2/ E2R5F5-1
Canis lupus dingo (wolf): XP_025320979.1
Vulpes vulpes (fox): XP_025854818.1/A0A3Q7SF13-1
Ursus arctos horribilis (bear): XP_026361326.1
Felis catus (cat): XP_023094970.1/M3WIG3-1
Panthera pardus (leopard): XP_019324393.1
Oryctolagus cuniculus (rabbit): NP_001076133.1/Q95209-1
Rattus norvegicus (rat): NP_445971.1/P0DSP1-1
Mus musculus (mouse): NP_035566.2/O88307-1
Ornithorhynchus anatinus (platypus):XP_028930988.1/F7D9P5-1
Gallus gallus (chicken): NP_001292089.1/E1BUD4-1
Anas platyrhynchos (duck): XP_027299930.1
Columba livia (pigeon): XP_021152551.1/A0A2I0LS76-1
Haliaeetus leucocephalus (eagle): XP_010579730.1

Falco cherrug (falcon):	XP_027662563.1
Aptennodytes forsteri (penguin):	XP_009279556.1
Danio rerio (zebra fish):	XP_005157607.1/X1WHE3-1
Salmo salar (salmon):	XP_014017958.1/A0A1S3NRA8-1
Amphiprion ocellaris (clown fish):	XP_023117170.1
Pygocentrus nattereri (piranha):	XP_017568534.1
Esox lucius (pike):	XP_010891973.1
Hippocampus comes (seahorse):	XP_019716899.1
Rhincodon typus (whale shark):	XP_020382540.1
Xenopus tropicalis (frog):	XP_031762503.1/A0A1L8FLB9-1
Pelodiscus sinensis (turtle):	XP_025045904.1/ K7F2T1-1
Alligator mississippiensis (alligator):	XP_014449241.1/ A0A151NQK7-1
Crocodylus porosus (crocodile):	XP_019402601.1
Gekko japonicus (gecko):	XP_015277064.1
Python bivittatus (python):	XP_007431813.2
Anolis carolinensis (anole):	XP_016850209.1/ H9G6H6-1
Podarcis muralis (lizard):	XP_028563124.1

3.8 SORL1 sequence alignment

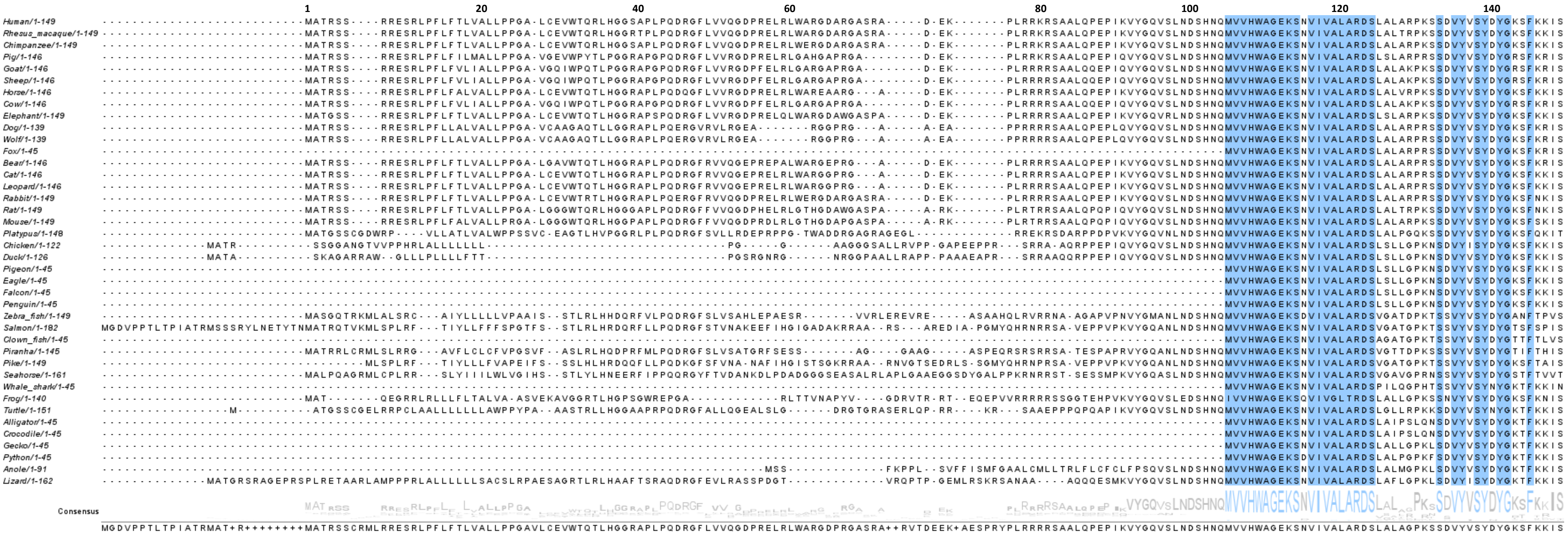
1
20
40
60
80
100
120
140
Human/1-149
Rhesus_macaque/1-149
Chimpanzee/1-149
Pig/1-146
Goat/1-146
Sheep/1-146
Horse/1-146
Cow/1-146
Elephant/1-149
Dog/1-139
Wolf/1-139
Fox/1-45
Bear/1-146
Cat/1-146
Leopard/1-146
Rabbit/1-149
Rat/1-149
Mouse/1-149
Platypus/1-148
Chicken/1-122
Duck/1-126
Pigeon/1-45
Eagle/1-45
Falcon/1-45
Penguin/1-45
Zebra_fish/1-149
Salmon/1-182
Clown_fish/1-45
Piranha/1-145
Pike/1-149
Seahorse/1-161
Whale_shark/1-45
Frog/1-140
Turtle/1-151
Alligator/1-45
Crocodile/1-45
Gecko/1-45
Python/1-45
Anole/1-91
Lizard/1-162
Consensus

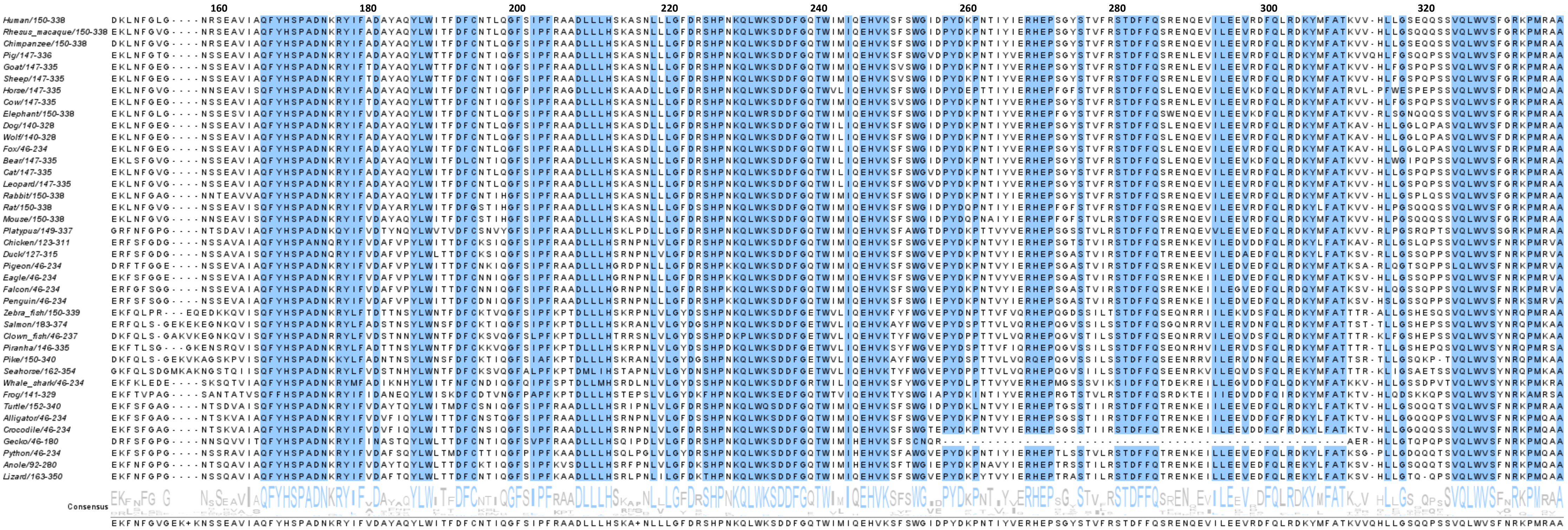
160
180
200
220
240
260
280
300
320
Human/150-338
Rhesus_macaque/150-338
Chimpanzee/150-338
Pig/147-336
Goat/147-335
Sheep/147-335
Horse/147-335
Cow/147-335
Elephant/150-338
Dog/140-328
Wolf/140-328
Fox/46-234
Bear/147-335
Cat/147-335
Leopard/147-335
Rabbit/150-338
Rat/150-338
Mouse/150-338
Platypus/149-337
Chicken/123-311
Duck/127-315
Pigeon/46-234
Eagle/46-234
Falcon/46-234
Penguin/46-234
Zebra_fish/150-339
Salmon/183-374
Clown_fish/46-237
Piranha/146-335
Pike/150-340
Seahorse/162-354
Whale_shark/46-234
Frog/141-329
Turtle/152-340
Alligator/46-234
Crocodile/46-234
Gecko/46-180
Python/46-234
Anole/92-280
Lizard/163-350
Consensus

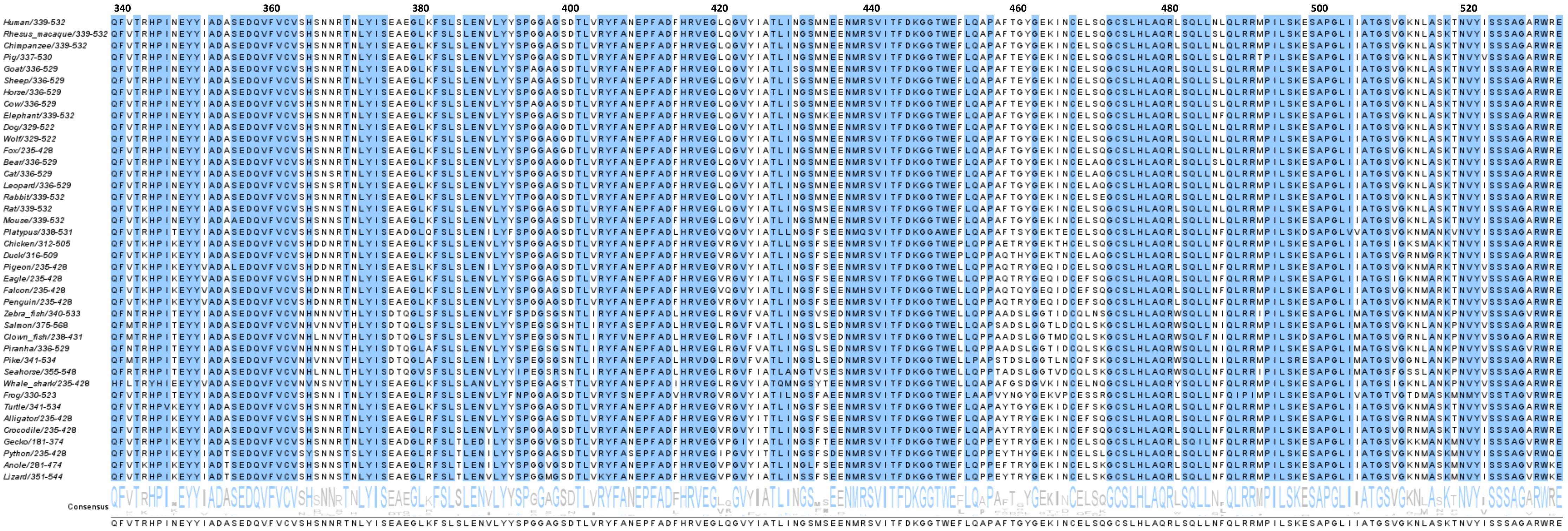
340
360
380
400
420
440
460
480
500
520
Human/339-532
Rhesus_macaque/339-532
Chimpanzee/339-532
Pig/337-530
Goat/336-529
Sheep/336-529
Horse/336-529
Cow/336-529
Elephant/339-532
Dog/329-522
Wolf/329-522
Fox/235-428
Bear/336-529
Cat/336-529
Leopard/336-529
Rabbit/339-532
Rat/339-532
Mouse/339-532
Platypus/338-531
Chicken/312-505
Duck/316-509
Pigeon/235-428
Eagle/235-428
Falcon/235-428
Penguin/235-428
Zebra_fish/340-533
Salmon/375-568
Clown_fish/238-431
Piranha/336-529
Pike/341-534
Seahorse/355-548
Whale_shark/235-428
Frog/330-523
Turtle/341-534
Alligator/235-428
Crocodile/235-428
Gecko/181-374
Python/235-428
Anole/281-474
Lizard/351-544
Consensus

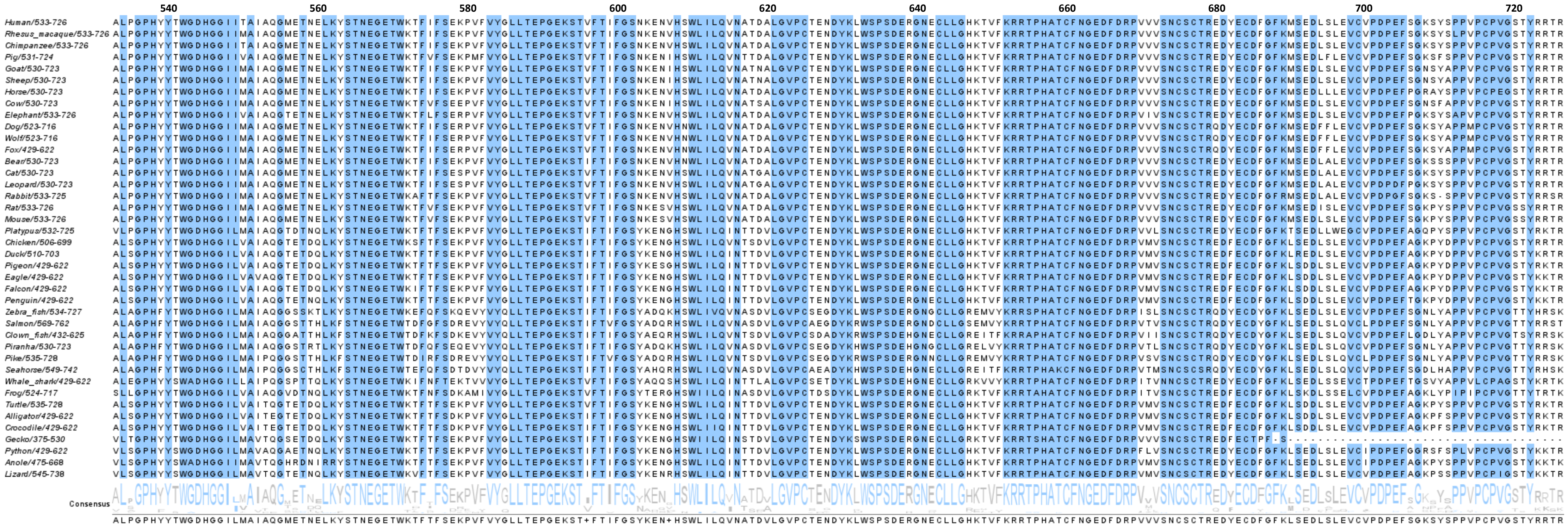

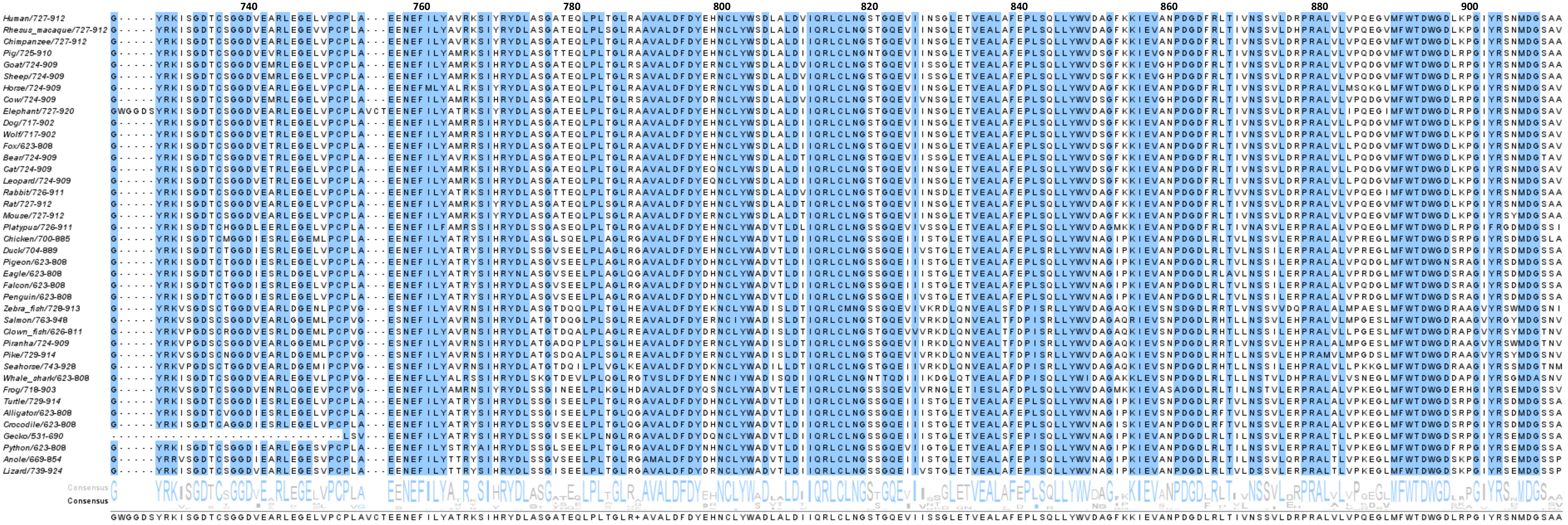
740
760
780
800
820
840
860
880
900
Human/727-912
Rhesus_macaque/727-912
Chimpanzee/727-912
Pig/725-910
Goat/724-909
Sheep/724-909
Horse/724-909
Cow/724-909
Elephant/727-920
Dog/717-902
Wolf/717-902
Fox/623-808
Bear/724-909
Cat/724-909
Leopard/724-909
Rabbit/726-911
Rat/727-912
Mouse/727-912
Platypus/726-911
Chicken/700-885
Duck/704-889
Pigeon/623-808
Eagle/623-808
Falcon/623-808
Penguin/623-808
Zebra_fish/728-913
Salmon/763-948
Clown_fish/626-811
Piranha/724-909
Pike/729-914
Seahorse/743-928
Whale_shark/623-808
Frog/718-903
Turtle/729-914
Alligator/623-808
Crocodile/623-808
Gecko/531-690
Python/623-808
Anole/669-854
Lizard/739-924
Consensus
Consensus

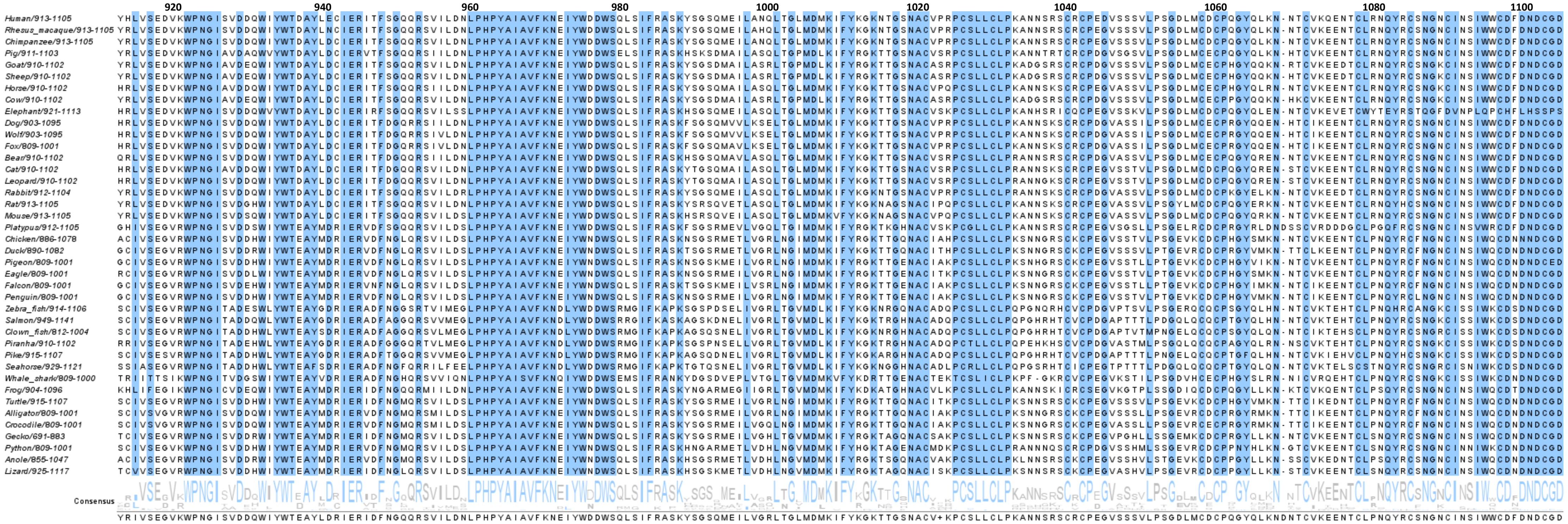
920
940
960
980
1000
1020
1040
1060
1080
1100
Human/913-1105
Rhesus_macaque/913-1105
Chimpanzee/913-1105
Pig/911-1103
Goat/910-1102
Sheep/910-1102
Horse/910-1102
Cow/910-1102
Elephant/921-1113
Dog/903-1095
Wolf/903-1095
Fox/809-1001
Bear/910-1102
Cat/910-1102
Leopard/910-1102
Rabbit/912-1104
Rat/913-1105
Mouse/913-1105
Platypus/912-1105
Chicken/886-1078
Duck/890-1082
Pigeon/809-1001
Eagle/809-1001
Falcon/809-1001
Penguin/809-1001
Zebra_fish/914-1106
Salmon/949-1141
Clown_fish/812-1004
Piranha/910-1102
Pike/915-1107
Seahorse/929-1121
Whale_shark/809-1000
Frog/904-1096
Turtle/915-1107
Alligator/809-1001
Crocodile/809-1001
Gecko/691-883
Python/809-1001
Anole/855-1047
Lizard/925-1117
Consensus

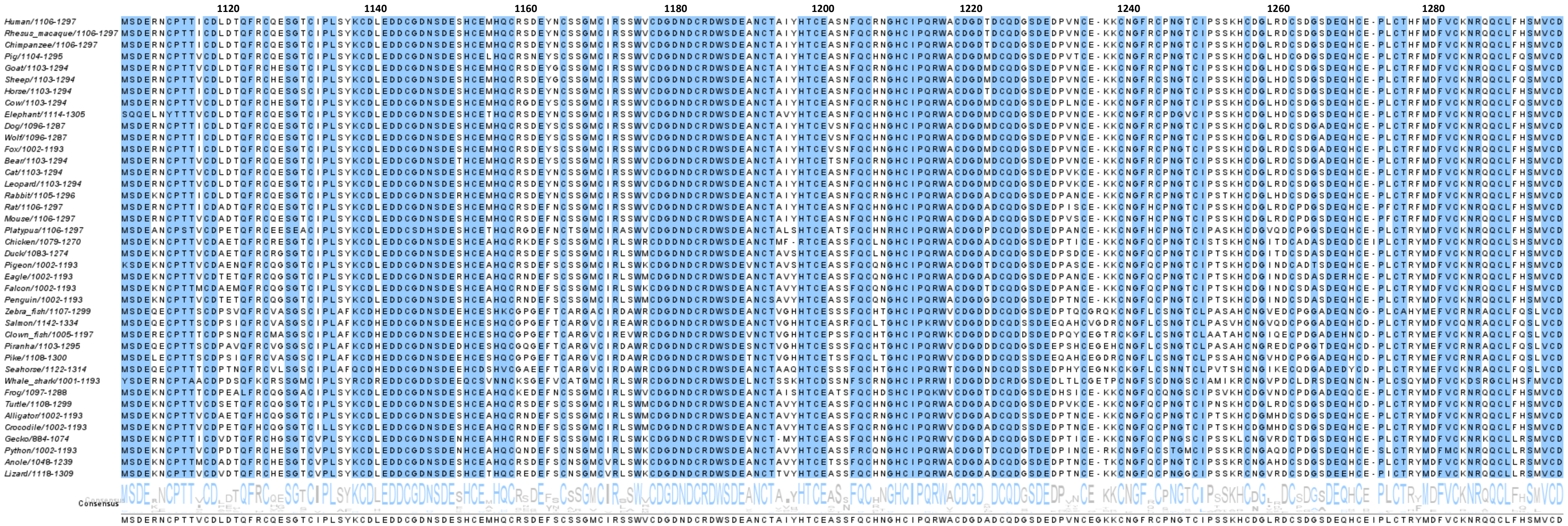
1120
1140
1160
1180
1200
1220
1240
1260
1280
Human/1106-1297
Rhesus_macaque/1106-1297
Chimpanzee/1106-1297
Pig/1104-1295
Goat/1103-1294
Sheep/1103-1294
Horse/1103-1294
Cow/1103-1294
Elephant/1114-1305
Dog/1096-1287
Wolf/1096-1287
Fox/1002-1193
Bear/1103-1294
Cat/1103-1294
Leopard/1103-1294
Rabbit/1105-1296
Rat/1106-1297
Mouse/1106-1297
Platypus/1106-1297
Chicken/1079-1270
Duck/1083-1274
Pigeon/1002-1193
Eagle/1002-1193
Falcon/1002-1193
Penguin/1002-1193
Zebra_fish/1107-1299
Salmon/1142-1334
Clown_fish/1005-1197
Piranha/1103-1295
Pike/1108-1300
Seahorse/1122-1314
Whale_shark/1001-1193
Frog/1097-1288
Turtle/1108-1299
Alligator/1002-1193
Crocodile/1002-1193
Gecko/884-1074
Python/1002-1193
Anole/1048-1239
Lizard/1118-1309
Consensus
MSDERNCPTTVCDLDTQFRCQESGTCIPLSYKCDLEDDCGDNSDESHCEMHQCRSDEFSCSSGMCIRSSWVCDGDNDCRDWSDEANCTAIYHTCEASSFQCHNGHCIPQRWACDGDADCQDGSDEDPVNCEGKKCNGFRCPNGTCIPSSKHCDGLRDCSDGSDEQHCEIPLCTRYMDFVCKNRQQCLFHSMVCD

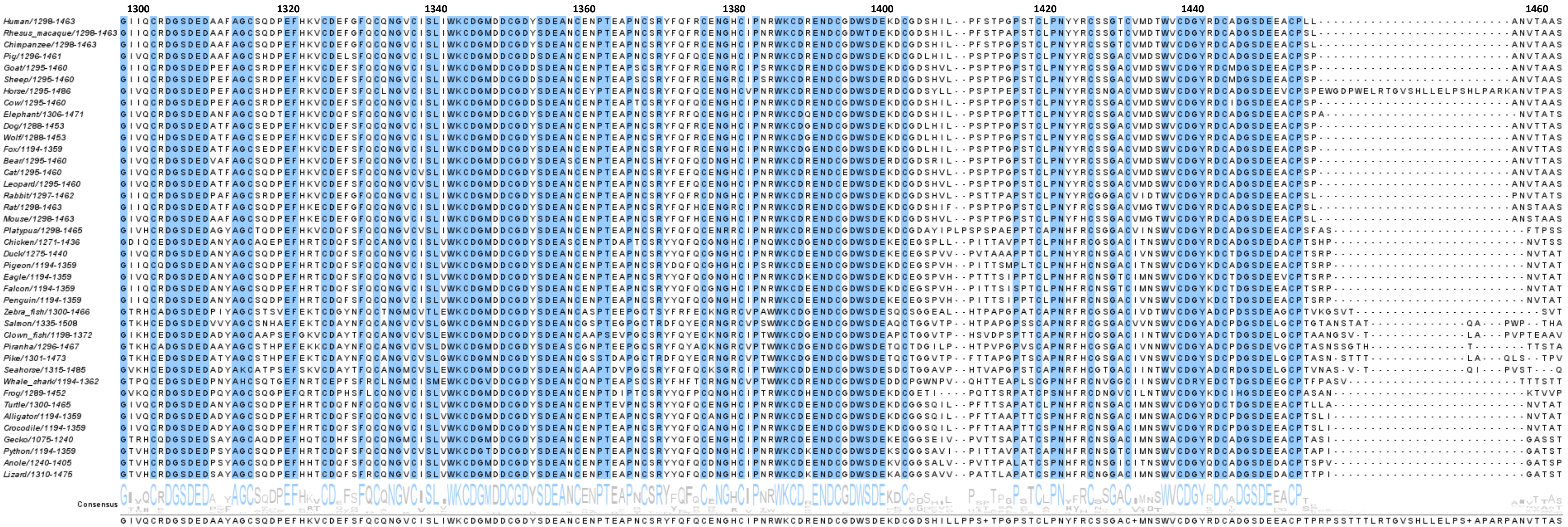
1300
1320
1340
1360
1380
1400
1420
1440
1460
Human/1298-1463
Rhesus_macaque/1298-1463
Chimpanzee/1298-1463
Pig/1296-1461
Goat/1295-1460
Sheep/1295-1460
Horse/1295-1486
Cow/1295-1460
Elephant/1306-1471
Dog/1288-1453
Wolf/1288-1453
Fox/1194-1359
Bear/1295-1460
Cat/1295-1460
Leopard/1295-1460
Rabbit/1297-1462
Rat/1298-1463
Mouse/1298-1463
Platypus/1298-1465
Chicken/1271-1436
Duck/1275-1440
Pigeon/1194-1359
Eagle/1194-1359
Falcon/1194-1359
Penguin/1194-1359
Zebra_fish/1300-1466
Salmon/1335-1508
Clown_fish/1198-1372
Piranha/1296-1467
Pike/1301-1473
Seahorse/1315-1485
Whale_shark/1194-1362
Frog/1289-1452
Turtle/1300-1465
Alligator/1194-1359
Crocodile/1194-1359
Gecko/1075-1240
Python/1194-1359
Anole/1240-1405
Lizard/1310-1475
Consensus

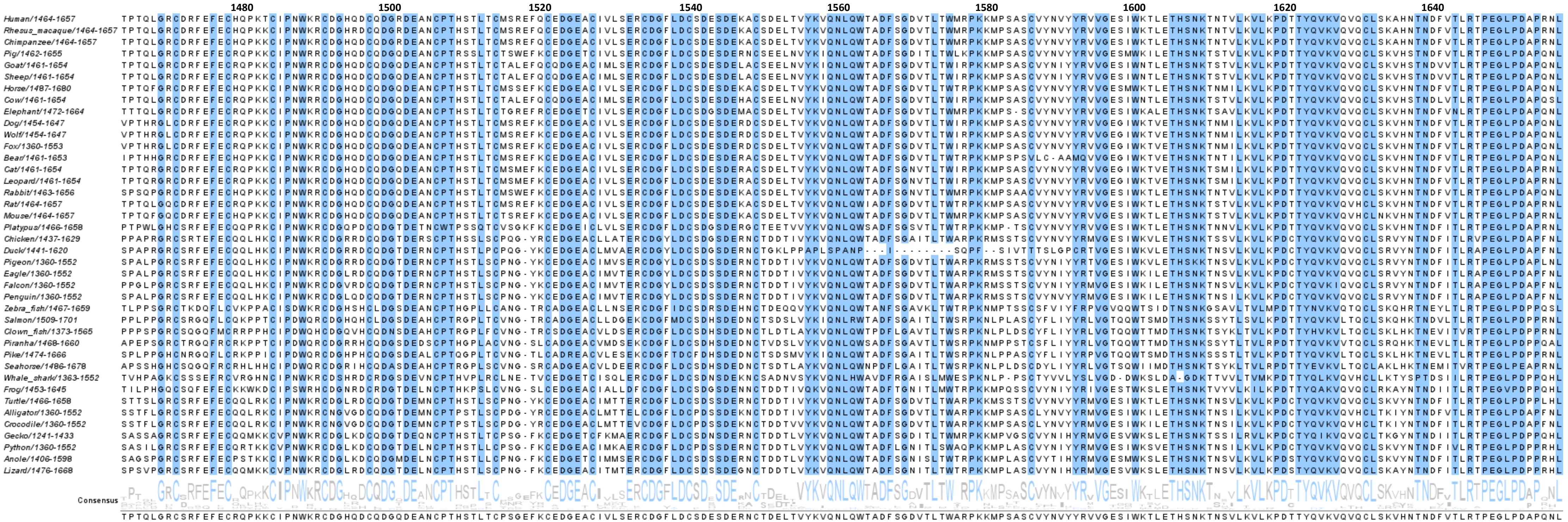
1480
1500
1520
1540
1560
1580
1600
1620
1640
Human/1464-1657
Rhesus_macaque/1464-1657
Chimpanzee/1464-1657
Pig/1462-1655
Goat/1461-1654
Sheep/1461-1654
Horse/1487-1680
Cow/1461-1654
Elephant/1472-1664
Dog/1454-1647
Wolf/1454-1647
Fox/1360-1553
Bear/1461-1653
Cat/1461-1654
Leopard/1461-1654
Rabbit/1463-1656
Rat/1464-1657
Mouse/1464-1657
Platypus/1466-1658
Chicken/1437-1629
Duck/1441-1620
Pigeon/1360-1552
Eagle/1360-1552
Falcon/1360-1552
Penguin/1360-1552
Zebra_fish/1467-1659
Salmon/1509-1701
Clown_fish/1373-1565
Piranha/1468-1660
Pike/1474-1666
Seahorse/1486-1678
Whale_shark/1363-1552
Frog/1453-1645
Turtle/1466-1658
Alligator/1360-1552
Crocodile/1360-1552
Gecko/1241-1433
Python/1360-1552
Anole/1406-1598
Lizard/1476-1668
Consensus

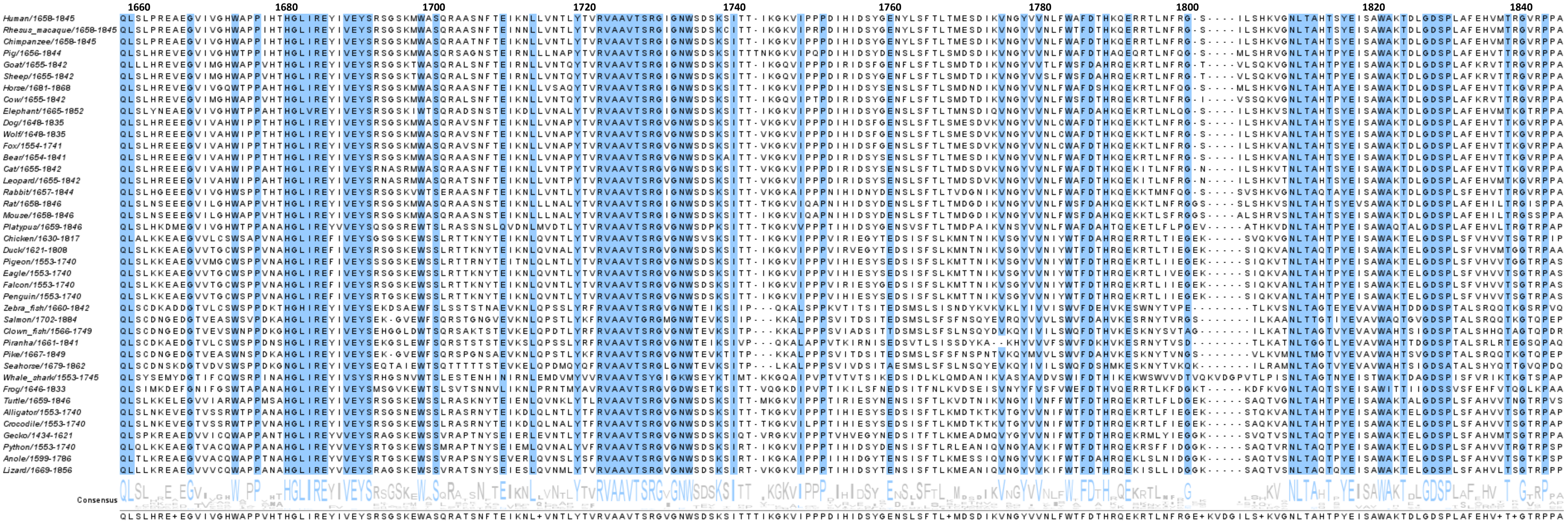
1660
1680
1700
1720
1740
1760
1780
1800
1820
1840
Human/1658-1845
Rhesus_macaque/1658-1845
Chimpanzee/1658-1845
Pig/1656-1844
Goat/1655-1842
Sheep/1655-1842
Horse/1681-1868
Cow/1655-1842
Elephant/1665-1852
Dog/1648-1835
Wolf/1648-1835
Fox/1554-1741
Bear/1654-1841
Cat/1655-1842
Leopard/1655-1842
Rabbit/1657-1844
Rat/1658-1846
Mouse/1658-1846
Platypus/1659-1846
Chicken/1630-1817
Duck/1621-1808
Pigeon/1553-1740
Eagle/1553-1740
Falcon/1553-1740
Penguin/1553-1740
Zebra_fish/1660-1842
Salmon/1702-1884
Clown_fish/1566-1749
Piranha/1661-1841
Pike/1667-1849
Seahorse/1679-1862
Whale_shark/1553-1745
Frog/1646-1833
Turtle/1659-1846
Alligator/1553-1740
Crocodile/1553-1740
Gecko/1434-1621
Python/1553-1740
Anole/1599-1786
Lizard/1669-1856
Consensus

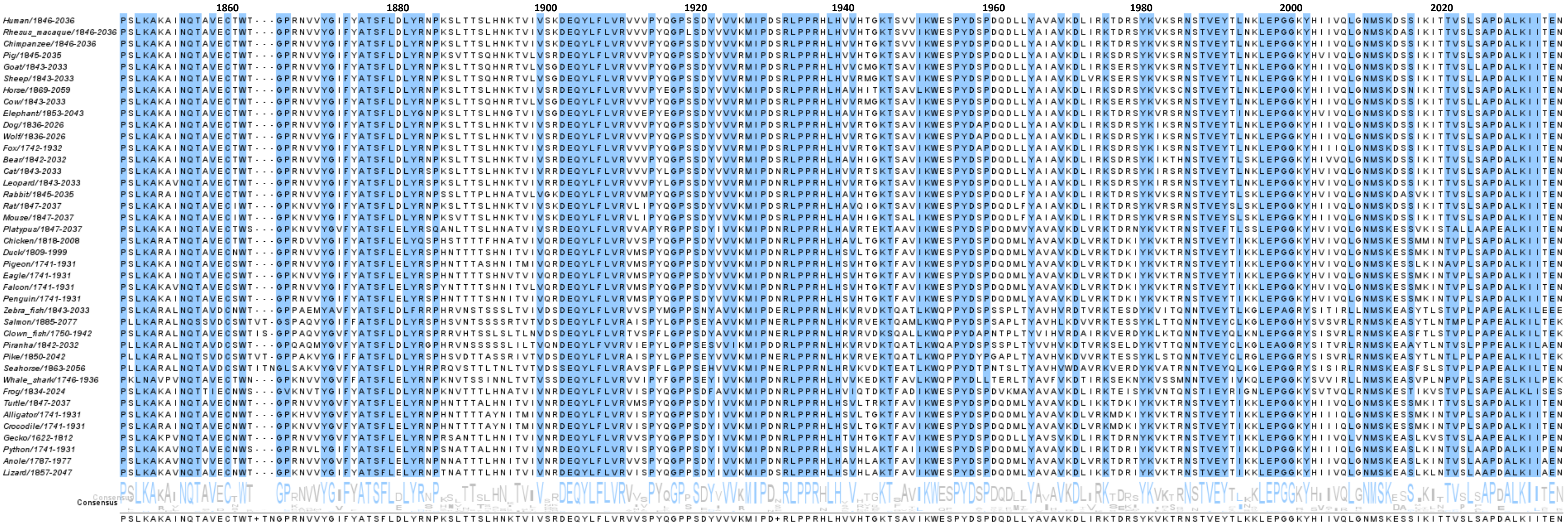
1860
1880
1900
1920
1940
1960
1980
2000
2020
Human/1846-2036
Rhesus_macaque/1846-2036
Chimpanzee/1846-2036
Pig/1845-2035
Goat/1843-2033
Sheep/1843-2033
Horse/1869-2059
Cow/1843-2033
Elephant/1853-2043
Dog/1836-2026
Wolf/1836-2026
Fox/1742-1932
Bear/1842-2032
Cat/1843-2033
Leopard/1843-2033
Rabbit/1845-2035
Rat/1847-2037
Mouse/1847-2037
Platypus/1847-2037
Chicken/1818-2008
Duck/1809-1999
Pigeon/1741-1931
Eagle/1741-1931
Falcon/1741-1931
Penguin/1741-1931
Zebra_fish/1843-2033
Salmon/1885-2077
Clown_fish/1750-1942
Piranha/1842-2032
Pike/1850-2042
Seahorse/1863-2056
Whale_shark/1746-1936
Frog/1834-2024
Turtle/1847-2037
Alligator/1741-1931
Crocodile/1741-1931
Gecko/1622-1812
Python/1741-1931
Anole/1787-1977
Lizard/1857-2047
Consensus
PSLKAKAINQTAVECTWT+TNGPRNVVYGIFYATSFLDLYRNPKSLTTSLHNKTVIVSRDEQYLFLVRVVSPYQGPPSDYVVVKMIPD+RLPPRHLHVVHTGKTSAVIKWESPYDSPDQDLLYAVAVKDLIRKTDRSYKVKTRNSTVEYTLKKLEPGGKYHIIVQLGNMSKESSIKITTVSLSAPDALKIITEN

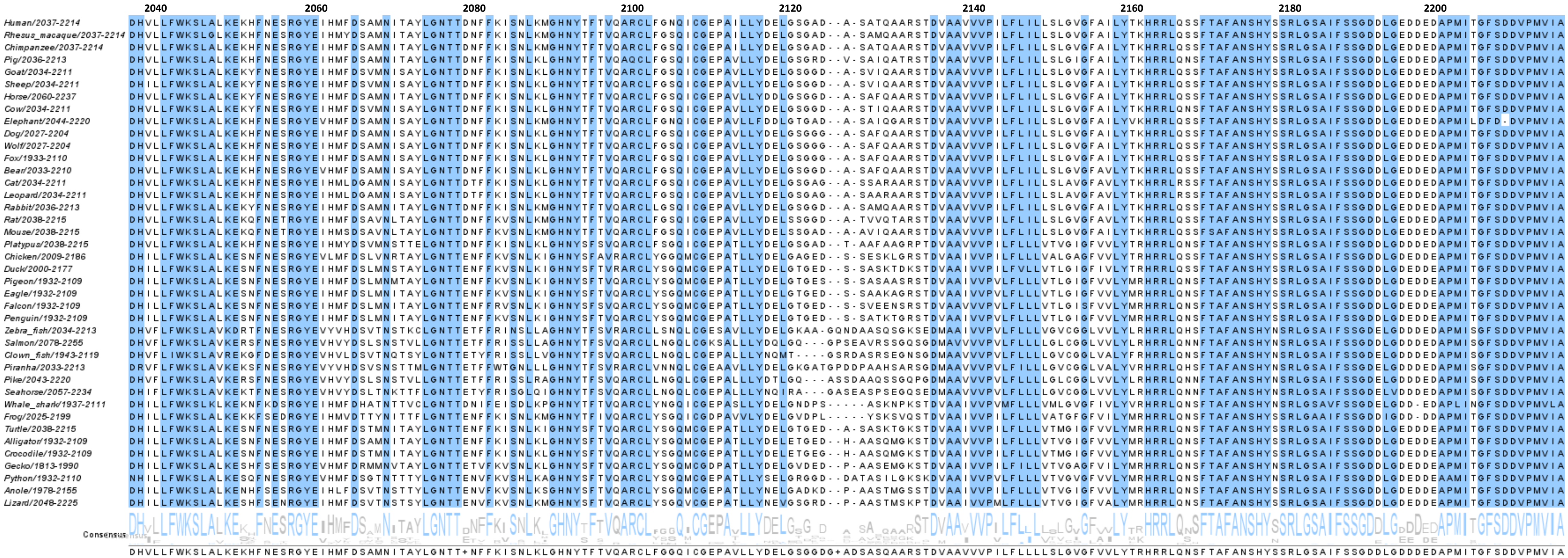

2040
2060
2080
2100
2120
2140
2160
2180
2200
Human/2037-2214
Rhesus_macaque/2037-2214
Chimpanzee/2037-2214
Pig/2036-2213
Goat/2034-2211
Sheep/2034-2211
Horse/2060-2237
Cow/2034-2211
Elephant/2044-2220
Dog/2027-2204
Wolf/2027-2204
Fox/1933-2110
Bear/2033-2210
Cat/2034-2211
Leopard/2034-2211
Rabbit/2036-2213
Rat/2038-2215
Mouse/2038-2215
Platypus/2038-2215
Chicken/2009-2186
Duck/2000-2177
Pigeon/1932-2109
Eagle/1932-2109
Falcon/1932-2109
Penguin/1932-2109
Zebra_fish/2034-2213
Salmon/2078-2255
Clown_fish/1943-2119
Piranha/2033-2213
Pike/2043-2220
Seahorse/2057-2234
Whale_shark/1937-2111
Frog/2025-2199
Turtle/2038-2215
Alligator/1932-2109
Crocodile/1932-2109
Gecko/1813-1990
Python/1932-2110
Anole/1978-2155
Lizard/2048-2225
Consensus

3.9 Phylogenetic tree for SORL1

Andersen
Supplemental Figure S13 - Phylogenetics

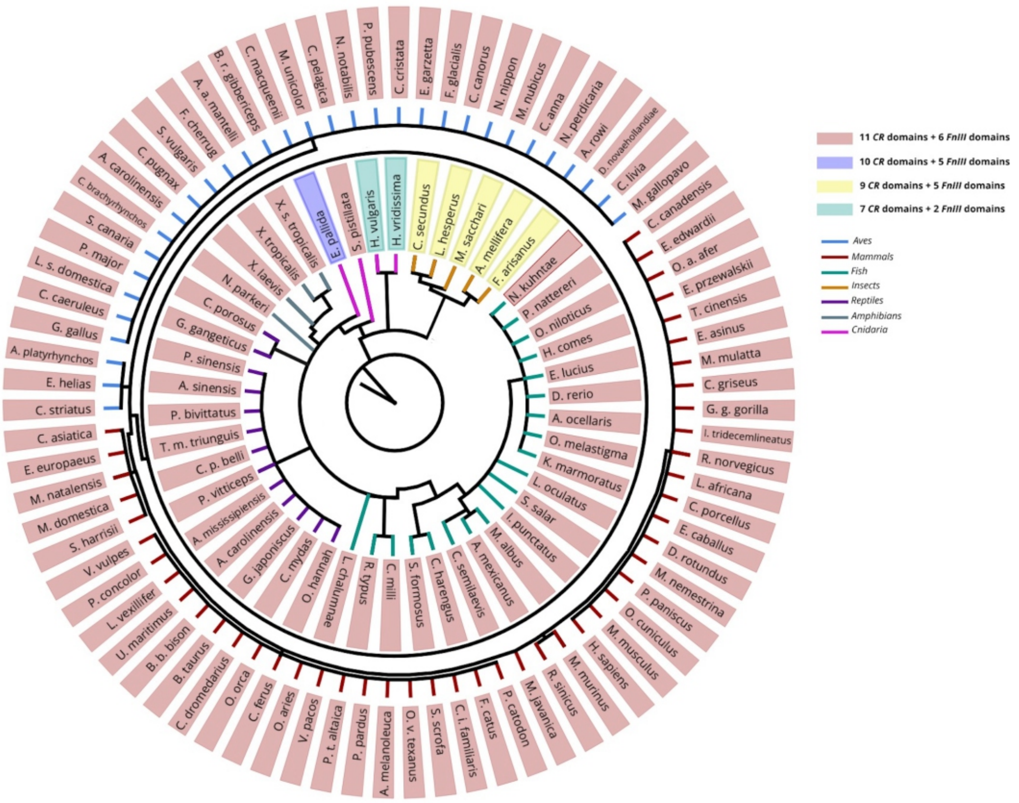

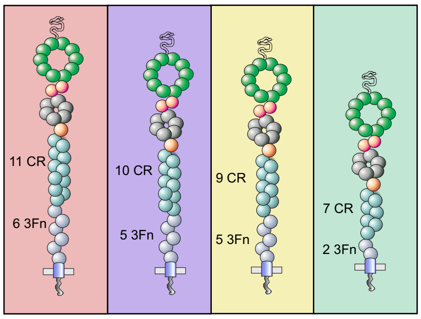

3.9.1 Supplemental Figure S13. Phylogenetic tree

Phylogenetic tree of 126 analyzed SORL1 sequences available from the NCBI website, showing how most receptors contain 11 CR-domains and 6 3Fn-domains (pink), but also isoforms with only 10 CR-domains and 5 3Fn-domains (blue), 9 CR-domains and 5 3Fn-domains (yellow), or 7 CR-domains and 2 3Fn-domains exist. Domain compositions are summarized by schematics at the bottom following same color scheme as above.

3.10 Supplemental methods

3.10.1 SORL1 DOMAIN SEQUENCE ALIGNMENTS

Manual alignment of the SORL1 sequence was performed under the listed considerations:

VPS10p: Alignment of the 10 internally repeated sequences is based on β-blade boundaries and identified β-strand sequences as defined by the solved crystal structure in its uncomplexed form [37]. There is only a limited conservation across the 10 sequences, so alignment is primarily done focusing on positions containing amino acid residues with hydrophobic side chains.

YWTD: Alignment of the 6 internally repeated sequences is based on β-blade boundaries of domains from the homologous LDLR domain [47] and guided by its β-strand sequences and conservation of a number of hydrophobic residues. Gaps in the alignment are preferentially assigned to positions in loop regions between β-strands. The sequences of YWTD-domains from SORL1 and 14 other domains from proteins from the LDLR family are presented in **Supplemental Information 3.2.4**.

EGF: The single eight-cysteines SORL1 domain of the EGF-type is aligned either with 15 EGF-domain sequences from the LDLR family (selected from domains located C-terminal to YWTD-domains) or with 8 EGF-domain sequences from the integrins (**Supplemental Information 3.3.4**).

CR: Alignment of the 11 individual CR-domain sequences from SORL1 is done according to domain boundaries as defined by the genomic exon sequences. These domains sequences were also aligned with an additional 32 sequences from proteins containing pathogenic variants (**Supplemental Information 3.4.4**)

3Fn: Alignment of the 6 individual 3Fn-domain sequences is done based on a secondary structure prediction to identify the suggested position of 42 β-strands together with the presence of 4 highly conserved residues at domain positions 25 (W, strand B), 41 (Y, strand C), 77 (L, EF-loop), and 83 (Y, strand F) and allowing loops to accommodate most gaps. The SORL1 domains were aligned with 3Fn-sequences from proteins containing disease-mutations in their 3Fn-domains, allowing the alignment to take into account also the partial conservation at positions 6, 7, 11, 13, 72, 74 and 94 (**Supplemental Information 3.5.4**).

TM/CD: This part of the human SORL1 sequence was aligned with 14 SORL1 sequences from mammalian and less related species.

3.10.2 SORL1-SPECIFIC DISEASE-MUTATION DOMAIN-MAPPING

The domain-mapping of disease-mutations (DMDM) approach displays an aggregate view of human pathogenic mutations by its position in a protein domain [259]. This tool requires the ability to accurately assign the correct position of an observed variant within a domain sequence, and thus requires highly accurate alignments as here performed for the SORL1 domains.

The VPS10- and EGF-domain sequences as well as the transmembrane and tail sequences were not included in the analysis: the VPS10p family is under-investigated, and there are no disease-associated variants firmly established for the other four family members (sortilin, SorCS1, SorCS2, SorCS3) (**Fig. 2**). However, the VPS10p-domain is the only domain in SORL1 for which the structure has been determined [37], such that *SORL1* variants with unknown significance in the VPS10p-domain may be assessed based on the crystal structure to determine their impact on conformation folding/stability/energy. The EGF-domain in SORL1 is longer than the typical ~40 amino acid EGF-domain, and thus alignments to other domains is not accurate and a disease mapping approach could therefore not be applied for this SORL1 domain. The transmembrane and tail sequences are considered SORL1 specific and has no direct comparable sequences in other proteins.

In contrast, we were able to apply the disease-mutation domain-mapping tool to SORL1 domains having strong sequence consensus with homologous domains in other proteins such as the YWTD- CR-, and 3Fn-domains (comprising almost 2/3 of the entire receptor). To limit variants to a manageable number, we included sequences from **human** proteins in Uniprot.org, and manually curated a comprehensive overview of disease-associated variants using information provided by "*Natural variants*" involved in diseases (i.e., listed under "*Pathology & Biotech*" in sequences. **YWTD:** The sequence corresponding to the YWTD-domain is termed "LDL-receptor class B" by Uniprot and annotated as PS51120 (LDLRB) in PROSITE. In this database 14 of the 67 proteins with this domain type are human, of which 6 proteins included a total of 102 unique disease-associated variants that could be mapped (in red) onto the YWTD-domain sequence (**Supplemental Information 3.2.4 and 3.2.5**). **CR**: The CR-domains are annotated as "LDL-receptor class A" in Uniprot and by PS50068 (LDLRA) in PROSITE. In the Uniprot database, 44 of the 168 proteins with CR-domains are human, of which 8 proteins contained at least one disease-associated variant within their CR-domain(s). This enabled us to map (in red) 63 different variants to the CR-domain sequences (**Supplemental Information 3.4.4 and 3.4.5**). **3Fn**: These domains are reported by Uniprot as "Fibronectin type-III" and correspond to PS50853 (FN3) in PROSITE that list as many as

804 known Eukaryotic proteins of which 194 are human. In 35 of these, we identified naturally occurring disease-associated variants enabling us to map 222 unique disease-associated variants (in red) across the 3Fn-domain sequence (**Supplemental Information 3.5.4 and 3.5.5**). Due to limited sequence conservation and variable number of amino acids between positions 50-71 of many 3Fn-domain sequences, variants that mapped to this part of 3Fn-domains were not included in the DMDM analysis.
A few additional variants that are not yet included in the Uniprot database at the time of the analysis were added for pathogenic variants identified from literature.

Finally, the number of disease-variants identified were plotted for each position within these three domain sequences, allowing the unambiguous identification of domain positions most likely to contain pathogenic variants when mutated in SORL1 (**Supplemental Figures S8d, S10d, and S11e**).

From the domain sequence alignments that established residues important for domain folding/stability (based on requirement of sequence conservation) in combination with the DMDM analysis informing on the prevalence of disease-associated mutations at given domain positions, we next generated a list/filter for the entire SORL1 protein highlighting positions that we believe is of '*high priority*' or '*moderate priority'* risk for developing AD. This filter can be applied for interrogations of larger case/control dataset or in a simpler manner by predicting whether single variants correspond to a dangerous position (**Fig 4, Table S4 and S5)**.

3.10.3 PHYLOGENETIC TREE GENERATION:

To create a comprehensive phylogenetic tree, the SORL1 amino acid sequences from 126 of the >300 different species with known SORL1 protein sequences were obtained from the NCBI website. Sequences were selected based on (1) having a sequence length of >1,000 aa, (2) obtaining a maximally diverse selection of animals within each species subclass (mammals, birds, fish, reptiles, insects, amphibians and cnidaria). Firstly, a multiple sequence alignment was created using the ClustallW algorithm [260,261] and the phylogenetic tree was constructed based on this alignment with the Maximum Likelihood method using the MEGA7 software [262] to create a rooted tree. Subsequently, the phylogenetic tree was prepared and edited using GravitDesigner (Corel Corporation, Alludo).

3.10.4 CONSERVATION ANALYSIS: ALIGNMENT OF 40 REPRESENTATIVE SORL1 SEQUENCES:

To determine semi-quantitative conservation of any residue within the human SORL1 sequence across species, we aligned 40 representative sequences (**Supplemental Information 3.8**). The amino acid sequences were obtained from the NCBI website and an alignment was created using the ClustalIW algorithm [260,261]. Subsequently, the alignment was edited using Jalview software [263] to visualize consensus sequence and to highlight amino acids with 100% conservation (blue). This alignment reveals whether an introduced amino acid by novel *SORL1* genetic variants is present in SORL1 from other species.

3.11 Putting the pieces together - the conformational space

Elongated or compact - Flexible or rigid structures?

Thus far, all attempts to solve the structure of the full SORL1 extracellular region have failed. Therefore, it is currently unknown how all the presented domains of the full-length SORL1 protein fold relative to each other. In accordance with the different SORL1 functions, the protein structure may also adopt different conformations. SORL1 engages in the binding and sorting of cargo through cellular compartments, each with distinct pH levels and capacity to modify post-translationally attached *N*-glycosylations and perhaps phosphorylations [194].

Schematic illustrations of the SORL1 molecule often show each domain lined up one after the other, extending to a predicted ~700 Å (=70 nm) (**Supplemental Figure S14a**). Such a linear conformation may be relevant at the plasma membrane if SORL1 uses its VPS10p-domain to scavenge extracellular ligands. Or, since the receptor can locate to the postsynapse, the elongated SORL1-conformation could reach out across the synaptic cleft to binding partners at the presynapse. However, when SORL1 is located in tubular extensions of the endosome (described to have a diameter of only 20-50 nm [264]), it is highly unlikely that SORL1 will adopt this conformation [243]. Here, SORL1 may adopt one or more compact conformations, similar to what has been found for LDLR and integrins [265-267].

The 3Fn-domain region may behave as a single compact structure, connected with the two β-propellers by the flexible CR-cluster. The region of the 3Fn-domains may be described *as a single and rather solid unit* [268]. In many receptor proteins the consecutive 3Fn-domains have a "*rod-like*" structure, with an angle between two neighboring 3Fn-domains close to 180 degrees: if all six 3Fn-domains of SORL1 adopt this rod-like structure, the total structure would span ~210 Å. Two neighboring 3Fn-domains can also "*tilt*" with an angle typically around 120 degrees [186]: if all six SORL1 3Fn-domains adopt this "*wiggling*" structure they span only ~140 Å (**Supplemental Figure S14b**).

It is still unclear whether the two β-propeller domains in SORL1 have a fixed or flexible orientation relative to one another. The two adjacent β-propeller domains may behave as a combined rigid structural block, which would be in accordance with the rigid connection between tandem β-propellers observed in LRP5 and LRP6. In this scenario the 5 amino acids (^{753}PLAEE^{757}) around the 10CC-YWTD linker that separates the two propellers, would not function as a hinge. Nevertheless, the 10CC-domain position relative to the VPS10p-domain may change with pH as described [37], arguing that this model may be over-simplified.

In contrast, the sequence directly following the EGF-domain of SORL1 may act as a first hinge region (**H1, Supplemental Figure S14b**), paralleling the EGF-domain of integrin (also of the 8 Cys-type) that act as a hinge region between larger rigid units [95]. The site between the end of the CR-cluster and beginning of the 3Fn-domains of SORL1 may serve as a second hinge region (**H2**), similar to the hinge region in LDLR that is found after its CR-cluster [134,265,269]. Interestingly, this second hinge would then locate next to the extremely long linkers in the SORL1 CR-cluster and allow for substantial structural flexibility for this region. These two hinge regions might bring the receptor from a very "*elongated*" fold toward a more "*compact*" form (**Supplemental Figure S14b; Fig. 1**). With such a flexible CR-cluster it is unclear whether the YWTD- or the VPS10p-domain propeller is closest to the cell membrane (**Supplemental Figure S14**). However, most VPS10p-domain receptors have the VPS10p-domain close to the membrane (**Fig. 2**), suggesting that this a preferred position that may relate to shared functionalities.

Monomer, dimer – or polymer

The ability of SORL1 to bind, traffic, and release cargo may depend on its flexibility but also on possible dimer formation. SORCS1 and SORCS2 exist mainly as a dimer; when the PKD-domains C-terminal to their VPS10p-domains interact, the top-face of their β-propellers are available for ligand binding, important for cell surface signaling [270,271].

When sortilin is internalized in endosomes it dimerizes upon the pH-drop, leading to ligand-release [272-274]. With the drop in pH, the side chains of a stretch of His residues on the top face of the VPS10p-domain β-propeller become protonated, leading to a conformational change of the VPS10p-domain loops, allowing dimerization at the top faces between two propeller-domains, enabling ligand displacement. It is unlikely that identical dimerization mechanisms occur for the SORL1 VPS10p-domain because the SORL1 sequence does not include any PKD-domains that drive dimer formation of SORCS1 and SORCS2. Neither does the SORL1 VPS10p-domain sequence hold the His residues responsible for pH-dependent rearrangements in sortilin, nor the residues in the loops important for the direct contacts between the two VPS10p-domains [273,275]

However, nearly all known receptor-like molecules containing 3Fn-domains have the capacity to form dimers [180], and also SORL1 dimerization can be mediated by its 3Fn-domains [56] (**Supplemental Figure S14**). Similar to SORL1, the extracellular region of growth-hormone-, prolactin-, and insulin receptors have 3Fn-domains that are located close to the plasma membrane and their activity is crucially associated with dimer conformation in the presence of bound ligands. The dimerization by 3Fn-domains of Tie1 and Tie2 receptors, from the tyrosine kinase family, is crucial for its activation [276]. While more studies are necessary to explore the

physiological role of 3Fn-domain-mediated SORL1-dimerization, it is interesting to notice that several key ligands of SORL1 (e.g. BDNF, APP, TrkB, HER2/3, GLUA1) exist in equilibrium between mono- and dimeric structures [277-279]. In contrast to the other sortilins, that only contain a single site of dimerization, the ability of a SORL1 protein to bind another SORL1 protein by either it's VPS10p-domain or its 3Fn-domains, suggest that also larger polymers of SORL1 receptors may form [56] (**Supplemental Figure S14).**

While members of the LDLR family are mainly monomers, they also release ligands upon endosome internalization [134,266]. Here, the pH drop in endosomes leads to protonation of several histidine residues of the LDLR YWTD β-propeller. At the cell surface, LDLR has an elongated conformation and ligands bind to the CR-domains. Upon entering the endosome, the receptor adopts a more compact form: the β-propeller becomes protonated, and serves an alternative binding partner for the CR-domains which leads to displacement of the internalized ligand [265,280]. The released ligand is free for transport to lysosomal degradation while the receptor —in its closed conformation— is recycled through the acidic environments back to the cell surface, where it may once again adopt its open conformation, allowing CR-domains to interact with new ligands [281]. In contrast with LDLR, several cargo ligands for SORL1 (incl. APP and HER2) have the highest affinity for SORL1-binding at *low* pH [102,282], allowing SORL1 to escort ligands *out* of the endosome to either the TGN or to the cell surface [279,282] This requires efficient ligand binding *within* the endosome. Notably, the SORL1 YWTD β-propeller sequence does *not* contain the His residues necessary for the pH-triggered release of ligands from the CR-domains [55]

Support of "hidden" disulfides

Based on the presented domain boundaries as defined by sequence alignments and expected domain folds, we present a 'compact' schematic diagram of SORL1, including all its 2,214 amino acids while taking into account the suggested presence of disulfides and β-strand secondary structures (**Fig. 1**). This diagram provides support for the presence of three disulfides that were not yet identified: (1) a disulfide between C^{801} and C^{816} at the β2-blade of the YWTD-domain, (2) a disulfide between C^{1586} and C^{1631} in the first 3Fn-domain, and (3) a disulfide between C^{2101} and C^{2108} in the sixth 3Fn-domain. (**Fig. 1**). In particular, the second suggested disulfide is separated by 45 amino acids in the primary structure, which can be appreciated only when taking the conserved topology of the 7-stranded 3Fn-domain scaffold into consideration. Both cysteine residues are predicted to point their side chain towards the hydrophobic interior of the 3Fn-domain β-blade sandwich as they occur "in-frame" with the alternating hydrophobic residues (see **Supplemental Figure S11**).

While many of these considerations about SORL1 conformation await experimental validation, they may contribute to a better understanding of the nature of variant pathogenicity: SORL1 folding, and thus activity, may not be compromised due to misfolding of single domains only, but also by damaging intramolecular interactions affecting bent and/or dimer/polymer conformations. We propose that next to variant effect prediction algorithms, the potential damagingness of genetic variants should be assessed based on features unique for SORL1: their position in the 3D alignments, and whether that position associates with disease in homologous proteins.

Andersen
Supplemental Figure S14- conformational space

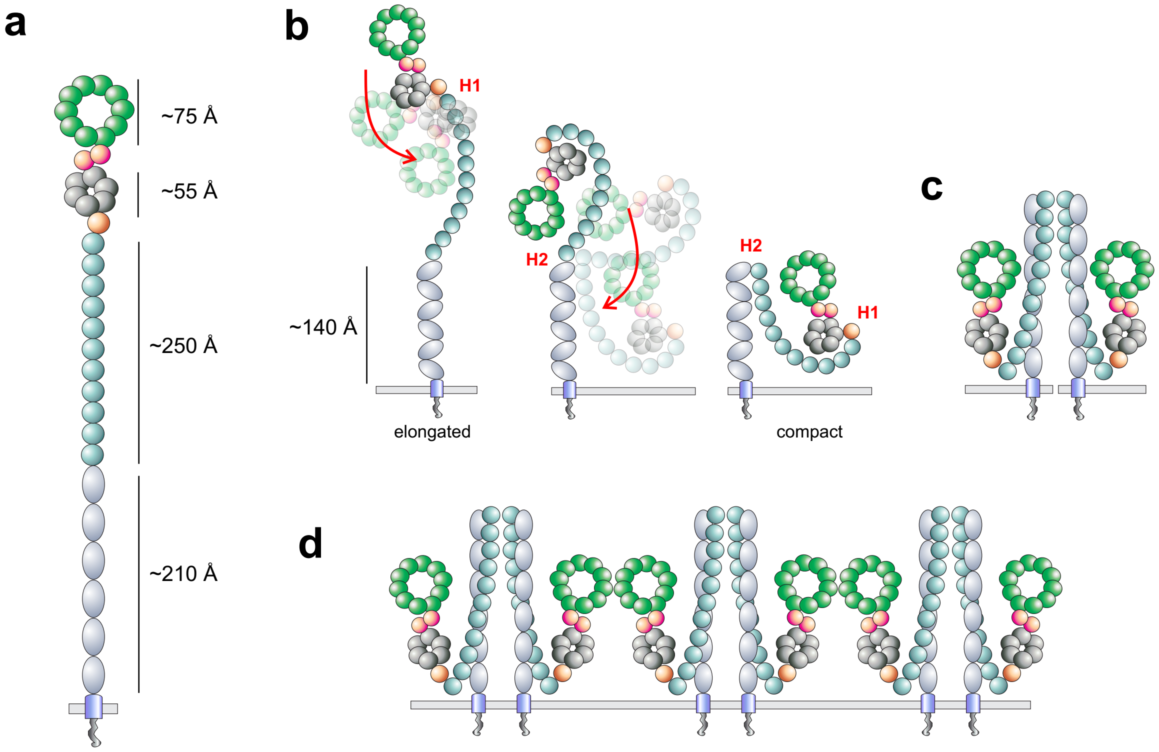

3.12 Supplemental Figure S14. The SORL1 conformational space

a. Schematic of SORL1 in an elongated conformation, spanning as much as ~700 Å if fully stretched, with indication of maximal expected size of the different receptor regions.

b. The speculated flexibility of the CR-cluster flanked by two potential hinge regions (H1 and H2) may contribute to several possible conformations, going from elongated to more compacts form. A tilt conformation between neighboring 3Fn-domains (angel set to 120) is indicated compared to the elongated possible conformation depicted in panel a.

c. Possible bend conformations with dimerization driven by the 3Fn-domains

d. Possible polymer formation of SORL1 receptors due to the presence of sites in both the VPS10p- and 3Fn-domains that can facilitate dimerization and thus potential building of larger polymeric structures as previously suggested [56].

4 ACKNOWLEDGMENTS

4.1 Study participants, personnel, and compute infrastructure

4.1.1 Participants

The authors are grateful to the study participants, their family members, and the participating general practitioners, pharmacists and all laboratory personnel involved in blood collection, DNA isolation, and DNA biobanking.

4.1.2 SURF supercomputer facility

The work in this manuscript was carried out on the Cartesius supercomputer, which is embedded in the Dutch national e-infrastructure with the support of SURF Cooperative. Computing hours were granted in 2016, 2017, 2018 and 2019 to H. Holstege by the Dutch Research Council (project name: ‘100plus’; project numbers 15318 and 17232).

4.2 Study Cohorts

4.2.1 ADES-FR

This study was funded by grants from the Clinical Research Hospital Program from the French Ministry of Health (GMAJ, PHRC, 2008/067), the CNR-MAJ, the JPND PERADES, Equipe FRM DEQ20170336711, and Fondation Alzheimer (ECASCAD study). This research was supported by the Laboratory of Excellence GENMED (Medical Genomics) grant no. ANR-10-LABX-0013 managed by the National Research Agency (ANR) part of the Investment for the Future program. This work was also supported by Foundation Alzheimer, the Institut Pasteur de Lille, Inserm, the Haut-de-France and Lille Métropole Communauté Urbaine council, and the French government's LABEX (laboratory of excellence program investment for the future) DISTALZ grant (Development of Innovative Strategies for a Transdisciplinary approach to Alzheimer's disease). The 3C Study supports are listed on the Study Website (www.three-city-study.com).

4.2.2 AgeCoDe-UKBonn

The AgeCoDe cohort was funded in part by the German Federal Ministry of Education and Research (BMBF) (grants KNDD 01GI0710, 01GI0711, 01GI0712, 01GI0713, 01GI0714, 01GI0715, 01GI0716, 01ET1006B). Sequencing of AgeCoDe sample was in part funded by the German Research Foundation (DFG) grant RA 1971/6-1 to Alfredo Ramirez.

4.2.3 Barcelona- SPIN

Support for Jordi Clarimon provided by Maratón RTVE (Spain).Support for Oriol Dols provided by the Association for Frontotemporal Degeneration (Clinical Research Postdoctoral Fellowship, AFTD).

4.2.4 AC-EMC

Exome sequencing was funded by Alzheimer Nederland.

4.2.5 ERF

The ERF study as a part of EUROSPAN (European Special Populations Research Network) was supported by European Commission FP6 STRP grant number 018947 (LSHG-CT-2006-01947) and also received funding from the European Community's Seventh Framework Programme (FP7/2007-2013)/grant agreement HEALTH-F4- 2007-201413 by the European Commission under the programme "Quality of Life and Management of the Living Resources" of 5th Framework Programme (no. QLG2-CT-2002-01254). High-throughput analysis of the ERF data was supported by a joint grant from the Netherlands Organization for Scientific Research and the Russian Foundation for Basic Research (NWO-RFBR 047.017.043).

4.2.6 Rotterdam Study

The generation and management of the exome sequencing data for the Rotterdam Study was executed by the Human Genotyping Facility of the Genetic Laboratory of the Department of Internal Medicine, Erasmus MC, the Netherlands. The Rotterdam Study is funded by Erasmus Medical Center and Erasmus University, Rotterdam, the Netherlands Organization for Health Research and Development (ZonMw), the Research Institute for

Diseases in the Elderly (RIDE) (014-93-015; RIDE2), the Ministry of Education, Culture and Science, the Ministry for Health, Welfare and Sports, the European Commission (DG XII), and the municipality of Rotterdam. Genetic data sets are also supported by the Netherlands Organization of Scientific Research NWO Investments (175.010.2005.011, 911-03-012), the Genetic Laboratory of the Department of Internal Medicine, Erasmus MC, and the Netherlands Genomics Initiative (NGI)/Netherlands Organization for Scientific Research (NWO), the Netherlands Consortium for Healthy Aging (NCHA), project 050-060-810, and by a Complementation Project of the Biobanking and Biomolecular Research Infrastructure Netherlands (BBMRI-NL; www.bbmri.nl ; project number CP2010-41). We thank Mr. Pascal Arp, Ms. Mila Jhamai, Mr. and Marijn Verkerk, for their help in creating the RS-Exome Sequencing database.

4.2.7 ADC-Amsterdam

We thank all study participants and all personnel involved in data collection for the contributing studies. Research of Alzheimer center Amsterdam is part of the neurodegeneration research program of Amsterdam Neuroscience. Alzheimer Center Amsterdam is supported by Stichting Alzheimer Nederland and Stichting VUmc fonds. The clinical database structure was developed with funding from Stichting Dioraphte. This work was supported by Stichting Alzheimer Nederland (WE.09-2014-06, WE.05-2010-06); Stichting Dioraphte; Internationale Stichting Alzheimer Onderzoek (#11519); JPND-PERADES (ZonMw 733051022): Centralized Facility for Sequence to Phenotype analyses (ZonMW 9111025); Netherlands Consortium for Healthy Aging (NCHA 050-060-810); Biobanking and Biomolecular Research Infrastructure Netherlands (BBMRI-NL CP2010-41); Netherlands Genomics Initiative (NGI)/NWO. This study is further supported by ABOARD, a public-private partnership receiving funding from ZonMW (#73305095007) and Health~Holland, Topsector Life Sciences & Health (PPP-allowance; #LSHM20106). This research is performed by using data from the Parelsnoer Institute an initiative of the Dutch Federation of University Medical Centres (www.parelsnoer.org).

4.2.8 100-plus Study

Cohort collection and exome sequencing of the 100-plus Study cohort was supported by Stichting Alzheimer Nederland (WE.09-2014-03); HorstingStuit Foundation, VUmc Foundation, and the Dioraphte Foundation (Project 17020403), Memorabel (ZonMW project number #733050814, #733050512) and Stichting VUmcFonds. Additional support is from ABOARD, a public-private partnership receiving funding from ZonMW (#73305095007) and Health~Holland, Topsector Life Sciences & Health (PPP-allowance; #LSHM20106).

4.2.9 EMIF-AD 90+

The EMIF-AD 90+ Study was funded by the EU/EFPIA Innovative Medicines Initiative Joint Undertaking EMIF grant agreement no. 115372.

4.2.10 CBC: Control Brain Consortium

This work was supported by the UK Dementia Research Institute which receives its funding from DRI Ltd, funded by the UK Medical Research Council, Alzheimer's Society and Alzheimer's Research UK, Medical Research Council (award number MR/N026004/1). Wellcome Trust Hardy (award number 202903/Z/16/Z), Dolby Family Fund; National Institute for Health Research University College London Hospitals Biomedical Research Centre; BRCNIHR Biomedical Research Centre at University College London Hospitals NHS Foundation Trust and University College London.

J. Hardy was supported by the Dolby Foundation and the JPND PERADES. J.B. and R.G were supported by the National Institute on Aging of the National Institutes of Health under Award Number R01AG067426. The content is solely the responsibility of the authors and does not necessarily represent the official views of the National Institutes of Health.

4.2.11 PERADES

We thank all individuals who participated in the study. We also want to express our gratitude to the MRC Centre Core Team for the laboratory support and the Advanced Research Computing at Cardiff University (ARCCA) for the computational support. Cardiff

University was supported by the Medical Research Council. Cardiff University was also supported by the European Joint Programme for Neurodegenerative Disease, Alzheimer's Research UK, the Welsh Assembly Government, and a donation from the Moondance Charitable Foundation. Cardiff University acknowledges the support of the UK Dementia Research Institute, which receives its funding from UK DRI Ltd, funded by the UK Medical Research Council, Alzheimer's Society and Alzheimer's Research UK. Cambridge University acknowledges support from the MRC. The University of Southampton acknowledges support from the Alzheimer’s Society. ARUK provided support to Nottingham University. Join Dementia Research (JDR) is funded by the Department of Health and delivered by the National Institute for Health Research in partnership with Alzheimer Scotland, Alzheimer's Research UK and Alzheimer's Society. IRCCS Santa Lucia Foundation acknowledges the Italian Ministry of Health for financial support (IMH_RC) of this study. The Centro de Biologia de Molecular Severo Ochoa (CSIS-UAM), CIBERNED, Instituto de Investigacion Sanitaria la Paz, University Hospital La Paz and the Universidad Autonoma de Madrid were supported by grants from the Ministerio de Educacion y Ciencia and the Ministerio de Sanidad y Consumo (Instituto de Salud Carlos III), and an institutional grant of the Fundacion Ramon Areces to the CMBSO. Thanks to I. Sastre and Dr A Martinez-Garcia for DNA preparation, and Drs P Gil and P Coria for their recruitment efforts. Department of Neurology, University Hospital Mutua de Terrassa, Terrassa, Barcelona, Spain was supported by CIBERNED, Centro de Investigacion Biomedica en Red de Enfermedades Neurodegenerativas, Instituto de Salud Carlos III, Madrid Spain and acknowledges Maria A Pastor (Department of Neurology, University of Navarra Medical School and Neuroimaging Laboratory, Center for Applied Medical Research, Pamplona, Spain), Manuel Seijo-Martinez (Department of Neurology, Hospital do Salnes, Pontevedra, Spain), Ramon Rene, Jordi Gascon and Jaume Campdelacreu (Department of Neurology, Hospital de Bellvitage, Barcelona, Spain) for providing DNA samples. Hospital de la Sant Pau, Universitat Autonoma de Spain acknowledges support from the Spanish Ministry of Economy and Competitiveness (grant number PI12/01311), and from Generalitat de Catalunya (2014SGR-235). The Santa Lucia Foundation and the Fondazione Ca’ Granda IRCCS Ospedale Policlinico, Italy, acknowledge the Italian Ministry of Health (grant RC 10.11.12.13/A)

4.2.12 StEP-AD cohort

The Stanford Extreme Phenotypes in Alzheimer's Disease (StEP AD) Study is funded by the National Institutes of Health: R01AG060747 and AG047366

4.2.13 Knight-ADRC

NIH P50 AG05681, P01 AG03991, P01 AG026276, NIA U01 AG058922;

4.2.14 UCSF/NYGC/UAB

Funding for genomes sequenced at HudsonAlpha was generously provided by the Daniel Foundation of Alabama and donors to the HudsonAlpha Foundation Memory and Mobility Fund.

4.2.15 UCL-DRC EOAD

This work was supported by the Medical Research Council (UK), the Biomedical Research Centre at University College London Hospitals NHS Foundation Trust and charitable donations to the UCL Dementia Research Centre.

4.2.16 ADSP

The Alzheimer's Disease Sequencing Project (ADSP) is comprised of two Alzheimer's Disease (AD) genetics consortia and three National Human Genome Research Institute (NHGRI) funded Large Scale Sequencing and Analysis Centers (LSAC). The two AD genetics consortia are the Alzheimer's Disease Genetics Consortium (ADGC) funded by NIA (U01 AG032984), and the Cohorts for Heart and Aging Research in Genomic Epidemiology (CHARGE) funded by NIA (R01 AG033193), the National Heart, Lung, and Blood Institute (NHLBI), other National Institute of Health (NIH) institutes and other foreign governmental and non-governmental organizations. The Discovery Phase analysis of sequence data is supported through UF1AG047133 (to Drs. Schellenberg, Farrer, Pericak-Vance, Mayeux, and Haines); U01AG049505 to Dr. Seshadri; U01AG049506 to Dr. Boerwinkle; U01AG049507 to Dr. Wijsman; and U01AG049508 to Dr. Goate and the Discovery Extension Phase analysis is supported through U01AG052411 to Dr. Goate,

U01AG052410 to Dr. Pericak-Vance and U01 AG052409 to Drs. Seshadri and Fornage, U54 AG052427 to Drs. Schellenberg and Wang, and R01 AG054060 to Dr Naj. The ADGC cohorts include: Adult Changes in Thought (ACT) (UO1 AG006781, UO1 HG004610, UO1 HG006375, U01 HG008657), the Alzheimer's Disease Centers (ADC) (P30 AG019610, P30 AG013846, P50 AG008702, P50 AG025688, P50 AG047266, P30 AG010133, P50 AG005146, P50 AG005134, P50 AG016574, P50 AG005138, P30 AG008051, P30 AG013854, P30 AG008017, P30 AG010161, P50 AG047366, P30 AG010129, P50 AG016573, P50 AG016570, P50 AG005131, P50 AG023501, P30 AG035982, P30 AG028383, P30 AG010124, P50 AG005133, P50 AG005142, P30 AG012300, P50 AG005136, P50 AG033514, P50 AG005681, and P50 AG047270), the Chicago Health and Aging Project (CHAP) (R01 AG11101, RC4 AG039085, K23 AG030944), Indianapolis Ibadan (R01 AG009956, P30 AG010133), the Memory and Aging Project (MAP) (R01 AG17917), Mayo Clinic (MAYO) (R01 AG032990, U01 AG046139, R01 NS080820, RF1 AG051504, P50 AG016574), Mayo Parkinson's Disease controls (NS039764, NS071674, 5RC2HG005605), University of Miami (R01 AG027944, R01 AG028786, R01 AG019085, IIRG09133827, A2011048), the Multi-Institutional Research in Alzheimer's Genetic Epidemiology Study (MIRAGE) (R01 AG09029, R01 AG025259), the National Cell Repository for Alzheimer's Disease (NCRAD) (U24 AG21886), the National Institute on Aging Late Onset Alzheimer's Disease Family Study (NIA- LOAD) (R01 AG041797), the Religious Orders Study (ROS) (P30 AG10161, R01 AG15819), the Texas Alzheimer's Research and Care Consortium (TARCC) (funded by the Darrell K Royal Texas Alzheimer's Initiative), Vanderbilt University/Case Western Reserve University (VAN/CWRU) (R01 AG019757, R01 AG021547, R01 AG027944, R01 AG028786, P01 NS026630, and Alzheimer's Association), the Washington Heights-Inwood Columbia Aging Project (WHICAP) (RF1 AG054023), the University of Washington Families (VA Research Merit Grant, NIA: P50AG005136, R01AG041797, NINDS: R01NS069719), the Columbia University HispanicEstudio Familiar de Influencia Genetica de Alzheimer (EFIGA) (RF1 AG015473), the University of Toronto (UT) (funded by Wellcome Trust, Medical Research Council, Canadian Institutes of Health Research), and Genetic Differences (GD) (R01 AG007584). The CHARGE cohorts are supported in part by National Heart, Lung, and Blood Institute (NHLBI) infrastructure grant HL105756 (Psaty),

RC2HL102419 (Boerwinkle) and the neurology working group is supported by the National Institute on Aging (NIA) R01 grant AG033193.
This work was also supported by National Institute on Aging grants R01 AG048927 to Dr. Farrer, RF1 AG054080 to Dr. Beecham, U24 AG056270 to Dr. Mayeux, RF1 AG057519 to Dr. Farrer, U01 AG062602 to Dr. Farrer, R01 AG067501 to Dr. Mayeux, and U19 AG068753 to Dr. Farrer.
The CHARGE cohorts participating in the ADSP include the following: Austrian Stroke Prevention Study (ASPS), ASPS-Family study, and the Prospective Dementia Registry-Austria (ASPS/PRODEM-Aus), the Atherosclerosis Risk in Communities (ARIC) Study, the Cardiovascular Health Study (CHS), the Erasmus Rucphen Family Study (ERF), the Framingham Heart Study (FHS), and the Rotterdam Study (RS). ASPS is funded by the Austrian Science Fond (FWF) grant number P20545-P05 and P13180 and the Medical University of Graz. The ASPS-Fam is funded by the Austrian Science Fund (FWF) project I904),the EU Joint Programme - Neurodegenerative Disease Research (JPND) in frame of the BRIDGET project (Austria, Ministry of Science) and the Medical University of Graz and the Steiermärkische Krankenanstalten Gesellschaft. PRODEM-Austria is supported by the Austrian Research Promotion agency (FFG) (Project No. 827462) and by the Austrian National Bank (Anniversary Fund, project 15435. ARIC research is carried out as a collaborative study supported by NHLBI contracts (HHSN268201100005C, HHSN268201100006C, HHSN268201100007C, HHSN268201100008C, HHSN268201100009C, HHSN268201100010C, HHSN268201100011C, and HHSN268201100012C). Neurocognitive data in ARIC is collected by U01 2U01HL096812, 2U01HL096814, 2U01HL096899, 2U01HL096902, 2U01HL096917 from the NIH (NHLBI, NINDS, NIA and NIDCD), and with previous brain MRI examinations funded by R01-HL70825 from the NHLBI. CHS research was supported by contracts HHSN268201200036C, HHSN268200800007C, N01HC55222, N01HC85079, N01HC85080, N01HC85081, N01HC85082, N01HC85083, N01HC85086, and grants U01HL080295 and U01HL130114 from the NHLBI with additional contribution from the National Institute of Neurological Disorders and Stroke (NINDS). Additional support was provided by R01AG023629, R01AG15928, and R01AG20098 from the NIA. FHS research is supported by NHLBI contracts N01-HC-25195 and HHSN268201500001I. This study

was also supported by additional grants from the NIA (R01s AG054076, AG049607 and AG033040 and NINDS (R01 NS017950). The ERF study as a part of EUROSPAN (European Special Populations Research Network) was supported by European Commission FP6 STRP grant number 018947 (LSHG-CT-2006-01947) and also received funding from the European Community's Seventh Framework Programme (FP7/2007-2013)/grant agreement HEALTH-F4- 2007-201413 by the European Commission under the programme "Quality of Life and Management of the Living Resources" of 5th Framework Programme (no. QLG2-CT-2002- 01254). High-throughput analysis of the ERF data was supported by a joint grant from the Netherlands Organization for Scientific Research and the Russian Foundation for Basic Research (NWO-RFBR 047.017.043). The Rotterdam Study is funded by Erasmus Medical Center and Erasmus University, Rotterdam, the Netherlands Organization for Health Research and Development (ZonMw), the Research Institute for Diseases in the Elderly (RIDE), the Ministry of Education, Culture and Science, the Ministry for Health, Welfare and Sports, the European Commission (DG XII), and the municipality of Rotterdam. Genetic data sets are also supported by the Netherlands Organization of Scientific Research NWO Investments (175.010.2005.011, 911-03-012), the Genetic Laboratory of the Department of Internal Medicine, Erasmus MC, the Research Institute for Diseases in the Elderly (014-93-015; RIDE2), and the Netherlands Genomics Initiative (NGI)/Netherlands Organization for Scientific Research (NWO) Netherlands Consortium for Healthy Aging (NCHA), project 050-060-810. All studies are grateful to their participants, faculty and staff. The content of these manuscripts is solely the responsibility of the authors and does not necessarily represent the official views of the National Institutes of Health or the U.S. Department of Health and Human Services.

The four LSACs are: the Human Genome Sequencing Center at the Baylor College of Medicine (U54 HG003273), the Broad Institute Genome Center (U54HG003067), The American Genome Center at the Uniformed Services University of the Health Sciences (U01AG057659), and the Washington University Genome Institute (U54HG003079).

Biological samples and associated phenotypic data used in primary data analyses were

stored at Study Investigators institutions, and at the National Cell Repository for Alzheimer's Disease (NCRAD, U24AG021886) at Indiana University funded by NIA. Associated Phenotypic Data used in primary and secondary data analyses were provided by Study Investigators, the NIA funded Alzheimer's Disease Centers (ADCs), and the National Alzheimer's Coordinating Center (NACC, U01AG016976) and the National Institute on Aging Genetics of Alzheimer's Disease Data Storage Site (NIAGADS, U24AG041689) at the University of Pennsylvania, funded by NIA This research was supported in part by the Intramural Research Program of the National Institutes of health, National Library of Medicine. Contributors to the Genetic Analysis Data included Study Investigators on projects that were individually funded by NIA, and other NIH institutes, and by private U.S. organizations, or foreign governmental or nongovernmental organizations.

Data collection and sharing for this project was funded by the Alzheimer's Disease Neuroimaging Initiative (ADNI) (National Institutes of Health Grant U01 AG024904) and DOD ADNI (Department of Defense award number W81XWH-12-2-0012). ADNI is funded by the National Institute on Aging, the National Institute of Biomedical Imaging and Bioengineering, and through generous contributions from the following: AbbVie, Alzheimer's Association; Alzheimer's Drug Discovery Foundation; Araclon Biotech; BioClinica, Inc.; Biogen; Bristol-Myers Squibb Company; CereSpir, Inc.; Cogstate; Eisai Inc.; Elan Pharmaceuticals, Inc.; Eli Lilly and Company; EuroImmun; F. Hoffmann-La Roche Ltd and its affiliated company Genentech, Inc.; Fujirebio; GE Healthcare; IXICO Ltd.; Janssen Alzheimer Immunotherapy Research & Development, LLC.; Johnson & Johnson Pharmaceutical Research & Development LLC.; Lumosity; Lundbeck; Merck & Co., Inc.; Meso Scale Diagnostics, LLC.; NeuroRx Research; Neurotrack Technologies; Novartis Pharmaceuticals Corporation; Pfizer Inc.; Piramal Imaging; Servier; Takeda Pharmaceutical Company; and Transition Therapeutics. The Canadian Institutes of Health Research is providing funds to support ADNI clinical sites in Canada. Private sector contributions are facilitated by the Foundation for the National Institutes of Health (www.fnih.org). The grantee organization is the Northern California Institute for Research and Education, and the study is coordinated by the Alzheimer's Therapeutic Research

Institute at the University of Southern California. ADNI data are disseminated by the Laboratory for Neuro Imaging at the University of Southern California.

4.3 Supplementary Authors

Investigators of several cohorts contributed to samples analyzed in this work, but did not participate in analysis or writing of this report:

4.3.1 PERADES Cohort:

Keeley Brookes[1], Tamar Guetta-Baranes[2], Elisa Toppi[3], Francesca Salani[3], Marina Arcaro[4], Chiara Fenoglio[5], Roberta Cecchetti[6], Elio Scarpini[7], Sandro Sorbi[8], Monica Diez-Fairen[9], Ignacio Alvarez[9], Miquel Aguilar[9], MRC: Simon Lovestone[10], John Powell[11], Carol Brayne[12], David Rubinsztein[12], Nandini Badarinarayan[13], Eloy Rodriguez-Rodriguez[14], Carmen Lage[14], Sara Lopez-Garcia[14], Emanuele Costantini[15], Michela Orsini[15], Francesco Panza[16], Nerisa Banaj[17], Federica Piras[17] and Daniela Vecchio[17]

(1) Nottingham Trent; **(2)**Human Genetics. UoN; (**3**) IRCCS Fondazione Santa Lucia, Department of Clinical and Behavioral Neurology, Experimental Neuro-psychobiology Lab Via Ardeatina, 306, I-00179 Roma, Italy; **(4)** Fondazione IRCCS Ca' Granda, Ospedale Policlinico; **(5)** University of Milan, Dino Ferrari Center, Milan, Italy; 6Institute of Gerontology and Geriatrics, Department of Medicine and Surgery, University of Perugia, Italy; **(7)** University of Milan, Centro Dino Ferrari, CRC Molecular basis of Neuro-Psycho-Geriatrics diseases, Milan, Italy; **(8)** Department of Neuroscience, Psychology, Drug Research and Child Health , University of Florence, Italy; **(9)** Memory Disorders Unit, Department of Neurology, Hospital Universitari Mutua de Terrassa, Terrassa, Barcelona, Spain; **(10)** Department of Psychiatry, Medical Sciences Division, University of Oxford, Oxford, UK.; **(11)** Kings College London, Institute of Psychiatry, Department of Neuroscience, De Crespigny Park, Denmark Hill, London, UK; **(12)** Institute of Public Health, University of Cambridge, Cambridge, UK.; (**13)** Division of Psychological Medicine and Clinical Neuroscience, School of Medicine, Cardiff University, Cardiff, UK; **(14)** Neurology Service and Centro de Investigación en Red de Enfermedades Neurodegenerativas (CIBERNED), Marques de Valdecilla University Hospital (University of Cantabria and IDIVAL), Santander, Spain **(15)** Department of Neuroscience, Catholic University of Sacred Heart, Fondazione Policlinico Universitario A. Gemelli IRCCS, Rome, Italy **(16)** National Institute of Gastroenterology and Research Hospital IRCCS “S. De Bellis” Castellana Grotte, Bari Italy **(17)** Laboratory of Neuropsychiatry,IRCCS Santa Lucia Foundation, Rome, Italy

4.3.2 StEP AD investigators

Clifton L. Dalgard, PhD[1], William J. Jagust, MD[2], Sterling C. Johnson, PhD[3], David A. Wolk, MD[4], Joel H. Kramer, PsyD[5], Bradford C. Dickerson, MD[6], David A. Bennett, MD[7], Sofiya Milman, MD[8], Bruno Dubois, MD, PhD[9], Ruth O'Hara, PhD[10], Sherry A. Beaudreau, PhD[11]

[1]Uniformed Services University of the Health Sciences;[2]University of California, Berkeley; [3]University of Wisconsin; [4]University of Pennsylvania; [5]University of California, San Francisco; [6]Harvard University; [7]Rush University; [8]Albert Einstein College of Medicine; [9]Brain and Spine Institute (ICM), France; [10]Stanford University
[11]VA Palo Alto Health Care System

4.3.3 Knight ADRC investigators

Achal Neupane[1,3,4], John P Budde[1,3,4], Fengxian Wang[1,3,4], Joanne Norton[1,3,4], Gen Gentsch[1,3,4], John C Morris[2,3]

Departments of [1]Psychiatry, [2]Neurology, [3]Hope Center for Neurological Disorders, [4]NeuroGenomics and Informatics Center, Washington University School of Medicine, St. Louis, Missouri, USA.

4.3.4 ADNI database

A subset of the data used in this article was obtained from the Alzheimer's Disease Neuroimaging Initiative (ADNI) database (adni.loni.usc.edu). A complete listing of ADNI investigators can be found at:
http://adni.loni.usc.edu/wp-content/uploads/how_to_apply/ADNI_Acknowledgement_List.pdf

5 References

1. Holstege, H. *et al.* Exome sequencing identifies rare damaging variants in ATP8B4 and ABCA1 as risk factors for Alzheimer's disease. *Nature Genetics* **54**, 1786-1794 (2022).
2. McKhann, G.M. *et al.* The diagnosis of dementia due to Alzheimer's disease: recommendations from the National Institute on Aging-Alzheimer's Association workgroups on diagnostic guidelines for Alzheimer's disease. *Alzheimers Dement* **7**, 263-9 (2011).
3. McKhann, G. *et al.* Clinical diagnosis of Alzheimer's disease: report of the NINCDS-ADRDA Work Group under the auspices of Department of Health and Human Services Task Force on Alzheimer's Disease. *Neurology* **34**, 939-44 (1984).
4. Bellenguez, C. *et al.* Contribution to Alzheimer's disease risk of rare variants in TREM2, SORL1, and ABCA7 in 1779 cases and 1273 controls. *Neurobiol Aging* **59**, 220 e1-220 e9 (2017).
5. Nicolas, G. *et al.* SORL1 rare variants: a major risk factor for familial early-onset Alzheimer's disease. *Mol Psychiatry* (2015).
6. Nicolas, G. *et al.* Screening of dementia genes by whole-exome sequencing in early-onset Alzheimer disease: input and lessons. *European Journal of Human Genetics* **24**, 710-716 (2015).
7. Lambert, J.C. *et al.* Genome-wide association study identifies variants at CLU and CR1 associated with Alzheimer's disease. *Nat Genet* **41**, 1094-9 (2009).
8. The 3C Study Group. Vascular Factors and Risk of Dementia: Design of the Three-City Study and Baseline Characteristics of the Study Population. *Neuroepidemiology* **22**, 316-325 (2003).
9. Genin, E. *et al.* The French Exome (FREX) Project: A Population-based Panel of Exomes to Help Filter Out Common Local Variants, in: The 2017 Annual Meeting of the International Genetic Epidemiology Society. *Genetic Epidemiology* **41**, 691 (2017).
10. Deli, M. *et al.* Prediction of Dementia in Primary Care Patients. *PLoS ONE* **6**(2011).
11. Luck, T. *et al.* Mild Cognitive Impairment in General Practice: Age-Specific Prevalence and Correlate Results from the German Study on Ageing, Cognition and Dementia in Primary Care Patients (AgeCoDe). *Dementia and Geriatric Cognitive Disorders* **24**, 307-316 (2007).
12. Zaudig, M. *et al.* SIDAM – A Structured Interview for the diagnosis of Dementia of the Alzheimer type, Multi-infarct dementia and dementias of other aetiology according to ICD-10 and DSM-III-R. *Psychological Medicine* **21**, 225-236 (2009).
13. Alcolea, D. *et al.* The Sant Pau Initiative on Neurodegeneration (SPIN) cohort: A data set for biomarker discovery and validation in neurodegenerative disorders. *Alzheimer's & Dementia: Translational Research & Clinical Interventions* **5**, 597-609 (2019).
14. Alcolea, D. *et al.* Amyloid precursor protein metabolism and inflammation markers in preclinical Alzheimer disease. *Neurology* **85**, 626-633 (2015).
15. Ikram, M.A. *et al.* Objectives, design and main findings until 2020 from the Rotterdam Study. *European Journal of Epidemiology* **35**, 483-517 (2020).

16. Hofman, A. *et al.* The Rotterdam Study: 2014 objectives and design update. *European Journal of Epidemiology* **28**, 889-926 (2013).
17. Copeland, J.R. *et al.* A semi-structured clinical interview for the assessment of diagnosis and mental state in the elderly: the Geriatric Mental State Schedule. I. Development and reliability. *Psychol Med* **6**, 439-49 (1976).
18. Roth, M. *et al.* CAMDEX. A standardised instrument for the diagnosis of mental disorder in the elderly with special reference to the early detection of dementia. *Br J Psychiatry* **149**, 698-709 (1986).
19. van der Flier, W.M. & Scheltens, P. Amsterdam Dementia Cohort: Performing Research to Optimize Care. *J Alzheimers Dis* **62**, 1091-1111 (2018).
20. Netherlands Brain Bank. http://www.brainbank.nl/.
21. Holstege, H. *et al.* The 100-plus Study of cognitively healthy centenarians: rationale, design and cohort description. *Eur J Epidemiol* **33**, 1229-1249 (2018).
22. Kahle-Wrobleski, K., Corrada, M.M., Li, B. & Kawas, C.H. Sensitivity and specificity of the mini-mental state examination for identifying dementia in the oldest-old: the 90+ study. *J Am Geriatr Soc* **55**, 284-9 (2007).
23. Legdeur, N. *et al.* Resilience to cognitive impairment in the oldest-old: design of the EMIF-AD 90+ study. *BMC Geriatrics* **18**(2018).
24. Guerreiro, R. *et al.* (2018).
25. Berg, L. *et al.* Clinicopathologic Studies in Cognitively Healthy Aging and Alzheimer Disease. *Archives of Neurology* **55**(1998).
26. Cochran, J.N. *et al.* Genome sequencing for early-onset or atypical dementia: high diagnostic yield and frequent observation of multiple contributory alleles. *Molecular Case Studies* **5**(2019).
27. Kunkle, B.W. *et al.* Genetic meta-analysis of diagnosed Alzheimer's disease identifies new risk loci and implicates Aβ, tau, immunity and lipid processing. *Nature Genetics* **51**, 414-430 (2019).
28. Beecham, G.W. *et al.* The Alzheimer's Disease Sequencing Project: Study design and sample selection. *Neurology Genetics* **3**(2017).
29. Andersen, O.M. *et al.* Relying on the relationship with known disease-causing variants in homologous proteins to predict pathogenicity of SORL1 variants in Alzheimer's disease. *BioRxiv preprint* (2023).
30. Bellenguez, C. *et al.* New insights into the genetic etiology of Alzheimer's disease and related dementias. *Nature Genetics* **54**, 412-436 (2022).
31. Ioannidis, N.M. *et al.* REVEL: An Ensemble Method for Predicting the Pathogenicity of Rare Missense Variants. *The American Journal of Human Genetics* **99**, 877-885 (2016).
32. Cheng, J. *et al.* Accurate proteome-wide missense variant effect prediction with AlphaMissense. *Science* **381**(2023).
33. Thomas, G. Furin at the cutting edge: from protein traffic to embryogenesis and disease. *Nat Rev Mol Cell Biol* **3**, 753-66 (2002).
34. Jacobsen, L. *et al.* Activation and functional characterization of the mosaic receptor SorLA/LR11. *J. Biol. Chem.* **276**, 22788-22796 (2001).
35. Hermey, G. The Vps10p-domain receptor family. *Cell. Mol. Life Sci.* **66**, 2677-2689 (2009).

36. Quistgaard, E.M. *et al.* Ligands bind to Sortilin in the tunnel of a ten-bladed b-propeller domain. *Nat. Struct. Mol. Biol.* **16**, 96-98 (2009).
37. Kitago, Y. *et al.* Structural basis for amyloidogenic peptide recognition by sorLA. *Nat. Struct. Mol. Biol.* **22**, 199-206 (2015).
38. Copley, R.R., Russell, R.B. & Ponting, C.P. Sialidase-like Asp-boxes: sequence-similar structures within different protein folds. *Protein Sci* **10**, 285-92 (2001).
39. Crennell, S.J., Garman, E.F., Laver, W.G., Vimr, E.R. & Taylor, G.L. Crystal structure of a bacterial sialidase (from Salmonella typhimurium LT2) shows the same fold as an influenza virus neuraminidase. *Proc Natl Acad Sci U S A* **90**, 9852-6 (1993).
40. Quistgaard, E.M. & Thirup, S.S. Sequence and structural analysis of the Asp-box motif and Asp-box beta-propellers; a widespread propeller-type characteristic of the Vps10 domain family and several glycoside hydrolase families. *BMC Struct. Biol.* **9**, 46 (2009).
41. Westergaard, U.B. *et al.* Functional organization of the sortilin Vps10p domain. *J. Biol. Chem.* **279**, 50221-50229 (2004).
42. Jacobsen, L. *et al.* Molecular characterization of a novel human hybrid-type receptor that binds the a_2-macroglobulin receptor-associated protein. *J. Biol. Chem.* **271**, 31379-31383 (1996).
43. Pottier, C. *et al.* High frequency of potentially pathogenic SORL1 mutations in autosomal dominant early-onset Alzheimer disease. *Mol. Psychiatry* **17**, 875-879 (2012).
44. Caglayan, S. *et al.* Lysosomal sorting of amyloid-beta by the SORLA receptor is impaired by a familial Alzheimer's disease mutation. *Sci. Transl. Med.* **6**, 223ra20 (2014).
45. Vardarajan, B.N. *et al.* Coding mutations in SORL1 and Alzheimer disease. *Ann. Neurol.* **77**, 215-227 (2015).
46. Springer, T.A. An extracellular beta-propeller module predicted in lipoprotein and scavenger receptors, tyrosine kinases, epidermal growth factor precursor, and extracellular matrix components. *J. Mol. Biol.* **283**, 837-862 (1998).
47. Jeon, H. *et al.* Implications for familial hypercholesterolemia from the structure of the LDL receptor YWTD-EGF domain pair. *Nat. Struct. Biol.* **8**, 499-504. (2001).
48. Zong, Y. *et al.* Structural basis of agrin-LRP4-MuSK signaling. *Genes Dev.* **26**, 247-258 (2012).
49. Chen, S. *et al.* Structural and functional studies of LRP6 ectodomain reveal a platform for Wnt signaling. *Dev. Cell* **21**, 848-861 (2011).
50. Cheng, Z. *et al.* Crystal structures of the extracellular domain of LRP6 and its complex with DKK1. *Nat. Struct. Mol. Biol.* **18**, 1204-1210 (2011).
51. Ahn, V.E. *et al.* Structural basis of Wnt signaling inhibition by Dickkopf binding to LRP5/6. *Dev. Cell* **21**, 862-873 (2011).
52. Bourhis, E. *et al.* Wnt antagonists bind through a short peptide to the first b-propeller domain of LRP5/6. *Structure* **19**, 1433-1442 (2011).
53. Hirai, H. *et al.* Structural basis for ligand capture and release by the endocytic receptor ApoER2. *EMBO Rep* **18**, 982-999 (2017).

54. Takagi, J., Yang, Y., Liu, J.H., Wang, J.H. & Springer, T.A. Complex between nidogen and laminin fragments reveals a paradigmatic beta-propeller interface. *Nature* **424**, 969-974 (2003).
55. Andersen, O.M., Dagil, R. & Kragelund, B.B. New horizons for lipoprotein receptors: communication by beta-propellers. *J. Lipid Res.* **54**, 2763-2774 (2013).
56. Jensen, A.M.G. *et al.* Dimerization of the Alzheimer's disease pathogenic receptor SORLA regulates its association with retromer *Proc. Natl. Acad. Sci. U. S. A.* (2022).
57. Nogi, T. How multi-scale structural biology elucidated context-dependent variability in ectodomain conformation along with the ligand capture and release cycle for LDLR family members. *Biophys. Rev.* **10**, 481-492 (2018).
58. Widhalm, K., Dirisamer, A., Lindemayr, A. & Kostner, G. Diagnosis of families with familial hypercholesterolaemia and/or Apo B-100 defect by means of DNA analysis of LDL-receptor gene mutations. *J. Inherit. Metab. Dis.* **30**, 239-247 (2007).
59. Kantarci, S. *et al.* Mutations in LRP2, which encodes the multiligand receptor megalin, cause Donnai-Barrow and facio-oculo-acoustico-renal syndromes. *Nat. Genet.* **39**, 957-959 (2007).
60. Ai, M., Heeger, S., Bartels, C.F. & Schelling, D.K. Clinical and molecular findings in osteoporosis-pseudoglioma syndrome. *Am J Hum Genet* **77**, 741-53 (2005).
61. Toomes, C. *et al.* Mutations in LRP5 or FZD4 Underlie the Common Familial Exudative Vitreoretinopathy Locus on Chromosome 11q. *Am J Hum Genet* **74**, 721-30 (2004).
62. Jensen, H.K. *et al.* Spectrum of LDL receptor gene mutations in Denmark: implications for molecular diagnostic strategy in heterozygous familial hypercholesterolemia. *Atherosclerosis* **146**, 337-344 (1999).
63. Etxebarria, A. *et al.* Functional characterization and classification of frequent low-density lipoprotein receptor variants. *Hum. Mutat.* **36**, 129-141 (2015).
64. Jensen, H.K. *et al.* A common W556S mutation in the LDL receptor gene of Danish patients with familial hypercholesterolemia encodes a transport-defective protein. *Atherosclerosis* **131**, 67-72 (1997).
65. Van Wesenbeeck, L. *et al.* Six Novel Missense Mutations in the LDL Receptor-Related Protein 5 (LRP5) Gene in Different Conditions with an Increased Bone Density. *Am. J. Hum. Genet.* **72**, 763-771. (2003).
66. Santos, P.C. *et al.* Presence and type of low density lipoprotein receptor (LDLR) mutation influences the lipid profile and response to lipid-lowering therapy in Brazilian patients with heterozygous familial hypercholesterolemia. *Atherosclerosis* **233**, 206-210 (2014).
67. Hobbs, H.H., Brown, M.S. & Goldstein, J.L. Molecular genetics of the LDL receptor gene in familial hypercholesterolemia. *Hum. Mutat.* **1**, 445-466 (1992).
68. Ekstrom, U., Abrahamson, M., Sveger, T., Lombardi, P. & Nilsson-Ehle, P. An efficient screening procedure detecting six novel mutations in the LDL receptor gene in Swedish children with hypercholesterolemia. *Hum. Genet.* **96**, 147-150 (1995).
69. Bertolini, S. *et al.* Clinical expression of familial hypercholesterolemia in clusters of mutations of the LDL receptor gene that cause a receptor-defective or receptor-negative phenotype. *Arterioscler. Thromb. Vasc. Biol.* **20**, E41-52 (2000).

70. Li, Y. *et al.* LRP4 mutations alter Wnt/beta-catenin signaling and cause limb and kidney malformations in Cenani-Lenz syndrome. *Am. J. Hum. Genet.* **86**, 696-706 (2010).
71. Steel, E. *et al.* Cenani-Lenz syndactyly in siblings with a novel homozygous LRP4 mutation and recurrent hypoglycaemia. *Clin. Dysmorphol.* **29**, 73-80 (2020).
72. Holstege, H. *et al.* Exome sequencing identifies rare damaging variants in ATP8B4 and ABCA1 as risk factors for Alzheimer’s disease. *Nat. Genet.* **54**, 1786-1794 (2022).
73. Gorski, B., Kubalska, J., Naruszewicz, M. & Lubinski, J. LDL-R and Apo-B-100 gene mutations in Polish familial hypercholesterolemias. *Hum. Genet.* **102**, 562-565 (1998).
74. Ebhardt, M. *et al.* Mutation analysis in 46 German families with familial hypercholesterolemia: identification of 8 new mutations. . *Hum. Mutat.* **13**, 257 (1999).
75. Tricot-Guerber, F. *et al.* Identification of a mutation, N543H, in exon 11 of the low-density lipoprotein receptor gene in a French family with familial hypercholesterolemia. *Hum. Mutat.* **6**, 87-88 (1995).
76. Jensen, H.K. *et al.* Two mutations in the same low-density lipoprotein receptor allele act in synergy to reduce receptor function in heterozygous familial hypercholesterolemia. *Hum. Mutat.* **9**, 437-444 (1997).
77. Ohkawara, B. *et al.* LRP4 third beta-propeller domain mutations cause novel congenital myasthenia by compromising agrin-mediated MuSK signaling in a position-specific manner. *Hum. Mol. Genet.* **23**, 1856-1868 (2014).
78. Leupin, O. *et al.* Bone overgrowth-associated mutations in the LRP4 gene impair sclerostin facilitator function. *J. Biol. Chem.* **286**, 19489-19500 (2011).
79. Cheung, W.M. *et al.* A family with osteoporosis pseudoglioma syndrome due to compound heterozygosity of two novel mutations in the LRP5 gene. *Bone* **39**, 470-476 (2006).
80. Gong, Y. *et al.* LDL receptor-related protein 5 (LRP5) affects bone accrual and eye development. *Cell* **107**, 513-523 (2001).
81. Jiao, X., Ventruto, V., Trese, M.T., Shastry, B.S. & Hejtmancik, J.F. Autosomal recessive familial exudative vitreoretinopathy is associated with mutations in LRP5. *Am. J. Hum. Genet.* **75**, 878-884 (2004).
82. Singh, R. *et al.* Rare nonconservative LRP6 mutations are associated with metabolic syndrome. *Hum. Mutat.* **34**, 1221-1225 (2013).
83. Day, I.N. *et al.* Spectrum of LDL receptor gene mutations in heterozygous familial hypercholesterolemia. *Hum. Mutat.* **10**, 116-127 (1997).
84. Humphries, S.E. *et al.* Genetic causes of familial hypercholesterolaemia in patients in the UK: relation to plasma lipid levels and coronary heart disease risk. *J. Med. Genet.* **43**, 943-949 (2006).
85. Zhang, L. *et al.* Whole Exome Sequencing Analysis Identifies Mutations in LRP5 in Indian Families with Familial Exudative Vitreoretinopathy. *Genet. Test. Mol. Biomarkers.* **20**, 346-351 (2016).
86. Boonstra, F.N. *et al.* Clinical and molecular evaluation of probands and family members with familial exudative vitreoretinopathy. *Invest. Ophthalmol. Vis. Sci.* **50**, 4379-4385 (2009).

87. Fei, P. *et al.* Identification of two novel LRP5 mutations in families with familial exudative vitreoretinopathy. *Mol. Vis.* **20**, 395-409 (2014).
88. Cnossen, W.R. *et al.* Whole-exome sequencing reveals LRP5 mutations and canonical Wnt signaling associated with hepatic cystogenesis. *Proc. Natl. Acad. Sci. U. S. A.* **111**, 5343-5348 (2014).
89. Sukenik Halevy, R. *et al.* Mutations in the fourth beta-propeller domain of LRP4 are associated with isolated syndactyly with fusion of the third and fourth fingers. *Hum. Mutat.* **39**, 811-815 (2018).
90. Huybrechts, Y. *et al.* Identification of Compound Heterozygous Variants in LRP4 Demonstrates That a Pathogenic Variant outsie the Third b-propeller Domain Can Cause Sclerosteosis. *Genes* **13**(2022).
91. Campbell, I.D. & Bork, P. Epidermal growth factor-like modules. *Curr. Opin. Struct. Biol.* **3**, 385-392 (1993).
92. Herz, J. The LDL receptor gene family: (un)expected signal transducers in the brain. *Neuron* **29**, 571-581. (2001).
93. Yamazaki, H. *et al.* Elements of neural adhesion molecules and a yeast vacuolar protein sorting receptor are present in a novel mammalian low density lipoprotein receptor family member. *J. Biol. Chem.* **271**, 24761-24768 (1996).
94. Mörwald, S. *et al.* A novel mosaic protein containing LDL receptor elements is highly conserved in humans and chickens. *Arterioscler. Thromb. Vasc. Biol.* **17**, 996-1002 (1997).
95. Takagi, J., Beglova, N., Yalamanchili, P., Blacklow, S.C. & Springer, T.A. Definition of EGF-like, closely interacting modules that bear activation epitopes in integrin beta subunits. *Proc. Natl. Acad. Sci. U. S. A.* **98**, 11175-11180 (2001).
96. Zhang, D.W. *et al.* Binding of proprotein convertase subtilisin/kexin type 9 to epidermal growth factor-like repeat A of low density lipoprotein receptor decreases receptor recycling and increases degradation. *J. Biol. Chem.* **282**, 18602-18612 (2007).
97. Thonberg, H. *et al.* Identification and description of three families with familial Alzheimer disease that segregate variants in the SORL1 gene. *Acta Neuropathol. Commun.* **5**, 43 (2017).
98. Beglova, N., Blacklow, S.C., Takagi, J. & Springer, T.A. Cysteine-rich module structure reveals a fulcrum for integrin rearrangement upon activation. *Nat. Struct. Biol.* **9**, 282-287 (2002).
99. Andersen, O.M. *et al.* SorLA/LR11, a neuronal sorting receptor that regulates processing of the amyloid precursor protein. *Proc. Natl. Acad. Sci. U. S. A.* **102**, 13461-13466 (2005).
100. Andersen, O.M. *et al.* Molecular dissection of the interaction between APP and its neuronal trafficking receptor SorLA/LR11. *Biochemistry* **45**, 2618-2628 (2006).
101. Monti, G. & Andersen, O.M. 20 Years Anniversary for SORLA/SORL1 (1996-2016). *Receptor Clin. Invest.* **4**, e1611 (2017).
102. Mehmedbasic, A. *et al.* SorLA Complement-type Repeat Domains Protect the Amyloid Precursor Protein against Processing. *J. Biol. Chem.* **290**, 3359-3376 (2015).

103. Bieri, S., Djordjevic, J.T., Daly, N.L., Smith, R. & Kroon, P.A. Disulfide bridges of a cysteine-rich repeat of the LDL receptor ligand- binding domain. *Biochemistry* **34**, 13059-13065 (1995).
104. Bieri, S., Djordjevic, J.T., Jamshidi, N., Smith, R. & Kroon, P.A. Expression and disulfide-bond connectivity of the second ligand-binding repeat of the human LDL receptor. *FEBS Lett.* **371**, 341-344 (1995).
105. Daly, N.L., Scanlon, M.J., Djordjevic, J.T., Kroon, P.A. & Smith, R. Three-dimensional structure of a cysteine-rich repeat from the low- density lipoprotein receptor. *Proc. Natl. Acad. Sci. U. S. A.* **92**, 6334-6338 (1995).
106. Daly, N.L., Djordjevic, J.T., Kroon, P.A. & Smith, R. Three-dimensional structure of the second cysteine-rich repeat from the human low-density lipoprotein receptor. *Biochemistry* **34**, 14474-14481 (1995).
107. Fass, D., Blacklow, S., Kim, P.S. & Berger, J.M. Molecular basis of familial hypercholesterolaemia from structure of LDL receptor module. *Nature* **388**, 691-693 (1997).
108. Huang, W., Dolmer, K. & Gettins, P.G.W. NMR solution structure of complement-like repeat CR8 from the low density lipoprotein receptor-related protein. *J. Biol. Chem.* **274**, 14130-14136 (1999).
109. Dolmer, K., Huang, W. & Gettins, P.G.W. NMR solution structure of complement-like repeat CR3 from the low density lipoprotein receptor-related rrotein. Evidence for specific binding to the receptor binding domain of human a_2-macroglobulin. *J. Biol. Chem.* **275**, 3264-3269 (2000).
110. Simonovic, M. *et al.* Calcium coordination and pH dependence of the calcium affinity of ligand-binding repeat CR7 from the LRP. Comparison with related domains from the LRP and the LDL receptor. *Biochemistry* **40**, 15127-15134. (2001).
111. Guttman, M., Prieto, J.H., Handel, T.M., Domaille, P.J. & Komives, E.A. Structure of the minimal interface between ApoE and LRP. *J. Mol. Biol.* **398**, 306-319 (2010).
112. Dagil, R., O'Shea, C., Nykjaer, A., Bonvin, A.M. & Kragelund, B.B. Gentamicin binds to the megalin receptor as a competitive inhibitor using the common ligand binding motif of complement type repeats: insight from the nmr structure of the 10th complement type repeat domain alone and in complex with gentamicin. *J. Biol. Chem.* **288**, 4424-4435 (2013).
113. Jensen, G.A. *et al.* Binding Site Structure of One LRP-RAP Complex:Implications for a Common Ligand-Receptor Binding Motif. *J. Mol. Biol.* **362**, 700-716 (2006).
114. Yamamoto, T. & Ryan, R.O. Domain swapping reveals that low density lipoprotein (LDL) type A repeat order affects ligand binding to the LDL receptor. *J. Biol. Chem.* **284**, 13396-13400 (2009).
115. Kim, D.-H. *et al.* Exon/intron organization, chromosome localization, alternative splicing, and transcription units of the human apolipoprotein E receptor 2 gene. *J. Biol. Chem.* **272**, 8498-8504. (1997).
116. Brandes, C. *et al.* Alternative splicing in the ligand binding domain of mouse apoE receptor-2 produces receptor variants binding reelin but not alpha2-macroglobulin. *J. Biol. Chem.* **276**, 22160-22169 (2001).
117. Sakai, K. *et al.* A neuronal VLDLR variant lacking the third complement-type repeat exhibits high capacity binding of apoE containing lipoproteins. *Brain Res.* **1276**, 11-21 (2009).

118. Atkins, A.R., Brereton, I.M., Kroon, P.A., Lee, H.T. & Smith, R. Calcium is essential for the structural integrity of the cysteine-rich, ligand-binding repeat of the low-density lipoprotein receptor. *Biochemistry* **37**, 1662-1670 (1998).
119. Abbadi, A. *et al.* Involvement of side functions in peptide structures: the Asx turn. Occurence and conformational aspects. *J. Am. Chem. Soc.* **113**, 2729-2735 (1991).
120. Andersen, O.M. *et al.* Identification of the Minimal Functional Unit in the Low Density Lipoprotein Receptor-related Protein for Binding the Receptor-associated Protein (RAP). *J. Biol. Chem.* **275**, 21017-21024 (2000).
121. Andersen, O.M. *et al.* Specific Binding of a-Macroglobulin to Complement-type Repeat CR4 of the Low Density Lipoprotein Receptor-related Protein. *Biochemistry* **39**, 10627-10633 (2000).
122. Andersen, O.M. *et al.* Analysis of a two-domain binding site for the urokinase-type plasminogen activator - plasminogen activator inhibitor-1 complex in low density lipoprotein receptor-related protein. *Biochem. J.*, 289-296 (2001).
123. Nielsen, K.L. *et al.* Identification of residues in a-macroglobulins important for binding to the a_2-macroglobulin receptor/Low density lipoprotein receptor-related protein. *J. Biol. Chem.* **271**, 12909-12912 (1996).
124. Yasui, N. *et al.* Structure of a receptor-binding fragment of reelin and mutational analysis reveal a recognition mechanism similar to endocytic receptors. *Proc. Natl. Acad. Sci. U. S. A.* **104**, 9988-9993 (2007).
125. Verdaguer, N., Fita, I., Reithmayer, M., Moser, R. & Blaas, D. X-ray structure of a minor group human rhinovirus bound to a fragment of its cellular receptor protein. *Nat. Struct. Mol. Biol.* **11**, 429-434 (2004).
126. Fisher, C., Beglova, N. & Blacklow, S.C. Structure of an LDLR-RAP complex reveals a general mode for ligand recognition by lipoprotein receptors. *Mol. Cell* **22**, 277-283 (2006).
127. Yasui, N., Nogi, T. & Takagi, J. Structural basis for specific recognition of reelin by its receptors. *Structure* **18**, 320-331 (2010).
128. Nikolic, J. *et al.* Structural basis for the recognition of LDL-receptor family members by VSV glycoprotein. *Nat. Commun.* **9**, 1029 (2018).
129. Andersen, O.M., Benhayon, D., Curran, T. & Willnow, T.E. Differential binding of ligands to the apolipoprotein e receptor 2. *Biochemistry* **42**, 9355-9364. (2003).
130. Goncalves-Mendes, N. *et al.* Mouse SCO-spondin, a gene of the thrombospondin type 1 repeat (TSR) superfamily expressed in the brain. *Gene* **312**, 263-270 (2003).
131. Bieri, S. *et al.* Folding, calcium binding, and structural characterization of a concatemer of the first and second ligand-binding modules of the low-density lipoprotein receptor. *Biochemistry* **37**, 10994-11002 (1998).
132. North, C.L. & Blacklow, S.C. Structural independence of ligand-binding modules five and six of the LDL receptor. *Biochemistry* **38**, 3926-3935 (1999).
133. North, C.L. & Blacklow, S.C. Evidence that familial hypercholesterolemia mutations of the LDL receptor cause limited local misfolding in an LDL-A module pair. *Biochemistry* **39**, 13127-13135 (2000).
134. Rudenko, G. *et al.* Structure of the LDL receptor extracellular domain at endosomal pH. *Science* **298**, 2353-8. (2002).
135. Banerjee, K., Yakovlev, S., Gruschus, J.M., Medved, L. & Tjandra, N. Nuclear Magnetic Resonance Solution Structure of the Recombinant Fragment Containing

Three Fibrin-Binding Cysteine-Rich Domains of the Very Low Density Lipoprotein Receptor. *Biochemistry* **57**, 4395-4403 (2018).
136. Skeldal, S. *et al.* Binding areas of urokinase-type plasminogen activator-plasminogen activator inhibitor-1 complex for endocytosis receptors of the low-density lipoprotein receptor family, determined by site-directed mutagenesis. *FEBS J.* **273**, 5143-5159 (2006).
137. Steentoft, C. *et al.* Precision mapping of the human O-GalNAc glycoproteome through SimpleCell technology. *EMBO J.* **32**, 1478-1488 (2013).
138. Pedersen, N.B. *et al.* Low density lipoprotein receptor class A repeats are O-glycosylated in linker regions. *J. Biol. Chem.* **289**, 17312-17324 (2014).
139. De Falco, L. *et al.* Functional and clinical impact of novel TMPRSS6 variants in iron-refractory iron-deficiency anemia patients and genotype-phenotype studies. *Hum. Mutat.* **35**, 1321-1329 (2014).
140. McDonald, C.J. *et al.* Functional analysis of matriptase-2 mutations and domains: insights into the molecular basis of iron-refractory iron deficiency anemia. *Am. J. Physiol. Cell Physiol.* **308**, C539-547 (2015).
141. Witzel-Schlomp, K. *et al.* Heterogeneity in the genetic basis of human complement C9 deficiency. *Immunogenetics* **48**, 144-147 (1998).
142. El Bitar, F. *et al.* Genetic Study of Alzheimer's Disease in Saudi Population. *J. Alzheimers Dis.* **67**, 231-242 (2019).
143. Cao, L., Zhu, F. & Qiu, G. Early-onset Alzheimer's disease may be associated with sortilin-related receptor 1 gene mutation: A family report and review. *Radiol. Case Rep.* **16**, 30-34 (2021).
144. Verheijen, J. *et al.* A comprehensive study of the genetic impact of rare variants in SORL1 in European early-onset Alzheimer's disease. *Acta Neuropathol.* **132**, 213-224 (2016).
145. Joutel, A. *et al.* Strong clustering and stereotyped nature of Notch3 mutations in CADASIL patients. *Lancet* **350**, 1511-1515 (1997).
146. Blacklow, S.C. & Kim, P.S. Protein folding and calcium binding defects arising from familial hypercholesterolemia mutations of the LDL receptor. *Nat. Struct. Biol.* **3**, 758-762 (1996).
147. Rubinsztein, D.C., Jialal, I., Leitersdorf, E., Coetzee, G.A. & van der Westhuyzen, D.R. Identification of two new LDL-receptor mutations causing homozygous familial hypercholesterolemia in a South African of Indian origin. *Biochim. Biophys. Acta* **1182**, 75-82 (1993).
148. Leitersdorf, E. *et al.* A missense mutation in the low density lipoprotein receptor gene causes familial hypercholesterolemia in Sephardic Jews. *Hum. Genet.* **91**, 141-147 (1993).
149. Etxebarria, A. *et al.* Activity-associated effect of LDL receptor missense variants located in the cysteine-rich repeats. *Atherosclerosis* **238**, 304-312 (2015).
150. Gundersen, K.E. *et al.* Two novel missense mutations in the LDL receptor gene causing familial hypercholesterolemia. *Clin. Genet.* **49**, 85-87 (1996).
151. Giesel, J., Holzem, G. & Oette, K. Screening for mutations in exon 4 of the LDL receptor gene in a German population with severe hypercholesterolemia. *Hum. Genet.* **96**, 301-304 (1995).

152. Cenarro, A. *et al.* Identification of recurrent and novel mutations in the LDL receptor gene in Spanish patients with familial hypercholesterolemia. Mutations in brief no. 135. Online. *Hum. Mutat.* **11**, 413 (1998).
153. Alonso, R. *et al.* Genetic diagnosis of familial hypercholesterolemia using a DNA-array based platform. *Clin. Biochem.* **42**, 899-903 (2009).
154. Webb, J.C. *et al.* Characterization of mutations in the low density lipoprotein (LDL)-receptor gene in patients with homozygous familial hypercholesterolemia, and frequency of these mutations in FH patients in the United Kingdom. *J. Lipid. Res.* **37**, 368-81 (1996).
155. Leitersdorf, E., Van der Westhuyzen, D.R., Coetzee, G.A. & Hobbs, H.H. Two common low density lipoprotein receptor gene mutations cause familial hypercholesterolemia in Afrikaners. *J. Clin. Invest.* **84**, 954-961 (1989).
156. Maruyama, T. *et al.* Common mutations in the low-density-lipoprotein-receptor gene causing familial hypercholesterolemia in the Japanese population. *Arterioscler. Thromb. Vasc. Biol.* **15**, 1713-1718 (1995).
157. Leitersdorf, E., Tobin, E.J., Davignon, J. & Hobbs, H.H. Common low-density lipoprotein receptor mutations in the French Canadian population. *J. Clin. Invest.* **85**, 1014-1023. (1990).
158. Vasli, N. *et al.* Identification of a homozygous missense mutation in LRP2 and a hemizygous missense mutation in TSPYL2 in a family with mild intellectual disability. *Psychiatr. Genet.* **26**, 66-73 (2016).
159. Schrauwen, I. *et al.* Broadening the phenotype of LRP2 mutations: a new mutation in LRP2 causes a predominantly ocular phenotype suggestive of Stickler syndrome. *Clin. Genet.* **86**, 282-286 (2014).
160. Guipponi, M. *et al.* The transmembrane serine protease (TMPRSS3) mutated in deafness DFNB8/10 activates the epithelial sodium channel (ENaC) in vitro. *Hum. Mol. Genet.* **11**, 2829-2836 (2002).
161. Wattenhofer, M. *et al.* Mutations in the TMPRSS3 gene are a rare cause of childhood nonsyndromic deafness in Caucasian patients. *J. Mol. Med.* **80**, 124-131 (2002).
162. Finberg, K.E. *et al.* Mutations in TMPRSS6 cause iron-refractory iron deficiency anemia (IRIDA). *Nat. Genet.* **40**, 569-571 (2008).
163. Silvestri, L. *et al.* Molecular mechanisms of the defective hepcidin inhibition in TMPRSS6 mutations associated with iron-refractory iron deficiency anemia. *Blood* **113**, 5605-5608 (2009).
164. Nicolas, G. *et al.* SORL1 rare variants: a major risk factor for familial early-onset Alzheimer's disease. *Mol. Psychiatry* **21**, 831-836 (2016).
165. Bellenguez, C. *et al.* Contribution to Alzheimer's disease risk of rare variants in TREM2, SORL1, and ABCA7 in 1779 cases and 1273 controls. *Neurobiol. Aging* (2017).
166. Lacour, M. *et al.* Causative Mutations and Genetic Risk Factors in Sporadic Early Onset Alzheimer's Disease Before 51 Years. *J. Alzheimers Dis.* **71**, 227-243 (2019).
167. Bjarnadottir, K. *et al.* Recent SORL1 missense variant causing a SORLA maturation defect is associated with Alzheimer’s disease. **In preparation**(2023).
168. Scheltens, P. *et al.* Alzheimer's disease. *Lancet* (2021).

169. Leren, T.P. *et al.* Molecular genetics of familial hypercholesterolaemia in Norway. *J. Intern. Med.* **241**, 185-194 (1997).
170. Esser, V., Limbird, L.E., Brown, M.S., Goldstein, J.L. & Russell, D.W. Mutational analysis of the ligand binding domain of the low density lipoprotein receptor. *J. Biol. Chem.* **263**, 13282-13290 (1988).
171. Takahashi, M. *et al.* A novel mutation in exon 2 of the low-density lipoprotein-receptor gene in a patient with homozygous familial hypercholesterolemia. *Clin. Genet.* **59**, 290-292 (2001).
172. Nauck, M.S. *et al.* Identification of recurrent and novel mutations in the LDL receptor gene in German patients with familial hypercholesterolemia. *Hum. Mutat.* **18**, 165-166 (2001).
173. Couture, P. *et al.* Identification of three mutations in the low-density lipoprotein receptor gene causing familial hypercholesterolemia among French Canadians. *Hum. Mutat.* **Suppl 1**, S226-231 (1998).
174. Lee, W.K. *et al.* Identification of a common low density lipoprotein receptor mutation (C163Y) in the west of Scotland. *J. Med. Genet.* **35**, 573-8 (1998).
175. Sundvold, H. *et al.* A common missense mutation (C210G) in the LDL receptor gene among Norwegian familial hypercholesterolemia subjects. *Hum. Mutat.* **7**, 70-71 (1996).
176. Ekström, U. *et al.* An individual with a healthy phenotype in spite of a pathogenic LDL receptor mutation (C240F). *Clin. Genet.* **55**, 332-339 (1999).
177. Holstege, H. *et al.* Characterization of pathogenic SORL1 genetic variants for association with Alzheimer's disease: a clinical interpretation strategy. *Eur. J. Hum. Genet.* **25**, 973-981 (2017).
178. Campion, D., Charbonnier, C. & Nicolas, G. SORL1 genetic variants and Alzheimer disease risk: a literature review and meta-analysis of sequencing data. *Acta. Neuropathol.* **138**, 173-186 (2019).
179. Proctor, R.A. Fibronectin: an enhancer of phagocyte function. *Rev Infect Dis* **9 Suppl 4**, S412-9 (1987).
180. Bork, P. Mobile modules and motifs. *Curr. Opin. Struct. Biol.* **2**, 413-421 (1992).
181. Main, A.L., Harvey, T.S., Baron, M., Boyd, J. & Campbell, I.D. The three-dimensional structure of the tenth type III module of fibronectin: an insight into RGD-mediated interactions. *Cell* **71**, 671-678 (1992).
182. Carr, P.A., Erickson, H.P. & Palmer, A.G., 3rd. Backbone dynamics of homologous fibronectin type III cell adhesion domains from fibronectin and tenascin. *Structure* **5**, 949-959 (1997).
183. Bencharit, S. *et al.* Structural insights into fibronectin type III domain-mediated signaling. *J. Mol. Biol.* **367**, 303-309 (2007).
184. Leahy, D.J., Hendrickson, W.A., Aukhil, I. & Erickson, H.P. Structure of a fibronectin type III domain from tenascin phased by MAD analysis of the selenomethionyl protein. *Science* **258**, 987-91 (1992).
185. Leahy, D.J., Aukhil, I. & Erickson, H.P. 2.0 Å crystal structure of a four-domain segment of human fibronectin encompassing the RGD loop and synergy region. *Cell* **84**, 155-164 (1996).
186. Campbell, I.D. & Spitzfaden, C. Building proteins with fibronectin type III modules. *Structure* **2**, 333-337 (1994).

187. Craig, D., Gao, M., Schulten, K. & Vogel, V. Tuning the mechanical stability of fibronectin type III modules through sequence variations. *Structure (Camb)* **12**, 21-30 (2004).
188. Hamill, S.J., Cota, E., Chothia, C. & Clarke, J. Conservation of folding and stability within a protein family: the tyrosine corner as an evolutionary cul-de-sac. *J. Mol. Biol.* **295**, 641-649 (2000).
189. Hemmingsen, J.M., Gernert, K.M., Richardson, J.S. & Richardson, D.C. The tyrosine corner: a feature of most Greek key beta-barrel proteins. *Protein Sci.* **3**, 1927-1937 (1994).
190. Batori, V., Koide, A. & Koide, S. Exploring the potential of the monobody scaffold: effects of loop elongation on the stability of a fibronectin type III domain. *Protein Eng.* **15**, 1015-1020 (2002).
191. Helenius, A. & Aebi, M. Intracellular functions of N-linked glycans. *Science* **291**, 2364-2369 (2001).
192. Hamming, O.J. *et al.* Crystal structure of interleukin-21 receptor (IL-21R) bound to IL-21 reveals that sugar chain interacting with WSXWS motif is integral part of IL-21R. *J. Biol. Chem.* **287**, 9454-9460 (2012).
193. Kotlarz, D. *et al.* Loss-of-function mutations in the IL-21 receptor gene cause a primary immunodeficiency syndrome. *J. Exp. Med.* **210**, 433-443 (2013).
194. Christensen, S.K. *et al.* Endosomal trafficking is required for glycosylation and normal maturationof the Alzheimer's-associated protein sorLA. *bioRxiv*, 2020.07.12.199885 (2020).
195. Potts, J.R. & Campbell, I.D. Structure and Function of Fibronectin Modules. *Matrix. Biol.* **15**, 313-320 (1996).
196. Olsen, J.G. & Kragelund, B.B. Who climbs the tryptophan ladder? On the structure and function of the WSXWS motif in cytokine receptors and thrombospondin repeats. *Cytokine Growth Factor Rev.* **25**, 337-341 (2014).
197. Holt, G.D., Pangburn, M.K. & Ginsburg, V. Properdin binds to sulfatide [Gal(3-SO4)beta 1-1 Cer] and has a sequence homology with other proteins that bind sulfated glycoconjugates. *J. Biol. Chem.* **265**, 2852-2855 (1990).
198. Fiete, D., Mi, Y., Oats, E.L., Beranek, M.C. & Baenziger, J.U. N-linked oligosaccharides on the low density lipoprotein receptor homolog SorLA/LR11 are modified with terminal GalNAc-4-SO4 in kidney and brain. *J. Biol. Chem.* **282**, 1873-1881 (2007).
199. Xu, W. *et al.* Seven novel mutations in the long isoform of the USH2A gene in Chinese families with nonsyndromic retinitis pigmentosa and Usher syndrome Type II. *Mol. Vis.* **17**, 1537-1552 (2011).
200. Dreyer, B. *et al.* Spectrum of USH2A mutations in Scandinavian patients with Usher syndrome type II. *Hum. Mutat.* **29**, 451 (2008).
201. Schäfer, M.K. *et al.* L1 syndrome mutations impair neuronal L1 function at different levels by divergent mechanisms. *Neurobiol. Dis.* **40**, 222-237 (2010).
202. Castelletti, F. *et al.* Mutations in FN1 cause glomerulopathy with fibronectin deposits. *Proc. Natl. Acad. Sci. U. S. A.* **105**, 2538-2543 (2008).
203. Albuisson, J. *et al.* Kallmann syndrome: 14 novel mutations in KAL1 and FGFR1 (KAL2). *Hum. Mutat.* **25**, 98-99 (2005).

204. Van Driest, S.L. *et al.* Myosin binding protein C mutations and compound heterozygosity in hypertrophic cardiomyopathy. *J. Am. Coll. Cardiol.* **44**, 1903-1910 (2004).
205. Sobrier, M.L. *et al.* Nine novel growth hormone receptor gene mutations in patients with Laron syndrome. *J. Clin. Endocrinol. Metab.* **82**, 435-437 (1997).
206. Rovelet-Lecrux, A. *et al.* Impaired SorLA maturation and trafficking as a new mechanism for SORL1 missense variants in Alzheimer disease. *Acta Neuropathol. Commun.* **9**, 196 (2021).
207. Souma, T. *et al.* Angiopoietin receptor TEK mutations underlie primary congenital glaucoma with variable expressivity. *J. Clin. Invest.* **126**, 2575-2587 (2016).
208. Nobile, S., Semple, R.K. & Carnielli, V.P. A novel mutation of the insulin receptor gene in a preterm infant with Donohue syndrome and heart failure. *J. Pediatr. Endocrinol. Metab.* **25**, 363-366 (2012).
209. Hosoe, J. *et al.* Structural Basis and Genotype-Phenotype Correlations of INSR Mutations Causing Severe Insulin Resistance. *Diabetes* **66**, 2713-2723 (2017).
210. MacFarlane, J.R. *et al.* Nine novel L1 CAM mutations in families with X-linked hydrocephalus. *Hum. Mutat.* **9**, 512-518 (1997).
211. Enberg, B. *et al.* Characterisation of novel missense mutations in the GH receptor gene causing severe growth retardation. *Eur. J. Endocrinol.* **143**, 71-76 (2000).
212. Longo, N. *et al.* Genotype-phenotype correlation in inherited severe insulin resistance. *Hum. Mol. Genet.* **11**, 1465-1475 (2002).
213. Piras, R. *et al.* Expanding the mutational spectrum of CRLF1 in Crisponi/CISS1 syndrome. *Hum. Mutat.* **35**, 424-433 (2014).
214. O'Marcaigh, A.S., Puck, J.M., Pepper, A.E., De Santes, K. & Cowan, M.J. Maternal mosaicism for a novel interleukin-2 receptor gamma-chain mutation causing X-linked severe combined immunodeficiency in a Navajo kindred. *J. Clin. Immunol.* **17**, 29-33 (1997).
215. Altare, F. *et al.* Interleukin-12 receptor beta1 deficiency in a patient with abdominal tuberculosis. *J. Infect. Dis.* **184**, 231-236 (2001).
216. Döffinger, R. *et al.* Partial interferon-gamma receptor signaling chain deficiency in a patient with bacille Calmette-Guerin and Mycobacterium abscessus infection. *J. Infect. Dis.* **181**, 379-384 (2000).
217. Moncada-Velez, M. *et al.* Partial IFN-gammaR2 deficiency is due to protein misfolding and can be rescued by inhibitors of glycosylation. *Blood* **122**, 2390-2401 (2013).
218. Germeshausen, M., Ballmaier, M. & Welte, K. MPL mutations in 23 patients suffering from congenital amegakaryocytic thrombocytopenia: the type of mutation predicts the course of the disease. *Hum. Mutat.* **27**, 296 (2006).
219. Triot, A. *et al.* Inherited biallelic CSF3R mutations in severe congenital neutropenia. *Blood* **123**, 3811-3817 (2014).
220. Du, Y.Z., Srivastava, A.K. & Schwartz, C.E. Multiple exon screening using restriction endonuclease fingerprinting (REF): detection of six novel mutations in the L1 cell adhesion molecule (L1CAM) gene. *Hum. Mutat.* **11**, 222-230 (1998).
221. Michaelis, R.C., Du, Y.Z. & Schwartz, C.E. The site of a missense mutation in the extracellular Ig or FN domains of L1CAM influences infant mortality and the severity of X linked hydrocephalus. *J. Med. Genet.* **35**, 901-904 (1998).

222. Marsh, A.P. *et al.* Mutations in DCC cause isolated agenesis of the corpus callosum with incomplete penetrance. *Nat. Genet.* **49**, 511-514 (2017).
223. Agrawal, P.B. *et al.* SPEG interacts with myotubularin, and its deficiency causes centronuclear myopathy with dilated cardiomyopathy. *Am. J. Hum. Genet.* **95**, 218-226 (2014).
224. Amyere, M. *et al.* Germline Loss-of-Function Mutations in EPHB4 Cause a Second Form of Capillary Malformation-Arteriovenous Malformation (CM-AVM2) Deregulating RAS-MAPK Signaling. *Circulation* **136**, 1037-1048 (2017).
225. McGee, T.L., Seyedahmadi, B.J., Sweeney, M.O., Dryja, T.P. & Berson, E.L. Novel mutations in the long isoform of the USH2A gene in patients with Usher syndrome type II or non-syndromic retinitis pigmentosa. *J. Med. Genet.* **47**, 499-506 (2010).
226. Hanchate, N.K. *et al.* SEMA3A, a gene involved in axonal pathfinding, is mutated in patients with Kallmann syndrome. *PLoS Genet.* **8**, e1002896 (2012).
227. Hermey, G., Sjogaard, S.S., Petersen, C.M., Nykjær, A. & Gliemann, J. Tumour necrosis factor a-converting enzyme mediates ectodomain shedding of Vps10p-domain receptor family members. *Biochem. J.* **395**, 285-293 (2006).
228. Fu, R. *et al.* In situ structural characterization of a recombinant protein in native Escherichia coli membranes with solid-state magic-angle-spinning NMR. *J. Am. Chem. Soc.* **133**, 12370-12373 (2011).
229. Watson, R.T. & Pessin, J.E. Functional cooperation of two independent targeting domains in syntaxin 6 is required for its efficient localization in the trans-golgi network of 3T3L1 adipocytes. *J Biol Chem* **275**, 1261-8 (2000).
230. Strøm, T.B., Tveten, K., Laerdahl, J.K. & Leren, T.P. Mutation G805R in the transmembrane domain of the LDL receptor gene causes familial hypercholesterolemia by inducing ectodomain cleavage of the LDL receptor in the endoplasmic reticulum. *FEBS Open Bio.* **4**, 321-327 (2014).
231. Strøm, T.B., Laerdahl, J.K. & Leren, T.P. Mutation p.L799R in the LDLR, which affects the transmembrane domain of the LDLR, prevents membrane insertion and causes secretion of the mutant LDLR. *Hum. Mol. Genet.* **24**, 5836-5844 (2015).
232. Schmidt, V. *et al.* SorLA/LR11 regulates processing of amyloid precursor protein via interaction with adaptors GGA and PACS-1. *J. Biol. Chem.* **282**, 32956-32964 (2007).
233. Bonifacino, J.S. The GGA proteins: adaptors on the move. *Nat. Rev. Mol. Cell Biol.* **5**, 23-32 (2004).
234. Nielsen, M.S. *et al.* The sortilin cytoplasmic tail conveys Golgi-endosome transport and binds the VHS domain of the GGA2 sorting protein. *EMBO J.* **20**, 2180-2190. (2001).
235. Puertollano, R. & Bonifacino, J.S. Interactions of GGA3 with the ubiquitin sorting machinery. *Nat Cell Biol* **6**, 244-51 (2004).
236. Jacobsen, L. *et al.* The sorLA cytoplasmic domain interacts with GGA1 and -2 and defines minimum requirements for GGA binding. *FEBS Lett.* **511**, 155-158 (2002).
237. Cramer, J.F. *et al.* GGA autoinhibition revisited. *Traffic* **11**, 259-273 (2010).
238. Herskowitz, J.H. *et al.* GGA1-mediated endocytic traffic of LR11/SorLA alters APP intracellular distribution and amyloid-beta production. *Mol. Biol. Cell* **23**, 2645-2657 (2012).

239. Dumanis, S.B. *et al.* Distinct Functions for Anterograde and Retrograde Sorting of SORLA in Amyloidogenic Processes in the Brain. *J. Neurosci.* **35**, 12703-12713 (2015).
240. Binkle, L., Klein, M., Borgmeyer, U., Kuhl, D. & Hermey, G. The adaptor protein PICK1 targets the sorting receptor SorLA. *Mol. Brain* **15**, 18 (2022).
241. Chen, W.-J., Goldstein, J.L. & Brown, M.S. NPXY, a sequence often found in cytoplasmic tails, is required for coated pit-mediated internalization of the low density lipoprotein receptor. *J. Biol. Chem.* **265**, 3116-3123 (1990).
242. Small, S.A. & Petsko, G.A. Retromer in Alzheimer disease, Parkinson disease and other neurological disorders. *Nat. Rev. Neurosci.* **16**, 126-132 (2015).
243. Fjorback, A.W. *et al.* Retromer binds the FANSHY sorting motif in sorLA to regulate amyloid precursor protein sorting and processing. *J. Neurosci.* **32**, 1467-1480 (2012).
244. Ghai, R. *et al.* Phox homology band 4.1/ezrin/radixin/moesin-like proteins function as molecular scaffolds that interact with cargo receptors and Ras GTPases. *Proc. Natl. Acad. Sci. U. S. A.* **108**, 7763-7768 (2011).
245. McNally, K.E. *et al.* Retriever is a multiprotein complex for retromer-independent endosomal cargo recycling. *Nat. Cell. Biol.* **19**, 1214-1225 (2017).
246. Huang, T.Y. *et al.* SNX27 and SORLA Interact to Reduce Amyloidogenic Subcellular Distribution and Processing of Amyloid Precursor Protein. *J. Neurosci.* **36**, 7996-8011 (2016).
247. Gill, R.L., Jr., Wang, X. & Tian, F. A membrane proximal helix in the cytosolic domain of the human APP interacting protein LR11/SorLA deforms liposomes. *Biochim. Biophys. Acta.* **1848**, 323-328 (2015).
248. Nielsen, M.S. *et al.* Sorting by the cytoplasmic domain of the amyloid precursor protein binding receptor SorLA. *Mol. Cell. Biol.* **27**, 6842-6851 (2007).
249. Burgert, T. *et al.* SORLA-dependent and -independent functions for PACS1 in control of amyloidogenic processes. *Mol. Cell. Biol.* **33**, 4308-4320 (2013).
250. Klinger, S.C. *et al.* Polarized trafficking of the sorting receptor SorLA in neurons and MDCK cells. *FEBS J* **283**, 2476-2493 (2016).
251. Bechler, M.E., de Figueiredo, P. & Brown, W.J. A PLA1-2 punch regulates the Golgi complex. *Trends Cell Biol.* **22**, 116-124 (2012).
252. Hirst, J. *et al.* The fifth adaptor protein complex. *PLoS Biol.* **9**, e1001170 (2011).
253. Madsen, P. *et al.* HSPA12A targets the cytoplasmic domain and affects the trafficking of the Amyloid Precursor Protein receptor SorLA. *Sci. Rep.* **9**, 611 (2019).
254. Lane, R.F., Gatson, J.W., Small, S.A., Ehrlich, M.E. & Gandy, S. Protein kinase C and rho activated coiled coil protein kinase 2 (ROCK2) modulate Alzheimer's APP metabolism and phosphorylation of the Vps10-domain protein, SorL1. *Mol. Neurodegener.* **5**, 62 (2010).
255. Herskowitz, J.H. *et al.* Rho kinase II phosphorylation of the lipoprotein receptor LR11/SORLA alters amyloid-b production. *J. Biol. Chem.* **286**, 6117-6127 (2011).
256. Bellenguez, C. *et al.* New insights into the genetic etiology of Alzheimer's disease and related dementias. *Nat. Genet.* **54**, 412-436 (2022).
257. Cuccaro, M.L. *et al.* SORL1 mutations in early- and late-onset Alzheimer disease. *Neurol. Genet.* **2**, e116 (2016).

258. Böhm, C. *et al.* SorLA signaling by regulated intramembrane proteolysis. *J. Biol. Chem.* **281**, 14547-14553 (2006).
259. Peterson, T.A. *et al.* DMDM: domain mapping of disease mutations. *Bioinformatics* **26**, 2458-2459 (2010).
260. Sievers, F. *et al.* Fast, scalable generation of high-quality protein multiple sequence alignments using Clustal Omega. *Mol. Syst. Biol.* **7**, 539 (2011).
261. Goujon, M. *et al.* A new bioinformatics analysis tools framework at EMBL-EBI. *Nucleic Acids Res.* **38**, W695-9 (2010).
262. Kumar, S., Stecher, G. & Tamura, K. MEGA7: Molecular Evolutionary Genetics Analysis Version 7.0 for Bigger Datasets. *Mol. Biol. Evol.* **33**, 1870-1874 (2016).
263. Waterhouse, A.M., Procter, J.B., Martin, D.M., Clamp, M. & Barton, G.J. Jalview Version 2--a multiple sequence alignment editor and analysis workbench. *Bioinformatics* **25**, 1189-1191 (2009).
264. Cullen, P.J. & Steinberg, F. To degrade or not to degrade: mechanisms and significance of endocytic recycling. *Nat. Rev. Mol. Cell. Biol.* **19**, 679-696 (2018).
265. Rudenko, G. & Deisenhofer, J. The low-density lipoprotein receptor: ligands, debates and lore. *Curr Opin Struct Biol* **13**, 683-9 (2003).
266. Beglova, N. & Blacklow, S.C. The LDL receptor: how acid pulls the trigger. *Trends Biochem. Sci.* **30**, 309-317 (2005).
267. Bouvard, D., Pouwels, J., De Franceschi, N. & Ivaska, J. Integrin inactivators: balancing cellular functions in vitro and in vivo. *Nat. Rev. Mol. Cel. Biol.*, 430-442 (2013).
268. Aricescu, A.R. *et al.* Structure of a tyrosine phosphatase adhesive interaction reveals a spacer-clamp mechanism. *Science*, 1217-1220 (2007).
269. Beglova, N., Jeon, H., Fisher, C. & Blacklow, S.C. Cooperation between fixed and low pH-inducible interfaces controls lipoprotein release by the LDL receptor. *Mol. Cell* **16**, 281-292 (2004).
270. Januliene, D. *et al.* Hidden Twins: SorCS Neuroreceptors Form Stable Dimers. *J. Mol. Biol.* **429**, 2907-2917 (2017).
271. Leloup, N., Chataigner, L.M.P. & Janssen, B.J.C. Structural insights into SorCS2-Nerve Growth Factor complex formation. *Nat. Commun.* **9**, 2979 (2018).
272. Leloup, N. *et al.* Low pH-induced conformational change and dimerization of sortilin triggers endocytosed ligand release. *Nat. Commun.* **8**, 1708 (2017).
273. Januliene, D. *et al.* Acidic Environment Induces Dimerization and Ligand Binding Site Collapse in the Vps10p Domain of Sortilin. *Structure* **25**, 1809-1819 e3 (2017).
274. Yabe-Wada, T., Matsuba, S., Unno, M. & Onai, N. Crystal structure of the ligand-free form of the Vps10 ectodomain of dimerized Sortilin at acidic pH. *FEBS Lett.* **592**, 2647-2657 (2018).
275. Quistgaard, E.M. *et al.* Revisiting the structure of the Vps10 domain of human sortilin and its interaction with neurotensin. *Protein Sci.* **23**, 1291-1300 (2014).
276. Moore, J.O., Lemmon, M.A. & Ferguson, K.M. Dimerization of Tie2 mediated by its membrane-proximal FNIII domains. *Proc. Natl. Acad. Sci. U. S. A.* **114**, 4382-4387 (2017).
277. Schmidt, V. *et al.* Quantitative modelling of amyloidogenic processing and its influence by SORLA in Alzheimer's disease. *EMBO J.* **31**, 187-200 (2011).

278. Eggert, S. *et al.* Dimerization leads to changes in APP (amyloid precursor protein) trafficking mediated by LRP1 and SorLA. *Cell. Mol. Life Sci.* **75**, 301-322 (2018).
279. Pietila, M. *et al.* SORLA regulates endosomal trafficking and oncogenic fitness of HER2. *Nat. Commun.* **10**, 2340 (2019).
280. Blacklow, S.C. Versatility in ligand recognition by LDL receptor family proteins: advances and frontiers. *Curr. Opin. Struct. Biol.* **17**, 419-426 (2007).
281. Huang, S., Henry, L., Ho, Y.K., Pownall, H.J. & Rudenko, G. Mechanism of LDL binding and release probed by structure-based mutagenesis of the LDL receptor. *J. Lipid Res.* **51**, 297-308 (2010).
282. Al-Akhrass, H. *et al.* A feed-forward loop between SorLA and HER3 determines heregulin response and neratinib resistance. *Oncogene* **40**, 1300-1317 (2021).
